# Outcomes following major dysvascular lower limb amputation: an updated systematic review

**DOI:** 10.1186/s12872-026-06377-5

**Published:** 2026-08-06

**Authors:** Shashank Ghai, Sander L. Hitzig, Maha Edrees, Amanda L. Mayo, Virginie Blanchette, Natalie Habra, Audrey Zucker-Levin, Diana Zidarov

**Affiliations:** 1 Department of Political, Historical, Religious and Cultural Studies, Karlstads Universitet, Karlstad, Sweden; 2 Centre for Societal Risk Research, Karlstads Universitet, Karlstad, Sweden; 3 Department of Occupational Science and Occupational Therapy, Temerty Faculty of Medicine, University of Toronto, Toronto, Canada; 4 St. John’s Rehab Research Program, Sunnybrook Research Institute, Sunnybrook Health Sciences Centre, Toronto, Canada; 5 Rehabilitation Sciences Institute, Temerty Faculty of Medicine, University of Toronto, Toronto, Canada; 6 Dalla Lana School of Public Health, University of Toronto, Toronto, Canada; 7 Physical Medicine and Rehabilitation Department, King Fahad Medical City, Riyadh, Saudi Arabia; 8 Division of Physical Medicine and Rehabilitation, Department of Medicine, Sunnybrook Health Sciences Centre, Toronto, Canada; 9 Centre for Quality Improvement and Patient Safety (CQuIPS), Temerty Faculty of Medicine, University of Toronto, Toronto, Canada; 10 VITAM - Centre de Recherche en Santé Durable, Centre Intégré Universitaire de Santé Et Services Sociaux de La Capitale-Nationale, Québec, Canada; 11 Department of Human Kinetics and Podiatric Medicine, Université du Québec À Trois-Rivières, Trois-Rivières, Canada; 12 Faculté de Médecine, Université de Montréal, Montréal, Canada; 13 Centre de Recherche Interdisciplinaire en Réadaptation (CRIR), Institut Universitaire Sur La Réadaptation en Déficience Physique de Montréal (IURDPM), Centre Intégré Universitaire de Santé Et de Services Sociaux du Centre-Sud-de-L’Île-de-Montréal, Montréal, Canada; 14 School of Rehabilitation Science, University of Saskatchewan, Saskatoon, Canada; 15 École de Réadaptation, Faculté de Médecine, Université de Montréal, Montréal, Québec Canada

**Keywords:** Amputation, Lower extremity, Outcome assessment, Dysvascular amputation, Systematic review

## Abstract

**Background:**

Adults with major lower limb amputation (LLA) due to dysvascular etiology often experience diverse health outcomes. However, the absence of standardized outcome reporting limits the ability to synthesize evidence, evaluate care models, and develop consistent clinical pathways. This review updates a systematic review on outcome measures and indicators reported in this population.

**Methods:**

A systematic review update was conducted across six databases (MEDLINE, EMBASE, EBM Reviews, PsycINFO, Web of Science, and CINAHL) from 20 April 2017 to 1 December 2022. All study designs were eligible; however, the articles needed to report outcome measures and indicators on at least 50% of the adult population with major dysvascular LLAs. The extracted outcome measures and indicators were organized according to Dodd’s framework and then reclassified alongside those reported in the study by Ambler and colleagues. The pilot phase of data extraction was carried out independently by multiple reviewers, after which a single reviewer completed the data extraction.

**Results:**

Of the 12,557 articles, 160 studies reporting data on 228,116 individuals with major dysvascular LLA met the inclusion criteria. Combining these with the 440 studies from the previous review, 2,466 outcome measures and indicators were categorized in five core areas: life impact (25.7%), physiological/clinical (23.4%), death (18.7%), resource use (17.6%), and adverse events (14.6%). In terms of the outcome domains, mortality/survival was the most commonly reported domain, followed by physical functioning (e.g. prosthesis fit, use), skin (e.g., stump wound healing), nervous system outcomes (e.g., residual limb pain), need for further interventions (e.g., prescription of medications), hospital outcomes (e.g., duration of stay, discharge), and adverse events.

**Conclusion:**

This study builds on a previous review by identifying and categorizing outcome measures and indicators in adults with major dysvascular LLA, highlighting inconsistencies in outcome reporting across studies. The absence of a standardized set of outcome measures and indicators limits the ability to conduct meta-analyses or systematic reviews, evaluate the effectiveness of care models, and establish clinical pathways. Developing a core outcome set tailored to the needs of individuals with dysvascular LLA could help address these challenges by promoting consistency in outcome reporting and enabling meaningful comparisons across studies. Ultimately, this could contribute to improving the quality, efficiency, and person-centeredness of care.

**Trial registration:**

PROSPERO (CRD42023388570).

**Supplementary Information:**

The online version contains supplementary material available at 10.1186/s12872-026-06377-5.

## Introduction

A major lower limb amputation (LLA), defined as an amputation performed at the ankle level or above, is a life-altering event with profound consequences at both individual and societal levels [1, 2]. This is particularly true for major LLAs resulting from dysvascular complications, which are primarily caused by peripheral vascular disease [3]. The prevalence of peripheral vascular disease and its associated complications, including dysvascular LLAs, is rising worldwide [4]. Globally, the majority of major LLAs result from dysvascular complications [5–7]. Large cohort studies have reported that approximately 96% of individuals with LLAs had peripheral arterial disease as the primary underlying etiology, followed by diabetes, itself a form of vascular disease [8, 9].

Major dysvascular LLAs impose a significant burden on healthcare systems worldwide due to the high cost of associated medical care. Studies across different healthcare systems have consistently demonstrated substantial economic burden, with initial hospitalization costs alone representing a significant portion of total healthcare expenditure, excluding physician compensation and follow-up costs [10–13]. Beyond the economic burden, major dysvascular LLAs are linked to poor clinical outcomes, including high rates of prolonged hospitalization, readmission, re-amputation, and mortality [10, 14–16]. International data indicate that patients undergoing major dysvascular LLAs have a median hospital stay of approximately 19 days, a 19 to 27% readmission rate within 30 days for subsequent amputations, and a 30-day mortality rate as high as 8% [10, 14, 17–21].

Another challenge for healthcare systems and clinical research involving people with dysvascular amputation is the variability in the reporting of outcome measures and indicators. Outcome measures refer to the tools and instruments used to assess the impact of an intervention or program over a defined period [22]. Outcome indicators, on the other hand, are measurable variables or attributes that capture and reflect the clinical parameters under evaluation [23]. A notable lack of consensus exists internationally regarding both program and patient-reported outcomes in amputee rehabilitation services, with many programs across different healthcare systems relying on informal, non-standardized outcome measures [24–26]. This lack of consensus compromises patient care quality and rehabilitation outcomes, while also limiting the ability to compare outcomes between different healthcare services internationally to identify more effective care models and clinical pathways for the LLA population [27]. Moreover, this lack of consensus limits the feasibility of conducting evidence-based syntheses, such as systematic reviews and meta-analyses, which impedes the development of standardized clinical practice guidelines and reduces the utility of research findings for informing clinical decision-making and patient care [24, 28]. Core outcome sets (COS) may help address these issues, by allowing relevant stakeholders to collaborate and reach a consensus on the key outcome measures and indicators that should be measured and reported in all clinical trials related to a specific health condition [29]. The first step in developing a condition-specific COS is to conduct a systematic review of the existing literature to identify and categorize outcome measures and indicators reported in previous studies. This ensures that the development process is grounded in current evidence and helps inform subsequent phases, including qualitative data collection to capture perspectives from patients and healthcare professionals, and the use of a Delphi survey to establish consensus on the COS [30].

To the best of our knowledge, only one systematic review conducted by Ambler, et al. [31], has categorized the outcome measures and indicators for adults with dysvascular LLAs (drawing on 440 studies) using Dodd's taxonomy and developed a COS for LLA through a Delphi consensus process involving multiple stakeholder groups [32]. Dodd’s taxonomy provides a standardized hierarchical framework for organizing outcomes across clinical research. It groups outcomes into five core areas, namely death, physiological/clinical outcomes, life impact, resource use, and adverse events, further subdivided into domains and subdomains. Its comprehensive and granular structure facilitates consistent classification, comparison, and synthesis of outcomes across studies, thereby supporting the development of COS [32]. While Ambler, et al. [31] represents an important foundation for outcome standardization in this population, several methodological considerations warrant attention and justify an updated, comprehensive review. First, the review included studies published only up to April 2017, and a substantial number of studies have been published since. Given the rapidly evolving nature of amputation rehabilitation research and clinical practices, systematic reviews in this field require regular updating to ensure clinical recommendations remain current and evidence-based [33]. Second, as knowledge and practice in this field continue to advance, so too does our understanding of which stakeholders are best positioned to inform outcome selection. Stakeholder engagement in Ambler, et al. [31]’s COS development showed limited representation from certain key professional groups, particularly rehabilitation professionals (e.g., physiotherapists) and individuals with lived experience of amputation, whose perspectives are increasingly recognized as essential in shaping meaningful, patient-centered outcomes [34]. This is particularly relevant given that individuals with major LLA typically experience extended rehabilitation trajectories and require ongoing community-based support [35, 36]. Third, the review presented additional limitations, including limited specificity regarding the reported outcomes and the source studies from which they were extracted. Furthermore, contextual classification of outcomes, such as whether they pertain to acute care, rehabilitation, or community settings, was not clearly addressed. This contextual information has become increasingly relevant as each phase of care following major dysvascular LLA involves distinct clinical priorities, patient needs, and expected outcomes [37]. Understanding the context in which outcomes are reported can inform when and how they should be measured, thereby enhancing their relevance for clinical practice and research.

In light of these considerations, the objective of the present review was to update the findings of Ambler, et al. [31] which identified and categorized outcome measures and indicators reported in adults with major dysvascular LLA. Specifically, this review aimed to systematically map and categorize outcome measures and indicators for adults with major dysvascular LLA, using Dodd’s framework, and to explicitly link outcomes to their source studies and clinical contexts. By focusing on studies published after April 2017, this review aims to provide a more comprehensive analysis of reported outcomes and to refine the categorization process to support its clarity and utility in both clinical practice and research.

## Methods

### Systematic review

The systematic review was conducted following established systematic review methodology and reported in accordance with the Preferred Reporting Items for Systematic Reviews and Meta-Analyses (PRISMA 2020) guidelines [38]. The review protocol was pre-registered in PROSPERO (CRD42023388570). A PRISMA 2020 checklist is provided in Supplementary Table 1.

### Eligibility criteria

Articles were considered eligible for inclusion if they met the following criteria: 1) at least 50% of the study population consisted of individuals with major LLAs (i.e., ankle level and above) resulting from dysvascular etiologies (i.e., secondary to complications of peripheral vascular disease [39]); 2) amputations occurred in adults (aged 18 years or older); 3) the study reported outcome measures and indicators, and 4) the articles were published in English, German, or French after April 20, 2017 (the completion date of the search by Ambler, et al. [31]).

Articles were excluded if they included populations with pediatric-onset or congenital limb loss, minor amputations (i.e., below the ankle), or amputations due to non-dysvascular etiologies (e.g., cancer or trauma). Studies reporting solely on amputation rate trends (e.g., incidence, prevalence, temporal patterns) were excluded, as this review focused on evaluating clinical and functional outcome measures and indicators in individuals with dysvascular LLA rather than population-level epidemiological trends. Non-peer-reviewed literature, including conference proceedings, abstracts, editorials, posters, theses, systematic reviews, meta-analyses, and narrative reviews, were also excluded. Additionally, studies were excluded if outcome measures and indicators specific to the dysvascular etiology of the amputation could not be clearly identified.

### Information sources and search strategy

Six major online databases: MEDLINE, EMBASE, EBM Reviews, PsycINFO, Web of Science, and CINAHL were searched from April 2017 to December 2022 using MeSH terms (subject headings) and relevant keywords. In addition, Google Scholar was searched to identify potentially relevant peer-reviewed articles that may not have been indexed in the selected databases. Records identified through Google Scholar were screened according to the same predefined eligibility criteria, and non-peer-reviewed sources were excluded.

The updated search strategy (Supplementary Table 2) was developed in collaboration with the research team and validated by an academic librarian from Université de Montréal. It followed Cochrane guidelines for updating reviews using an incremental approach [40]. While retaining information from the original search conducted by Ambler, et al. [31], the updated strategy aimed to capture newly published data on major LLAs associated with peripheral vascular disease that had been published since the previous review.

### Study selection process

The Covidence systematic review platform was used to manage the article screening process for this systematic review [41]. To ensure methodological rigor, a pilot phase was first conducted on the initial 200 titles and abstracts, which were independently screened by two reviewers (SG, MBE) to ensure consistent application of the inclusion criteria. Following the pilot, the remaining titles and abstracts were independently screened by the same two reviewers, achieving a 92% agreement rate. All full-text articles were also independently screened by the same pair. Discrepancies were resolved through discussion, and when consensus could not be reached, a third reviewer (DZ) was consulted to finalize the selection.

### Data extraction

Data for adults with major dysvascular LLA was extracted using a customized data extraction sheet developed by two reviewers (SG, DZ) and approved by the research team. The extraction sheet captured key categories of information, including general study characteristics (e.g., author, publication year, study design), participant characteristics (e.g., age, sex, cause of amputation, time since amputation, amputation laterality), research context (acute care, rehabilitation or community setting), and outcome measures and indicators (e.g., total number, name, definition, and type).

Outcome measures and indicators were organized and classified using the classification framework developed by Dodd and colleagues [32], which was also used by Ambler, et al. [31]. This taxonomy was developed in response to the lack of a comprehensive and standardized system for classifying outcomes in clinical research, where inconsistent terminology and reporting have historically limited comparability across studies. It was selected for its detailed structure, extensive coverage, and suitability as a practical classification tool for organizing trial outcomes into consistent and searchable categories, characteristics often lacking in other systems such as the Patient-Reported Outcomes Measurement Information System [42], Outcome Measures in Rheumatology [43], and the Nursing Outcomes Classification [44]. While these alternative frameworks prioritize patient-centered outcomes, they often lack the specificity and depth required for disease-specific studies. In contrast, Dodd’s framework provides a standardized and structured approach to outcome classification, which can support systematic literature synthesis and guide COS development processes, including Delphi surveys.

**Fig. 1:**
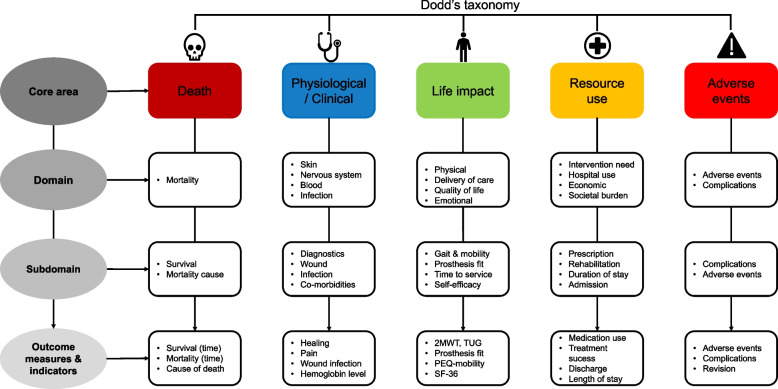
Overview of Dodd's outcome taxonomy. Dodd's framework provides a hierarchical structure for classifying outcomes in clinical research, organized into five core areas: death, physiological/clinical outcomes, life impact, resource use, and adverse events. Each core area is subdivided into domains and subdomains, within which specific outcome measures and indicators reported in the literature can be classified. This figure presents a simplified representation of the taxonomy. Not all domains or subdomains are shown

Dodd's taxonomy comprises five core areas (death, physiological/clinical outcomes, impact on life, resource use, and adverse events) and 38 outcome domains, further subdivided into subdomains. Each subdomain includes specific outcome measures, the tools or instruments used for assessment (e.g. the Timed Up and Go test), and outcome indicators, the specific variables or operational definitions those tools capture or that are derived directly from clinical records (e.g. time in seconds, mortality rate). The overview of Dodd's outcome taxonomy is illustrated in Fig. 1. The framework was applied to update and expand the work of Ambler, et al. [31] by classifying outcome measures and indicators for individuals undergoing major LLAs due to peripheral vascular disease, with the ultimate aim of informing COS development for this population.

Data extraction began with a pilot phase in which SG and two other research assistants independently extracted data from a random sample of 40 studies (25% of the total 160 included studies), with an inter-rater agreement of 85%. Discrepancies were resolved through consensus to ensure consistency in the application of the extraction criteria. Following the pilot, SG independently completed the data extraction for the remaining studies and classified the outcome measures and indicators according to Dodd’s framework.

### Quality assessment

As the primary aim of this review was to identify outcome measures and indicators reported in studies of major LLAs due to peripheral vascular disease and related complications, the included studies were not assessed for methodological quality or risk of bias.

### Results synthesis

Following the extraction of all identified outcome measures and indicators from the included studies, the research team categorized them using the framework proposed by Dodd and colleagues [32]. Outcome measures and indicators were systematically organized into the following categories: core areas, outcome domains, outcome subdomains, outcome measures and indicators, and the type of outcome assessment (e.g., patient-reported, clinician-reported, or performance-based). For consistency and comparability, the outcome measures and indicators previously reported by Ambler, et al. [31] in their supplementary file were also reclassified using Dodd’s framework. To ensure classification validity, the same predefined hierarchical criteria were applied throughout, and ambiguous decisions were discussed with a senior experienced researcher (DZ).

Given the large number of unique outcome measures and indicators identified (n = 933), and to enhance the clarity and interpretability of the results, only those outcome measures and indicators reported with a frequency of ≥ 1% were highlighted in the main text of the Results section. This threshold was selected to focus on the most commonly used outcome measures and indicators while maintaining a comprehensive overview of outcome reporting patterns. A complete list of all outcome measures and indicators, including those reported in < 1% of cases, is provided in Supplementary Table 3.

## Results

### Selection procedure

**Fig. 2:**
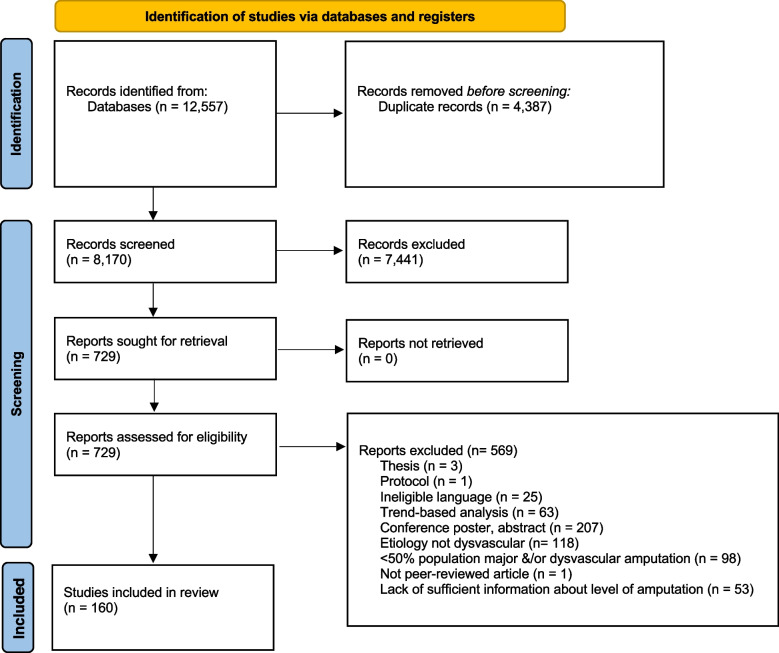
PRISMA flow chart developed through an R package Shiny app by Haddaway, et al. [45]

The updated search identified 12,557 abstracts. After removing duplicates, 8,170 unique titles and abstracts were screened. Of these, 729 full-text articles were assessed for eligibility, resulting in the inclusion of 160 studies. The study selection process is detailed in the PRISMA flowchart (Fig. 2).

### Characteristics of sources of evidence

#### Study design

Study designs varied across 160 included studies: 89 (55.6%) were retrospective cohort studies [46–134], 20 (12.5%) were cross-sectional studies [135–154], 15 (9.4%) were case studies [155–169], 14 (8.8%) were prospective cohort studies [170–183], 6 (3.8%) were randomized controlled trials [184–189], 3 (1.9%) used mixed methods studies [190–192], 3 (1.9%) were case series [193–195], 2 (1.3%) were randomized crossover trials [196, 197], 2 (1.3%) were case–control studies [198, 199], 2 (1.3%) were secondary analyses of cross-sectional studies [200, 201], 2 (1.3%) were quasi-experimental studies (pre- vs. post-test) [202, 203], 1 (0.6%) was a qualitative descriptive study [204], and 1 (0.6%) was a secondary analysis of a randomized control trial [205]. The detailed characteristics of all included studies are presented in Supplementary Table 4.

In comparison, Ambler, et al. [31], which included 440 studies, reported the following distribution: 292 (66.4%) were retrospective case series or cohort studies, 100 (22.7%) were prospective cohort studies, 20 (4.5%) were randomized controlled trials, 16 (3.6%) were case reports, 9 (2%) were non-randomized controlled trials, 2 (0.5%) included both prospective and retrospective cohorts, and 1 (0.2%) was qualitative.

### Description of the sample

The 160 included studies reported data on a total of 228,116 individuals. A detailed description of participant characteristics is provided in Table 1. Five studies did not report the sex distribution of their population [63, 118, 146, 153, 205], and seven studies did not report participants’ age [55, 80, 86, 118, 120, 132, 154].

**Table 1:** Demographic and clinical characteristics of participants across the 160 included studies

Characteristics	Mean ± SD (range), or N	Number of studies with missing or incomplete reporting; [reference]
Total participants	228,116	-
Sex		
Female	73,036	5; [63, 118, 146, 153, 205]
Male	153,819	
Unknown sex	4	
Age (years)	66.6 ± 9.1 (range: 18 to 101)	7; [55, 80, 86, 118, 120, 132, 154]
Etiology of LLA		
Dysvascular causes	156,430	-
Dysvascular (≥ 50%) + others	71,686	
Time since amputation (years)	5.8 (range: 0–27)	122; [46–69, 71–83, 85–96, 98–128, 130, 132–135, 140, 141, 143, 146, 149, 151, 153, 154, 156–160, 162, 169–182, 184, 185, 188, 189, 193, 195, 198, 200, 202]
Laterality of LLA		
Unilateral	86,099	50; [49, 51, 54, 55, 57, 58, 60, 72, 73, 75, 79, 82, 83, 85, 86, 89, 92–94, 96, 99, 101–103, 105–109, 112–114, 116, 120, 124, 125, 132, 135, 136, 143, 147, 148, 151, 152, 179, 181, 184, 198, 200, 202]
Bilateral	1,215	
Level of LLA		
TTA	116,646	31; [48, 49, 52, 57, 62, 76, 79, 80, 84, 89, 93, 96, 100, 102, 106, 111, 113, 114, 117, 130, 133–135, 154, 164, 171, 178–181, 183]
TFA	64,531	
TFA/TKA	14,461	
TTA/TFA	2,201	
TTA/Syme's	350	
TKA/KDA	406	
Gritti-Stokes	95	
Hip disarticulation	9	
Hemipelvectomy	1	

*TTA* Transtibial, *TFA* Transfemoral, *TKA* Through-knee, *KDA* Knee disarticulation

Several studies included comparator groups. Three studies reported data on non-amputee controls with medical conditions: 7,787 controls with femoral neck fractures, 10 controls with diabetes, 1,070 controls with peripheral vascular disease and 550 adults with trauma- or cancer-related pathologies [77, 94, 146]. Five studies included 56 healthy controls [137, 145, 146, 198, 199], two studies included 146 caregivers of amputees [97, 171], and one included seven health care professionals [97].

The review by Ambler, et al. [31] did not report the total number of participants across their 440 included studies but noted that the majority were male (61.2%) with a weighted mean age of 73.5 years (range: 34–93.5 years).

### Lower limb amputation characteristics

Details regarding amputation etiology, time since amputation, laterality, and level of amputation are summarized in Table 1.

### Context of the study

Of the 160 included studies, 83 (51.9%) were conducted in acute care settings [46–49, 51–58, 60–63, 68, 72–77, 79–86, 88, 91–96, 98–100, 102–104, 106–117, 119–123, 125–128, 132–134, 136, 149, 155, 156, 159, 161, 164, 165, 169, 172–174, 177, 185, 198], 51 (31.9%) in rehabilitation settings [59, 64, 65, 67, 69–71, 78, 87, 90, 97, 101, 105, 118, 124, 129–131, 135, 137, 140, 141, 143, 145–148, 150, 151, 153, 158, 160, 162, 166, 168, 170, 175, 176, 178, 180, 184, 187, 188, 193, 197, 199–204], and 19 (11.9%) in community-based settings [66, 89, 138, 139, 142, 144, 152, 154, 171, 181–183, 186, 189–192, 196, 205]. Six studies (3.8%) spanned both acute care and rehabilitation settings [50, 157, 163, 167, 194, 195], and one study (0.6%) involved both acute care and community-based settings [179].

### Classification of outcome measures and indicators using Dodd's framework

Outcome measures and indicators were classified for all 160 studies included in the present review, as well as for the 440 studies included in the review by Ambler, et al. [31], using Dodd’s classification framework [32]. This framework organizes outcomes hierarchically into five core areas, which are further divided into domains, subdomains, and finally into specific measures and indicators. In Ambler, et al. [31]’s study, the outcome measures and indicators were reported in their supplementary file but were not linked to specific studies and were not classified into subdomains. To enable comprehensive comparison, the present study reclassified all outcome measures and indicators from both reviews according to Dodd’s hierarchical structure.

All five core areas of Dodd’s framework [32] were used to categorize 1,004 outcome measures and indicators from the current review and 1,462 from Ambler, et al. [31]’s review. Frequency analysis of these 2,466 (i.e., 1,004 + 1,462) outcome measures and indicators yielded 933 unique measures and indicators. Of these, 521 were identified exclusively in the present update, 345 were identified exclusively in Ambler et al. [31] and 67 were shared across both reviews, representing an overlap of 7.2%.

### Distribution across core areas

Among these, the most frequently represented core area was life impact (298 unique outcome measures and indicators), followed by physiological/clinical (270), resource use (171), adverse events (117), and death (77).

### Distribution of outcome domains across core areas

**Fig. 3:**
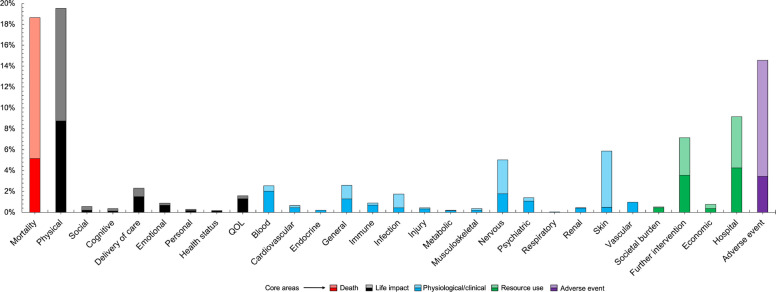
Distribution (%) of core outcome domains in the present study and in Ambler, et al. [31]. Dark shading indicates core outcomes from Ambler, et al. [31], light shading indicates updated core outcome domains from the present study. Percentages are cumulative (QOL: Quality of life)

A total of 29 domains were used to classify 135 subdomains. The list of prevalent domains within each core area is presented in Fig. 3 and summarized below:

### Core area 1: death

The domain of mortality/survival within the death core area accounted for 457 outcome measures and indicators (18.7%).

### Core area 2: life impact

This core area comprised eight outcome domains. The most frequently reported domain within this core area was physical functioning with 483 outcome measures and indicators (19.5%), followed by delivery of care 56 (2.3%), global quality of life 39 (1.6%), emotional functioning 22 (0.9%), social functioning 14 (0.6%), cognitive functioning 9 (0.3%), personal circumstances 7 (0.3%), and perceived health status 4 (0.16%).

### Core area 3: physiological/clinical

Within the physiological/clinical core area, 15 outcome domains were identified. The most frequently reported domain was skin and subcutaneous tissue with 145 outcome measures and indicators (5.9%), followed by nervous system 124 (5.0%), general 64 (2.6%), and blood and lymphatic system 64 (2.6%).

Other reported domains included: infection and infestation 43 (1.7%), psychiatric 35 (1.4%), immune system 22 (0.9%), vascular system 24 (1.0%), and cardiovascular 16 (0.6%).

Less frequently reported domains were injury and poisoning 11 (0.4%), musculoskeletal and connective tissue 9 (0.4%), endocrine 5 (0.2%), metabolic and nutritional 5 (0.2%), renal and urinary 11 (0.4%), and respiratory, thoracic and mediastinal 1 (0.04%).

### Core area 4: resource use

Four outcome domains were identified within the resource use core area. The most frequently reported was hospital-related with 227 outcome measures and indicators (9.2%), followed by need for further interventions 179 (7.2%), economic 19 (0.8%), and societal care/burden 13 (0.5%).

### Core area 5: adverse events/effects

Only one domain was identified in this core area: adverse events/effects, which accounted for 359 outcome measures and indicators (14.6%).

### Distribution across outcome subdomains

A total of 135 subdomains were used to classify the outcome measures and indicators identified in the included studies. The list of prevalent subdomains, organized by core area and domain, are presented in Table 2.

**Table 2:** Core areas, outcome domains and subdomains with percentage distribution of outcome measures/indicators

Core area	Outcome domain	Outcome subdomain	Outcome measures/indicators	
			Updated 160 studies)	Ambler et al. (440 studies)
Death (18.7%)	Mortality	Cause of mortality	0.12%	0.85%
		Mortality	0.81%	3.08%
		Survival	4.22%	9.57%
Life impact (25.7%)	Physical	Gait: Spatiotemporal	1.87%	0.36%
		Gait: Kinetics	0.16%	0.00%
		Gait: Kinematics	0.08%	0.00%
		Balance	0.28%	0.12%
		Balance confidence	0.24%	0.00%
		Endurance	0.04%	0.00%
		Energy efficiency	0.04%	0.00%
		Functional independence	0.61%	1.38%
		Impact of disease/conditional on activities of daily living	0.49%	0.12%
		Locomotion ability	0.69%	1.99%
		Mobility	1.50%	1.50%
		Flexibility	0.04%	0.00%
		Strength	0.04%	0.00%
		Mobility potential	0.28%	0.04%
		Physical activity	0.08%	0.04%
		Prosthesis fit	0.36%	2.84%
		Prosthesis use	1.18%	1.66%
		Prosthesis-related mobility	0.57%	0.73%
		Range of motion	0.16%	0.00%
		Weight bearing	0.04%	0.00%
	Social functioning	Social participation	0.12%	0.24%
		Social relationship	0.08%	0.00%
		Sexual function	0.00%	0.12%
	Cognitive functioning	Cognitive function	0.12%	0.24%
	Delivery of care	Time to receive services	0.57%	0.69%
		Feasibility	0.53%	0.00%
		Patient satisfaction	0.04%	0.08%
		Patient experience	0.04%	0.04%
		Patient preference	0.04%	0.00%
		Fidelity	0.04%	0.00%
		Reliability of scoring	0.04%	0.00%
		Global impression of change	0.04%	0.00%
		Accessibility, quality and adequacy of intervention	0.04%	0.00%
		Caregiver satisfaction	0.04%	0.00%
		Clinician satisfaction	0.04%	0.00%
		Barriers to care	0.04%	0.00%
	Emotional	Self-efficacy	0.24%	0.00%
		Coping ability	0.16%	0.12%
		Motivation	0.12%	0.00%
		Loneliness	0.08%	0.00%
		Self-esteem	0.04%	0.00%
		Emotional functioning	0.00%	0.04%
		Body image	0.04%	0.04%
	Personal	Residential status	0.12%	0.12%
		Employment	0.04%	0.00%
	Health status	Health status	0.08%	0.04%
		Perceived exertion	0.04%	0.00%
	QOL	Prosthesis-related quality of life	0.41%	0.00%
		Quality of life	0.89%	0.28%
Physiological/clinical (23.4%)	Blood	Laboratory parameters	1.18%	0.16%
		Blood transfusion	0.45%	0.08%
		Blood loss	0.28%	0.20%
		Biomarkers	0.04%	0.04%
		Diagnostic test	0.04%	0.04%
		Anemia	0.04%	0.00%
	Cardiovascular	Diagnostic test	0.45%	0.20%
	Endocrine	Diagnostic test	0.08%	0.00%
		Laboratory parameters	0.08%	0.00%
		Physical examination of diabetic neuropathy	0.04%	0.00%
	General	Comorbidities	0.57%	1.14%
		Frailty	0.32%	0.00%
		Diagnostic test	0.12%	0.00%
		Amputation	0.20%	0.12%
		Physical examination	0.08%	0.04%
	Immune	Laboratory parameters	0.49%	0.20%
		Biomarkers	0.20%	0.00%
	Infection	Infection	0.36%	1.30%
		Microbial culture	0.08%	0.00%
	Injury	Falls	0.20%	0.04%
		Pressure ulcers	0.08%	0.08%
		Fracture	0.04%	0.00%
	Metabolic	Nutritional control	0.08%	0.00%
		Body mass	0.08%	0.04%
	Musculoskeletal	Radiology	0.12%	0.00%
		Diagnostic test	0.08%	0.00%
		Contractures	0.00%	0.16%
	Nervous	General pain	0.57%	0.08%
		Low back pain	0.12%	0.00%
		Neuropathic pain	0.08%	0.04%
		Pain catastrophizing	0.04%	0.00%
		Pain relief	0.04%	0.08%
		Pain interference	0.04%	0.00%
		Phantom limb pain	0.41%	0.73%
		Phantom limb sensation	0.08%	0.45%
		Radiology	0.04%	0.00%
		Sensation	0.04%	0.04%
		Residual limb pain	0.32%	1.82%
	Psychiatric	Addiction outcomes	0.28%	0.00%
		Anxiety	0.12%	0.04%
		Behavior changes	0.04%	0.00%
		Delirium	0.12%	0.04%
		Mental health	0.16%	0.08%
		Fear of falling	0.04%	0.00%
		Depression	0.32%	0.16%
	Respiratory	Cardiopulmonary fitness	0.04%	0.00%
	Renal	Laboratory parameters	0.41%	0.04%
	Skin	Stump wound healing	0.28%	5.15%
		Wound	0.20%	0.24%
	Vascular	Chronic limb threatening ischemia staging	0.04%	0.00%
		Diagnostic test	0.57%	0.00%
		Radiology	0.36%	0.00%
Resource use (17.6%)	Societal burden	Carer burden	0.04%	0.00%
		Family support	0.04%	0.00%
		Social support	0.36%	0.08%
	Further intervention	Hemodialysis	0.04%	0.08%
		Emergency procedure	0.08%	0.08%
		Respiratory support	0.08%	0.24%
		Management of fractures	0.04%	0.00%
		Mobility aid	0.20%	0.53%
		Predicting outcome	0.08%	0.00%
		Prescription of medication	1.09%	0.97%
		Resuscitation	0.04%	0.00%
		Rehabilitation	0.24%	1.34%
		Torniquet use	0.08%	0.00%
		Wound management	0.08%	0.00%
		Use of anesthesia	0.73%	0.20%
		Special provisions	0.04%	0.08%
		Specialist consultation	0.41%	0.00%
		Reoperation	0.32%	0.08%
	Economic	Cost	0.28%	0.41%
		Socioeconomic disadvantage	0.04%	0.00%
		Insurance coverage	0.04%	0.00%
	Hospital	Admission	0.65%	0.04%
		Discharge	0.85%	1.42%
		Duration of operation	0.36%	0.08%
		Operative outcome	0.24%	0.00%
		Operative success	0.16%	0.41%
		Duration of stay	1.30%	2.72%
		Readmission	0.69%	0.24%
Adverse event (14.6%)	Adverse event	Adverse events	0.49%	3.12%
		Complications	1.30%	2.07%
		Re-amputation/revision	1.66%	5.92%

### Distribution of outcome measures and indicators across subdomains

Among the 933 unique outcome measures and indicators identified, only a limited number of outcome measures and indicators were reported in ≥ 1% of studies. The most frequently reported included mortality (4.30%), wound healing (3.85%), 30-day mortality (3.12%), successful prosthesis fitting (2.68%), survival (2.55%), length of stay (2.11%), re-amputation rate (1.62%), use of medications (1.58%), discharge destination (1.42%), and one-year mortality (1.22%). A detailed list of outcome measures and indicators, along with their frequencies, is presented in Supplementary Table 3.

### Outcome characteristics

Of the 1,004 outcome measures and indicators identified in this study, 66.9% were classified as clinician-reported, 23.9% as patient-reported, and 9.2% as performance-based outcomes. Outcome measures and indicators from Ambler, et al. [31] were not characterized, as the authors did not provide a list of studies or additional information upon request.

### Heterogeneity of outcome measures and indicators

A detailed list of measures and indicators and their frequencies is presented in Supplementary Table 3. The table highlights the heterogeneity of outcomes by showing the number of unique outcome measures and indicators reported within each subdomain, domain, and core area.

## Discussion

This review provides a comprehensive update of Ambler, et al. [31], extending and harmonizing the classification of outcome measures and indicators reported for adults with major dysvascular LLA. Across both review periods, a combined total of 2,466 outcome measures and indicators were classified across five core areas, 29 domains, and 135 subdomains. The results indicate not only an expansion in the number of reported outcome measures and indicators, but also a substantial evolution in outcome reporting over time. Among the 933 unique outcome measures and indicators identified across both reviews, only 67 (7.2%) were reported in both review periods, meaning that more than 92% of identified outcome measures and indicators differed between reviews. This highlights the considerable heterogeneity and evolution of outcome reporting in dysvascular LLA research, where similar outcomes are often assessed using multiple, non-standardized measures and indicators. While this expansion may signal greater research activity, it also underscores the persistent lack of consensus in outcome selection and reporting, which continues to limit comparability across studies and the ability to synthesize evidence effectively.

While the present review builds directly on the foundational work of Ambler et al. [31], it extends that work in several meaningful ways. First, it extends the evidence base by capturing studies published between April 2017 and December 2022, adding 160 studies reporting data on over 228,000 individuals and bringing the combined total to 600 studies. Second, all outcome measures and indicators identified in the present updated review were explicitly linked to their source study, clinical context (i.e., acute care, rehabilitation, or community setting), and outcome type (i.e., patient-reported, clinician-reported, or performance-based), providing a higher level of granularity and transparency than reported in the original review [31]. Third, to enable consistent comparison across review periods, the outcome measures and indicators from Ambler et al. [31] were reclassified using a harmonized application of Dodd's framework allowing outcomes reported across both reviews to be examined within a common classification structure. Importantly, Dodd's taxonomy provides core areas and outcome domains but encourages further refinement according to the needs of specific clinical fields. Therefore, additional clinically relevant subdomains were developed where greater specificity was required for dysvascular LLA, enabling a more granular classification of outcome measures and indicators.

Beyond updating and harmonizing the evidence base, this review provides new insights into current outcome reporting practices in dysvascular LLA research. Specifically, the findings highlight substantial heterogeneity in reported outcomes, the predominance of clinician-reported outcomes, particularly mortality and clinical indicators, and the comparatively limited representation of patient-reported and rehabilitation-focused outcomes. These findings identify important gaps in current outcome reporting and provide priorities for future standardization efforts and COS development in this population.

Collectively, these contributions position the present review as an expanded and methodologically consistent update of the work by Ambler et al., while providing a more detailed and clinically relevant framework to support future COS development and outcome standardization in individuals with major dysvascular LLA.

### Core areas

To ensure consistency with Dodd’s taxonomy, the outcome measures and indicators from the Ambler, et al. [31] review were reclassified in this study. This step was necessary because the original review did not categorize the measures and indicators according to the core area hierarchy proposed by Dodd et al. [32], limiting the assessment of outcome distribution across the recommended core areas. Following reclassification, outcome measures and indicators were most frequently reported in the core areas of life impact and physiological/clinical, followed by death, resource use, and adverse events. This distribution suggests that outcome reporting in this population has historically prioritized clinically measurable outcomes over those capturing broader patient-centered and system-level impacts. These patterns mirror those observed in a previous review on traumatic LLAs [206] and remain equally concerning. In particular, the underreporting of outcomes related to resource use and adverse events limits our ability to fully understand the broader impact of interventions in adults with major dysvascular LLAs. This underreporting may reflect a combination of study design priorities that tend to emphasize functional and clinical endpoints, as suggested by the distribution of outcome measures and indicators identified in this review [207], as well as the practical challenges associated with capturing economic and safety-related data across fragmented, multidisciplinary care pathways [36].

Resource use outcomes, including length of hospital stay, readmissions, and rehabilitation costs, directly affect both individuals with LLAs and healthcare systems. A recent study from the United States highlighted this issue, reporting an average two-year hospitalization cost of $114,292 per person for individuals with dysvascular LLAs [208]. Beyond hospitalization, many patients require prosthetic devices and rehabilitation services to regain mobility and achieve community reintegration [209, 210]. These services significantly contribute to total healthcare expenditures [211, 212], underscoring the importance of consistently reporting resource use indicators to inform healthcare planning, policy development, and long-term care trajectories for this population. Similarly, improved reporting is needed in the core area of adverse events. Adverse events such as infections and prosthesis-related complications are often underreported [213], distorting understanding of the true burden of care, hindering risk–benefit analyses, and compromising efforts to design responsive and cost-effective models of care, collectively limiting the ability to allocate resources appropriately and to optimize outcomes for this population.

Beyond the underreporting of these domains, the choice of classification framework itself warrants consideration. The choice of Dodd's taxonomy was not incidental; as this review is an update, methodological consistency required its continued use to enable meaningful comparison across review iterations. Beyond continuity, Dodd's taxonomy is broader in scope than the ICF, encompassing outcome domains particularly relevant to intervention research that the ICF does not systematically address. Both frameworks recognize physical, social, emotional, and cognitive dimensions of health outcomes, and the ICF's Activities and Participation component maps reasonably well onto Dodd's functioning domains. However, they diverge significantly in purpose: the ICF is a biopsychosocial conceptual framework [214], whereas Dodd's taxonomy is an operational classification tool designed specifically for organizing outcomes in clinical trials and systematic reviews [32]. Mortality, adverse events, and resource use are either outside the ICF's scope or only partially and indirectly addressable within its structure, domains that would likely have been inconsistently classified or underrepresented had the ICF been employed, which would have been particularly consequential given that underreporting of these outcomes was itself a key finding of this review.

### Outcome domains

Comparing outcome domains across the two reviews reveals both consistencies and meaningful shifts, potentially reflecting evolving research priorities over the intervening five years. Physical functioning, mortality, hospital-based outcomes, and need for further interventions were consistently prominent across both reviews. Notable differences included a greater prominence of adverse events and blood and lymphatic system outcomes in the current review, and of skin and subcutaneous tissue outcomes in Ambler et al., [31].

These discrepancies can be attributed to two main factors. First, the review by Ambler, et al. [31] included a larger number of studies (n = 440) compared to the present review (n = 160). This broader sample likely contributed to a more diverse representation of outcome domains and may have inflated the frequency of some outcomes. Second, differences in outcome classification may have contributed to variation in reporting, even though both studies used Dodd’s taxonomy. Specifically, we identified inconsistencies in how certain outcome measures and indicators were categorized in Ambler et al.’s review. As a result, we re-examined and reclassified all outcome measures and indicators from their study (see Supplementary Table 3 for a detailed classification). Following reclassification and integration of outcome measures and indicators from both reviews, physical functioning was the most frequently reported domain overall, followed by mortality, adverse events, hospital use, and need for further interventions. Notably, the increase in outcome measures and indicators across the two reviews does not appear to reflect progress toward standardization. Rather, it reflects a growing but increasingly fragmented literature, where the proliferation of non-comparable measures further complicates evidence synthesis and limits the ability to draw meaningful conclusions at the population level.

Furthermore, within the life impact core area, a notable finding emerged: physical functioning was frequently reported (19.5%), while other critical domains, namely global quality of life, social function, and emotional well-being, were significantly underreported (i.e., < 2%). This is concerning given the well-documented impact of major dysvascular LLAs on these aspects of life [215–217].

Similar discrepancies were observed in other core areas, including physiological/clinical, resource use, and mortality. Within the physiological/clinical core area, outcomes related to skin and subcutaneous tissue, as well as the nervous system, comprised 10.9% of total reported outcome measures and indicators. In contrast, domains such as psychiatric, vascular, musculoskeletal, and injury-related outcomes were seldom reported. The underreporting of psychiatric outcomes, specifically depression and anxiety, is particularly concerning given their high prevalence among individuals with LLAs [218] and their well-established impact on holistic outcomes [192].

Within the resource use core area, hospital utilization and need for further interventions together comprised a combined 16.3% of all outcome measures and indicators. In contrast, domains reflecting economic burden and societal burden were rarely (< 1%) reported. This is concerning, given the substantial financial and societal toll associated with major dysvascular LLAs [208, 219], and this gap further hinders efforts to inform effective policy and resource allocation.

### Outcome subdomains

Moving to a more granular level, within the physical functioning domain, subdomains such as mobility, spatiotemporal gait parameters, prosthesis fit, and prosthesis use were reported more frequently than balance, endurance, and energy efficiency. This discrepancy has important implications for both research and clinical practice. For example, balance is critical for falls prevention, a major concern for individuals with LLAs [205]. Likewise, endurance and energy efficiency are often significantly affected in individuals with dysvascular LLA. Ettema, et al. [220] highlighted that the energy cost of walking is significantly higher in this population due to their reliance on less efficient propulsion and leg swing strategies when using a prosthesis. In addition, reduced physical fitness, largely driven by comorbidities, may further contribute to increased energy expenditure and reduced endurance and efficiency [221].

Comparable gaps were observed within the need for further interventions domain. Notably, subdomains such as specialist consultation and need for mobility aids were markedly underreported (i.e., < 1%), despite their significant influence on prognosis following major dysvascular LLA. Early specialist consultation is particularly critical, as timely involvement of vascular interventionalists can facilitate limb salvage through procedures such as angioplasty or revascularization, potentially preventing amputation [222, 223]. Likewise, the need for mobility aids remains underrepresented despite being central to post-amputation rehabilitation. Individuals with major dysvascular LLAs often rely on devices such as prostheses, wheelchairs, or walkers to regain mobility and functional independence, and limited reporting of this subdomain restricts understanding of rehabilitation needs and hinders efforts to optimize care planning and quality of life [153, 224].

### Outcome measures and indicators

Beyond subdomains, considerable heterogeneity was observed in the reporting of individual outcome measures and indicators. For instance, 24 unique outcome measures and indicators were used to evaluate spatiotemporal gait parameters alone. Similarly, the mobility and quality of life subdomains showed notable variability, with 30 and 12 unique outcome measures and indicators, respectively. Although some variability is expected, particularly in broad domains like mobility where different tools may capture specific aspects with varying sensitivity [225], this degree of heterogeneity reflects a deeper issue: the absence of standardized outcome selection. When similar measures and indicators are measured with multiple, non-comparable tools, data aggregation becomes highly challenging and the ability to draw robust, generalizable conclusions across studies is substantially diminished.

This fragmentation carries real consequences. It obscures population-level effect estimates, complicates cross-study comparison, and ultimately impedes the development of consistent, evidence-based clinical guidelines, limiting the translation of research findings into practice and policy [226]. Compounding this issue, the psychometric properties of outcome measures, including their validity, reliability, and responsiveness, are rarely systematically considered despite being fundamental criteria for both clinical research and COS development [227, 228]. Future COS development for dysvascular LLA should therefore consider formal psychometric properties to ensure that selected measures are both methodologically robust and clinically relevant [228].

### Types of outcome assessment

Beyond what is measured, how outcomes are measured is equally important for evidence quality. Types of outcome measurement were identified only for the 160 studies included in the present review, as this information was not available in the original review by Ambler, et al. [31]. Nevertheless, a clear imbalance was observed, with clinician-reported outcomes dominating (66.9%), while patient-reported (23.9%) and performance-based outcomes (9.2%) were less frequently used.

Although clinician-reported outcomes are valuable, they should complement, rather than replace, patient-reported outcomes [229]. For instance, Nielsen, et al. [230] demonstrated that patient-reported and performance-based measures of functional ability are correlated but capture distinct aspects of function, highlighting the importance of combining these outcome types. This is particularly relevant in research involving older adults, who may have limited awareness of their true functional capacity or whose self-reports may be influenced by cognitive decline [230, 231], a consideration that is especially pertinent given the mean participant ages of 66 and 73.5 years in the current and original reviews, respectively.

Collectively, these findings contribute to current knowledge by providing a methodologically consistent classification of outcome measures and indicators for this population and by making the gaps in the existing evidence base explicit in a manner that can directly inform the scope and priorities of future COS development.

### Limitations

This study has several limitations that should be considered when interpreting the findings. First, the methodological quality of the included studies in this review was not assessed, as the primary objective was to map and categorize outcome measures and indicators rather than to evaluate the effectiveness of specific interventions. Consequently, some included studies may be of lower methodological quality, and findings derived from these studies should be interpreted with appropriate caution. This approach is appropriate for the aims of the review, which focused on providing a comprehensive inventory of outcome measures and indicators in the field, regardless of study quality. Restricting inclusion to high-quality studies could have resulted in the omission of relevant outcome measures and indicators that, while reported in methodologically weaker studies, may nonetheless be clinically meaningful and relevant to future COS development. This methodological approach is consistent with that of the original review by Ambler et al., which also did not include a formal quality appraisal.

Second, independent data extraction was conducted for only 40 of the 160 studies, while the remaining data were extracted and classified by a single reviewer (SG). Although this may be viewed as a limitation, double data extraction for all studies was not feasible due to limited resources. Several procedural safeguards were nonetheless in place to promote consistency and minimize classification bias. The initial 40 studies demonstrated a high level of agreement between reviewers (85%), providing a robust foundation for subsequent classification. Furthermore, the use of Dodd's taxonomy as a predefined and structured framework reduced the degree of subjective judgement required at each level of classification. Likewise, the transparent reporting of all outcome measures and indicators in Supplementary Tables 3 and 4 further enhances the reproducibility and utility of this review for future updates and enables independent verification of the classifications [232].

Third, the review included only articles published in English, German, or French, and the reference lists of included studies were not screened, which may have led to the unintentional exclusion of relevant literature.

Fourth, although outcome measures and indicators from Ambler, et al. [31]’s supplementary file were reclassified using Dodd’s framework, this process may not fully reflect the context-specific intent of the original studies, as the references for individual outcome measures and indicators were not provided. Attempts to obtain this information from the corresponding author were unsuccessful. This introduces the possibility that some outcome measures and indicators may have been misclassified relative to the original authors' intent, which could influence comparisons between the two reviews. To minimize this risk, reclassification was conducted systematically using the predefined hierarchical structure of Dodd's taxonomy, applying the same classification criteria used for the present review. Ambiguous reclassification decisions were discussed with and verified by a senior experienced researcher (DZ), further reducing the risk of misclassification. Furthermore, all reclassified outcome measures and indicators are reported transparently in Supplementary Table 3, enabling readers to independently audit the classification decisions and assess their validity. Comparisons between the two reviews should therefore be interpreted with appropriate caution, recognizing that observed differences may partially reflect reclassification decisions rather than true changes in outcome reporting practices.

Fifth, while systematic reviews should ideally be updated regularly to capture emerging evidence, the final search for this review was conducted in December 2022. Given the substantial scope of this work, including independent screening of 12,557 records, full-text assessment of 729 reports, data extraction from 160 studies, and reclassification of outcome measures and indicators according to Dodd's framework, conducting an updated search prior to publication was not feasible. Consequently, relevant studies published after December 2022 may not be represented in this review. An updated search on MEDLINE Ovid identified 1,243 additional records. Given the extensive resources required for independent screening, full-text assessment, data extraction, and outcome reclassification, a complete update was beyond the scope of the current revision. Accordingly, this review should be understood as a systematic and methodologically consistent mapping of outcome measures and indicators reported in the literature up to December 2022, rather than a fully updated synthesis. Nevertheless, this review provides a systematic and transparent synthesis of outcome measurement in dysvascular LLA research and could serve as a foundation for future updates as the field evolves.

Sixth, socioeconomic data were not systematically extracted from the included studies. Socioeconomic factors may influence amputation risk, rehabilitation access, and functional outcomes in individuals with dysvascular LLA, and their absence may limit the contextual application of findings across populations with differing levels of socioeconomic status [132]. Future studies and COS development efforts should consider the systematic collection and reporting of socioeconomic variables to better account for health inequities and support more equitable, person-centered care pathways.

Finally, contextual information, such as the setting or type of outcome assessment, was only available for the 160 studies included in the present review. Despite these limitations, this study underscores inconsistencies in outcome reporting and highlights the need for standardized, comprehensive, and context-sensitive outcome measurements to improve the consistency and quality of research for major dysvascular LLAs.

### Clinical and policy implications

The persistent underreporting of patient-reported, resource use, and adverse event outcome measures and indicators identified in this review has important implications for both clinical practice and health policy. For clinicians, the predominance of clinician-reported physiological measures may result in an incomplete understanding of recovery, potentially overlooking quality of life, emotional well-being, and social participation outcomes which are highly relevant to individuals living with major dysvascular LLA. For policymakers and healthcare planners, the lack of consistent reporting of economic and resource use outcomes limits the ability to evaluate care models, compare outcomes across systems, and inform decisions regarding investment in rehabilitation and community-based services. These findings highlight the need for a condition-specific COS, developed with meaningful input from individuals with lived experience as well as clinical and policy stakeholders, to potentially support more comprehensive and standardized outcome reporting across the continuum of care.

### Future directions

The next steps in this research are to follow the recommendations outlined in the COMET (Core Outcome Measures in Effectiveness Trials) handbook to develop a COS for individuals with major dysvascular LLAs receiving care in rehabilitation and community settings [207]. This updated systematic review constitutes the first phase of a planned three-phase process: i) completion of two systematic reviews, one on traumatic LLAs [206], and the present review on dysvascular LLAs; ii) stakeholder consultations involving clinical experts from rehabilitation and community settings as well as individuals with lived experience of major dysvascular LLA; and iii) implementation of a Delphi survey to reach consensus on the COS among key stakeholder groups, including individuals with major LLA, healthcare professionals, and decision-makers. Future work could also explore whether hybrid classification approaches combining ICF's recognized functioning terminology with Dodd's broader coverage of mortality, adverse events, and resource use might further strengthen outcome classification in this field, particularly given ICF's existing clinical uptake in rehabilitation settings.

## Conclusion

This study updates the findings of a previous review by reclassifying 2,466 outcome measures and indicators from 600 studies involving adults with dysvascular LLA. The results show that physical functioning was the most frequently reported outcome domain, followed by mortality and adverse events. A comparison with the earlier review by Ambler, et al. [31] reveals that, despite the passage of five years, outcome reporting has remained heterogeneous, with limited evidence of progress toward standardization, as numerous non-comparable measures and indicators continue to be used to assess the same outcomes, highlighting the pressing need for a standardized COS.

This review also points to notable discrepancies in the types of outcome measures and indicators reported across the 160 included studies, with clinician-reported outcomes dominating, followed by patient-reported and performance-based outcomes. It represents a step toward establishing a COS tailored to rehabilitation and community settings, one that could support patient-centered and evidence-based comparative research and may ultimately contribute to identifying best-practice clinical pathways and interventions that could improve outcomes for individuals living with major dysvascular LLA.

## Supplementary Information

Supplementary Material 1.

## Data Availability

All data generated or analyzed during this study are included in this article and its Supplementary Tables 3 and 4.

## Abbreviations

LLA: Lower limb amputation
COS: Core outcome set
ICF: International Classification of Functioning, Disability and Health
PRISMA: Preferred Reporting Items for Systematic Reviews and Meta-Analyses
PROSPERO: International Prospective Register of Systematic Reviews
COMET: Core Outcome Measures in Effectiveness Trials
QOL: Quality of life
