# Supplementary material for: Outcomes following major dysvascular lower limb amputation: an updated systematic review
Source: BMC Cardiovasc Disord. 2026 Aug 6;26:674. doi: 10.1186/s12872-026-06377-5 (PMC13450402; doi:10.1186/s12872-026-06377-5)
Supplement: Supplementary file 1 [file 12872_2026_6377_MOESM1_ESM.docx]

**Supplementary file**

**Supplementary table 1.** PRISMA checklist

| **Section and Topic** | **Item #** | **Checklist item** | **Location where item is reported** |
| --- | --- | --- | --- |
| **TITLE** | | |  |
| Title | 1 | Identify the report as a systematic review. | 1 |
| **ABSTRACT** | | |  |
| Abstract | 2 | See the PRISMA 2020 for Abstracts checklist. | 1 |
| **INTRODUCTION** | | |  |
| Rationale | 3 | Describe the rationale for the review in the context of existing knowledge. | 2-3 |
| Objectives | 4 | Provide an explicit statement of the objective(s) or question(s) the review addresses. | 3 |
| **METHODS** | | |  |
| Eligibility criteria | 5 | Specify the inclusion and exclusion criteria for the review and how studies were grouped for the syntheses. | 4 |
| Information sources | 6 | Specify all databases, registers, websites, organisations, reference lists and other sources searched or consulted to identify studies. Specify the date when each source was last searched or consulted. | 4 |
| Search strategy | 7 | Present the full search strategies for all databases, registers and websites, including any filters and limits used. | Supplementary table 2 |
| Selection process | 8 | Specify the methods used to decide whether a study met the inclusion criteria of the review, including how many reviewers screened each record and each report retrieved, whether they worked independently, and if applicable, details of automation tools used in the process. | 4 |
| Data collection process | 9 | Specify the methods used to collect data from reports, including how many reviewers collected data from each report, whether they worked independently, any processes for obtaining or confirming data from study investigators, and if applicable, details of automation tools used in the process. | 4-5 |
| Data items | 10a | List and define all outcomes for which data were sought. Specify whether all results that were compatible with each outcome domain in each study were sought (e.g. for all measures, time points, analyses), and if not, the methods used to decide which results to collect. | 5 |
|  | 10b | List and define all other variables for which data were sought (e.g. participant and intervention characteristics, funding sources). Describe any assumptions made about any missing or unclear information. | 5 |
| Study risk of bias assessment | 11 | Specify the methods used to assess risk of bias in the included studies, including details of the tool(s) used, how many reviewers assessed each study and whether they worked independently, and if applicable, details of automation tools used in the process. | NA |
| Effect measures | 12 | Specify for each outcome the effect measure(s) (e.g. risk ratio, mean difference) used in the synthesis or presentation of results. | NA |
| Synthesis methods | 13a | Describe the processes used to decide which studies were eligible for each synthesis (e.g. tabulating the study intervention characteristics and comparing against the planned groups for each synthesis (item #5)). | 5 |
|  | 13b | Describe any methods required to prepare the data for presentation or synthesis, such as handling of missing summary statistics, or data conversions. | 5-6 |
|  | 13c | Describe any methods used to tabulate or visually display results of individual studies and syntheses. | 5-6 |
|  | 13d | Describe any methods used to synthesize results and provide a rationale for the choice(s). If meta-analysis was performed, describe the model(s), method(s) to identify the presence and extent of statistical heterogeneity, and software package(s) used. | 5-6 |
|  | 13e | Describe any methods used to explore possible causes of heterogeneity among study results (e.g. subgroup analysis, meta-regression). | NA |
|  | 13f | Describe any sensitivity analyses conducted to assess robustness of the synthesized results. | NA |
| Reporting bias assessment | 14 | Describe any methods used to assess risk of bias due to missing results in a synthesis (arising from reporting biases). | NA |
| Certainty assessment | 15 | Describe any methods used to assess certainty (or confidence) in the body of evidence for an outcome. | NA |
| **RESULTS** | | |  |
| Study selection | 16a | Describe the results of the search and selection process, from the number of records identified in the search to the number of studies included in the review, ideally using a flow diagram. | 6-8 |
|  | 16b | Cite studies that might appear to meet the inclusion criteria, but which were excluded, and explain why they were excluded. | 6 |
| Study characteristics | 17 | Cite each included study and present its characteristics. | 6-8, Supplementary table 3 |
| Risk of bias in studies | 18 | Present assessments of risk of bias for each included study. | NA |
| Results of individual studies | 19 | For all outcomes, present, for each study: (a) summary statistics for each group (where appropriate) and (b) an effect estimate and its precision (e.g. confidence/credible interval), ideally using structured tables or plots. | NA |
| Results of syntheses | 20a | For each synthesis, briefly summarise the characteristics and risk of bias among contributing studies. | Supplementary table 3 |
|  | 20b | Present results of all statistical syntheses conducted. If meta-analysis was done, present for each the summary estimate and its precision (e.g. confidence/credible interval) and measures of statistical heterogeneity. If comparing groups, describe the direction of the effect. | 6-15 |
|  | 20c | Present results of all investigations of possible causes of heterogeneity among study results. | 15, Supplementary table 4 |
|  | 20d | Present results of all sensitivity analyses conducted to assess the robustness of the synthesized results. | NA |
| Reporting biases | 21 | Present assessments of risk of bias due to missing results (arising from reporting biases) for each synthesis assessed. | NA |
| Certainty of evidence | 22 | Present assessments of certainty (or confidence) in the body of evidence for each outcome assessed. | NA |
| **DISCUSSION** | | |  |
| Discussion | 23a | Provide a general interpretation of the results in the context of other evidence. | 15-18 |
|  | 23b | Discuss any limitations of the evidence included in the review. | 15-20 |
|  | 23c | Discuss any limitations of the review processes used. | 19-20 |
|  | 23d | Discuss implications of the results for practice, policy, and future research. | 20-21 |
| **OTHER INFORMATION** | | |  |
| Registration and protocol | 24a | Provide registration information for the review, including register name and registration number, or state that the review was not registered. | 3 |
|  | 24b | Indicate where the review protocol can be accessed, or state that a protocol was not prepared. | 3 |
|  | 24c | Describe and explain any amendments to information provided at registration or in the protocol. | NA |
| Support | 25 | Describe sources of financial or non-financial support for the review, and the role of the funders or sponsors in the review. | 21 |
| Competing interests | 26 | Declare any competing interests of review authors. | NA |
| Availability of data, code and other materials | 27 | Report which of the following are publicly available and where they can be found: template data collection forms; data extracted from included studies; data used for all analyses; analytic code; any other materials used in the review. | NA |

*From:* Page MJ, McKenzie JE, Bossuyt PM, Boutron I, Hoffmann TC, Mulrow CD, et al. The PRISMA 2020 statement: an updated guideline for reporting systematic reviews. BMJ 2021;372:n71. doi: 10.1136/bmj.n71

For more information, visit: <http://www.prisma-statement.org/>

**Supplementary table 2.** Search keywords used for review for Ovid MEDLINE(R) ALL

| **Steps** | **Search query** |
| --- | --- |
| 1. | exp Amputees/ |
| 2. | exp Amputation Stumps/ or exp Amputation/ or exp Amputation, Traumatic/ |
| 3. | exp Artificial Limbs/ |
| 4. | "prostheses and implants"/ or artificial limbs/ or bone-anchored prosthesis/ or implants, experimental/ or joint prosthesis/ |
| 5. | (amputee* or amputation* or stump* or limb loss* or artificial limb* or prosthesis or prostheses or disarticulation*).ab,ti. |
| 6. | 1 or 2 or 4 or 5 |
| 7. | exp Lower Extremity/ |
| 8. | (LLA or LEA or lower limb* or lower extremit* or leg or legs or ankle* or buttock* or foot or feet or heel* or hip or hips or knee* or thigh* or transtibial or transfemoral or choppart or syme or gluteal region or hemipelvectom* or toe or toes or forefoot or forefeet or rear foot or rear feet or rearfoot or rearfeet or transmetatarsal or TMA or pedal or hallux or digit or digits).ab,ti. |
| 9. | or/7-8 |
| 10. | exp Peripheral Vascular Diseases/ |
| 11. | exp Arterial Occlusive Diseases/ |
| 12. | (atherosclero* or arteriosclero* or PVD or PAOD or PAD).ab,ti. |
| 13. | ((occlus* or steno* or obstruct* or lesio* or block* or obliter*) adj2 (vein or veno* or arterial or vascular or peripheral)).ab,ti. |
| 14. | (peripheral adj3 dis*).ab,ti. |
| 15. | arteriopath*.ab,ti. |
| 16. | (CLI or critical limb ischemia).ab,ti. |
| 17. | dysvascular*.ab,ti. |
| 18. | or/10-17 |
| 19. | 6 and 9 and 18 |
| 20. | exp animals/ not humans.sh. |
| 21. | 19 not 20 |
| 22. | limit 21 to dt=20170401-20221201 |
| Link to search: <https://ovidsp.ovid.com/ovidweb.cgi?T=JS&NEWS=N&PAGE=main&SHAREDSEARCHID=3OVMqrFNrDnvPNRaiLzMqkEokqzu2pM29FlAUiR8Us0UCKVERgLrAuYeBtsgOf26u> | |

**Supplementary table 3.** Complete classification of outcome measures and indicators from the present review and from Ambler et al. (outcomes from Ambler et al. shown in red), organized according to Dodd's hierarchical framework across core areas, outcome domains, outcome subdomains, and outcome measures and indicators.

| **Death** | | | |  |  |
| --- | --- | --- | --- | --- | --- |
| Outcome domains | Outcome Subdomains | Outcome measures/indicators | Number of studies reporting measures/indicators | % of overall measures/indicators | Unique outcome measures/indicators |
| Mortality/survival | Cause of mortality | Cause of mortality | 1+**19** | 0.81% | 4 |
|  |  | Possible cause of mortality | 2 | 0.08% |  |
|  |  | *Cause of in-hospital death* | **1** | 0.04% |  |
|  |  | *Mortality from purulent and necrotic complications* | **1** | 0.04% |  |
|  | Survival | Duration of post-operative survival (days) | 1 | 0.04% | 21 |
|  |  | Duration of survival (months) | 1 | 0.04% |  |
|  |  | Median survival time | 1 | 0.04% |  |
|  |  | Overall survival probability | 1 | 0.04% |  |
|  |  | Survival duration (years) | 1 | 0.04% |  |
|  |  | Life years per patient | 1 | 0.04% |  |
|  |  | Survival status (survived/deceased) | 1 | 0.04% |  |
|  |  | Survival (1-month) | 1 | 0.04% |  |
|  |  | Survival rate (6-month) | 1 | 0.04% |  |
|  |  | Survival (6-month) | 1 | 0.04% |  |
|  |  | Survival rate (1-year) | 2 | 0.08% |  |
|  |  | Survival (1-year) | 4+**4** | 0.32% |  |
|  |  | Survival (2-year) | 1+**1** | 0.08% |  |
|  |  | Survival (3-year) | 1+**1** | 0.08% |  |
|  |  | Survival rate (5-year) | 1 | 0.04% |  |
|  |  | Survival (5-year) | 1**+1** | 0.08% |  |
|  |  | *Survival (2-month)* | **1** | 0.04% |  |
|  |  | *Survival* | **63** | 2.55% |  |
|  |  | *Long term patient survival* | **1** | 0.04% |  |
|  |  | *Additional amputation free survival* | **1** | 0.04% |  |
|  |  | *Contralateral amputation free survival* | **2** | 0.08% |  |
|  | Mortality | All-cause mortality | 1+**1** | 0.08% | 52 |
|  |  | All-cause mortality (1-year) | 1 | 0.04% |  |
|  |  | All-cause mortality (30-day) | 1 | 0.04% |  |
|  |  | All-cause mortality (5-year) | 1 | 0.04% |  |
|  |  | All-cause mortality (90-day) | 1 | 0.04% |  |
|  |  | Cumulative mortality (2-year) | 1 | 0.04% |  |
|  |  | Death (3 to 5 years) | 1 | 0.04% |  |
|  |  | Early <30-day mortality | 1 | 0.04% |  |
|  |  | In-hospital mortality | 4+**14** | 0.73% |  |
|  |  | Long term mortality | 1 | 0.04% |  |
|  |  | Mean age at time of death | 1 | 0.04% |  |
|  |  | Mortality | 5+**101** | 4.30% |  |
|  |  | Mortality (>30-day) | 1 | 0.04% |  |
|  |  | Mortality (≤30-day) | 1 | 0.04% |  |
|  |  | Mortality (1-week) | 1+**1** | 0.08% |  |
|  |  | Mortality (1-year) | 19+**11** | 1.22% |  |
|  |  | Mortality (2-year) | 5+**6** | 0.45% |  |
|  |  | Mortality (30-day) | 31+**46** | 3.12% |  |
|  |  | Mortality (3-year) | 1 | 0.04% |  |
|  |  | Mortality (48-hour) | 1 | 0.04% |  |
|  |  | Mortality (60-day) | 1 | 0.04% |  |
|  |  | Mortality (6-month) | 6+**6** | 0.49% |  |
|  |  | Mortality (in-patient) | 1 | 0.04% |  |
|  |  | Mortality (mean years) after index amputation | 1 | 0.04% |  |
|  |  | Mortality before (1 year) | 2 | 0.08% |  |
|  |  | Mortality before (28 days) | 1 | 0.04% |  |
|  |  | Mortality following ICU stay (1 year) | 1 | 0.04% |  |
|  |  | Mortality following ICU stay (28 days) | 1 | 0.04% |  |
|  |  | Mortality prior to contralateral major/minor lower limb amputation | 1 | 0.04% |  |
|  |  | Mortality rate (1-year) | 2 | 0.08% |  |
|  |  | Mortality rate (5-year) | 2 | 0.08% |  |
|  |  | Overall mortality | 1 | 0.04% |  |
|  |  | Overall mortality (23-month) | 1 | 0.04% |  |
|  |  | Overall mortality (30-day) | 1 | 0.04% |  |
|  |  | Postoperative days to death | 1 | 0.04% |  |
|  |  | Time to death (months) | 1 | 0.04% |  |
|  |  | Mortality (operative) | 1 | 0.04% |  |
|  |  | *Mortality (8-year)* | **1** | 0.04% |  |
|  |  | *Death after discharge* | **1** | 0.04% |  |
|  |  | *Death before discharge* | **1** | 0.04% |  |
|  |  | *Died before healing* | **1** | 0.04% |  |
|  |  | *Died within (2-year) of amputation* | **1** | 0.04% |  |
|  |  | *Early mortality rate within (14-day)* | **3** | 0.12% |  |
|  |  | *In-patient death in rehabilitation* | **2** | 0.08% |  |
|  |  | *Mortality (3-month)* | **4** | 0.16% |  |
|  |  | *Mortality (3-year)* | **2** | 0.08% |  |
|  |  | *Mortality (5-year)* | **5** | 0.20% |  |
|  |  | *Perioperative mortality* | **19** | 0.77% |  |
|  |  | *Postoperative death* | **2** | 0.08% |  |
|  |  | *Postoperative early mortality* | **1** | 0.04% |  |
|  |  | *Postoperative mortality* | **3** | 0.12% |  |
|  |  | *Total mortality* | **2** | 0.08% |  |
| **Life impact** \| \| \| \| \|; Physical functioning \| Gait: Spatiotemporal \| 10-meter walk test \| 4+**1** \| 0.24% \| 24; \| \| 2MWT \| 11+**3** \| 0.57% \|; \| \| 5-meter walk test \| 1+**1** \| 0.08% \|; \| \| 6MWT \| 6 \| 0.24% \|; \| \| Gait speed \| 4+**2** \| 0.24% \|; \| \| Gait speed (self-selected) \| 2 \| 0.08% \|; \| \| Gait speed (fast gait) \| 1 \| 0.04% \|; \| \| Stance time asymmetry index (self-selected) \| 1 \| 0.04% \|; \| \| Stance time asymmetry index (fast gait) \| 1 \| 0.04% \|; \| \| Static weight bearing and ability to walk (without prosthesis/ with light prosthesis/ with Osseo integrated implant) \| 1 \| 0.04% \|; \| \| Step count \| 6 \| 0.24% \|; \| \| Step length \| 1+**1** \| 0.08% \|; \| \| Step length asymmetry index (self-selected) \| 1 \| 0.04% \|; \| \| Step length asymmetry index (fast gait) \| 1 \| 0.04% \|; \| \| Limp index \| 1 \| 0.04% \|; \| \| Time spent walking \| 1 \| 0.04% \|; \| \| Walking time prior to lower limb amputation (minutes) \| 1 \| 0.04% \|; \| \| Stance characteristics \| 1 \| 0.04% \|; \| \| Step ascent task completion time \| 1 \| 0.04% \|; \| \| Step descent task completion time \| 1 \| 0.04% \|; \| \| *Cadence* \| **1** \| 0.04% \|; \| \| *Step time* \| **1** \| 0.04% \|; \| \| *Velocity* \| **1** \| 0.04% \|; \| \| *Distance walked* \| **2** \| 0.08% \|; \| Gait: Kinetics \| Index scores of peak sagittal plane moment \| 1 \| 0.04% \| 4; \| \| Peak vertical ground reaction force \| 1 \| 0.04% \|; \| \| Vertical impulse \| 1 \| 0.04% \|; \| \| Peak internal moments with step ascent and descent during single limb support \| 1 \| 0.04% \|; \| Gait: Kinematics \| Trunk rotational angular momentum during step ascent \| 1 \| 0.04% \| 2; \| \| Trunk rotational angular momentum during step descent \| 1 \| 0.04% \|; \| Balance \| Berg balance scale \| 4 \| 0.16% \| 7; \| \| Four square step test \| 1 \| 0.04% \|; \| \| One leg stand test \| 1 \| 0.04% \|; \| \| Centre of gravity excursions \| 1 \| 0.04% \|; \| \| *Inclination angles obtained through second order regression analysis using stabilogram data* \| **1** \| 0.04% \|; \| \| *Distribution of reaction forces between the two legs: balance* \| **1** \| 0.04% \|; \| \| *Centre of pressure trajectories and time function* \| **1** \| 0.04% \|; \| Balance confidence \| Activities and balance confidence scale \| 3 \| 0.12% \| 3; \| \| Falls efficacy scale-international \| 2 \| 0.08% \|; \| \| Modified falls efficacy scale \| 1 \| 0.04% \|; \| Endurance \| Standing tolerance (minutes) \| 1 \| 0.04% \| 1; \| Energy efficiency \| Physical activity scale for individuals with physical disabilities questionnaire \| 1 \| 0.04% \| 1; \| Functional independence \| Barthel index \| 4 \| 0.16% \| 18; \| \| Functional impact of symptoms \| 1 \| 0.04% \|; \| \| Functional independence \| 1+**4** \| 0.20% \|; \| \| Functional independence measure \| 3+**5** \| 0.32% \|; \| \| Functional status (bedridden) \| 1 \| 0.04% \|; \| \| Modified Barthel index \| 2 \| 0.08% \|; \| \| Physical function (independent, partially dependent, totally dependent) \| 1 \| 0.04% \|; \| \| Loss of independence \| 1 \| 0.04% \|; \| \| Pre-amputation functional status \| 1 \| 0.04% \|; \| \| *Barthel index effectiveness at discharge* \| **3** \| 0.12% \|; \| \| *Discharge functional status* \| **4** \| 0.16% \|; \| \| *Return to pre-morbid functional status* \| **1** \| 0.04% \|; \| \| *Functional activity level* \| **1** \| 0.04% \|; \| \| *Functional measure for amputees* \| **1** \| 0.04% \|; \| \| *Functional outcome* \| **2** \| 0.08% \|; \| \| *Independence of activities of daily living* \| **1** \| 0.04% \|; \| \| *Maintenance of independent living* \| **6** \| 0.24% \|; \| \| *Independence* \| **2** \| 0.08% \|; \| Impact of disease/conditional on activities of daily living \| World Health Organization disability assessment schedule \| 5 \| 0.20% \| 10; \| \| Activity limitations (bed mobility, sitting, standing balance transfer, standing tolerance, gait) \| 1 \| 0.04% \|; \| \| Effects of phantom phenomena on daily activities \| 1 \| 0.04% \|; \| \| Impact of pain and comorbid conditions (semi-structured interview) \| 1 \| 0.04% \|; \| \| Late life function and disability index-disability scale (frequency, limitation) \| 1 \| 0.04% \|; \| \| Negative changes in ambulatory status \| 1 \| 0.04% \|; \| \| Pain disability index \| 1 \| 0.04% \|; \| \| Pain with gait assessed by a numerical rating scale \| 1 \| 0.04% \|; \| \| *Patient specific functional scale* \| **1** \| 0.04% \|; \| \| *Groningen activity restriction scale (1-year)* \| **2** \| 0.08% \|; \| Locomotion ability \| Locomotor capability index-5 \| 7+**14** \| 0.85% \| 27; \| \| Modified Locomotor Capability Index \| 1 \| 0.04% \|; \| \| Post-operative ambulatory status (non-ambulatory, wheelchair, outdoor ambulation \| 1 \| 0.04% \|; \| \| Walking ability (no ability, at home/with barrier, no limitation) \| 1 \| 0.04% \|; \| \| Walking ability (with walker assistance) \| 1 \| 0.04% \|; \| \| Walking ability Index: Ability to walk 10 ft on a level (Gait, use of stick, crutch) \| 1 \| 0.04% \|; \| \| Walking ability with support \| 1 \| 0.04% \|; \| \| Walking category (independent community, limited community, limited household) \| 1 \| 0.04% \|; \| \| 100-meter walking ability outdoor and indoor (with/without cane) \| 1 \| 0.04% \|; \| \| Ambulatory status at discharge (ambulatory, ambulatory with assistance, wheelchair, bedridden) \| 1 \| 0.04% \|; \| \| Duration to ambulation (days) \| 1 \| 0.04% \|; \| \| *Functional ambulation level* \| **7** \| 0.28% \|; \| \| *Successful ambulation with prosthesis* \| **6** \| 0.24% \|; \| \| *Maintenance of ambulation* \| **4** \| 0.16% \|; \| \| *Independent Walking/ambulation* \| **3** \| 0.12% \|; \| \| *Walking ability at follow-up* \| **3** \| 0.12% \|; \| \| *Satisfactory ambulation by Walking ability index* \| **2** \| 0.08% \|; \| \| *Ambulation* \| **2** \| 0.08% \|; \| \| *Walking with pneumatic walking aid* \| **1** \| 0.04% \|; \| \| *Appearance and gait at 6 months* \| **1** \| 0.04% \|; \| \| *Locomotor performance after prosthesising* \| **1** \| 0.04% \|; \| \| *Means of ambulation* \| **1** \| 0.04% \|; \| \| *Method of ambulation at discharge* \| **1** \| 0.04% \|; \| \| *Mobilized without crutches* \| **1** \| 0.04% \|; \| \| *Prosthetic gait (mobile in or out of doors with prosthesis)* \| **1** \| 0.04% \|; \| \| *Walking in prosthesis (6-week, 4-month, 12-month)* \| **1** \| 0.04% \|; \| \| *Satisfactory walking results* \| **1** \| 0.04% \|; \| Mobility \| Timed up and go test \| 12+**5** \| 0.69% \| 30; \| \| Timed up and down stairs test \| 1 \| 0.04% \|; \| \| Basic amputee mobility score \| 1 \| 0.04% \|; \| \| Changes in functional mobility (wheelchair, walking ability) and activities of daily living (washing, dressing, toileting) \| 1 \| 0.04% \|; \| \| Interviews regarding mobility (walking with prosthesis, crutch, bedridden, wheelchair) \| 1 \| 0.04% \|; \| \| Life space assessment questionnaire \| 2 \| 0.08% \|; \| \| L-test of functional mobility \| 1 \| 0.04% \|; \| \| Mobility at 2-year (wheelchair, bedridden, ambulatory) \| 1 \| 0.04% \|; \| \| Mobility score \| 1+**1** \| 0.08% \|; \| \| Mobility status (ambulant/non-ambulant) \| 1 \| 0.04% \|; \| \| Pre-amputation mobility \| 1 \| 0.04% \|; \| \| Sitting, standing, and stepping behavior \| 1 \| 0.04% \|; \| \| Stanmore Harold Wood mobility grade \| 1+**1** \| 0.08% \|; \| \| Special Interest Group Amputation Medicine- Workgroup Amputation and Prosthetics mobility scale \| 1+**3** \| 0.16% \|; \| \| Special interest group in amputee medicine \| 3 \| 0.12% \|; \| \| Functional independence measure-mobility subscale \| 1+**2** \| 0.12% \|; \| \| Ambulation \| 1 \| 0.04% \|; \| \| Ambulatory rate (1-year) \| 1 \| 0.04% \|; \| \| Ambulatory status \| 1 \| 0.04% \|; \| \| Amputee mobility predictor \| 1 \| 0.04% \|; \| \| Amputee mobility predictor with/without prosthesis \| 1 \| 0.04% \|; \| \| Post-operative time to mobilization (days) \| 1 \| 0.04% \|; \| \| Amputee Single Item Mobility Measure \| 1 \| 0.04% \|; \| \| *Mobility level* \| **16** \| 0.65% \|; \| \| *Mobility at 6-month* \| **2** \| 0.08% \|; \| \| *Mobility at 1-year* \| **2** \| 0.08% \|; \| \| *Mobility in the home at discharge from rehabilitation* \| **2** \| 0.08% \|; \| \| *Minimum Data Set Activities of Daily Living Long Form Score* \| **1** \| 0.04% \|; \| \| *Mobility unaided without wheelchair* \| **1** \| 0.04% \|; \| \| *Rivermead mobility index* \| **1** \| 0.04% \|; \| Flexibility \| Modified Thomas test \| 1 \| 0.04% \| 1; \| Strength \| Strength (trunk) \| 1 \| 0.04% \| 1; \| Mobility potential \| K-level mobility \| 7+**1** \| 0.32% \| 1; \| Physical activity \| Percentage time in activity (sedentary, light, moderate/vigorous activity) \| 1 \| 0.04% \| 3; \| \| Accelerometer wear time for assessing physical activity \| 1 \| 0.04% \|; \| \| *Improvement of physical function at rehabilitation discharge* \| **1** \| 0.04% \|; \| Prosthesis fit \| Prosthesis fitting \| 8 \| 0.32% \| 7; \| \| Fit and function of the prosthesis (semi-structured interview) \| 1 \| 0.04% \|; \| \| *Successful fitting with prosthesis* \| **66** \| 2.68% \|; \| \| *Independent fitting of prosthesis* \| **1** \| 0.04% \|; \| \| *Rate of prosthetic fitting (1-year)* \| **1** \| 0.04% \|; \| \| *Initial prosthetic casting* \| **1** \| 0.04% \|; \| \| *Readiness for prosthetic fitting* \| **1** \| 0.04% \|; \| Prosthesis use \| Assessed suitability for prosthesis at discharge \| 1 \| 0.04% \| 39; \| \| Duration of prosthesis use (hours) \| 3+**1** \| 0.16% \|; \| \| Duration of prosthesis use (minutes) \| 1 \| 0.04% \|; \| \| Duration of prosthesis use (years) \| 1 \| 0.04% \|; \| \| Environmental factors item bank (assessed effects of environment on prosthetic functioning, accessibility) \| 1 \| 0.04% \|; \| \| Failure to long-term prosthetic use \| 1 \| 0.04% \|; \| \| Foot, socket type, and suspension method of prosthesis \| 1 \| 0.04% \|; \| \| Houghton scale \| 5+**1** \| 0.28% \|; \| \| Peak intra socket pressure analysis (7 to 28 psi using Fujifilm pre-scale film) \| 1 \| 0.04% \|; \| \| Pre-operative intention to use prosthesis \| 1 \| 0.04% \|; \| \| Prosthesis modification \| 1 \| 0.04% \|; \| \| Prosthesis use \| 5 \| 0.20% \|; \| \| Prosthesis use (rate and timing) \| 1 \| 0.04% \|; \| \| Prosthetic use score \| 1 \| 0.04% \|; \| \| Questionnaire for persons with a TFA modified for TTA for daily hours of prosthesis use \| 1 \| 0.04% \|; \| \| Received prosthesis (influence on mortality, major adverse cardiovascular events) \| 1 \| 0.04% \|; \| \| Re-initiation of prosthesis use \| 1 \| 0.04% \|; \| \| % received prosthesis \| 1 \| 0.04% \|; \| \| Type of prosthesis used \| 1 \| 0.04% \|; \| \| *Achievement of prosthesis use* \| **5** \| 0.20% \|; \| \| *Prosthetic prescription rate* \| **5** \| 0.20% \|; \| \| *Prosthetic use at follow-up* \| **5** \| 0.20% \|; \| \| *Prosthesis use (1-year)* \| **3** \| 0.12% \|; \| \| *Prosthesis use (6-month)* \| **3** \| 0.12% \|; \| \| *Rates of abandoning prosthesis use* \| **3** \| 0.12% \|; \| \| *Prosthetic prescription (1-year of amputation)* \| **2** \| 0.08% \|; \| \| *Daily hours of prosthetic wear (12-month, 18-month, 24-month)* \| **1** \| 0.04% \|; \| \| *Need for socket change* \| **1** \| 0.04% \|; \| \| *Prosthesis comfort (6-month)* \| **1** \| 0.04% \|; \| \| *Prosthetic device acquisition* \| **1** \| 0.04% \|; \| \| *Prosthetic restoration* \| **1** \| 0.04% \|; \| \| *Prosthetic use at 6 weeks and 4 months* \| **1** \| 0.04% \|; \| \| *Prosthetic wearing at follow-up* \| **1** \| 0.04% \|; \| \| *Referral for prosthetic rehabilitation* \| **1** \| 0.04% \|; \| \| *Self-reported hours of prosthetic use (6-month)* \| **1** \| 0.04% \|; \| \| *Unsuitable for prosthesis* \| **1** \| 0.04% \|; \| \| *Progress to definitive prosthesis* \| **1** \| 0.04% \|; \| \| *Use of prosthesis on discharge* \| **1** \| 0.04% \|; \| \| *Rate of progression through temporary prostheses* \| **1** \| 0.04% \|; \| Prosthesis-related mobility \| PEQ-mobility subscale \| 7+**1** \| 0.32% \| 8; \| \| Prosthetic limb user survey of mobility \| 5+**1** \| 0.24% \|; \| \| Satisfaction with mobility questionnaire \| 1 \| 0.04% \|; \| \| Walking ability with prosthesis and support \| 1 \| 0.04% \|; \| \| *Mobility with prosthesis* \| **11** \| 0.45% \|; \| \| *Function with a prosthesis (1-year)* \| **2** \| 0.08% \|; \| \| *Independent mobility with prosthesis* \| **1** \| 0.04% \|; \| \| *Mobility with temporary prosthesis* \| **1** \| 0.04% \|; \| Range of motion \| Knee joint range of motion \| 1 \| 0.04% \| 4; \| \| Peak hip extension range of motion \| 1 \| 0.04% \|; \| \| Active range of motion (shoulder, elbow, wrist) \| 1 \| 0.04% \|; \| \| Passive range of motion (shoulder, elbow, wrist) \| 1 \| 0.04% \|; \| Weight bearing \| Weight bearing on remaining leg \| 1 \| 0.04% \| 1; Social functioning \| Social participation \| Participation scale \| 1 \| 0.04% \| 8; \| \| Reintegration to normal living index \| 1+**1** \| 0.08% \|; \| \| PROMIS (participation in social roles and activities) \| 1 \| 0.04% \|; \| \| *Effect on home and work capacity* \| **1** \| 0.04% \|; \| \| *Return to work* \| **1** \| 0.04% \|; \| \| *Community participation (12-month)* \| **1** \| 0.04% \|; \| \| *Social integration (12-month)* \| **1** \| 0.04% \|; \| \| *Low social functioning (6-month)* \| **1** \| 0.04% \|; \| Social relationship \| Quality of relationship inventory \| 1 \| 0.04% \| 2; \| \| Social network index \| 1 \| 0.04% \|; \| Sexual activity \| *Self-reported frequency of sexual activity* \| **1** \| 0.04% \| 3; \| \| *Desire for sexual activity* \| **1** \| 0.04% \|; \| \| *Importance of sexual activity to satisfaction with life* \| **1** \| 0.04% \|; Cognitive functioning \| Cognitive function \| Montreal Cognitive assessment \| 1 \| 0.04% \| 9; \| \| Mini Mental State Examination \| 1 \| 0.04% \|; \| \| Folstein mental state examination (version 1) \| 1 \| 0.04% \|; \| \| *Digit span* \| **1** \| 0.04% \|; \| \| *List learning* \| **1** \| 0.04% \|; \| \| *List recall* \| **1** \| 0.04% \|; \| \| *Semantic fluency* \| **1** \| 0.04% \|; \| \| *Short portable mental status questionnaire* \| **1** \| 0.04% \|; \| \| *Right-left foot recognition* \| **1** \| 0.04% \| | | | | | |
| Delivery of care | Time to receive services | Time interval to delivery of prosthetic and orthotic care services | 2 | 0.08% | 18 |
|  |  | Time to receive angiography | 1 | 0.04% |  |
|  |  | Time to start physiotherapy | 1 | 0.04% |  |
|  |  | Days with no physiotherapy in hospital | 1 | 0.04% |  |
|  |  | Duration of delayed amputation | 1 | 0.04% |  |
|  |  | Reason for delayed amputation | 1 | 0.04% |  |
|  |  | Duration from amputation to rehabilitation | 1 | 0.04% |  |
|  |  | Duration from amputation to prosthesis mounting | 1 | 0.04% |  |
|  |  | Waiting for prosthesis | 1 | 0.04% |  |
|  |  | Days to early walking aid | 1 | 0.04% |  |
|  |  | Duration to revision | 1 | 0.04% |  |
|  |  | Days to compression therapy | 1 | 0.04% |  |
|  |  | Days to casting | 1+**1** | 0.08% |  |
|  |  | *Time until definitive prosthesis* | **5** | 0.20% |  |
|  |  | *Time to prosthetic fitting* | **5** | 0.20% |  |
|  |  | *Time to walking with prosthesis* | **2** | 0.08% |  |
|  |  | *Time to mobilisation* | **2** | 0.08% |  |
|  |  | *Days until patella tendon bearing cast* | **1** | 0.04% |  |
|  | Feasibility | Participant recruitment rate | 3 | 0.12% | 8 |
|  |  | Participant consent rate | 2 | 0.08% |  |
|  |  | Participant response rate | 2 | 0.08% |  |
|  |  | Participant retention rate | 2 | 0.08% |  |
|  |  | Completion of questionnaire rate | 1 | 0.04% |  |
|  |  | Participants reaching follow-up assessment | 1 | 0.04% |  |
|  |  | Time to conduct questionnaires | 1 | 0.04% |  |
|  |  | Recruited participants who provided adequate putative primary effectiveness outcomes data | 1 | 0.04% |  |
|  | Patient satisfaction | Semi-structured interviews for patients (assessing course of disease and treatment, moment amputation discusses 1st time and impact on life, decision making process to amputate, what is important in life, limitations due to disease, doctor’s enquiry about goals, information about amputation, rehabilitation, quality of life after amputation and best moment to talk about amputation) | 1 | 0.04% | 3 |
|  |  | *Satisfaction with mobility (12-month)* | **1** | 0.04% |  |
|  |  | *Satisfaction with treatment of pain* | **1** | 0.04% |  |
|  | Patient experience | Subjective interview regarding experience with nerve block | 1 | 0.04% | 2 |
|  |  | *Experiences of living with an amputation* | **1** | 0.04% |  |
|  | Patient preference | Patient plan to have prosthesis at day 21 | 1 | 0.04% | 1 |
|  | Fidelity | Dose attainment goal | 1 | 0.04% | 1 |
|  | Reliability of scoring | Reliability of Basic amputee mobility score scoring between experienced and inexperienced rater | 1 | 0.04% | 1 |
|  | Global impression of change | Patient global impression of change | 1 | 0.04% | 1 |
|  | Accessibility, quality and adequacy of intervention | Availability of and access to community resources (semi-structured interview) | 1 | 0.04% | 1 |
|  | Caregiver satisfaction | Semi-structured interviews for caregivers (assessing course of disease and treatment, moment amputation discusses 1st time and impact on life, decision making process to amputate, what is important in life, limitations due to disease, doctor’s enquiry about goals, information about amputation, rehabilitation, quality of life after amputation and best moment to talk about amputation) | 1 | 0.04% | 1 |
|  | Clinician satisfaction | Semi-structured interviews for healthcare professional (assessing typical disease process and treatment, when amputation is offered as treatment, communication with patient, when do you see amputation as realistic option, advantages/disadvantages and most important reason for amputation, information about amputation and rehabilitation, important in life for these patients, inquiring after goals, values and preferences, suggestions for improving communication, quality of life after amputation | 1 | 0.04% | 1 |
|  | Barriers to care | Barrier to care | 1 | 0.04% | 1 |
| Emotional functioning/well being | Self-efficacy | Self-efficacy of managing chronic disease questionnaire | 2 | 0.08% | 5 |
|  |  | 6-item self-efficacy for managing chronic disease scale | 1 | 0.04% |  |
|  |  | General self-efficacy scale | 1 | 0.04% |  |
|  |  | Self-efficacy of exercise scale | 1 | 0.04% |  |
|  |  | Washington self-efficacy scale | 1 | 0.04% |  |
|  | Coping ability | Connor-Davidson resilience questionnaire | 2 | 0.08% | 5 |
|  |  | Brief COPE scale to assess dispositional coping strategies | 1 | 0.04% |  |
|  |  | Coping attitudes evaluation scale | 1 | 0.04% |  |
|  |  | *Coping Strategies Questionnaire (6-month)* | **1** | 0.04% |  |
|  |  | *Psychological adaptation to limb loss* | **2** | 0.08% |  |
|  | Motivation | Intrinsic motivation inventory (interest and enjoyment subscale) | 2 | 0.08% | 2 |
|  |  | Motivation and attitude (semi-structured interview) | 1 | 0.04% |  |
|  | Loneliness | University of California, Los Angeles loneliness scale | 1 | 0.04% | 2 |
|  |  | Living alone | 1 | 0.04% |  |
|  | Self-esteem | Rosenberg self-esteem scale | 1 | 0.04% | 1 |
|  | Emotional functioning | *Low emotional functioning (6-month)* | **1** | 0.04% | 1 |
|  | Body image | Amputee body image scale | 1 | 0.04% | 2 |
|  |  | *Body Image Questionnaire (inpatient, in-rehabilitation, after discharge from rehabilitation)* | **1** | 0.04% |  |
| Personal circumstances | Residential status | Living alone | 1 | 0.04% | 6 |
|  |  | Accommodation (independent, wheelchair accessibility, nursing home) | 1 | 0.04% |  |
|  |  | Residential status pre-injury, post-operative (own home, residential home, nursing home) | 1 | 0.04% |  |
|  |  | *Change of residence* | **1** | 0.04% |  |
|  |  | *Living environment* | **1** | 0.04% |  |
|  |  | *Independent living status* | **1** | 0.04% |  |
|  | Employment | Change in employment | 1 | 0.04% | 1 |
| Perceived health status | Health status | Supporting Active Ageing through Multimodal coaching questionnaire | 1 | 0.04% | 3 |
|  |  | EQ-5D utility index | 1 | 0.04% |  |
|  |  | Sickness impact profile-68 | **1** | 0.04% |  |
|  | Perceived exertion | Rate of perceived exertion | 1 | 0.04% | 1 |
| Global quality of life | Prosthesis-related quality of life | Prosthesis evaluation questionnaire | 6 | 0.24% | 4 |
|  |  | Trinity amputation and prosthesis experience scale | 2 | 0.08% |  |
|  |  | Prostheses-specific Trinity amputation and prosthesis experience scales-revised questionnaire | 1 | 0.04% |  |
|  |  | Questionnaire for persons with transfemoral amputation | 1 | 0.04% |  |
|  | Quality of life | Short form-36 | 8+**2** | 0.41% | 12 |
|  |  | World Health Organization Quality of life Bref score | 3 | 0.12% |  |
|  |  | Euroqual-5D | 3+**1** | 0.16% |  |
|  |  | Satisfaction with life scale | 2 | 0.08% |  |
|  |  | Life satisfaction-11 questionnaire | 1 | 0.04% |  |
|  |  | Vascular amputee adapted quality of life questionnaire (adapted from World Health Organization quality of life-BREF and SF-36 questionnaires) | 1 | 0.04% |  |
|  |  | Structured interviews on quality-of-life domains relevant to vascular amputees (mobility, pain, concern about their remaining limb, mental health, independence, social function, personal postoperative experience and feelings at time of interview), additional focus group interviews for further exploration of structured interviews) | 1 | 0.04% |  |
|  |  | 12-item short form health survey | 1 | 0.04% |  |
|  |  | Semi-structured interviews concerning perceptions and experiences of living with amputation on altered mobility, social disruption, psychological impact, socioeconomic factors, accessibility, social support | 1 | 0.04% |  |
|  |  | Semi-structured interview (general health, rehabilitation after amputation, physical exercise, prosthesis, post-amputation phase) | 1 | 0.04% |  |
|  |  | *Quality of life* | **3** | 0.12% |  |
|  |  | *Satisfaction with life (12-month)* | **1** | 0.04% |  |
| **Physiological/clinical** | | | | | |
| Blood and lymphatic system outcomes | Laboratory parameters | Hemoglobin | 7+**3** | 0.41% | 15 |
|  |  | Hematocrit% | 3 | 0.12% |  |
|  |  | Platelet count | 3 | 0.12% |  |
|  |  | Albumin level | 3 | 0.12% |  |
|  |  | Partial thromboplastin time | 2 | 0.08% |  |
|  |  | Potassium level | 2 | 0.08% |  |
|  |  | Fibrinogen level | 1 | 0.04% |  |
|  |  | Sodium level | 1 | 0.04% |  |
|  |  | Erythrocyte sedimentation rate | 2 | 0.08% |  |
|  |  | Total protein | 1 | 0.04% |  |
|  |  | Thrombocyte level | 1 | 0.04% |  |
|  |  | PaCO_2_ | 1 | 0.04% |  |
|  |  | PaO_2_ | 1 | 0.04% |  |
|  |  | Lactate dehydrogenase levels | 1 | 0.04% |  |
|  |  | *Resting Tcpo_2_ (post therapy)* | **1** | 0.04% |  |
|  | Blood transfusion | Blood transfusion | 5+**2** | 0.28% | 6 |
|  |  | Need for blood transfusion | 2 | 0.08% |  |
|  |  | Blood transfusion per kilogram of bodyweight | 1 | 0.04% |  |
|  |  | Mean perioperative blood transfusion (units) | 1 | 0.04% |  |
|  |  | Use of blood transfusion (intra- and post-operative) | 1 | 0.04% |  |
|  |  | Red blood cell transfusion | 1 | 0.04% |  |
|  | Blood loss | Estimated blood loss | 1 | 0.04% | 10 |
|  |  | Total blood loss | 2 | 0.08% |  |
|  |  | Estimated blood loss (<250 ml, >250 ml) | 1 | 0.04% |  |
|  |  | Mean blood loss | 1 | 0.04% |  |
|  |  | Delta total intraoperative blood loss | 1 | 0.04% |  |
|  |  | Bleeding (very good, good, medium, minimal) | 1 | 0.04% |  |
|  |  | *Blood loss* | **2** | 0.08% |  |
|  |  | *Haemorrhage* | **1** | 0.04% |  |
|  |  | *Bleeding requiring 4 units blood (within 72 hours of surgery)* | **1** | 0.04% |  |
|  |  | *Wound bleeding* | **1** | 0.04% |  |
|  | Biomarkers | D-dimer levels | 1 | 0.04% | 2 |
|  |  | *CK-B level* | **1** | 0.04% |  |
|  | Diagnostic test | Oxygen saturation | 1+**1** | 0.08% | 1 |
|  | Anemia | Anemia (pre- and post-operative) | 1 | 0.04% | 1 |
| Cardiovascular outcomes | Diagnostic test | Ankle brachial index | 4 | 0.16% | 11 |
|  |  | Heart rate | 2 | 0.08% |  |
|  |  | Doppler ultrasonography | 1 | 0.04% |  |
|  |  | Echocardiography (ejection fraction) | 1 | 0.04% |  |
|  |  | Electrocardiogram | 1+**1** | 0.08% |  |
|  |  | Cardiovascular assessment prior to amputation (7 to 90 days, >91 days) | 1 | 0.04% |  |
|  |  | Blood pressure | 1 | 0.04% |  |
|  |  | *Resting heart rate* | **1** | 0.04% |  |
|  |  | *Heart rate maximum* | **1** | 0.04% |  |
|  |  | *Blood pressure maximum* | **1** | 0.04% |  |
|  |  | *% predicted heart rate maximum* | **1** | 0.04% |  |
| Endocrine outcomes | Diagnostic test | HbA1c levels | 2 | 0.08% | 1 |
|  | Laboratory parameters | Fasting blood glucose level | 1 | 0.04% | 2 |
|  |  | Blood glucose level | 1 | 0.04% |  |
|  | Physical examination of diabetic neuropathy | Michigan neuropathy screen physical assessment | 1 | 0.04% | 1 |
| General outcomes | Comorbidities | Functional comorbidity index | 5 | 0.20% | 11 |
|  |  | Age-adjusted Charlson comorbidity index | 2 | 0.08% |  |
|  |  | Charlson comorbidity index | 2 | 0.08% |  |
|  |  | Comorbidities | 3 | 0.12% |  |
|  |  | Dysvascular condition scale | 2 | 0.08% |  |
|  |  | *Pulmonary embolism* | **8** | 0.32% |  |
|  |  | *Venous thrombosis (deep vein thrombosis/pulmonary embolism)* | **7** | 0.28% |  |
|  |  | *Deep vein thrombosis* | **8** | 0.32% |  |
|  |  | *Peripheral vascular disease* | **1** | 0.04% |  |
|  |  | *Morbidity* | **3** | 0.12% |  |
|  |  | *Major morbidity* | **1** | 0.04% |  |
|  | Frailty | Modified frailty index | 3 | 0.12% | 5 |
|  |  | Frailty status | 2 | 0.08% |  |
|  |  | Modified frailty index (5-item) | 1 | 0.04% |  |
|  |  | Modified frailty index (11-item) | 1 | 0.04% |  |
|  |  | Risk analysis to classify frailty | 1 | 0.04% |  |
|  | Diagnostic test | Ultrasound | 2 | 0.08% | 2 |
|  |  | Frequency of diagnosis of single/multiple risk factors | 1 | 0.04% |  |
|  | Amputation | Age-standardized rates of major lower limb amputation | 1 | 0.04% | 7 |
|  |  | Stages of amputation (single/double stage) | 1 | 0.04% |  |
|  |  | Incident dysvascular amputation level (according to geographical regions, race/ethnicity) | 1 | 0.04% |  |
|  |  | Amputation of dominant limb | 1 | 0.04% |  |
|  |  | Odds of TFA as compared to TTA or TMT | 1 | 0.04% |  |
|  |  | *Contralateral limb preservation* | **2** | 0.08% |  |
|  |  | *Contralateral limb survival* | **1** | 0.04% |  |
|  | Physical examination | Physical examination | 2 | 0.08% | 2 |
|  |  | *Fever* | **1** | 0.04% |  |
| Immune system outcomes | Laboratory parameters | White blood cell count | 6 | 0.24% | 9 |
|  |  | C-reactive protein level | 4 | 0.16% |  |
|  |  | Lymphocyte level | 1 | 0.04% |  |
|  |  | Neutrophil level | 1 | 0.04% |  |
|  |  | *Oxidative burst capacity* | **1** | 0.04% |  |
|  |  | *Expression of CD-3, 4, 8, 19, 25, 69* | **1** | 0.04% |  |
|  |  | *IFN-gamma* | **1** | 0.04% |  |
|  |  | *Interleukin-4* | **1** | 0.04% |  |
|  |  | *Phagocytosis burst* | **1** | 0.04% |  |
|  | Biomarkers | Neutrophil /Albumin ratio | 1 | 0.04% | 5 |
|  |  | CRP/Albumin ratio | 1 | 0.04% |  |
|  |  | Platelet/lymphocyte ratio | 1 | 0.04% |  |
|  |  | Neutrophil/lymphocyte ratio | 1 | 0.04% |  |
|  |  | Neutrophil/cytokine ratio | 1 | 0.04% |  |
| Infection and infestation outcomes | Infection | Surgical site infection | 6+**1** | 0.28% | 12 |
|  |  | COVID-19 infection | 1 | 0.04% |  |
|  |  | Presence of infection | 1 | 0.04% |  |
|  |  | Post-operative skin infection | 1 | 0.04% |  |
|  |  | *Wound infection* | **15** | 0.61% |  |
|  |  | *Superficial wound infection* | **1** | 0.04% |  |
|  |  | *Deep wound infection* | **1** | 0.04% |  |
|  |  | *Stump infection* | **3** | 0.12% |  |
|  |  | *Wound infection (requiring re-operation)* | **1** | 0.04% |  |
|  |  | *Urinary tract infection* | **4** | 0.16% |  |
|  |  | *Sepsis* | **5** | 0.20% |  |
|  |  | *Lipid peroxidation processes* | 1 | 0.04% |  |
|  | Microbial culture | Microbial culture | 1 | 0.04% | 2 |
|  |  | Wound culture | 1 | 0.04% |  |
| Injury and poisoning outcomes | Falls | Fall incidence | 3+**1** | 0.16% | 3 |
|  |  | Falls per 1000 person years (fatal, non-fatal) | 1 | 0.04% |  |
|  |  | Fall description (activity at time of fall transfer, sit/stand transition, reaching/stationary activity, walking on level/uneven surface, other, curbs/steps, stair ascent/descent; prosthesis status: on, off, fell off, unknown; fall location: community, home) | 1 | 0.04% |  |
|  | Pressure ulcers | Braden scale | 1 | 0.04% | 3 |
|  |  | Pressure injuries present on admission and acquired at hospital (stage 1 to 4) | 1 | 0.04% |  |
|  |  | *Pressure sore incidence* | **2** | 0.08% |  |
|  | Fracture | Frequency of fractures (intracapsular, extracapsular) | 1 | 0.04% | 1 |
| Metabolic and nutritional outcomes | Nutritional control | Dieting i.e., nutritional control (occasional, daily) | 1 | 0.04% | 2 |
|  |  | Nutritional status (>10% weight loss in 6 months before operation) | 1 | 0.04% |  |
|  | Body mass | Body mass index | 2+**1** | 0.12% | 1 |
| Musculoskeletal and connective tissue outcomes | Radiology | X-ray | 3 | 0.12% | 1 |
|  | Diagnostic test | Integrated-EMG during step ascent and descent | 1 | 0.04% | 2 |
|  |  | 3-phase bone scintigraphy | 1 | 0.04% |  |
|  | Contractures | *Contracture of knee joint* | **1** | 0.04% | 4 |
|  |  | *flexion contractures* | **1** | 0.04% |  |
|  |  | *Frequency of contractures* | **1** | 0.04% |  |
|  |  | *Degree of contractures* | **1** | 0.04% |  |
| Nervous system outcomes | General pain | Numerical rating scale | 4 | 0.16% | 12 |
|  |  | Visual analogue scale | 2 | 0.08% |  |
|  |  | Changes/relapse in painful involuntary contractions | 1 | 0.04% |  |
|  |  | Pain | 1 | 0.04% |  |
|  |  | Pain intensity (0 to 10 scale) | 1 | 0.04% |  |
|  |  | Pain score | 1 | 0.04% |  |
|  |  | PROMIS (3a, 8a) short form for pain | 1 | 0.04% |  |
|  |  | Rest pain within 30 days before operation | 1 | 0.04% |  |
|  |  | Verbal rating scale for pain (0 to 10) | 1 | 0.04% |  |
|  |  | Wong-Baker FACES score | 1 | 0.04% |  |
|  |  | *Intraoperative pain in the sciatic nerve distribution* | **1** | 0.04% |  |
|  |  | *Pain during injection* | **1** | 0.04% |  |
|  | Low back pain | Number of low back pain events (prior and after the index amputation) | 1 | 0.04% | 3 |
|  |  | Age at 1st low back pain event | 1 | 0.04% |  |
|  |  | Low back pain (subjective) | 1 | 0.04% |  |
|  | Neuropathic pain | Leeds assessment of neuropathic symptoms and signs scores | 1 | 0.04% | 3 |
|  |  | Modified DN4 questionnaire | 1 | 0.04% |  |
|  |  | *Neuropathic pain scale* | **1** | 0.04% |  |
|  | Pain catastrophizing | Pain catastrophizing scale | 1 | 0.04% | 1 |
|  | Pain relief | Overall benefit of analgesia score | 1 | 0.04% | 4 |
|  |  | *Effective pain relief* | **1** | 0.04% |  |
|  |  | *Pain relief with peripheral block* | **1** | 0.04% |  |
|  |  | *Successful pain control* | **1** | 0.04% |  |
|  | Pain interference | Pain interference scale | 1 | 0.04% | 1 |
|  | Phantom limb pain | Phantom limb pain | 3+**15** | 0.73% | 11 |
|  |  | Phantom limb pain post-operative (subjective) | 1 | 0.04% |  |
|  |  | Phantom pain (no/little vs some/much) | 1 | 0.04% |  |
|  |  | % with phantom limb pain | 1 | 0.04% |  |
|  |  | Brief pain inventory short form (phantom limb pain) | 1 | 0.04% |  |
|  |  | Groningen questionnaire (phantom limb pain and chronic stump pain) | 1 | 0.04% |  |
|  |  | Phantom pain sensation amputee questionnaire | 1 | 0.04% |  |
|  |  | Modified phantom pain questionnaire modified | 1 | 0.04% |  |
|  |  | *Postoperative phantom pain intensity* | **1** | 0.04% |  |
|  |  | *Phantom pain (1-year)* | **1** | 0.04% |  |
|  |  | *Sherman's phantom limb questionnaire* | **1** | 0.04% |  |
|  | Phantom limb sensation | Phantom limb sensations | 1+**6** | 0.28% | 7 |
|  |  | Modified phantom phenomena questionnaire | 1 | 0.04% |  |
|  |  | *Quantitative sensory testing* | **1** | 0.04% |  |
|  |  | *Kinetic sensation* | **1** | 0.04% |  |
|  |  | *Kinesthetic sensation* | **1** | 0.04% |  |
|  |  | *Exteroceptive sensation* | **1** | 0.04% |  |
|  |  | *Super added sensation* | **1** | 0.04% |  |
|  | Radiology | Side of corticospinal tracts, lines, fractional anisotropy and apparent diffusion coefficient assessed by magnetic brain resonance imaging with diffusion tensor imaging | 1 | 0.04% | 1 |
|  | Sensation | Sensation in lower limbs | 1 | 0.04% | 2 |
|  |  | *Paresthesia during injection* | **1** | 0.04% |  |
|  | Residual limb pain | Residual limb pain | 2 | 0.08% | 30 |
|  |  | Stump limb pain | 1+**9** | 0.41% |  |
|  |  | Stump pain (no/little vs some/much) | 1 | 0.04% |  |
|  |  | Stump pain (subjective) | 1 | 0.04% |  |
|  |  | Brief pain inventory short form (residual limb pain) | 1 | 0.04% |  |
|  |  | Euroqual 5D-visual analogue scale | 1 | 0.04% |  |
|  |  | Groningen questionnaire (chronic stump pain) | 1 | 0.04% |  |
|  |  | *Pain (6-month)* | **2** | 0.08% |  |
|  |  | *Pain (3-month)* | **1** | 0.04% |  |
|  |  | *Stump pain (1-year)* | **2** | 0.08% |  |
|  |  | *Pain (3 to 6 months)* | **1** | 0.04% |  |
|  |  | *Stump pain in first 30 days, at 3 months, and at 6 months* | **1** | 0.04% |  |
|  |  | *Visual analogue scale* | **2** | 0.08% |  |
|  |  | *Numerical rating scale* | **2** | 0.08% |  |
|  |  | *McGill Pain Questionnaire* | **3** | 0.12% |  |
|  |  | *Pain characterization and intensity over time* | **1** | 0.04% |  |
|  |  | *Average pain scores per day* | **1** | 0.04% |  |
|  |  | *Chronic stump pain* | **2** | 0.08% |  |
|  |  | *Highest pain score* | **1** | 0.04% |  |
|  |  | *Pain intensity (first 24-hour)* | **1** | 0.04% |  |
|  |  | *Pain intensity/frequency* | **1** | 0.04% |  |
|  |  | *Pain threshold* | **2** | 0.08% |  |
|  |  | *Perioperative pain scores* | **1** | 0.04% |  |
|  |  | *Post-amputation chronic pain* | **2** | 0.08% |  |
|  |  | *Post-operative pain* | **3** | 0.12% |  |
|  |  | *Post-operative wound pain intensity* | **1** | 0.04% |  |
|  |  | *Prolonged limb pain* | **1** | 0.04% |  |
|  |  | *Reflex sympathetic dystrophy in stump* | **1** | 0.04% |  |
|  |  | *Stump allodynia* | **2** | 0.08% |  |
|  |  | *Temporal summation of pain* | **1** | 0.04% |  |
| Psychiatric outcomes | Addiction outcomes | Smoking history | 2 | 0.08% | 4 |
|  |  | Alcohol consumption | 2 | 0.08% |  |
|  |  | Illicit drug use | 2 | 0.08% |  |
|  |  | Tobacco use | 1 | 0.04% |  |
|  | Anxiety | General anxiety disorder-7 | 1 | 0.04% | 2 |
|  |  | Anxiety (Hospital Anxiety and Depression Score) | 2+**1** | 0.12% |  |
|  | Behavior change | Exercise stages of change scale | 1 | 0.04% | 1 |
|  | Delirium | Delirium | 1+**1** | 0.08% | 3 |
|  |  | Length of delirium | 1 | 0.04% |  |
|  |  | Post-operative confusion episodes | 1 | 0.04% |  |
|  | Mental health | Mental health diagnosis | 1 | 0.04% | 6 |
|  |  | Short portable mental status questionnaire | 1 | 0.04% |  |
|  |  | Change in mental status | 1 | 0.04% |  |
|  |  | Psychological impact of symptoms | 1 | 0.04% |  |
|  |  | *Low mental health (6-month)* | **1** | 0.04% |  |
|  |  | *Rate of suicidal ideation* | **1** | 0.04% |  |
|  | Fear of falling | Fear of falling (semi-structured interview) | 1 | 0.04% | 1 |
|  | Depression | Patient health questionnaire-9 | 4+**1** | 0.20% | 6 |
|  |  | Depression (Hospital anxiety and depression scale) | 2+**1** | 0.12% |  |
|  |  | Geriatric depression scale (15-item) | 1 | 0.04% |  |
|  |  | Center for epidemiological studies depression scale | 1 | 0.04% |  |
|  |  | *Depression (6-month)* | **1** | 0.04% |  |
|  |  | *Beck depression inventory (in-patient, in-rehabilitation, after discharge from rehabilitation)* | **1** | 0.04% |  |
| Respiratory, thoracic and mediastinal outcomes | Cardiopulmonary fitness | VO_2_ max | 1 | 0.04% | 1 |
| Renal and urinary outcomes | Laboratory parameters | Creatinine level | 5+**1** | 0.24% | 3 |
|  |  | Blood urea nitrogen | 4 | 0.16% |  |
|  |  | Estimated glomerular filtration rate | 1 | 0.04% |  |
| Skin and subcutaneous tissue outcomes | Stump wound healing | Wound healing | 2+**93** | 3.85% | 18 |
|  |  | Duration to healing (days) | 1+**6** | 0.28% |  |
|  |  | Postoperative wound healing (primary, delayed, no healing) | 1 | 0.04% |  |
|  |  | Residual limb wound healing | 1 | 0.04% |  |
|  |  | Time from amputation to healing (days) | 1 | 0.04% |  |
|  |  | Visual analogue scale (wound healing) | 1 | 0.04% |  |
|  |  | *Primary healing rate* | **6** | 0.24% |  |
|  |  | Healing rate | 1 | 0.04% |  |
|  |  | *Mode of wound healing* | **1** | 0.04% |  |
|  |  | *Stump healing* | **8** | 0.32% |  |
|  |  | *Delayed wound healing* | **4** | 0.16% |  |
|  |  | *Failed primary healing* | **2** | 0.08% |  |
|  |  | *Failure to heal* | **1** | 0.04% |  |
|  |  | *Secondary healing* | **1** | 0.04% |  |
|  |  | *Healing after revision* | **1** | 0.04% |  |
|  |  | *Healing after re-amputation to AKA* | **1** | 0.04% |  |
|  |  | *Not healed at follow-up* | **1** | 0.04% |  |
|  |  | *Stump volume reduction* | **1** | 0.04% |  |
|  | Wound | Chakrabarty scale determined residual limb quality and presence of wound | 1 | 0.04% | 10 |
|  |  | Physical examination of stump wound (presence of edema, discharge, infection) | 1 | 0.04% |  |
|  |  | Presence of residual limb wound | 1 | 0.04% |  |
|  |  | Wound classification (clean, clean-contaminated, contaminated, dirty) | 1 | 0.04% |  |
|  |  | Wound ischemia and foot infections core | 1 | 0.04% |  |
|  |  | *Wounds from prosthesis use (6-month)* | **1** | 0.04% |  |
|  |  | *Skin maceration* | **1** | 0.04% |  |
|  |  | *Decubitus ulcer* | **1** | 0.04% |  |
|  |  | *Wound occurrence* | **2** | 0.08% |  |
|  |  | *Stump defects and type* | **1** | 0.04% |  |
| Vascular outcomes | Chronic limb threatning ischemia staging | Infrainguinal Global Limb Anatomic Staging System  stage (I to III) | 1 | 0.04% | 1 |
|  | Diagnostic test | Doppler ultrasound | 2 | 0.08% | 13 |
|  |  | Duplex ultrasound | 1 | 0.04% |  |
|  |  | Arterial flow: Biphasic, monophasic, no flow (external iliac, femoral, deep artery of thigh, superficial femoral artery) | 1 | 0.04% |  |
|  |  | Intravascular ultrasound | 1 | 0.04% |  |
|  |  | Post-occlusive reactive hyperemia response following applied physical stress | 1 | 0.04% |  |
|  |  | Physical examination of popliteal and femoral pulse (palpable, not palpable, not described) | 1 | 0.04% |  |
|  |  | Transcutaneous oxygen tension by near infrared spectroscopy | 1 | 0.04% |  |
|  |  | Tissue saturation index by near infrared spectroscopy | 1 | 0.04% |  |
|  |  | Distal artery spectral waveform (triphasic, biphasic, monophasic) with Doppler ultrasonography on popliteal artery, anterior tibial artery, and posterior tibial artery | 1 | 0.04% |  |
|  |  | Peak systolic velocity (cm/sec) with Doppler ultrasonography on popliteal artery, anterior tibial artery, and posterior tibial artery | 1 | 0.04% |  |
|  |  | Volume flow (ml/min) with Doppler ultrasonography on popliteal artery, anterior tibial artery, and posterior tibial artery | 1 | 0.04% |  |
|  |  | Arterial diameter (cm) with Doppler ultrasonography on popliteal artery, anterior tibial artery, and posterior tibial artery | 1 | 0.04% |  |
|  |  | Deepest spectroscopy receiver (total hemoglobin, oxyhemoglobin, deoxyhemoglobin) | 1 | 0.04% |  |
|  | Radiology | Computed tomography angiogram | 6 | 0.24% | 4 |
|  |  | Angiogram scores (Grade 1 to 3) for femoropopliteal, infrapopliteal, and inframalleolar grades | 1 | 0.04% |  |
|  |  | Pre-operative angiography to demonstrate vessel run-off | 1 | 0.04% |  |
|  |  | Optical coherence tomography-based microangiography | 1 | 0.04% |  |
| **Resource use** | | | | | |
| Societal/carer burden | Carer burden | Zarit burden interview | 1 | 0.04% | 1 |
|  | Family support | Family support | 1 | 0.04% | 1 |
|  | Social support | Multidimensional scale of perceived social support | 3 | 0.12% | 7 |
|  |  | Modified social support survey | 3 | 0.12% |  |
|  |  | Daily assistance (homecare, relatives) | 1 | 0.04% |  |
|  |  | Informal and formal support (semi-structured interview) | 1 | 0.04% |  |
|  |  | Interpersonal support evaluation list–short-form scale | 1 | 0.04% |  |
|  |  | *Social service use* | **1** | 0.04% |  |
|  |  | *Home care arrangements* | **1** | 0.04% |  |
| Need for further intervention | Hemodialysis | Hemodialysis (admission) | 1 | 0.04% | 2 |
|  |  | *New onset dialysis* | **2** | 0.08% |  |
|  | Emergency procedure | Emergency procedures | 1 | 0.04% | 4 |
|  |  | Need for emergency surgery | 1 | 0.04% |  |
|  |  | *Deep vein thrombosis requiring therapy* | **1** | 0.04% |  |
|  |  | *Requirement for skin graft* | **1** | 0.04% |  |
|  | Respiratory support | Intubation | 1 | 0.04% | 8 |
|  |  | Use of respiratory support (intubation, continuous positive airway support) | 1 | 0.04% |  |
|  |  | *Re-intubation* | **1** | 0.04% |  |
|  |  | *Ventilator dependence* | **1** | 0.04% |  |
|  |  | *Prolonged mechanical ventilation requiring tracheostomy* | **1** | 0.04% |  |
|  |  | *Unplanned intubation for respiratory/cardiac failure* | **1** | 0.04% |  |
|  |  | *Prolonged intubation (48 hours)* | **1** | 0.04% |  |
|  |  | *Ventilatory support (port-operative)* | **1** | 0.04% |  |
|  | Management of fractures | Management of fractures (multiple parallel screws, cemented hemiarthroplasty, uncemented hemiarthroplasty, sliding hip screw, intramedullary nail, cemented hemiarthroplasty, non-operative) | 1 | 0.04% | 1 |
|  | Mobility aid | Use of assistive device | 1 | 0.04% | 10 |
|  |  | Discharge mobility aid | 1+**5** | 0.24% |  |
|  |  | Discharged TKA patients provided/not provided with prosthesis | 1 | 0.04% |  |
|  |  | Lacking assistive devices | 1 | 0.04% |  |
|  |  | Use of aids (pre-injury, post-operative, i.e., none, one stick, two sticks, Zimmer, wheelchair) | 1 | 0.04% |  |
|  |  | *Mobility aids used at follow-up* | **3** | 0.12% |  |
|  |  | *Wheelchair use (dependence)* | **2** | 0.08% |  |
|  |  | *Need for mobility (6-month)* | **1** | 0.04% |  |
|  |  | *Use of early walking aids* | **1** | 0.04% |  |
|  |  | *Ability to use parallel bars/walkers/crutches on discharge* | **1** | 0.04% |  |
|  | Predicting outcome | Estimation of likely outcomes of surgery by the healthcare professionals (surgeons: consultants and registrar level trainees regarding % likelihood prediction of death, morbidity and revision within 30-day) (anesthetists: consultants and registrar level trainees regarding morbidity and mortality at 30-day using visual analogue scale (0 to 100%) | 1 | 0.04% | 2 |
|  |  | Healthcare professionals using existing risk prediction tools | 1 | 0.04% |  |
|  | Prescription of medication | Use of medications | 22+**17** | 1.58% | 5 |
|  |  | Duration of medication use | 2+**3** | 0.20% |  |
|  |  | Dosage of medications | 2+**5** | 0.28% |  |
|  |  | Time until medication (analgesia) | 1 | 0.04% |  |
|  |  | Indication of medications | 1 | 0.04% |  |
|  | Resuscitation | Need for resuscitation | 1 | 0.04% | 1 |
|  | Rehabilitation | Duration of rehabilitation | 3+**6** | 0.36% | 12 |
|  |  | Delivery of physiotherapy | 1 | 0.04% |  |
|  |  | Time of inpatient physical therapy consultation (≤1 day vs >1 day) post-operatively (days) | 1 | 0.04% |  |
|  |  | Frequency of physiotherapy | 1 | 0.04% |  |
|  |  | *Rehabilitation success* | **11** | 0.45% |  |
|  |  | *Degree of rehabilitation* | **6** | 0.24% |  |
|  |  | *Rehabilitation outcome* | **4** | 0.16% |  |
|  |  | *Successful maintained level of rehabilitation at follow-up* | **1** | 0.04% |  |
|  |  | *Rehabilitation accomplishments* | **1** | 0.04% |  |
|  |  | *Rehabilitation status* | **1** | 0.04% |  |
|  |  | *Number and duration of physiotherapy treatments during early walking aid/prosthesis use* | **1** | 0.04% |  |
|  |  | *Weeks to achieve maximum benefit* | **2** | 0.08% |  |
|  | Torniquet use | Duration of torniquet | 1 | 0.04% | 2 |
|  |  | Use of torniquet during operation | 1 | 0.04% |  |
|  | Wound management | Wound vac (admission) | 1 | 0.04% | 2 |
|  |  | Postoperative stump dressing (elastic bandage, Plaster of Paris) | 1 | 0.04% |  |
|  | Use of anesthesia | American Society of Anesthesiologists grade | 6 | 0.24% | 11 |
|  |  | Use of anesthesia | 3 | 0.12% |  |
|  |  | Duration of anesthesia | 2 | 0.08% |  |
|  |  | Use of nerve block | 3 | 0.12% |  |
|  |  | Type of anesthesia | 2 | 0.08% |  |
|  |  | Conversion to general anesthesia | 1 | 0.04% |  |
|  |  | Need for anesthesia | 1 | 0.04% |  |
|  |  | *Onset time of nerve block* | **1** | 0.04% |  |
|  |  | *Sedation* | **2** | 0.08% |  |
|  |  | *Mean sedation score over first 96 hours* | **1** | 0.04% |  |
|  |  | *Sedation on a 1-5 numerical rating scale (first 3 days)* | **1** | 0.04% |  |
|  | Special provisions | Service provision by vascular center (immediate postoperative rigid dressing, specialist physiotherapist in first 14 days, daily inpatient gym, inpatient gym session ≥1-hour, prosthetic center on site when inpatient, prosthetic provision as inpatient, specialist physiotherapy outpatient service) | **1** | 0.04% | 3 |
|  |  | *Use of compression therapy* | **1** | 0.04% |  |
|  |  | *Quality improvement framework compliance* | **1** | 0.04% |  |
|  | Specialist consultation | Experience of specialist (resident/consultant) | 3 | 0.12% | 7 |
|  |  | Specialty of doctor (vascular, non-vascular) | 2 | 0.08% |  |
|  |  | Mental health outpatient visits (≥1 past year) | 1 | 0.04% |  |
|  |  | Visit to outpatient clinics (2 and 6 weeks after discharge) | 1 | 0.04% |  |
|  |  | Outpatient treatment per 1000 person years | 1 | 0.04% |  |
|  |  | Primary care consultation prior to amputation (7 to 30, 31 to 90, 91 to 180, 181 to 365, >365 days) | 1 | 0.04% |  |
|  |  | Palliative care consultation (time of death, amputation) | 1 | 0.04% |  |
|  | Reoperation | Reoperation | 2 | 0.08% | 9 |
|  |  | Reoperation rate | 1 | 0.04% |  |
|  |  | Repeat debridement | 1 | 0.04% |  |
|  |  | Reintervention | 1 | 0.04% |  |
|  |  | Need for re-operation (60-day) | 1 | 0.04% |  |
|  |  | Number of operations required | 1 | 0.04% |  |
|  |  | Unplanned operation (30-day) | 1 | 0.04% |  |
|  |  | *Debridement of stump* | **1** | 0.04% |  |
|  |  | *Further procedures* | **1** | 0.04% |  |
| Economic | Cost | Cost-effectiveness | 1 | 0.04% | 16 |
|  |  | Budget-impact analysis of microprocessor vs. non-microprocessor controlled prosthetic knee | 1 | 0.04% |  |
|  |  | Costs in Euros (prosthesis costs, fall-related medical costs, total costs) | 1 | 0.04% |  |
|  |  | Total summative costs of managing patients in dollars (mean and standard deviation for compensation of employees, theatre costs, radiology costs, laboratory costs, costs of medicines, goods and services for intensive care unit and general wards) | 1 | 0.04% |  |
|  |  | Economic evaluation (key cost drivers, resource usage during first 6-month post-operation i.e., general practitioner consultation, practice nurse consultation, primary care contact, other hospital contact, National Health Service contact, ambulance/paramedic contact, attendance at accident and emergency care, nights spent at hospital, contact at surgical/rehabilitation department, visits to hospital, saw a specialist nurse, saw a physiotherapist/occupational therapist) | 1 | 0.04% |  |
|  |  | Bespoke health resource use questionnaire | 1 | 0.04% |  |
|  |  | Intervention costs with perineural catheter (equipment used: reservoir pump, epidural mini, catheter replacement, staff time, drug acquisition) | 1 | 0.04% |  |
|  |  | *Resource utilization* | **1** | 0.04% |  |
|  |  | *Acute medical costs* | **1** | 0.04% |  |
|  |  | *Post acute medical costs* | **1** | 0.04% |  |
|  |  | *Hospital charges* | **2** | 0.08% |  |
|  |  | *Expenses* | **1** | 0.04% |  |
|  |  | *Hospital costs* | **1** | 0.04% |  |
|  |  | *Cost analysis* | **1** | 0.04% |  |
|  |  | *Financial burden of prosthesis* | **1** | 0.04% |  |
|  |  | *Rehabilitation charges* | **1** | 0.04% |  |
|  | Socioeconomic disadvantage | Area deprivation index | 1 | 0.04% | 1 |
|  | Insurance coverage | National health insurance coverage of amputees | 1 | 0.04% | 1 |
| Hospital | Admission | Admitted to intensive care unit | 3 | 0.12% | 12 |
|  |  | Need for intensive care unit | 2 | 0.08% |  |
|  |  | Admitted to emergency care | 2 | 0.08% |  |
|  |  | Admission to high dependency area/intensive care unit | 1+**1** | 0.08% |  |
|  |  | Admission to operating room | 1 | 0.04% |  |
|  |  | Admission to rehabilitation unit | 1 | 0.04% |  |
|  |  | Admitted from (home, nursing home, inpatient rehabilitation, hospital) | 1 | 0.04% |  |
|  |  | Hospitalizations (per 1000 person years) | 1 | 0.04% |  |
|  |  | Number of patients admitted to nursing home, residential care | 1 | 0.04% |  |
|  |  | Living in a nursing home | 1 | 0.04% |  |
|  |  | Transfer to ward (general, orthopedic) | 1 | 0.04% |  |
|  |  | Transfer to intensive care unit | 1 | 0.04% |  |
|  | Discharge | Discharge destination | 11+**24** | 1.42% | 17 |
|  |  | Days to discharge | 3 | 0.12% |  |
|  |  | Number of patients discharged home | 2 | 0.08% |  |
|  |  | Discharge from clinic | 1 | 0.04% |  |
|  |  | Discharge from hospital | 1 | 0.04% |  |
|  |  | Discharge to independent living | 1 | 0.04% |  |
|  |  | Discharged/not discharged to previous residence | 1 | 0.04% |  |
|  |  | Unplanned discharge from rehabilitation unit | 1 | 0.04% |  |
|  |  | *Discharge to independent living* | **2** | 0.08% |  |
|  |  | *Ultimately returned home* | **1** | 0.04% |  |
|  |  | *Proportion returned to previous living conditions (1-year)* | **1** | 0.04% |  |
|  |  | *Discharge setting* | **1** | 0.04% |  |
|  |  | *Disposition of patients* | **1** | 0.04% |  |
|  |  | *Residential status at discharge* | **1** | 0.04% |  |
|  |  | *Institutionalization* | **1** | 0.04% |  |
|  |  | *Discharge supports* | **2** | 0.08% |  |
|  |  | *Discharge with assistance* | **1** | 0.04% |  |
|  | Duration of operation | Duration of operation | 9+**2** | 0.81% | 1 |
|  | Operative outcome | Return to operation room | 4 | 0.16% | 4 |
|  |  | Intra-operative events (transfusion, hypertension, vasopressor use, hypoxia, tachycardia) | 1 | 0.04% |  |
|  |  | Failure to rescue score with active care curtailed (yes/no) | 1 | 0.04% |  |
|  |  | *Medical stability* | **1** | 0.04% |  |
|  | Operative success | Successful preservation of collateral circulation | 1 | 0.04% | 6 |
|  |  | Success of endovascular aneurysm repair procedure | 1 | 0.04% |  |
|  |  | Rate of successful identification of nerve and perineural catheter placement | 1 | 0.04% |  |
|  |  | Operative success | 1 | 0.04% |  |
|  |  | *Successful surgery after peripheral nerve blockade* | **9** | 0.36% |  |
|  |  | *Long term graft survival* | **1** | 0.04% |  |
|  | Duration of stay | Length of hospital stay | 20+**1** | 0.85% | 13 |
|  |  | Length of intensive care unit stay | 3+**1** | 0.16% |  |
|  |  | Postoperative length of hospital stay | 3 | 0.12% |  |
|  |  | Length of inpatient rehabilitation stay | 1 | 0.04% |  |
|  |  | Length of stay | 1+**51** | 2.11% |  |
|  |  | Length of stay at orthopedic department | 1 | 0.04% |  |
|  |  | Hospital or rehabilitation days | 1 | 0.04% |  |
|  |  | Length of admission | 1 | 0.04% |  |
|  |  | Length of acute rehabilitation unit stay | 1 | 0.04% |  |
|  |  | *Rehabilitation length of stay* | **11** | 0.45% |  |
|  |  | *Time in recovery room* | **1** | 0.04% |  |
|  |  | *Time to discharge* | **1** | 0.04% |  |
|  |  | *Prolonged hospital stay* | **1** | 0.04% |  |
|  | Readmission | Readmission (30-day) | 8 | 0.32% | 12 |
|  |  | Readmission rate (30-day) | 2 | 0.08% |  |
|  |  | Readmission (within 30 days of index discharge) | 1 | 0.04% |  |
|  |  | Readmission (within 90 days of index discharge) | 1 | 0.04% |  |
|  |  | Readmission (within 180 days of index discharge) | 1 | 0.04% |  |
|  |  | Readmission rate | 1 | 0.04% |  |
|  |  | Cause specific re-admission | 1 | 0.04% |  |
|  |  | Re-admission | 1 | 0.04% |  |
|  |  | Unexpected re-admission (30-day) | 1 | 0.04% |  |
|  |  | *Proportion having readmission* | **4** | 0.16% |  |
|  |  | *Occurrence of transfer back to acute service* | **1** | 0.04% |  |
|  |  | *Time to readmission with acute coronary syndrome* | **1** | 0.04% |  |
| **Adverse events** | | | | | |
| Adverse events/effects | Adverse events | Adverse events | 5 | 0.20% | 36 |
|  |  | New York heart association classification | 1 | 0.04% |  |
|  |  | Major adverse cardiovascular events | 2 | 0.08% |  |
|  |  | Incidence of nausea and vomiting (during in-patient stay) | 1 | 0.04% |  |
|  |  | Adverse events within 30-day | 1 | 0.04% |  |
|  |  | Adverse effect/ reactions | 1 | 0.04% |  |
|  |  | Adverse and serious events | 1 | 0.04% |  |
|  |  | Nausea | **1** | 0.04% |  |
|  |  | *Side effect occurrences* | **1** | 0.04% |  |
|  |  | *Stroke* | **14** | 0.57% |  |
|  |  | *Haemorrhagic stroke rate* | **1** | 0.04% |  |
|  |  | *Stroke (1-year)* | **1** | 0.04% |  |
|  |  | *Late stroke* | **1** | 0.04% |  |
|  |  | *Coma (24-hour)* | **1** | 0.04% |  |
|  |  | *Cardiac event* | **1** | 0.04% |  |
|  |  | *Cardiac arrhythmias* | **3** | 0.12% |  |
|  |  | *Cardiac arrest* | **2** | 0.08% |  |
|  |  | *Angina* | **1** | 0.04% |  |
|  |  | *Myocardial infarction* | **15** | 0.61% |  |
|  |  | *Myocardial infarction (1-year)* | **1** | 0.04% |  |
|  |  | *Late Myocardial infarction* | **1** | 0.04% |  |
|  |  | *Congestive heart failure* | **3** | 0.12% |  |
|  |  | *Acute renal failure* | **6** | 0.24% |  |
|  |  | *Renal insufficiency* | **1** | 0.04% |  |
|  |  | *Urinary retention* | **1** | 0.04% |  |
|  |  | *Delayed renal graft function* | **1** | 0.04% |  |
|  |  | *Primary nonfunction* | **1** | 0.04% |  |
|  |  | *Graft loss* | **1** | 0.04% |  |
|  |  | *Pneumonia* | **11** | 0.45% |  |
|  |  | *Pneumothorax* | **1** | 0.04% |  |
|  |  | *Aspiration* | **1** | 0.04% |  |
|  |  | *Venous thromboembolism (at 14 days in first 8 weeks)* | **1** | 0.04% |  |
|  |  | *Skin irritation (6-month)* | **1** | 0.04% |  |
|  |  | *Stump irritation* | **1** | 0.04% |  |
|  |  | *Synovial effusion* | **2** | 0.08% |  |
|  |  | *Leg oedema* | **1** | 0.04% |  |
|  | Complications | Postoperative complications | 10+**4** | 0.57% | 39 |
|  |  | Complications | 4 | 0.16% |  |
|  |  | Complications (30-day) | 2 | 0.08% |  |
|  |  | Gangrene (30-day before operation) | 1 | 0.04% |  |
|  |  | Postoperative myocardial infarction | 1 | 0.04% |  |
|  |  | Amputation complications | 1 | 0.04% |  |
|  |  | Clavien-Dindo severity grading system (post-operative complications) | 1 | 0.04% |  |
|  |  | Clinical course including complications | 1 | 0.04% |  |
|  |  | Inpatient complications | 1 | 0.04% |  |
|  |  | Necrosis % | 1 | 0.04% |  |
|  |  | Overall complications | 1 | 0.04% |  |
|  |  | Postoperative complications (30-day) | 1 | 0.04% |  |
|  |  | Pre-operative complications | 1 | 0.04% |  |
|  |  | Risk factors for wound complications (surgical site infection, wound dehiscence) | 1 | 0.04% |  |
|  |  | Stump complications | 1 | 0.04% |  |
|  |  | Stump revisions/complications | 1 | 0.04% |  |
|  |  | Surgical complications | 1 | 0.04% |  |
|  |  | Wound complications (30-day) | 1 | 0.04% |  |
|  |  | Perioperative complications | 1 | 0.04% |  |
|  |  | *Perioperative systemic complications* | **4** | 0.16% |  |
|  |  | *Cardiorespiratory complications* | **1** | 0.04% |  |
|  |  | *Nerve complications* | **1** | 0.04% |  |
|  |  | *Seroma/hematoma* | **1** | 0.04% |  |
|  |  | *Hematoma* | **1** | 0.04% |  |
|  |  | *Diabetic complications* | **1** | 0.04% |  |
|  |  | *Perforated duodenal ulcer* | **1** | 0.04% |  |
|  |  | *Gastrointestinal tract complications (constipation)* | **1** | 0.04% |  |
|  |  | *Cardiac complications* | **3** | 0.12% |  |
|  |  | *Bleeding complications* | **2** | 0.08% |  |
|  |  | *Block-related complications* | **1** | 0.04% |  |
|  |  | *Renal complications* | **1** | 0.04% |  |
|  |  | *Renal/urological complications* | **1** | 0.04% |  |
|  |  | *Respiratory/pulmonary complications* | **2** | 0.08% |  |
|  |  | *Stump related complications* | **1** | 0.04% |  |
|  |  | *Wound complications* | **17** | 0.69% |  |
|  |  | *Soft tissue complications* | **1** | 0.04% |  |
|  |  | *Postoperative stump necrosis* | **4** | 0.16% |  |
|  |  | *Biopsy-proven acute rejection* | **1** | 0.04% |  |
|  |  | *Wound dehiscence* | **2** | 0.08% |  |
|  | Reamputation/revision | Reamputation | 6 | 0.24% | 42 |
|  |  | Ipsilateral reamputation (1-year) | 2 | 0.08% |  |
|  |  | Revision surgery | 4 | 0.16% |  |
|  |  | Revision to higher level amputation | 3+**15** | 0.73% |  |
|  |  | Revision of major lower limb amputation | 1 | 0.04% |  |
|  |  | Revision (30-day) | 1 | 0.04% |  |
|  |  | Freedom from amputation stump revision/failure | 1 | 0.04% |  |
|  |  | Need for stump revision | 1 | 0.04% |  |
|  |  | Need of revision surgery | 1+**1** | 0.08% |  |
|  |  | Number of revision procedures (1-year) | 1 | 0.04% |  |
|  |  | Re-amputation (1-year) | 2+**1** | 0.12% |  |
|  |  | Re-amputation (30-day) | 3 | 0.12% |  |
|  |  | Re-amputation rate during hospitalization | 1 | 0.04% |  |
|  |  | Reamputation (within 180-day of index discharge) | 1 | 0.04% |  |
|  |  | Reamputation (within 90-day of index discharge) | 1 | 0.04% |  |
|  |  | Risk of contralateral minor lower limb amputation | 1 | 0.04% |  |
|  |  | Additional (ipsilateral/ contralateral) amputation (2-year) | 1 | 0.04% |  |
|  |  | Additional (ipsilateral/ contralateral) amputation (30-day) | 1 | 0.04% |  |
|  |  | Conversion from TTA to TFA | 1 | 0.04% |  |
|  |  | Duration from first to second amputation | 1 | 0.04% |  |
|  |  | Incidence of contralateral major lower limb amputation (30-days after index procedure) | 1 | 0.04% |  |
|  |  | Incidence of ipsilateral reamputation | 1 | 0.04% |  |
|  |  | Ipsilateral reamputation (30-day) | 1 | 0.04% |  |
|  |  | Mean time to TFA progression | 1 | 0.04% |  |
|  |  | Need for reamputation | 1 | 0.04% |  |
|  |  | Proximal major ipsilateral re-amputation | 1 | 0.04% |  |
|  |  | Re-amputation (1-month) | 1 | 0.04% |  |
|  |  | *Stump revision rate* | **9** | 0.36% |  |
|  |  | *Revision to AKA* | **24** | 0.97% |  |
|  |  | *Revision at same level* | **1** | 0.04% |  |
|  |  | *Revision rate* | **26** | 1.05% |  |
|  |  | *Revision or reamputation (30-day)* | **2** | 0.08% |  |
|  |  | *Re-amputation rate* | **40** | 1.62% |  |
|  |  | *Major amputation of the contralateral lower limb* | 15 | 0.61% |  |
|  |  | *Final amputation level* | **3** | 0.12% |  |
|  |  | *Ipsilateral reamputation* | **1** | 0.04% |  |
|  |  | *Time to contralateral amputation* | **1** | 0.04% |  |
|  |  | *Additional amputation rate* | **3** | 0.12% |  |
|  |  | *Time from amputation to revision* | **1** | 0.04% |  |
|  |  | *Re-amputation level* | **1** | 0.04% |  |
|  |  | *Re-amputation timing* | **1** | 0.04% |  |
|  |  | *Late revision or re-amputation* | **1** | 0.04% |  |
| TFA: Transfemoral amputation, TTA: Transtibial amputation, TKA: Through knee amputation, TMT: Transmetatarsal amputation | | | | | |

**Supplementary Table 4.** Details of included studies

| **S.no** | **Study** | **Study design** | **Sample description** | **Reason for amputation**  **Time since amputation** (M ± S.D years) | **Amputation level (Laterality)** | **Context** | **Dodd Framework** | | | |  |
| --- | --- | --- | --- | --- | --- | --- | --- | --- | --- | --- | --- |
|  |  |  |  |  |  |  | **Core area** | **Outcome domain** | **Outcome subdomain** | **Outcome measure or indicator*** | **Outcome type** |
| 1. | Godlwana, et al. [188] | RCT | Int: 28F, 49M (58.5 ± 9.9)  Ct: 26F, 51M (57.7 ± 9.6) | Diabetes/ PVD: 154  (NA) | TFA: 50  TTA: 104  (UL: 154) | Rehabilitation | Life impact | Physical functioning | Mobility | TUG | Performance-related |
|  |  |  |  |  |  |  | Life impact | Physical functioning | Functional independence | Barthel index | Performance-related |
|  |  |  |  |  |  |  | Life impact | Social functioning | Social participation | Participation scale | Patient-reported |
|  |  |  |  |  |  |  | Physiological/ clinical | Nervous system outcomes | Residual limb pain | Euroqual 5D-visual analogue scale | Patient-reported |
|  |  |  |  |  |  |  | Life impact | Perceived health status | Health status | Euroqual 5D-utility index | Patient-reported |
|  |  |  |  |  |  |  | Life impact | Physical functioning | Locomotion ability | Modified Locomotor Capability Index | Patient-reported |
| 2. | Akhtar, et al. [193] | Case series | 2F,4M  (64.1 ± 16.7) | PVD: 6  (NA) | TTA: 6  (UL: 5, BL: 1) | Rehabilitation | Life impact | Global QOL | QOL | SF-36 | Patient-reported |
|  |  |  |  |  |  |  | Life impact | Physical functioning | Prosthesis use | Questionnaire for persons with a TFA modified for TTA for daily hours of prosthesis use | Patient-reported |
|  |  |  |  |  |  |  | Life impact | Physical functioning | Mobility potential | K-level for mobility (K0: no ambulatory potential, K4 ambulatory capacity exceeds basic skills) | Clinician-reported |
|  |  |  |  |  |  |  | Life impact | Physical functioning | Gait: spatiotemporal walking | 6MWT | Performance-related |
|  |  |  |  |  |  |  | Life impact | Physical functioning | Mobility | TUG | Performance-related |
|  |  |  |  |  |  |  | Adverse events | Adverse events/effects | Adverse events | Adverse events (revision surgery, fracture, infection, implant failure, further vascular surgery, additional amputation) | Clinician-reported |
|  |  |  |  |  |  |  | Death | Mortality/survival | Mortality | Death (3 to 5 years) | Clinician-reported |
| 3. | AlMehman, et al. [48] | RCS | 187F, 458M  (57.4 ± 12.0) | PVD: 47  Thromboembolism/acute limb ischemia: 19  Diabetes complications/ diabetic foot: 490  Infection: 73  Trauma: 8  Cancer: 5  Other: 3  (NA) | Hip disarticulation: 2  TFA: 271  TTA: 230  Foot amputation: 33  Forefoot amputation: 33  Toe amputation: 88  (UL: 605, BL: 40) | Acute care | Physiological/ clinical | Nervous system outcomes | Phantom limb pain | Modified phantom pain questionnaire modified  Phantom pain sensation amputee questionnaire | Patient-reported |
|  |  |  |  |  |  |  | Physiological/ clinical | Nervous system outcomes | Neuropathic pain | Modified DN4 questionnaire | Clinician-reported |
| 4. | Brier, et al. [135] | Cross-sectional study | 7F, 130M (62.2 ± 7.7) | Diabetes: 111  PAD: 26  (NA) | TFA: 21  TTA: 75  TMT: 41  (NA) | Rehabilitation | Life impact | Social functioning | Social relationship | Quality of relationship inventory | Patient-reported |
|  |  |  |  |  |  |  | Physiological/ clinical | Psychiatric outcomes | Depression | PHQ-9 | Patient-reported |
|  |  |  |  |  |  |  | Resource use | Societal/carer burden | Social support | Modified social support survey | Patient-reported |
|  |  |  |  |  |  |  | Life impact | Physical functioning | Locomotion ability | Locomotor capability index-5 | Performance-related |
|  |  |  |  |  |  |  | Life impact | Global QOL | QOL | SF-36  Satisfaction with life scale | Patient-reported |
| 5. | Hanafi, et al. [158] | Case Study | 1F (39) | Diabetic foot ulcer Infection: 1  (NA) | TFA: 1  TTA: 1  (BL: 1) | Rehabilitation | Life impact | Physical functioning | Functional independence | Modified Barthel index | Clinician-reported |
|  |  |  |  |  |  |  | Life impact | Physical functioning | Prosthesis fit | Prosthesis fitting | Patient-reported |
|  |  |  |  |  |  |  | Physiological/clinical | Cardiovascular outcome | Diagnostic test | ECG | Clinician-reported |
|  |  |  |  |  |  |  | Physiological/ clinical | Skin and subcutaneous tissue outcomes | Stump wound healing | Wound healing | Clinician-reported |
| 6. | Davie-Smith, et al. [64] | RCS | PAD: 371F, 530M  (71.1)  PAD & diabetes: 252F, 582M (67.5) | PAD: 901  PAD & diabetes: 834  (NA) | TTA: 796  TFA: 575  (BL: 364) | Rehabilitation | Life impact | Physical functioning | Prosthesis fit | Prosthetic limb fit | Clinician-reported |
| 7. | Kwong, et al. [88] | RCS | 35F, 76M (62.3 ± 13.7) | CLI: 111  (NA) | TTA: 111  (UL: 111) | Acute care | Death | Mortality/survival | Mortality | Mortality (30-day)  Mortality (1-year) | Clinician-reported |
|  |  |  |  |  |  |  | Resource use | Need for further intervention | Specialist consultation | Palliative care consultation at time of death, amputation | Clinician-reported |
|  |  |  |  |  |  |  | Resource use | Hospital | Readmission | Readmission (30-day) | Clinician-reported |
|  |  |  |  |  |  |  | Resource use | Hospital | Operative outcome | Return to operating room (30-day) | Clinician-reported |
|  |  |  |  |  |  |  | Adverse events | Adverse events/effects | Complications | 30-day wound complication  Pre-operative complications | Clinician-reported |
|  |  |  |  |  |  |  | Resource use | Hospital | Discharge | Number of patients discharged to home | Clinician-reported |
|  |  |  |  |  |  |  | Resource use | Need for further intervention | Use of medication | Post-operative medications | Clinician-reported |
| 8. | Fard, et al. [69] | RCS | 135F, 247M (71.9 ± 12.5) | Dysvascular: 382  (NA) | TFA/ KDA: 169  TTA: 210  (UL: 248, BL: 31) | Rehabilitation | Resource use | Hospital | Discharge | Number of patients discharged home | Clinician reported |
|  |  |  |  |  |  |  | Resource use | Hospital | Admission | Number of patients admitted to nursing home, residential care | Clinician reported |
|  |  |  |  |  |  |  | Death | Mortality/survival | Mortality | Mortality (1-year) | Clinician reported |
| 9. | Gaffney, et al. [199] | Case-control study | Amputation: 10M (56.3 ± 4.5)  Healthy: 10M (64.6 ± 3.3) | Dysvascular: 10  (16.7 ± 5.2) | TTA: 10  (UL: 10) | Rehabilitation | Physiological/clinical | Musculoskeletal and connective tissue outcome | Diagnostic test | Integrated-EMG during step ascent and descent | Clinician reported |
|  |  |  |  |  |  |  | Life impact | Physical functioning | Gait: spatiotemporal walking | Step ascent completion time  Step descent task completion time | Performance-related |
|  |  |  |  |  |  |  | Life impact | Physical functioning | Gait: kinematics walking | Trunk rotational angular momentum during step ascent  Trunk rotational angular momentum during descent | Performance-related |
| 10. | Shiraev, et al. [116] | RCS | 32F, 237M (68 ± 19.9) | Ischemia: 149  Infection: 82  Malignancy: 19  Trauma: 12  Chronic pain or instability: 7  (NA) | TFA: 121  TKA: 1  TTA: 147  (NA) | Acute care | Death | Mortality/survival | Mortality | Mortality (in-patient)  Mortality (1-year) | Clinician reported |
|  |  |  |  |  |  |  | Adverse events | Adverse events/effects | Complications | Postoperative complications | Clinician reported |
|  |  |  |  |  |  |  | Resource use | Hospital | Duration of stay | Length of hospital stay | Clinician reported |
| 11. | Pourghaderi, et al. [109] | RCS | 23110F, 39181M (66.4 ± 13.7) | Diabetes: 16422  PAD: 16405  Diabetes & PAD: 29464  (NA) | TFA: 21304  TTA: 40987  (NA) | Acute care | Adverse events | Adverse events/effects | Complications | Postoperative complications | Clinician reported |
|  |  |  |  |  |  |  | Death | Mortality/survival | Mortality | Mortality | Clinician reported |
| 12. | Miller, et al. [143] | Cross-sectional study | 5F, 27M (62.6 ± 7.8) | Dysvascular*: 17  Trauma: 15  (NA) | TFA/TKA: 4  TTA: 13  (NA) | Rehabilitation | Life impact | Physical functioning | Gait: spatiotemporal walking | 2MWT | Performance-related |
|  |  |  |  |  |  |  | Life impact | Physical functioning | Mobility | Sitting, standing, and stepping behavior with accelerometry | Performance-related |
|  |  |  |  |  |  |  | Physiological/clinical | General outcomes | Comorbidities | Functional comorbidity Index | Patient-reported |
|  |  |  |  |  |  |  | Life impact | Physical functioning | Prosthesis-related mobility | PEQ-mobility subscale | Patient-reported |
|  |  |  |  |  |  |  | Life impact | Physical functioning | Mobility | Life space assessment questionnaire | Patient-reported |
|  |  |  |  |  |  |  | Life impact | Emotional functioning/wellbeing | Self-efficacy | Self-efficacy of managing chronic disease questionnaire | Patient-reported |
|  |  |  |  |  |  |  | Life impact | Emotional functioning/well being | Coping ability | Connor-Davidson resilience questionnaire | Patient-reported |
|  |  |  |  |  |  |  | Life impact | Physical functioning | Impact of disease/condition on ADL | 12-itemworld Health Organization disability assessment schedule 2.0 | Patient-reported |
| 13. | Batten, et al. [170] | PCS | 25F, 85M (63 ± 13) | Diabetes/PVD/venous: 77  Trauma: 14  Tumor: 7  Infection: 8  Other: 4  (NA) | TFA: 30  TTA: 78  KDA: 2  (UL: 110) | Rehabilitation | Life impact | Physical functioning | Mobility potential | K-Level determined by Amputee mobility predictor | Clinician-reported |
|  |  |  |  |  |  |  | Life impact | Physical functioning | Gait: spatiotemporal walking | 10-meter walk test | Performance-related |
|  |  |  |  |  |  |  | Life impact | Physical functioning | Mobility | Functional independence measure-mobility subscale | Clinician-reported |
|  |  |  |  |  |  |  | Resource use | Hospital | Duration of stay | Length of stay | Clinician-reported |
|  |  |  |  |  |  |  | Resource use | Need for further intervention | Mobility aid | Discharge mobility aid | Clinician-reported |
| 14. | Kline, et al. [203] | Quasi-experimental study (pre vs post-test) | 4M (66.7 ± 4.8) | Diabetes/PAD: 4  (2.2 ± 1) | TTA: 4  (UL: 4) | Rehabilitation | Physiological/clinical | Endocrine outcomes | Physical examination of diabetic neuropathy | Michigan neuropathy screen physical assessment | Patient-reported |
|  |  |  |  |  |  |  | Physiological/clinical | General outcomes | Comorbidities | Functional comorbidity index | Patient-reported |
|  |  |  |  |  |  |  | Life impact | Physical functioning | Prosthesis use | Foot, socket type, and suspension method of prosthesis  Houghton scale | Patient-reported |
|  |  |  |  |  |  |  | Life impact | Physical functioning | Prosthesis-related mobility | PEQ-mobility subscale | Patient-reported |
|  |  |  |  |  |  |  | Life impact | Physical functioning | Gait: spatiotemporal walking | Gait speed (self-selected, fast gait)  Step length asymmetry index (self-selected, fast gait)  Stance time asymmetry index (self-selected, fast gait) | Performance-related |
|  |  |  |  |  |  |  | Life impact | Emotional functioning/well being | Motivation | Intrinsic motivation- Interest/enjoyment subscale | Patient-reported |
| 15. | Kwak, et al. [87] | RCS | Diabetes: 2F, 16M (59.3 ± 7.8)  Non-Diabetes: 3F, 23M (56.6 ± 16.5) | Diabetes: 18  Non-Diabetes: PVD: 5, Trauma: 17, Cancer: 2  (NA) | TTA: 44  UL: 44 | Rehabilitation | Life impact | Physical functioning | Locomotion ability | 100-meter walking ability outdoor and indoor (with/without cane) | Performance-related |
| 16. | Jayaraman, et al. [197] | Randomized crossover design | 6F, 4M (63 ± 9) | Circulation problems: 6  Hypertension/diabetes/CAD: 2  Diabetes/circulation problems: 1  Hypertension/circulation problems: 1  (5.8± 8.1) | TFA: 10  (UL: 10) | Rehabilitation | Life impact | Physical functioning | Gait: spatiotemporal walking | 10-meter walk test  6MWT | Performance-related |
|  |  |  |  |  |  |  | Life impact | Physical functioning | Balance | BBS  Four square step test | Performance-related |
|  |  |  |  |  |  |  | Life impact | Physical functioning | Mobility | Amputee mobility predictor  TUG | Performance-related |
|  |  |  |  |  |  |  | Life impact | Physical functioning | Balance confidence | Modified falls efficacy scale | Patient-reported |
|  |  |  |  |  |  |  | Life impact | Global QOL | Prosthesis-related QOL | PEQ | Patient-reported |
|  |  |  |  |  |  |  | Adverse events | Adverse events/effects | Adverse events | Adverse events: Falls, skin-related issues | Clinical reported |
| 17. | Kimble [162] | Case study | 1F (59) | Intravascular coagulation: 1  (NA) | TTA: 1  (BL: 1) | Rehabilitation | Life impact | Physical functioning | Gait: spatiotemporal walking | 10-meter walk test  6MWT | Performance-related |
|  |  |  |  |  |  |  | Life impact | Physical functioning | Functional independence | Functional independence measure | Clinician-reported |
| 18. | Siriwardena, et al. [118] | RCS | 598 (NA) | Arteriosclerotic: 598  (NA) | TTA: 310  TFA: 248  Gritti Stokes: 95  (UL: 653  BL: 55) | Rehabilitation | Life impact | Physical functioning | Locomotion ability | Walking ability Index: Ability to walk 10 ft on a level (gait, use of stick, crutch) | Performance related |
| 19. | Wong, et al. [152] | Cross-sectional study | 85F, 183M (55.3 ± 14.9) | Vascular: 130  Nonvascular: 130  (6.8±12.6) | TFA: 105  TTA: 138  (NA) | Community | Life impact | Physical functioning | Balance confidence | ABC scale | Patient-reported |
|  |  |  |  |  |  |  | Physiological/clinical | Injury and poisoning outcomes | Falls | Fall incidence | Clinician reported |
|  |  |  |  |  |  |  | Life impact | Global QOL | Prosthesis-related QOL | PEQ | Patient-reported |
|  |  |  |  |  |  |  | Life impact | Physical functioning | Mobility | TUG | Performance-related |
|  |  |  |  |  |  |  | Life impact | Physical functioning | Gait: spatiotemporal walking | 2MWT | Performance-related |
|  |  |  |  |  |  |  | Life impact | Physical functioning | Balance | BBS | Performance-related |
|  |  |  |  |  |  |  | Life impact | Physical functioning | Prosthesis use | Houghton scale | Patient-reported |
|  |  |  |  |  |  |  | Life impact | Delivery of care | Barriers to care | Barriers to care | Patient-reported |
| 20. | Frengopoulos, et al. [70] | RCS | 38F, 92M  (66.2 ± 11.1) | Primary etiology:  Diabetes: 75  PVD: 49  Diabetes and PVD: 6  Secondary etiology:  Infection: 54  Peripheral neuropathy: 6  Osteomyelitis: 6  Trauma: 1  No secondary etiology: 63  (0.48±0.90) | TTA: 103  TFA: 27  (UL: 130) | Rehabilitation | Life impact | Cognitive functioning | Cognitive function | Montreal Cognitive assessment | Performance-related |
|  |  |  |  |  |  |  | Life impact | Physical functioning | Mobility | L-test of functional mobility | Performance-related |
|  |  |  |  |  |  |  | Life impact | Physical functioning | Gait: spatiotemporal walking | 2MWT | Performance-related |
| 21. | Zorn, et al. [169] | Case study | 1F  (61) | PAD: 1  (NA) | TFA: 1  (UL: 1) | Acute care | Physiological/clinical | General outcomes | Physical examination | Physical examination | Clinician-reported |
|  |  |  |  |  |  |  | Physiological/clinical | Musculoskeletal and connective tissue outcomes | Diagnostic test | 3-phase bone scintigraphy | Clinician reported |
|  |  |  |  |  |  |  | Physiological/clinical | Musculoskeletal and connective tissue outcomes | Radiology | X-ray | Clinician reported |
|  |  |  |  |  |  |  | Physiological/clinical | General outcomes | Diagnostic test | Ultrasound | Clinician reported |
|  |  |  |  |  |  |  | Adverse events | Adverse events/effects | Adverse events | Adverse effect/ reactions | Patient reported |
|  |  |  |  |  |  |  | Life impact | Physical functioning | Prosthesis use | Duration of prosthesis use (hours) | Patient reported |
|  |  |  |  |  |  |  | Life impact | Physical functioning | Prosthesis fit | Prosthesis fit | Patient reported |
| 22. | Norvell, et al. [178] | PCS | 2F, 111M  (63.5 ± 8.1) | Diabetes/ PVD: 113  (NA) | TMT: 26  TTA: 59  TFA: 28  (UL: 113) | Rehabilitation | Life impact | Physical functioning | Mobility | Amputee Single Item Mobility Measure | Patient-reported |
|  |  |  |  |  |  |  | Life impact | Physical functioning | Locomotion ability | Locomotor Capabilities index-5 | Patient-reported |
|  |  |  |  |  |  |  | Life impact | Physical functioning | Prosthesis use | Duration of prosthesis use (hours) | Patient-reported |
|  |  |  |  |  |  |  | Life impact | Global QOL | Prosthesis-related QOL | Trinity amputation and prosthesis experience scale | Patient-reported |
|  |  |  |  |  |  |  | Life impact | Physical functioning | Prosthesis-related mobility | Satisfaction with mobility questionnaire | Patient-reported |
| 23. | Wong, et al. [183] | PCS | One time: 74F, 159M  (55.8 ± 15.1)  Committed: 9F, 21M  (54.9 ± 15.7) | One tine: Dysvascular: 119  Trauma, cancer, congenital: 103  (6.4 ± 12.3)  Committed: Dysvascular: 11  Trauma, cancer, congenital: 18  (4.2 ± 8.9) | One time: TFA: 73  KDA: 1  TTA: 108  Ankle disarticulation: 1  (UL: 183, BL: 20)  Committed: TFA: 11  KDA: 1  TTA: 14  Ankle disarticulation: 0  (UL: 24, BL: 5) | Community | Life impact | Physical functioning | Balance confidence | ABC-scale | Patient-reported |
|  |  |  |  |  |  |  | Life impact | Physical functioning | Prosthesis use | Houghton Scale | Patient-reported |
|  |  |  |  |  |  |  | Life impact | Global QOL | Prosthesis-related QOL | PEQ | Patient-reported |
|  |  |  |  |  |  |  | Life impact | Physical functioning | Balance | BBS | Performance-related |
|  |  |  |  |  |  |  | Life impact | Physical functioning | Gait: spatiotemporal walking | 2MWT | Performance-related |
|  |  |  |  |  |  |  | Life impact | Physical functioning | Locomotion ability | Walking category (independent community, limited community, limited household) | Patient-reported |
|  |  |  |  |  |  |  | Physiological/clinical | Metabolism and nutrition outcome | Nutritional control | Dieting i.e., nutritional control (occasional, daily) | Patient-reported |
| 24. | Torbjörnsson, et al. [182] | PCS | Non-walker: 25F, 25M  (79)  Walker: 4F, 19M  (70) | PAD :78  (NA) | Non-walker: TTA: 40  TFA: 10  (UL: 50)  Walker: TTA: 20  TFA: 3  (UL: 23) | Community | Life impact | Global QOL | QOL | Euroqual-5D-3L | Patient-reported |
|  |  |  |  |  |  |  | Life impact | Physical functioning | Mobility | Stanmore Harold Wood mobility grade | Clinician-reported |
|  |  |  |  |  |  |  | Life impact | Physical functioning | Prosthesis use | Houghton scale | Patient-reported |
| 25. | Laroche, et al. [175] | PCS | 8F, 22M  (66.8) | PAD: 30  (NA) | TTA: 30  (UL: 30) | Rehabilitation | Physiological/ clinical | Nervous system outcomes | General pain | Visual analogue scale pain | Patient-reported |
|  |  |  |  |  |  |  | Physiological/ clinical | Skin and subcutaneous tissue outcomes | Stump wound healing | Visual analogue scale wound healing | Patient-reported |
|  |  |  |  |  |  |  | Life impact | Physical functioning | Gait: spatiotemporal walking | 2MWT | Performance-related |
|  |  |  |  |  |  |  | Physiological/ clinical | Vascular outcomes | Diagnostic test | Transcutaneous oxygen tension by near infrared spectroscopy  Tissue saturation index by near infrared spectroscopy  Deepest spectroscopy receiver (total hemoglobin, oxyhemoglobin, deoxyhemoglobin) by near infrared spectroscopy | Clinician-reported |
| 26. | Christensen, et al. [137] | Cross-sectional study | Amputee: 9M  (57 ± 4)  Healthy: 9M  (60 ± 5) | Dysvascular etiology (diabetes, PAD): 9  (1.4 ± 0.4) | TTA: 9  (UL: 9) | Rehabilitation | Life impact | Physical functioning | Gait: kinetics | Index scores of peak sagittal plane moment of knee, hip, ankle joints  Peak vertical ground reaction forc  Vertical impulse during overground walking and step ascent | Performance-related |
| 27. | Christiansen, et al. [186] | RCT | Intervention: 3F, 16M  (62)  Control: 19M  (65) | Intervention: Dysvascular (diabetes/PAD): 19  (0.30)  Control: Dysvascular (diabetes/PAD): 19  (0.38) | TTA: 38  (UL: 38) | Community | Life impact | Physical functioning | Mobility | TUG | Performance-related |
|  |  |  |  |  |  |  | Life impact | Physical functioning | Gait: spatiotemporal walking | 2MWT  Gait speed  Daily step count | Performance-related |
|  |  |  |  |  |  |  | Life impact | Physical functioning | Impact of disease/condition on ADL | World Health Organization disability assessment schedule 2.0 | Patient-reported |
|  |  |  |  |  |  |  | Life impact | Emotional functioning/wellbeing | Self-efficacy | 6-item self-efficacy for managing chronic disease scale | Patient-reported |
|  |  |  |  |  |  |  | Life impact | Physical functioning | Prosthesis-related mobility | PEQ-mobility subscale | Patient-reported |
|  |  |  |  |  |  |  | Life impact | Physical functioning | Physical activity | Percentage time in activity (sedentary, light, moderate/vigorous activity) | Performance-related |
| 28. | Atallah, et al. [194] | Case series | 3F, 2M  (67.8 ± 12.1) | PAD: 5  (8.4 ± 10.7) | TTA: 5  (UL: 5) | Acute care, rehabilitation | Physiological/ clinical | Nervous system outcomes | General pain | Numerical rating scale for pain | Patient-reported |
|  |  |  |  |  |  |  | Life impact | Physical functioning | Gait: spatiotemporal walking | Static weight bearing and ability to walk (without prosthesis/ with light prosthesis/ with Osseo integrated implant)  6MWT | Performance-related |
|  |  |  |  |  |  |  | Adverse events | Adverse events/effects | Adverse events | Adverse events after Osseo integration (infection, fracture, implant failure) | Clinician-reported |
|  |  |  |  |  |  |  | Death | Mortality/survival | Mortality | Mortality | Clinician-reported |
|  |  |  |  |  |  |  | Adverse events | Adverse events/effects | Re-amputation/revision | Re-amputation  Revision surgery | Clinician-reported |
|  |  |  |  |  |  |  | Life impact | Global QOL | Prosthesis-related QOL | Questionnaire for persons with transfemoral amputation | Patient-reported |
|  |  |  |  |  |  |  | Life impact | Physical functioning | Prosthesis use | Prosthetic use score | Patient-reported |
|  |  |  |  |  |  |  | Life impact | Physical functioning | Mobility potential | K-level assessment of mobility (K0: no ability, K4: ability/potential for ambulation exceeding basic ambulation) | Clinician-reported |
|  |  |  |  |  |  |  | Life impact | Physical functioning | Mobility | TUG | Performance-related |
|  |  |  |  |  |  |  | Life impact | Global QOL | QOL | SF-36 | Patient-reported |
| 29. | Miller, et al. [201] | Cross-sectional study (secondary analysis) | Veteran: 20M  (65.8 ± 8.4)  Non-veteran: 18M  (61 ± 9.4) | Veteran: Dysvascular: 20  (0.28 ± 0.12)  Non-veteran: Dysvascular: 18  (0.41 ± 0.08) | Veteran: TTA: 20  (UL: 20)  Non-veteran: TTA: 18  (UL: 18) | Rehabilitation | Life impact | Physical functioning | Impact of disease/condition on ADL | World Health Organization disability assessment schedule 2.0 | Patient-reported |
|  |  |  |  |  |  |  | Life impact | Emotional functioning/wellbeing | Self-efficacy | Self-efficacy of managing chronic disease questionnaire | Patient-reported |
|  |  |  |  |  |  |  | Life impact | Physical functioning | Prosthesis-related mobility | PEQ-mobility subscale | Patient-reported |
| 30. | Czerniecki, et al. [62] | RCS | No re-amputation: 40F, 3937M  (66.6 ± 10.2)  Re-amputation: 8F, 1275M  (64.9 ± 9.5) | No re-amputation: Diabetes/PVD: 3977  (NA)  Re-amputation: Diabetes/PVD: 1283  (NA) | No re-amputation: TMT: 735  TTA: 1817  TFA: 1425  (UL: 3977)  Re-amputation: TMT: 496  TTA: 634  TFA: 153  (UL: 1283) | Acute care | Physiological/clinical | Psychiatric outcomes | Mental health | Mental health diagnosis | Clinician-reported |
|  |  |  |  |  |  |  | Resource use | Need for further intervention | Specialist consultation | Mental health outpatient visits (≥1 past year) | Clinician-reported |
|  |  |  |  |  |  |  | Physiological/clinical | Metabolism and nutrition outcome | Nutritional control | Nutritional status (>10% weight loss in 6 months before operation) | Clinician-reported |
|  |  |  |  |  |  |  | Physiological/clinical | Psychiatric outcomes | Addiction outcomes | Illicit drug use within 1 year before operation  Smoking history  >2 alcoholic drinks/day in past 2 weeks | Patient-reported |
|  |  |  |  |  |  |  | Life impact | Physical functioning | Functional independence | Physical function (independent, partially dependent, totally dependent) | Clinician-reported |
|  |  |  |  |  |  |  | Resource use | Need for further intervention | Use of medication | Medication (outpatient anticoagulation, antiplatelet medication use) | Clinician-reported |
|  |  |  |  |  |  |  | Physiological/clinical | Blood and lymphatic system outcomes | Laboratory parameters | Platelet count  Potassium levels  Hematocrit % | Clinician-reported |
|  |  |  |  |  |  |  | Physiological/clinical | Renal and urinary outcomes | Laboratory parameters | Blood urea nitrogen  Estimated glomerular filtration rate | Clinician-reported |
|  |  |  |  |  |  |  | Physiological/clinical | Immune system outcomes | Laboratory parameters | White blood cell count | Clinician-reported |
|  |  |  |  |  |  |  | Physiological/ clinical | Nervous system outcomes | General pain | Rest pain within 30 days before operation | Patient-reported |
|  |  |  |  |  |  |  | Adverse events | Adverse events/effects | Complications | Gangrene within 30 days before operation | Clinician-reported |
|  |  |  |  |  |  |  | Physiological/clinical | Cardiovascular outcomes | Diagnostic test | Abnormal ankle brachial pressure index | Clinician-reported |
|  |  |  |  |  |  |  | Adverse events | Adverse events/effects | Re-amputation/revision | Re-amputation (1-year) | Clinician-reported |
| 31. | Lee, et al. [204] | Qualitative descriptive study | 11F, 22M  (56.7 ± 16.1) | Dysvascular: 17  Trauma: 10  Cancer: 3  Congenital: 1  Infection/other: 2  (8.9 ± 12.4) | TTA: 21  TFA: 8  (UL: 25, BL: 4) | Rehabilitation | Life impact | Physical functioning | Energy efficiency | Physical activity scale for individuals with physical disabilities questionnaire (energy expenditure with metabolic equivalent hours per day) | Patient-reported |
|  |  |  |  |  |  |  | Life impact | Delivery of care | Accessibility, quality and adequacy of intervention | Availability of and access to community resources (semi-structured interview) | Patient-reported |
|  |  |  |  |  |  |  | Life impact | Physical functioning | Impact of disease/condition on ADL | Impact of pain and comorbid conditions (semi-structured interview) | Patient-reported |
|  |  |  |  |  |  |  | Life impact | Emotional functioning/well being | Motivation | Motivation and attitude (semi-structured interview) | Patient-reported |
|  |  |  |  |  |  |  | Physiological/clinical | Psychiatric outcomes | Fear of falling | Fear of falling (semi-structured interview) | Patient-reported |
|  |  |  |  |  |  |  | Life impact | Physical functioning | Prosthesis fit | Fit and function of the prosthesis (semi-structured interview) | Patient-reported |
|  |  |  |  |  |  |  | Resource use | Societal/carer burden | Social support | Informal and formal support (semi-structured interview) | Patient-reported |
| 32. | García, et al. [73] | RCS | 67F, 142M  (71.8 ± 12.4) | Acute ischemia: 9  Isolated infection: 19  Critical ischemia: 110  Ischemia and infection: 71  (NA) | TTA: 209  (NA) | Acute care | Death | Mortality/survival | Mortality | Mortality (within 1-month)  Mortality (1-year) | Clinician-reported |
|  |  |  |  |  |  |  | Life impact | Physical functioning | Mobility | Ambulation | Clinician-reported |
|  |  |  |  |  |  |  | Life impact | Physical functioning | Prosthesis fit | Prosthesis fitting | Patient-reported |
| 33. | Chopra, et al. [58] | RCS | 59F, 147M  (63 ± 13.5) | Critical limb ischemia: 154  Acute limb ischemia: 23  Infection: 59  Other causes: 20  (NA) | TTA: 190  TKA: 3  TFA: 16  (NA) | Acute care | Death | Mortality/survival | Mortality | Mortality (30-day) | Clinician-reported |
|  |  |  |  |  |  |  | Adverse events | Adverse events/effects | Complications | Amputation complications (documented major falls, stump dehiscence, surgical revision, higher amputation) | Clinician-reported |
|  |  |  |  |  |  |  | Resource use | Hospital | Duration of stay | Hospital or rehabilitation days | Clinician-reported |
|  |  |  |  |  |  |  | Resource use | Hospital | Discharge | Postamputation discharge (home, nursing facility, rehabilitation) | Clinician-reported |
|  |  |  |  |  |  |  | Physiological/clinical | General outcomes | Frailty | Modified frailty index | Clinician-reported |
|  |  |  |  |  |  |  | Physiological/clinical | Psychiatric outcomes | Addiction outcomes | Illicit drug use  Alcohol abuse  Tobacco use | Patient-reported |
|  |  |  |  |  |  |  | Physiological/clinical | Cardiovascular outcomes | Diagnostic test | Ankle brachial index | Clinician-reported |
|  |  |  |  |  |  |  | Physiological/clinical | Immune system outcomes | Laboratory parameters | White blood cell count | Clinician-reported |
|  |  |  |  |  |  |  | Physiological/clinical | Immune system outcomes | Biomarkers | Neutrophil-cytokine ratio | Clinician-reported |
|  |  |  |  |  |  |  | Physiological/clinical | Blood and lymphatic system outcomes | Laboratory parameters | Hemoglobin  Hematocrit % | Clinician-reported |
|  |  |  |  |  |  |  | Physiological/ clinical | Skin and subcutaneous tissue outcomes | Stump wound healing | Wound ischemia and foot infections core | Clinician-reported |
|  |  |  |  |  |  |  | Physiological/clinical | Cardiovascular outcomes | Diagnostic test | Ankle brachial index | Clinician-reported |
|  |  |  |  |  |  |  | Physiological/clinical | Metabolism and nutrition outcome | Body mass | BMI | Clinician-reported |
|  |  |  |  |  |  |  | Death | Mortality/survival | Survival | Survival (1-year) | Clinician-reported |
|  |  |  |  |  |  |  | Life impact | Physical functioning | Mobility | Ambulatory rate (1-year) | Clinician-reported |
|  |  |  |  |  |  |  | Resource use | Societal/carer burden | Family support | Family support | Patient-reported |
| 34. | Cheng, et al. [136] | Cross-sectional study | 36F, 82M  (60.8 ± 12.9) | Dysvascular: 118  (15.3 ± 34.8) | TTA/TFA: 118  (NA) | Acute care | Resource use | Hospital | Duration of stay | Length of acute rehabilitation unit stay | Clinician-reported |
|  |  |  |  |  |  |  | Resource use | Hospital | Discharge | Unplanned discharge from rehabilitation unit | Clinician-reported |
|  |  |  |  |  |  |  | Life impact | Physical functioning | Functional independence | Functional independence measure | Clinician-reported |
|  |  |  |  |  |  |  | Physiological/clinical | Immune system outcomes | Laboratory parameters | White blood cell counts at admission | Clinician-reported |
|  |  |  |  |  |  |  | Physiological/clinical | Blood and lymphatic system outcomes | Laboratory parameters | Hemoglobin at admission | Clinician-reported |
|  |  |  |  |  |  |  | Physiological/clinical | Renal and urinary outcomes | Laboratory parameters | Creatinine at admission | Clinician-reported |
|  |  |  |  |  |  |  | Resource use | Need for further intervention | Wound management | Wound vac at admission | Clinician-reported |
|  |  |  |  |  |  |  | Resource use | Need for further intervention | Hemodialysis | Hemodialysis at admission | Clinician-reported |
|  |  |  |  |  |  |  | Resource use | Need for further intervention | Use of medication | Intravenous antibiotic use | Clinician-reported |
| 35. | Richardson, et al. [150] | Cross-sectional study | 17F, 72M  (65.5 ± 11.4) | Dysvascular: 89  (2.9 ± 4.6) | TTA: 57  TFA: 32  (UL: 85, BL: 4) | Rehabilitation | Physiological/ clinical | Nervous system outcomes | Phantom sensation | Modified phantom phenomena questionnaire | Patient-reported |
|  |  |  |  |  |  |  | Physiological/ clinical | Nervous system outcomes | General pain | Numerical rating scale | Patient-reported |
|  |  |  |  |  |  |  | Life impact | Physical functioning | Impact of disease/condition on ADLs | Effects of phantom phenomena on daily activities | Patient-reported |
|  |  |  |  |  |  |  | Resource use | Need for further intervention | Use of medication | Analgesic use | Patient-reported |
| 36. | Pandit, et al. [106] | RCS | Frail: 440F, 783M  (73 ± 4)  Non-frail: 1036F, 1959M  (73 ± 9) | Frail: PAD: 1223  (NA)  Non-frail: PAD: 2995  (NA) | Frail: TMT: 306  TTA: 455  TFA: 462  (NA)  Non-frail: TMT: 809  TTA: 1138  TFA: 1048  (NA) | Acute care | Physiological/clinical | General outcomes | Frailty | Frail status | Clinician-reported |
|  |  |  |  |  |  |  | Death | Mortality/survival | Mortality | Mortality (30-day) | Clinician-reported |
|  |  |  |  |  |  |  | Adverse events | Adverse events/effects | Complications | Complications (30-day) | Clinician-reported |
|  |  |  |  |  |  |  | Resource use | Hospital | Readmission | Readmission (30-day) | Clinician-reported |
| 37. | Pandit, et al. [107] | RCS | Frail: 2776F, 5905M  (76 ± 9) | PAD: 8681  (NA) | TTA: 4493  TFA: 3248  (NA) | Acute care | Death | Mortality/survival | Mortality | Mortality (30-day) | Clinician-reported |
|  |  |  |  |  |  |  | Adverse events | Adverse events/effects | Complications | Complications (30-day) | Clinician-reported |
|  |  |  |  |  |  |  | Resource use | Hospital | Readmission | 30-day readmission | Clinician-reported |
|  |  |  |  |  |  |  | Physiological/clinical | General outcomes | Frailty | Frailty status | Clinician-reported |
| 38. | Priyadharshan, et al. [148] | Cross-sectional study | 18F, 88M  (48.5 ± 15) | Diabetes: 49  PVD: 12  Trauma: 44  Hansen disease: 1  (2.8 ± 1.4) | TTA: 71  TFA: 18  (NA) | Rehabilitation | Life impact | Global QOL | QOL | World Health Organization Quality of life Bref score | Patient-reported |
|  |  |  |  |  |  |  | Life impact | Global QOL | Prosthesis-related QOL | Prostheses-specific Trinity amputation and prosthesis experience scales-revised questionnaire | Patient-reported |
| 39. | Torbjörnsson, et al. [121] | RCS | No re-amputation: 116F, 122M  (78, 76 to 79)  Re-amputation: 22F, 28M  (75, 72 to 78) | No re-amputation: Chronic limb-threatening ischemia induced:  Non-healing ulcer: 77  Ischemic pain: 36  Combination of both: 123  (NA)  Re-amputation: Chronic limb-threatening ischemia induced:  Non-healing ulcer: 11  Ischemic pain: 14  Combination of both: 23  (NA) | No re-amputation: TTA: 163  TKA: 40  TFA: 35  (UL: 191, BL: 47)  Re-amputation: TTA: 37  TKA: 8  TFA: 5  (UL: 31, BL: 19) | Acute care | Adverse events | Adverse events/effects | Re-amputation/revision | Incidence of ipsilateral re-amputation | Clinician-reported |
|  |  |  |  |  |  |  | Death | Mortality/survival | Mortality | All-cause mortality | Clinician-reported |
| 40. | Hitzig, et al. [139] | Cross-sectional study | 45F, 186M  (63.4, 31 to 93) | Dysvascular: 231  (3.3, 0.5 to 27) | TTA: 171  TFA: 24  (UL: 195, BL: 36) | Community | Life impact | Global QOL | QOL | SF-36  Life satisfaction-11 questionnaire | Patient-reported |
|  |  |  |  |  |  |  | Life impact | Physical functioning | Mobility | Special interest group in amputee medicine questionnaire | Patient-reported |
|  |  |  |  |  |  |  | Physiological/clinical | General outcomes | Comorbidities | Dysvascular condition scale | Patient-reported |
|  |  |  |  |  |  |  | Life impact | Emotional functioning/well being | Loneliness | University of California, Los Angeles loneliness scale | Patient-reported |
|  |  |  |  |  |  |  | Life impact | Social functioning | Social relationship | Social network index | Patient-reported |
|  |  |  |  |  |  |  | Resource use | Societal/carer burden | Social support | Interpersonal support evaluation list–short-form scale | Patient-reported |
|  |  |  |  |  |  |  | Life impact | Social functioning | Social participation | Reintegration to normal living index | Patient-reported |
|  |  |  |  |  |  |  | Life impact | Emotional functioning/wellbeing | Self-efficacy | Washington self-efficacy scale | Patient-reported |
| 41. | Vasilchenko [124] | RCS | 678F, 1290M  (67 ± 0.2) | Atherosclerosis/thromboangiitis: 1978  (NA) | TTA/TFA: 1978  (NA) | Rehabilitation | Life impact | Physical functioning | Prosthesis use | Prosthesis use (rate and timing) | Clinician-reported |
|  |  |  |  |  |  |  | Life impact | Delivery of care | Time to receive services | Time interval to delivery of prosthetic and orthotic care services | Clinician-reported |
| 42. | Ratliff, et al. [111] | RCS | 4777F, 10804M  (63.9 ± 12.5) | Diabetes: 15581  (NA) | TMT: 4340  FA/ankle: 670  TTA: 6134  TKA/TFA: 4437  (UL: 15581) | Acute care | Resource use | Hospital | Readmission | Readmission (within 30 days of index discharge)  Readmission (within 90 days of index discharge)  Readmission (within 180 days of index discharge)  Cause specific re-admission | Clinician-reported |
|  |  |  |  |  |  |  | Adverse events | Adverse events/effects | Re-amputation/revision | Re-amputation (within 30 days of index discharge)  Re-amputation (within 90 days of index discharge)  Re-amputation (within 180 days of index discharge)  Proximal major ipsilateral re-amputation | Clinician-reported |
|  |  |  |  |  |  |  | Death | Mortality/survival | Mortality | Mortality | Clinician-reported |
| 43. | Camazine, et al. [54] | RCS | 874F, 1521M  Unknown: 4  (65, 55 to 76) | PVD: 2399  (NA) | TTA: 1602  TFA: 797  (NA) | Acute care | Resource use | Hospital | Duration of stay | Length of hospital stay | Clinician-reported |
|  |  |  |  |  |  |  | Resource use | Hospital | Readmission | Readmission (30-day) | Clinician-reported |
|  |  |  |  |  |  |  | Adverse events | Adverse events/effects | Complications | Complications (renal failure, stroke, infection, cardiac/myocardial infarction) | Clinician-reported |
|  |  |  |  |  |  |  | Resource use | Need for further intervention | Use of medication | Opioid utilization (Morphine milligram equivalent per day) | Clinician-reported |
| 44. | Wurdeman, et al. [129] | RCS | Microprocessor knee: 40F, 97M  (61.9. 55.5 to 68.2)  Non-microprocessor knee: 205F, 539M  (62.4, 55.5 to 69.3) | Microprocessor knee:  Diabetes/vascular disease: 137  (6.2, 1 to 8.8)  Non-microprocessor knee: 744  Diabetes/vascular disease:  (5.9, 1.8 to 9.2) | Microprocessor knee:  TFA: 137  (UL: 137)  Non-microprocessor knee:  TFA: 744  (UL: 744) | Rehabilitation | Physiological/clinical | Injury and poisoning outcomes | Falls | Fall incidence in past 6 months (injurious/general falls) | Patient-reported |
| 45. | Çamur, et al. [171] | PCS | Amputee: Major: 16F, 62M  (73.7)  Minor: 19F, 32M  (72.1)  Caregivers: 108F, 31M  (56.8 ± 9.1) | Diabetic foot ulcer: 139  (NA) | Heel sparing: 51  TFA: 88  (UL: 139) | Community | Resource use | Societal/carer burden | Carer burden | Zarit Burden Interview | Patient-reported |
| 46. | Park, et al. [108] | RCS | 21F, 25M  (62.9 ± 11.6) | Diabetic end stage renal disease: 41  (NA) | TTA: 36  TFA: 10  (NA) | Acute care | Adverse events | Adverse events/effects | Complications | Complications (no, minor, major) | Clinician-reported |
| 47. | Franchin, et al. [173] | PCS | 98F, 188M  (79, 69 to 83) | CLI: 235  ALI: 46  Infection not related to peripheral arterial occlusive disease: 29 | TTA: 53  TFA: 257  (UL: 286, BL: 24) | Acute care | Death | Mortality/survival | Mortality | Early mortality (<30-day) | Clinician-reported |
|  |  |  |  |  |  |  | Adverse events | Adverse events/effects | Complications | Clavien-Dindo severity grading system (post-operative complications) | Clinician-reported |
|  |  |  |  |  |  |  | Physiological/clinical | General outcomes | Frailty | Modified frailty index | Clinician-reported |
|  |  |  |  |  |  |  | Adverse events | Adverse events/effects | Re-amputation/revision | Freedom from amputation stump revision/failure | Clinician-reported |
| 48. | Pereira, et al. [147] | Cross-sectional study | 23F, 50M  (63 ± 16.3) | Diabetes/PVD: 47  Other: 16  (3.7 ± 4.2) | TTA: 57  TFA: 6  (NA) | Rehabilitation | Life impact | Emotional functioning/well being | Coping ability | Brief COPE scale to assess dispositional coping strategies | Patient-reported |
|  |  |  |  |  |  |  | Life impact | Global QOL | QOL | Satisfaction with life scale | Patient-reported |
|  |  |  |  |  |  |  | Life impact | Physical functioning | Prosthesis use | Prosthesis use | Patient-reported |
|  |  |  |  |  |  |  | Life impact | Physical functioning | Locomotion ability | Walking ability (no ability, at home/with barrier, no limitation) | Patient-reported |
|  |  |  |  |  |  |  | Physiological/ clinical | Nervous system outcomes | Residual limb pain | Residual limb pain | Patient-reported |
|  |  |  |  |  |  |  | Physiological/ clinical | Nervous system outcomes | Phantom limb pain | Phantom limb pain | Patient-reported |
| 49. | Huseynova, et al. [80] | RCS | 3355F, 6604M  (NA) | Malignancy/trauma but related to diabetes mellitus and/or ischemia, gangrene or infection: 9561  Other: 402  (NA) | Major: 5816  Minor: 4142  (UL: 9959) | Acute care | Adverse events | Adverse events/effects | Re-amputation/revision | Incidence of contralateral major lower limb amputation 30-days after index procedure  Risk of contralateral minor lower limb amputation | Clinician-reported |
|  |  |  |  |  |  |  | Death | Mortality/survival | Mortality | Mortality prior to contralateral major/minor lower limb amputation | Clinician-reported |
| 50. | Baran, et al. [198] | Case-control study | Amputee: 2M  (64.5)  Healthy: 2  (26) | Diabetes: 1  Trauma: 1  (NA) | TTA: 2  (NA) | Acute care | Physiological/clinical | Vascular outcome | Diagnostic test | Post-occlusive reactive hyperemia response following applied physical stress | Clinician-reported |
|  |  |  |  |  |  |  | Physiological/clinical | Vascular outcome | Radiology | Optical coherence tomography-based microangiography | Clinician-reported |
| 51. | Peters, et al. [179] | PCS | 18F, 31M  (82, 75 to 84) | CLI: 49  (NA) | TTA: 30  TKA: 4  TFA: 15  Minor: 13  (NA) | Acute care, community | Life impact | Global QOL | QOL | World Health Organization Quality of Life Bref score  12-item short form health survey | Patient-reported |
|  |  |  |  |  |  |  | Physiological/ clinical | Psychiatric outcomes | Depression | Center for epidemiological studies depression scale | Patient-reported |
|  |  |  |  |  |  |  | Death | Mortality/survival | Mortality | Mortality (30-day)  Mortality (1-year)  Mortality (2-year) | Clinician-reported |
|  |  |  |  |  |  |  | Life impact | Physical functioning | Prosthesis use | Prosthesis use | Patient-reported |
|  |  |  |  |  |  |  | Resource use | Hospital | Discharge | Discharge after major amputation (home, nursing facility, inpatient rehabilitation, deceased before discharge) | Clinician-reported |
|  |  |  |  |  |  |  | Life impact | Delivery of care | Feasibility | Time to conduct questionnaires | Clinician-reported |
| 52. | Koivunen, et al. [85] | RCS | 437F, 454M  (77.4 ± 1.9) | Diabetes mellitus/PAD: 891  (118 amputated urgently with severe infection)  (NA) | TTA: 177  TFA: 714  (NA) | Acute care | Resource use | Need for further intervention | Use of medication | Use of medications (antithrombotic, anticoagulant, statins) | Clinician-reported |
|  |  |  |  |  |  |  | Adverse events | Adverse events/effects | Re-amputation/revision | Re-amputation  Revision surgery | Clinician-reported |
|  |  |  |  |  |  |  | Death | Mortality/survival | Survival | Survival (1-year)  Survival (3-year)  Survival (5-year) | Clinician-reported |
| 53. | Aziz, et al. [51] | RCS | 804F, 1361M  (73 ± 11.3) | Diabetes: 2165  (NA) | TTA: 1223  TKA: 21  TFA: 921  (NA) | Acute care | Death | Mortality/survival | Mortality | All-cause mortality (30-day)  All-cause mortality (90-day)  All-cause mortality (1-year)  All-cause mortality (5-year) | Clinician-reported |
|  |  |  |  |  |  |  | Physiological/clinical | General outcomes | Comorbidities | Age-adjusted Charlson comorbidity index | Clinician-reported |
|  |  |  |  |  |  |  | Physiological/clinical | General outcome | Amputation | Age-standardized rates of major lower limb amputation | Clinician-reported |
| 54. | Imanawanto, et al. [160] | Case study | 1F  (44) | Arterial stenosis: 1  (NA) | TTA: 1  (UL: 1) | Rehabilitation | Physiological/ clinical | Skin and subcutaneous tissue outcomes | Stump wound healing | Residual limb wound healing | Clinician-reported |
|  |  |  |  |  |  |  | Life impact | Physical functioning | ROM | Knee joint range of motion | Performance-related |
|  |  |  |  |  |  |  | Life impact | Physical functioning | Prosthesis fit | Prosthetic fitting | Patient-reported |
|  |  |  |  |  |  |  | Life impact | Physical functioning | Gait: spatiotemporal walking | 6MWT | Performance-related |
|  |  |  |  |  |  |  | Physiological/ clinical | Respiratory, thoracic and mediastinal outcomes | Cardiopulmonary fitness | VO_2_ max | Performance-related |
|  |  |  |  |  |  |  | Physiological/clinical | Cardiovascular outcomes | Diagnostic test | Ankle brachial index | Clinician-reported |
|  |  |  |  |  |  |  | Physiological/clinical | Vascular outcomes | Radiology | Computed tomography for angiography | Clinician-reported |
|  |  |  |  |  |  |  | Life impact | Physical functioning | Functional independence | Barthel index | Clinician-reported |
|  |  |  |  |  |  |  | Physiological/clinical | Vascular outcomes | Diagnostic test | Doppler ultrasound (popliteal artery velocity) | Clinician-reported |
|  |  |  |  |  |  |  | Physiological/clinical | Blood and lymphatic system outcomes | Diagnostic test | Oxygen saturation | Clinician-reported |
|  |  |  |  |  |  |  | Physiological/clinical | Nervous system outcomes | General pain | Wong-Baker FACES score | Patient-reported |
| 55. | Zhang, et al. [132] | RCS | 309F, 601M  (NA) | Diabetes: 910  (NA) | TTA: 487  TFA: 423  (NA) | Acute care | Resource use | Hospital | Readmission | Readmission (30-day) | Clinician-reported |
|  |  |  |  |  |  |  | Adverse events | Adverse events/effects | Re-amputation/revision | Re-amputation (1-year) | Clinician-reported |
|  |  |  |  |  |  |  | Adverse events | Adverse events/effects | Complications | Stump revisions/complications | Clinician-reported |
|  |  |  |  |  |  |  | Resource use | Economic | Socioeconomic disadvantage | Area deprivation index | Clinician-reported |
| 56. | Chahrour, et al. [55] | RCS | 7651F, 13798M  (NA) | Diabetes: 21449  (NA) | TTA: 13608  TFA: 7841  (NA) | Acute care | Death | Mortality/survival | Mortality | Mortality (30-day) | Clinician-reported |
|  |  |  |  |  |  |  | Physiological/clinical | Infection and infestation outcomes | Infection | Surgical site infection (superficial, deep, organ space) within 30-days of surgery | Clinician-reported |
| 57. | Pande, et al. [105] | RCS | 25F, 45M  (62.7, 35 to 88) | Dysvascular: 70  (NA) | TTA: 53  TFA: 17  (NA) | Rehabilitation | Resource use | Hospital | Duration of stay | Length of inpatient rehabilitation stay | Clinician-reported |
|  |  |  |  |  |  |  | Death | Mortality/survival | Survival | Survival rate (1-year) | Clinician-reported |
| 58. | Albright-Trainer, et al. [184] | RCT | 1F, 15M  (72 ± 18) | PVD: 7  PAD: 3  Pain: 2  Diabetes mellitus: 4  (NA) | TTA: 7  TFA: 9  (NA) | Rehabilitation | Physiological/ clinical | Nervous system outcomes | Residual limb pain | Brief pain inventory short form (residual limb pain) | Patient reported |
|  |  |  |  |  |  |  | Physiological/ clinical | Nervous system outcomes | Phantom limb pain | Brief pain inventory short form (phantom limb pain) | Patient reported |
|  |  |  |  |  |  |  | Life impact | Physical functioning | Functional independence | Functional independence measure | Clinician-reported |
|  |  |  |  |  |  |  | Life impact | Delivery of care | Global impression of change | Patient global impression of change | Patient reported |
|  |  |  |  |  |  |  | Physiological/ clinical | Nervous system outcomes | Pain catastrophizing | Pain catastrophizing scale | Patient reported |
|  |  |  |  |  |  |  | Physiological/ clinical | Nervous system outcomes | Pain interference | Pain interference score | Patient reported |
|  |  |  |  |  |  |  | Life impact | Physical functioning | Impact of disease/condition on ADL | Pain disability index | Patient reported |
|  |  |  |  |  |  |  | Resource use | Need for further intervention | Use of medication | Opioid consumption (oral morphine equivalent) | Clinician-reported |
|  |  |  |  |  |  |  | Resource use | Hospital | Duration of stay | Length of hospital stay | Clinician-reported |
|  |  |  |  |  |  |  | Resource use | Hospital | Readmission | Readmission rate (30-day) | Clinician-reported |
| 59. | MacKay, et al. [191] | Mixed method approach | 12F, 23M  (62, 32 to 86) | Diabetes/PVD/acute ischemia/embolism: 35  (2.6, 0.5 to 7) | TTA: 23  TFA; 5  (UL: 28, BL: 7) | Community | Life impact | Global QOL | QOL | Semi-structured interviews concerning perceptions and experiences of living with amputation on altered mobility, social disruption, psychological impact, socioeconomic factors, accessibility, social support | Patient-reported |
| 60. | England, et al. [67] | RCS | Dysvascular: 2068F, 5405M  (62, 54 to 70.3)  Trauma: 718F, 2892M  (55, 43 to 65)  Congenital: 190F, 212M  (42.5, 31 to 56.1)  Cancer: 217F, 293M  (53.8, 36 to 66.4) | Dysvascular: 7473  Trauma: 3610  Congenital: 402  Cancer: 510  (NA) | Dysvascular: TTA: 5988  TFA: 1485  (UL: 7473)  Trauma: TTA: 2559  TFA: 1051  (UL: 3610)  Congenital: TTA: 280  TFA: 122  (UL: 402)  Cancer: TTA: 251  TFA: 259  (UL: 510) | Rehabilitation | Life impact | Physical functioning | Prosthesis-related mobility | Prosthetic limb user survey of mobility-short form | Patient-reported |
| 61. | Nickinson, et al. [96] | RCS | 1176F, 2084M  (72, 61 to 81) | Chronic limb threatening ischemia/diabetes: 3260  (NA) | Major: 3260 (level not reported)  (NA) | Acute care | Physiological/clinical | Cardiovascular outcome | Diagnostic test | Cardiovascular assessment prior to amputation (7 to 90 days, >91 days) | Clinician-reported |
|  |  |  |  |  |  |  | Resource use | Need for further intervention | Specialist consultation | Primary care consultation prior to amputation (7 to 30, 31 to 90, 91 to 180, 181 to 365, >365 days) | Clinician-reported |
| 62. | Kuhlmann, et al. [86] | RCS | 4459F, 5039M  (NA) | Diabetes mellitus: 649  PVD: 3343  Diabetes mellitus and PVD: 4519  Other: 987  (NA) | TKA/TFA: 9498  (NA) | Acute care | Resource use | Economic | Cost | Cost-effectiveness  Budget-impact analysis of microprocessor vs. non-microprocessor controlled prosthetic knee  Costs in Euros (prosthesis costs, fall-related medical costs, total costs), quality adjusted life years per patient | Clinician-reported |
|  |  |  |  |  |  |  | Physiological/clinical | Injury and poisoning outcomes | Falls | Falls per 1000 person years (fatal, non-fatal) | Clinician-reported |
|  |  |  |  |  |  |  | Resource use | Hospital | Admission | Hospitalizations per 1000 person years | Clinician-reported |
|  |  |  |  |  |  |  | Resource use | Need for further intervention | Specialist consultation | Outpatient treatment per 1000 person years | Clinician-reported |
|  |  |  |  |  |  |  | Death | Mortality/survival | Survival | Life years per patient | Clinician-reported |
| 63. | Wurdeman, et al. [131] | RCS | Microprocessor ankle-foot: 4F, 24M  (57.1 ± 13)  Shank-foot system with vertical loading pylon: 11F, 144M  (57.7 ± 12.1)  Flex-walk system: 86F, 256M  (58.8 ± 10.4)  Flex-foot system: 34F, 89M  (58.6 ± 11.8)  Hydraulic ankle-foot system: 15F, 75M  (61.1 ± 10.2) | Microprocessor ankle-foot: Diabetes and/or vascular disease: 28  (10.7 ± 13.6)  Shank-foot system with vertical loading pylon:  Diabetes and/or vascular disease: 155  (8.1 ± 11)  Flex-walk system:  Diabetes and/or vascular disease: 342  (7.3 ± 10.1)  Flex-foot system:  Diabetes and/or vascular disease: 123  (8.6 ± 10.9)  Hydraulic ankle-foot system:  Diabetes and/or vascular disease: 90  (7.6 ± 11.7) | Microprocessor ankle-foot:  TTA: 14  TFA: 7  (UL: 21, BL: 7)  Shank-foot system with vertical loading pylon:  TTA: 121  TFA: 16  (UL: 137, BL: 18)  Flex-walk system:  TTA: 241  TFA: 67  (UL: 308, BL: 34)  Flex-foot system:  TTA: 80  TFA: 31  (UL: 121, BL: 12)  Hydraulic ankle-foot system:  TTA: 66  TFA: 7  (UL: 73, BL: 17) | Rehabilitation | Life impact | Physical functioning | Prosthesis-related mobility | Prosthetic limb users survey of mobility 12-item questionnaire | Patient-reported |
| 64. | Alothman, et al. [49] | RCS | 558F, 350M  (62.6 ± 12.1) | Diabetes: 908  (NA) | Toe: 216  FA: 59  Leg: 132  TTA: 321  TFA: 149  Hip: 5  (NA) | Acute care | Physiological/clinical | General outcomes | Diagnostic test | Frequency of diagnosis of single/multiple risk factors (PVD, neuropathy, and foot ulcer) | Clinician-reported |
| 65. | Shaw, et al. [151] | Cross-sectional study | 2F, 8M  (60 ± 8.4) | Dysvascular: 10  (NA) | TTA: 10  (NA) | Rehabilitation | Life impact | Physical functioning | ROM | Peak hip extension ROM by goniometer | Clinician-reported |
|  |  |  |  |  |  |  | Life impact | Physical functioning | Flexibility | Modified Thomas test | Clinician-reported |
| 66. | Wurdeman, et al. [130] | RCS | 146F, 363M  (56.4 ± 14.6) | Vascular disease/diabetes: 202  Injury/trauma: 163  Infection (without diabetes): 48  Cancer: 23  Congenital: 22  Other: 30  Unknown: 21  (NA) | Partial FA: 1  TTA/Symes: 350  TFA/TKA: 350  (UL: 456, BL: 53) | Rehabilitation | Life impact | Physical functioning | Prosthesis-related mobility | Prosthetic limb users survey of mobility | Patient-reported |
|  |  |  |  |  |  |  | Life impact | Global QOL | Prosthesis-related QOL | PEQ well-being subscale | Patient-reported |
| 67. | Morisaki, et al. [92] | RCS | 39F, 67M  (77.3 ± 11.2) | PAD: 106  (NA) | TTA: 39  TFA: 79  (NA) | Acute care | Death | Mortality/survival | Mortality | Mortality (30-day) | Clinician-reported |
|  |  |  |  |  |  |  | Adverse events | Adverse events/effects | Complications | Risk factors for wound complications (surgical site infection, wound dehiscence) | Clinician-reported |
|  |  |  |  |  |  |  | Death | Mortality/survival | Survival | Overall survival (1-year)  Overall survival (2-year) | Clinician-reported |
| 68. | Wang, et al. [125] | RCS | 12F, 16M  (76.1 ± 11.8) | Arteriosclerosis obliterans: 14  Arteriosclerosis obliterans with acute thrombosis: 8  Acute embolism: 6  (NA) | TTA: 7  TFA: 21  (NA) | Acute care | Resource use | Hospital | Duration of operation | Operation time | Clinician-reported |
|  |  |  |  |  |  |  | Resource use | Need for further intervention | Use of anesthesia | Conversion to general anesthesia  American society of anesthesiologist’s classification | Clinician-reported |
|  |  |  |  |  |  |  | Resource use | Need for further intervention | Emergency procedures | Emergency procedures | Clinician-reported |
|  |  |  |  |  |  |  | Resource use | Hospital | Operative outcome | Intra-operative events (transfusion, hypertension, vasopressor use, hypoxia, tachycardia) | Clinician-reported |
|  |  |  |  |  |  |  | Resource use | Hospital | Admission | Post-operative ICU admission | Clinician-reported |
|  |  |  |  |  |  |  | Adverse events | Adverse events/effects | Adverse events | New York heart association classification | Clinician-reported |
|  |  |  |  |  |  |  | Resource use | Need for further intervention | Use of medication | Postoperative opioid consumption  Additional analgesia and sedation | Clinician-reported |
|  |  |  |  |  |  |  | Resource use | Hospital | Duration of stay | Postoperative length of hospital stay | Clinician-reported |
|  |  |  |  |  |  |  | Adverse events | Adverse events/effects | Complications | Post-operative complications (wound infection, need for surgical revision, phantom limb pain, delirium, acute heart failure, pneumonia, acute cardiovascular disease) | Clinician-reported |
|  |  |  |  |  |  |  | Death | Mortality/survival | Mortality | Mortality (48-hour)  Mortality (30-day)  Mortality (1-year) | Clinician-reported |
| 69. | Roepke, et al. [181] | PCS | 1F, 67M  (62.6 ± 7.2) | Diabetes/ PAD: 68  (NA) | TMT: 17  TTA: 36  TFA: 15  (NA) | Community | Physiological/ clinical | Psychiatric outcomes | Depression | PHQ-9 | Patient-reported |
|  |  |  |  |  |  |  | Resource use | Societal/carer burden | Social support | Modified social support survey | Patient-reported |
|  |  |  |  |  |  |  | Life impact | Physical functioning | Locomotion ability | Locomotor capability index-5 | Patient-reported |
|  |  |  |  |  |  |  | Physiological/ clinical | Psychiatric outcomes | Mental health | Short portable mental status questionnaire | Patient-reported |
|  |  |  |  |  |  |  | Life impact | Global QOL | QOL | SF-36 | Patient-reported |
| 70. | D'Cruz, et al. [63] | RCS | 211 (?F, ?M)  (67.8 ± 16.5) | PAD related tissue loss or sepsis: 211  (NA) | TTA: 133  TFA: 79  (UL: 202, BL: 9) | Acute care | Resource use | Hospital | Readmission | Re-admission rate  Unexpected re-admission (30-day) | Clinician-reported |
|  |  |  |  |  |  |  | Death | Mortality/survival | Mortality | Mortality (30-day)  Mortality (6-month)  Mortality (30-day) | Clinician-reported |
|  |  |  |  |  |  |  | Adverse events | Adverse events/effects | Complications | Perioperative complications | Clinician-reported |
|  |  |  |  |  |  |  | Physiological/clinical | General outcomes | Frailty | Modified frailty index | Clinician-reported |
| 71. | Kuy, et al. [164] | Case study | 1M  (67) | Diabetic foot infection: 1  (3)  Osteomyelitis: 1  (0.08) | TTA: 1  Toe: 1  (BL: 1) | Acute care | Physiological/clinical | Endocrine outcomes | Laboratory parameters | Blood glucose level | Clinician-reported |
|  |  |  |  |  |  |  | Physiological/clinical | Immune system outcomes | Laboratory parameters | White blood count | Clinician-reported |
|  |  |  |  |  |  |  | Physiological/clinical | Musculoskeletal and connective tissue outcomes | Radiology | X-ray | Clinician-reported |
|  |  |  |  |  |  |  | Physiological/clinical | General outcomes | Physical examination | Physical examination | Clinician-reported |
|  |  |  |  |  |  |  | Resource use | Need for further intervention | Use of medication | Use of antibiotics (vancomycin, piperaciilin/ tazobactum) | Clinician-reported |
|  |  |  |  |  |  |  | Physiological/clinical | Infection and infestation outcomes | Microbial culture | Microbial cultures | Clinician-reported |
| 72. | Vanicek, et al. [189] | RCT | 8F, 47M  (68.8 ± 9.6) | Diabetes: 25  PVD: 20  Blood clot: 5  Deep vein thrombosis: 1  Aneurysm: 1  Diabetic neuropathy: 3  Another neuropathy: 1  Cancer: 2  Other: 11  (NA) | TTA: 55  (UL: 55) | Community | Life impact | Physical functioning | Locomotion ability | Locomotor capabilities index-5 | Patient-reported |
|  |  |  |  |  |  |  | Physiological/ clinical | Nervous system outcomes | General pain | PROMIS (3a, 8a) short form for pain | Patient-reported |
|  |  |  |  |  |  |  | Life impact | Physical functioning | Prosthesis use | Houghton scale | Patient-reported |
|  |  |  |  |  |  |  | Life impact | Physical functioning | Mobility | TUG  Timed up and down stairs test | Performance-related |
|  |  |  |  |  |  |  | Life impact | Physical functioning | Balance | BBS | Performance-related |
|  |  |  |  |  |  |  | Life impact | Physical functioning | Gait: spatiotemporal walking | Daily step count by activity monitor  2MWT  Time spent walking by activity monitor | Performance-related |
|  |  |  |  |  |  |  | Life impact | Delivery of care | Feasibility | Recruitment  Retention rate  Consent rate  Completion of questionnaire rate | Clinician-reported |
|  |  |  |  |  |  |  | Adverse events | Adverse events/effects | Adverse events | Adverse events (related to prosthesis, serious adverse events) | Patient-reported |
|  |  |  |  |  |  |  | Life impact | Global QOL | QOL | EQ-5D-5L | Patient-reported |
|  |  |  |  |  |  |  | Resource use | Economic | Cost | Bespoke health resource use questionnaire | Patient-reported |
| 73. | O’Banion, et al. [101] | RCS | 35F, 95M  (57 ± 13) | Dysvascular: 130  (NA) | TTA: 117  TFA: 13  (NA) | Rehabilitation | Resource use | Hospital | Discharge | Discharge to acute rehabilitation facility, home, skilled nursing facility | Clinician-reported |
|  |  |  |  |  |  |  | Life impact | Physical functioning | Mobility | Post-operative time to mobilization (days) | Clinician-reported |
|  |  |  |  |  |  |  | Resource use | Need for further intervention | Rehabilitation | Time of inpatient physical therapy consultation (≤1 day vs >1 day) post-operatively (days) | Clinician-reported |
|  |  |  |  |  |  |  | Resource use | Hospital | Duration of stay | Post-operative hospital length of stay (days) | Clinician-reported |
| 74. | Niskakangas, et al. [99] | RCS | 189F, 245M (76, 67 to 83) | PAD: 429  Other: 5  (NA) | TTA: 286  TFA: 249  (NA) | Acute care | Resource use | Need for further intervention | Use of anesthesia | Use of continuous epidural (% patients) in patients receiving spinal, general anesthesia during 4 post-operative days | Clinician-reported |
|  |  |  |  |  |  |  | Resource use | Need for further intervention | Use of medication | Use of oxycodone in patients receiving spinal, general anesthesia with TTA, TFA | Clinician-reported |
|  |  |  |  |  |  |  | Adverse events | Adverse events/effects | Complications | Surgical complications (need for revision, need for conversion of amputation, wound infection, stump necrosis, neuralgia), medical complications (acute myocardial infarction, pneumonia, sepsis) | Clinician-reported |
|  |  |  |  |  |  |  | Death | Mortality/survival | Mortality | Mortality before (28 days, 1 year)  Mortality following intensive care unit stay (28 days, 1 year) | Clinician-reported |
|  |  |  |  |  |  |  | Resource use | Hospital | Duration of stay | Length of hospital stay (days) | Clinician-reported |
|  |  |  |  |  |  |  | Resource use | Hospital | Admission | Need for ICU stay | Clinician-reported |
|  |  |  |  |  |  |  | Resource use | Hospital | Duration of stay | Length of ICU stay (days) | Clinician-reported |
| 75. | Cheun, et al. [57] | RCS | Staged amputation: 19F, 41M  (55 ± 10)  Primary amputation: 17F, 38M  (61 ± 11) | Diabetes with foot gangrene: 111  (NA) | Ankle: 50  TTA: 50  TKA: 9  TFA: 7  (NA) | Acute care | Adverse events | Adverse events/effects | Re-amputation/revision | Unplanned ipsilateral re-amputation rate (30-day) | Clinician-reported |
|  |  |  |  |  |  |  | Adverse events | Adverse events/effects | Complications | Stump complication (wound dehiscence, necrosis, infection, intractable pain necessitating revision)  Major adverse cardiovascular event (30-day) | Clinician-reported |
|  |  |  |  |  |  |  | Resource use | Hospital | Readmission | Readmission (30-day) | Clinician-reported |
|  |  |  |  |  |  |  | Death | Mortality/survival | Mortality | Mortality (30-day) | Clinician-reported |
|  |  |  |  |  |  |  | Resource use | Hospital | Discharge | Discharge to home | Clinician-reported |
|  |  |  |  |  |  |  | Resource use | Hospital | Duration of stay | Length of hospital stay | Clinician-reported |
|  |  |  |  |  |  |  | Physiological/clinical | Blood and lymphatic system outcomes | Blood transfusion | Blood product transfusion | Clinician-reported |
|  |  |  |  |  |  |  | Resource use | Hospital | Admission | Use of ICU | Clinician-reported |
| 76. | Vascular Quality, et al. [123] | RCS | 2379F, 4321M  (65.5, 65.2 to 65.8) | Ischemic tissue loss: 3285  Uncontrolled infection: 2121  Acute ischemia: 553  Ischemic rest pain: 441  Neuropathic tissue loss: 156  Other: 165  (NA) | TTA: 3855  TFA: 2856  (UL: 6701) | Acute care | Death | Mortality/survival | Mortality | Mortality (30-day) | Clinician-reported |
|  |  |  |  |  |  |  | Resource use | Hospital | Operative outcome | Return to operating room | Clinician-reported |
|  |  |  |  |  |  |  | Adverse events | Adverse events/effects | Complications | Post-operative myocardial infarction | Clinician-reported |
|  |  |  |  |  |  |  | Physiological/clinical | Infection and infestation outcomes | Infection | Post-operative skin infection | Clinician-reported |
| 77. | Satam, et al. [113] | RCS | Not candidate for percutaneous deep venous arterialization: 17F, 36M  (69.6 ± 10.4)  Candidate not for percutaneous deep venous arterialization: 6F, 9M  (65.8 ± 16.3) | CLTI: 68  (NA) | Not candidate for percutaneous deep venous arterialization: Major amputation: 53 (level not reported)  (4.1 ± 3.4)  Candidate not for percutaneous deep venous arterialization: Major amputation: 15 (level not reported)  (3.9 ± 3.4) | Acute care | Life impact | Delivery of care | Time to receive services | Time to receive angiography (days) | Clinician-reported |
|  |  |  |  |  |  |  | Physiological/clinical | Vascular outcome | CLTI classification | Infrainguinal GLASS stage (I to III) | Clinician-reported |
|  |  |  |  |  |  |  | Physiological/clinical | Vascular outcome | Radiology | Angiogram scores (Grade 1 to 3) for femoropopliteal, infrapopliteal, and inframalleolar grades | Clinician-reported |
|  |  |  |  |  |  |  | Death | Mortality/survival | Mortality | Long term mortality | Clinician-reported |
|  |  |  |  |  |  |  | Resource use | Need for further intervention | Reoperation | Re-interventions | Clinician-reported |
|  |  |  |  |  |  |  | Adverse events | Adverse events/effects | Re-amputation/revision | Re-amputation | Clinician-reported |
| 78. | Beyaz, et al. [53] | RCS | 171F, 299M  (64.3, 20 to 101) | Diabetic foot problems: 470  (NA) | TTA: 470  (UL: 414, BL: 56) | Acute care | Death | Mortality/survival | Survival | Overall survival probability | Clinician-reported |
|  |  |  |  |  |  |  | Physiological/clinical | Endocrine outcomes | Diagnostic test | Hba1c levels | Clinician-reported |
|  |  |  |  |  |  |  | Physiological/clinical | Immune system outcomes | Laboratory parameters | C-reactive protein levels | Clinician-reported |
|  |  |  |  |  |  |  | Physiological/clinical | Blood and lymphatic system outcomes | Laboratory parameters | Erythrocyte sedimentation rate | Clinician-reported |
|  |  |  |  |  |  |  | Physiological/clinical | Renal and urinary outcomes | Laboratory parameters | Blood urea nitrogen  Creatinine levels | Clinician-reported |
|  |  |  |  |  |  |  | Resource use | Need for further intervention | Use of medication | Period of oral antidiabetic drug and insulin use (years) | Clinician-reported |
|  |  |  |  |  |  |  | Adverse events | Adverse events/effects | Re-amputation/revision | Need for stump revision | Clinician-reported |
| 79. | Debeljak, et al. [66] | RCS | 5F, 35M  (70, 44 to 94) | Diabetes mellitus/PVD/other: 40  (NA) | TTA: 19  TFA: 23  Hip disarticulation: 1  (UL: 34, BL: 6) | Community | Life impact | Perceived health status | Health status | Supporting Active Ageing through Multimodal coaching questionnaire (demographics, use/ownership of information and communication technologies, satisfaction with social life and co-morbidities) | Patient-reported |
|  |  |  |  |  |  |  | Life impact | Delivery of care | Feasibility | Response rate to participation | Clinician-reported |
| 80. | Nijmeijer, et al. [98] | RCS | 57F, 81M  (74.2 ± 12.7) | Acute/chronic vascular disease: 133  Infection: 5  (NA) | TKA: 138  (UL: 123, BL: 15) | Acute care | Resource use | Hospital | Discharge | Discharge (nursing facility, rehabilitation center, rehabilitation day care, home) | Clinician-reported |
|  |  |  |  |  |  |  | Death | Mortality/survival | Mortality | In-hospital mortality | Clinician-reported |
|  |  |  |  |  |  |  | Resource use | Need for further intervention | Mobility aid | Discharged TKA patients provided/not provided with prosthesis | Patient-reported |
|  |  |  |  |  |  |  | Physiological/ clinical | Skin and subcutaneous tissue outcomes | Stump wound healing | Postoperative wound healing (primary, delayed, no healing) | Clinician-reported |
|  |  |  |  |  |  |  | Resource use | Need for further intervention | Wound management | Postoperative stump dressing (elastic bandage, Plaster of Paris) | Clinician-reported |
|  |  |  |  |  |  |  | Life impact | Physical functioning | Mobility | Special Interest Group Amputation Medicine- Workgroup Amputation and Prosthetics mobility scale | Patient-reported |
|  |  |  |  |  |  |  | Life impact | Physical functioning | Prosthesis use | Pre-operative intention to use prosthesis | Patient-reported |
|  |  |  |  |  |  |  | Death | Mortality/survival | Survival | Survival (1-month)  Survival (6-month)  Survival (1-year) | Clinician-reported |
|  |  |  |  |  |  |  | Adverse events | Adverse events/effects | Re-amputation/revision | Re-amputation | Clinician-reported |
| 81. | Madsen, et al. [176] | PCS | Baseline: 15F, 45M  (69 ± 11)  Non-participants: 21F, 24M  (77 ± 10) | Baseline: Diabetes: 24  PAD: 35  Trauma or cancer: 2  Non-participants: Diabetes: 16  PAD: 28  Trauma or cancer: 0  (NA) | Baseline: TTA: 31  TKA: 3  TFA: 26  (UL: 51, BL: 9)  Non-participants: Diabetes: TTA: 9  TKA: 99  TFA: 27  (UL: 39, BL: 6) | Rehabilitation | Resource use | Hospital | Discharge | Discharge to independent living | Clinician-reported |
|  |  |  |  |  |  |  | Life impact | Physical functioning | Prosthesis use | Assessed suitability for prosthesis at discharge | Clinician-reported |
|  |  |  |  |  |  |  | Life impact | Delivery of care | Patient preference | Patient plan to have prosthesis at day 21 | Patient-reported |
|  |  |  |  |  |  |  | Resource use | Hospital | Duration of stay | Length of stay at orthopedic department | Clinician-reported |
|  |  |  |  |  |  |  | Death | Mortality/survival | Mortality | Mortality 30-day | Clinician-reported |
|  |  |  |  |  |  |  | Life impact | Physical functioning | Locomotion ability | Locomotor capability index-5 | Patient-reported |
|  |  |  |  |  |  |  | Adverse events | Adverse events/effects | Re-amputation/revision | Re-amputation (30-day) | Clinician-reported |
|  |  |  |  |  |  |  | Life impact | Physical functioning | Functional independence | Barthel index | Clinician-reported |
|  |  |  |  |  |  |  | Resource use | Need for further intervention | Rehabilitation | Frequency of physiotherapy (2 times per week, >3 times per week) | Patient-reported |
|  |  |  |  |  |  |  | Life impact | Delivery of care | Time to receive services | Time to start physiotherapy (days)  Days with no physiotherapy in hospital | Patient-reported |
|  |  |  |  |  |  |  | Resource use | Need for further intervention | Use of anesthesia | American Society of Anesthesiologist score (1 to 5) | Clinician-reported |
|  |  |  |  |  |  |  | Life impact | Physical functioning | Weight bearing during standing | Weight bearing on remaining leg | Patient-reported |
|  |  |  |  |  |  |  | Physiological/ clinical | Nervous system outcomes | Residual limb pain | Stump pain (no/little vs some/much) | Patient-reported |
|  |  |  |  |  |  |  | Physiological/ clinical | Nervous system outcomes | Phantom limb pain | Phantom pain (no/little vs some/much) | Patient-reported |
|  |  |  |  |  |  |  | Physiological/clinical | Injury and poisoning outcomes | Falls | Fall incidents | Patient-reported |
|  |  |  |  |  |  |  | Life impact | Cognitive functioning | Cognitive function | Mini Mental State Examination | Clinician-reported |
|  |  |  |  |  |  |  | Physiological/clinical | Psychiatric outcomes | Delirium | Post-operative confusion episodes | Patient-reported |
|  |  |  |  |  |  |  | Life impact | Personal circumstances | Residential status | Accommodation (independent, wheelchair accessibility, nursing home)  Living alone | Patient-reported |
|  |  |  |  |  |  |  | Resource use | Societal/carer burden | Social support | Daily assistance (homecare, relatives) | Patient-reported |
|  |  |  |  |  |  |  | Resource use | Need for further intervention | Mobility aid | Lacking assistive devices | Patient-reported |
| 82. | Adiyeke, et al. [133] | RCS | 47F, 119M  (66.7 ± 13) | Diabetic foot infection: 166  (NA) | Foot/toe: 41  TTA: 64  TFA: 61  (UL: 166) | Acute care | Resource use | Hospital | Duration of stay | Days of hospitalization | Clinician-reported |
|  |  |  |  |  |  |  | Resource use | Need for further intervention | Reoperation | Number of operations required | Clinician-reported |
|  |  |  |  |  |  |  | Physiological/clinical | Vascular outcomes | Radiology | Computed angiography level (iliac, femoral, popliteal, dorsalis pedis/posterior tibial artery) | Clinician-reported |
|  |  |  |  |  |  |  | Physiological/clinical | Vascular outcomes | Diagnostic test | Doppler ultrasound iliac, femoral, popliteal, dorsalis pedis/posterior tibial artery) | Clinician-reported |
| 83. | Spiera, et al. [120] | RCS | 1601F, 3649  (NA) | Diabetes with gangrene: 5250  (NA) | TTA: 4331  TFA: 919  (NA) | Acute care | Death | Mortality/survival | Mortality | Mortality | Clinician-reported |
|  |  |  |  |  |  |  | Physiological/clinical | Infection and infestation outcomes | Infection | Surgical site infection (superficial, deep, organ space) | Clinician-reported |
|  |  |  |  |  |  |  | Life impact | Physical functioning | Functional independence | Loss of independence | Clinician-reported |
|  |  |  |  |  |  |  | Adverse events | Adverse events/effects | Complications | Complications (wound disruption, pneumonia, reintubation, pulmonary embolism, failure to wean, renal failure, urinary tract infection, cerebrovascular attack, cardiac arrest, myocardial infarction, transfusion, deep vein thrombosis, sepsis, septic shock) | Clinician-reported |
|  |  |  |  |  |  |  | Resource use | Need for further intervention | Reoperation | Re-operation | Clinician-reported |
|  |  |  |  |  |  |  | Resource use | Hospital | Readmission | Readmission | Clinician-reported |
| 84. | Wurdeman, et al. [153] | Cross-sectional study | 0-3 months: 24 (?F, ?M)  (57.5 ± 10.3)  4-6 months: 72 (?F, ?M)  (61.9 ± 10.1)  25-36 months: 91 (?F, ?M)  (61 ± 11.7)  37-48 months: 53 (?F, ?M)  (64 ± 11.6)  49-60 months: 47 (?F, ?M)  (59.2 ± 10.7)  61-84 months: 54 (?F, ?M)  (61.1 ± 13.8) | Dyscvascular: 341  ( NA) | 0-3 months: TTA: 23  TFA: 1  (0.21 ± 0.05)  4-6 months: TTA: 56  TKA: 1  TFA: 15  (0.40 ± 0.06)  25-36 months:  TTA: 71  TKA: 1  TFA: 19  (2.5 ± 0.26)  37-48 months:  TTA: 47  TFA: 6  (3.5 ± 0.26)  49-60 months: TTA: 39  TFA: 8  (4.5 ± 0.25)  61-84 months: TTA: 44  TFA: 10  (5.9 ± 0.61) | Rehabilitation | Life impact | Physical functioning | Prosthesis-related mobility | Prosthetic limb user survey of mobility | Patient-reported |
|  |  |  |  |  |  |  | Life impact | Global QOL | Prosthesis-related QOL | PEQ-well being | Patient-reported |
| 85. | Roepke, et al. [180] | PCS | 2F, 139M  (63.3 ± 8.6) | Diabetes mellitus/PAD: 141  (NA) | TMT: 36  TTA. 74  TFA: 31  (UL: 141) | Rehabilitation | Physiological/clinical | Psychiatric outcomes | Depression | PHQ-9 | Patient reported |
|  |  |  |  |  |  |  | Physiological/clinical | Nervous system outcome | General pain | Pain intensity (0 to 10 scale) | Patient reported |
|  |  |  |  |  |  |  | Resource use | Societal/carer burden | Social support | Modified social support survey | Patient reported |
|  |  |  |  |  |  |  | Life impact | Global QOL | QOL | SF-36 | Patient reported |
| 86. | Rombauts, et al. [167] | Case study | 1M  (52) | Vascular insufficiency: 1  (8) | TTA: 1  (UL. 1) | Acute care, rehabilitation | Resource use | Need for further intervention | Use of medication | Use of medications (baclofen, clonazepam, diazepam, clotiazepam, pramipexole, pregabalin, botulinum toxin) | Clinician-reported |
|  |  |  |  |  |  |  | Resource use | Need for further intervention | Use of anesthesia | Use of peroneal nerve block | Clinician-reported |
|  |  |  |  |  |  |  | Life impact | Physical functioning | Prosthesis use | Prosthesis modification | Clinician-reported |
|  |  |  |  |  |  |  | Resource use | Need for further interventions | Rehabilitation | Delivery of physiotherapy | Clinician-reported |
|  |  |  |  |  |  |  | Physiological/clinical | Nervous system outcomes | Phantom limb pain | Phantom limb pain | Patient-reported |
|  |  |  |  |  |  |  | Physiological/clinical | Nervous system outcomes | Residual limb pain | Residual limb pain | Patient-reported |
|  |  |  |  |  |  |  | Physiological/ clinical | Nervous system outcomes | General pain | Changes/relapse in painful involuntary contractions | Patient-reported |
|  |  |  |  |  |  |  | Adverse events | Adverse events/effects | Re-amputation/revision | Re-amputation  Surgical revision | Clinician-reported |
|  |  |  |  |  |  |  | Physiological/ clinical | Psychiatric outcomes | Mental health | Psychological impact of symptoms | Patient-reported |
|  |  |  |  |  |  |  | Life impact | Physical functioning | Functional independence | Functional impact of symptoms | Patient-reported |
| 87. | Aljarrah, et al. [47] | RCS | 54F, 86M  (62.9 ± 1.1) | Diabetic foot syndrome: 102  Chronic limb ischemia: 20  Acute limb ischemia: 18  (NA) | TTA: 110  TFA: 30  (UL: 103, BL: 37) | Acute care | Death | Mortality/survival | Mortality | Mortality (1-year) | Clinician-reported |
|  |  |  |  |  |  |  | Resource use | Hospital | Duration of stay | Length of hospital stay (days) | Clinician-reported |
|  |  |  |  |  |  |  | Adverse events | Adverse events/effects | Re-amputation/revision | Need of revision surgery (ipsilateral conversion to a higher-level amputation) | Clinician-reported |
|  |  |  |  |  |  |  | Resource use | Need for further intervention | Specialist consultation | Operating surgeon specialty (Vascular/non-vascular) | Clinician-reported |
|  |  |  |  |  |  |  | Physiological/clinical | General outcomes | Comorbidities | Comorbidities (diabetes mellitus, hypertension, dyslipidemia, ischemic heart disease, congestive heart failure, chronic kidney disease, stroke, Buerger disease) | Clinician-reported |
|  |  |  |  |  |  |  | Physiological/clinical | Blood and lymphatic system outcomes | Laboratory parameters | Albumin levels | Clinician-reported |
|  |  |  |  |  |  |  | Physiological/clinical | Endocrine outcomes | Diagnostic test | Hba1c levels | Clinician-reported |
| 88. | Kelly, et al. [83] | RCS | 31F, 66M  (68) | Limb ischemia: 71  Diabetic foot infection: 18  Non-diabetic infection: 8  (NA) | TTA: 51  TKA: 5  TFA: 31  (NA) | Acute care | Adverse events | Adverse events/effects | Complications | Inpatient complication (anemia, pneumonia, cerebrovascular accident, myocardial infarction, wound infection, any complication) | Clinician-reported |
|  |  |  |  |  |  |  | Adverse events | Adverse events/effects | Re-amputation/revision | Revision amputation to higher level | Clinician-reported |
|  |  |  |  |  |  |  | Resource use | Need for further intervention | Reoperation | Repeat debridement | Clinician-reported |
|  |  |  |  |  |  |  | Resource use | Hospital | Operative outcome | Return to operation theater | Clinician-reported |
|  |  |  |  |  |  |  | Resource use | Hospital | Admission | Admission to high dependency area/intensive care unit | Clinician-reported |
|  |  |  |  |  |  |  | Death | Mortality/survival | Mortality | Mortality (30-day)  Mortality (6-month)  Mortality (1-year) | Clinician-reported |
| 89. | Lee, et al. [166] | Case study | 1M  (78) | Diabetic arterial stenosis: 1  (9) | TTA: 2  (BL: 1) | Rehabilitation | Physiological/clinical | Nervous system outcome | Radiology | Side of corticospinal tracts, lines, fractional anisotropy and apparent diffusion coefficient assessed by magnetic brain resonance imaging with diffusion tensor imaging | Clinician-reported |
|  |  |  |  |  |  |  | Resource use | Hospital | Discharge | Discharge from clinic | Clinician-reported |
|  |  |  |  |  |  |  | Physiological/clinical | Nervous system outcomes | Phantom sensation | Presence of phantom sensations | Patient-reported |
|  |  |  |  |  |  |  | Resource use | Need for further intervention | Rehabilitation | Duration of intensive rehabilitation program | Clinician-reported |
|  |  |  |  |  |  |  | Life impact | Physical functioning | Mobility | Ambulatory status | Clinician-reported |
| 90. | Kristensen, et al. [141] | Cross-sectional study | 43F, 63M  (74.2 ± 12.2) | Diabetes: 41  Arteriosclerosis: 64  (NA) | TTA: 60  TKA: 2  TFA: 40  Hip disarticulation: 1  (UL: 100, BL: 3) | Rehabilitation | Death | Mortality/survival | Mortality | Mortality (30-day) | Clinician-reported |
|  |  |  |  |  |  |  | Life impact | Physical functioning | Mobility | Basic amputee mobility score | Performance-related |
|  |  |  |  |  |  |  | Resource use | Hospital | Discharge | Discharged/not discharged to previous residence  Postoperative days of discharge | Clinician-reported |
|  |  |  |  |  |  |  | Resource use | Need for further intervention | Use of anesthesia | American Society of Anesthesiologists grade | Clinician-reported |
|  |  |  |  |  |  |  | Life impact | Physical functioning | Balance | One leg stand test | Performance-related |
|  |  |  |  |  |  |  | Life impact | Delivery of care | Reliability of scoring | Reliability of Basic amputee mobility score scoring between experienced and inexperienced rater | Clinician-reported |
| 91. | Janssen, et al. [81] | RCS | 41F, 83M  (70 ± 11.5) | Chronic limb ischemia: 124  (NA) | TTA: 67  TFA: 57  (UL: 122, BL: 2) | Acute care | Physiological/clinical | Vascular outcomes | Diagnostic test | Duplex ultrasound (open, stenosis, occlusion, not described)  Physical examination of popliteal and femoral pulse (palpable, not palpable, not described) | Clinician-reported |
|  |  |  |  |  |  |  | Death | Mortality/survival | Mortality | Mortality (30-day)  Mortality (1-year) | Clinician-reported |
|  |  |  |  |  |  |  | Resource use | Need for further intervention | Reoperation | Reoperation rate | Clinician-reported |
|  |  |  |  |  |  |  | Adverse events | Adverse events/effects | Re-amputation/revision | Conversion from TTA to TFA | Clinician-reported |
| 92. | Gailey, et al. [187] | RCT | Intervention: 3F, 6M  (63.4 ± 11.5)  Waitlist: 7M  (63 ± 7.1) | Intervention: Diabetes mellitus: 4  PVD: 0  Diabetes mellitus and PVD: 1  Trauma: 3  Tumor: 1  (11.6 ± 11.4)  Waitlist: Diabetes mellitus: 1  PVD: 3  Diabetes mellitus and PVD: 3  Trauma: 0  Tumor: 0  (4.2 ± 4.3) | Intervention: TTA: 9  (UL: 9)  Waitlist:  TTA: 7  (UL: 7) | Rehabilitation | Life impact | Physical functioning | Balance confidence | ABC scale | Patient-reported |
|  |  |  |  |  |  |  | Life impact | Physical functioning | Gait: spatiotemporal walking | 6MWT | Performance-related |
|  |  |  |  |  |  |  | Life impact | Physical functioning | Mobility | Amputee mobility predictor with/without prosthesis | Performance-related |
|  |  |  |  |  |  |  | Physiological/ clinical | Cardiovascular outcomes | Diagnostic test | Heart rate | Clinician-reported |
|  |  |  |  |  |  |  | Life impact | Perceived health status | Perceived exertion | Rate of perceived exertion | Clinician-reported |
| 93. | Boussi, et al. [155] | Case study | 1M  (63) | Ischemia: 1  (1) | TTA: 1  (UL: 1) | Acute care | Resource use | Hospital | Duration of stay | Length of hospital stay | Clinician-reported |
|  |  |  |  |  |  |  | Resource use | Need for further intervention | Use of medication | Use of medications (heparin, steroids) | Clinician-reported |
|  |  |  |  |  |  |  | Physiological/clinical | Cardiovascular outcome | Diagnostic test | Doppler sonography (to detect brachial, ulnar, radial signals) | Clinician-reported |
|  |  |  |  |  |  |  | Physiological/clinical | Vascular outcomes | Radiology | Computed tomography angiogram | Clinician-reported |
|  |  |  |  |  |  |  | Physiological/clinical | Nervous system outcomes | General pain | Pain | Patient-reported |
|  |  |  |  |  |  |  | Death | Mortality/survival | Mortality | Mortality | Clinician-reported |
|  |  |  |  |  |  |  | Adverse events | Adverse events/effects | Complications | Clinical course including complications (acute renal failure, leukopenia, lactic acidosis with progressive hypotension) | Clinician-reported |
|  |  |  |  |  |  |  | Physiological/ clinical | Psychiatric outcomes | Mental health | Change in mental status | Clinician-reported |
|  |  |  |  |  |  |  | Resource use | Hospital | Admission | Admission to emergency care | Clinician-reported |
| 94. | Columbo, et al. [190] | Mixed method approach | 3F, 17M  (65, 45 to 88) | Arterial vascular disease: 20  (16, 4 to 51) | TTA: 14  TFA: 6  (UL: 17, BL: 3) | Community | Life impact | Global QOL | QOL | Structured interviews on quality-of-life domains relevant to vascular amputees (mobility, pain, concern about their remaining limb, mental health, independence, social function, personal postoperative experience and feelings at time of interview), additional focus group interviews for further exploration of structured interviews) | Patient-reported |
| 95. | Norvell, et al. [100] | RCS | TMT: 28F, 1480M  (65.6 ± 10)  TTA: 39F, 3227M  (66.8 ± 10.2)  TFA: 28F, 2385M  (70.7 ± 10.7) | Diabetes and/or PAD: 7187  (NA) | TMT: 1508  TTA: 3266  TFA: 2413  (UL) | Acute care | Adverse events | Adverse events/effects | Re-amputation/revision | Ipsilateral re-amputation, to a higher level (1-year)  Number of revision procedures (1-year) | Clinician-reported |
| 96. | Molina-Rueda, et al. [145] | Cross-sectional study | Vacscular: 2F, 7M  (54.8 ± 10.5)  Non-vascular: 1F, 8M  (47.6 ± 14.2)  Control: 3F, 21M  (54.7 ± 8.2) | Vascular: Diabetes and/or PVD: 9  (9.7 ± 2.3)  Non-vascular: Trauma: 7  Tumor: 2  (9.4 ± 4.2) | Vascular: TTA: 9  (UL: 9)  Non-vascular:  TTA: 9  (UL: 9) | Rehabilitation | Life impact | Physical functioning | Balance | Centre of gravity excursions (maximum, endpoint, directional control) assessed by Computerized dynamic posturography (with Smart equitest system) to assess stability | Performance-related |
| 97. | Miller, et al. [192] | Mixed method approach | 2F, 18F  (63.4, 57.5 to 70) | Dysvascular: 20  (5.5, 2.4 to 6.7) | TTA: 15  TFA: 2  Not reported: 3  (UL: 17, BL: 3) | Community | Life impact | Physical functioning | Mobility | TUG | Performance-related |
|  |  |  |  |  |  |  | Life impact | Physical functioning | Gait: Spatiotemporal walking | Daily step count | Performance-related |
|  |  |  |  |  |  |  | Life impact | Physical functioning | Impact of disease/condition on ADL | World Health Organization disability assessment schedule 2.0 | Patient-reported |
|  |  |  |  |  |  |  | Life impact | Global QOL | QOL | Semi-structured interview (general health, rehabilitation after amputation, physical exercise, prosthesis, post-amputation phase) | Patient-reported |
| 98. | Wied, et al. [127] | RCS | 25F, 49M  (72.3 ± 11) | Diabetes/arteriosclerosis: 35  Other: 4  (NA) | TTA: 39  (UL: 39) | Acute care | Resource use | Need for further intervention | Torniquet use | Duration of torniquet | Clinician-reported |
|  |  |  |  |  |  |  | Physiological/clinical | Blood and lymphatic system outcomes | Blood loss | Total blood loss (from day 0 to 4)  Delta total intraoperative blood loss | Clinician-reported |
|  |  |  |  |  |  |  | Physiological/clinical | Blood and lymphatic system outcomes | Blood transfusion | Blood transfusion per kilogram of bodyweight | Clinician-reported |
|  |  |  |  |  |  |  | Death | Mortality/survival | Mortality | Mortality (30-day) | Clinician-reported |
|  |  |  |  |  |  |  | Adverse events | Adverse events/effects | Re-amputation/revision | Re-amputation (30-day) | Clinician-reported |
|  |  |  |  |  |  |  | Resource use | Need for further intervention | Use of medication | Use of anti-fibrinolytic medication | Clinician-reported |
|  |  |  |  |  |  |  | Resource use | Hospital | Duration of operation | Duration of surgery | Clinician-reported |
|  |  |  |  |  |  |  | Resource use | Need for further intervention | Specialist consultation | Rank of surgeon (resident/consultant) | Clinician-reported |
|  |  |  |  |  |  |  | Physiological/clinical | Blood and lymphatic system outcomes | Laboratory parameters | Hemoglobin level  Thrombocyte level | Clinician-reported |
|  |  |  |  |  |  |  | Physiological/clinical | Metabolism and nutrition outcome | Body mass | BMI | Clinician-reported |
|  |  |  |  |  |  |  | Resource use | Need for further intervention | Use of anesthesia | American Society of anesthesiologist’s grade | Clinician-reported |
| 99. | Roijers, et al. [112] | RCS | 41F, 54M  (80.2 ± 7) | Chronic limb ischemia: 95  (NA) | TTA: 13  TKA: 2  TFA: 14  (NA) | Acute care | Physiological/clinical | Psychiatric outcomes | Delirium | Delirium (pre- and post-operative)  Length of delirium (days) | Clinician-reported |
|  |  |  |  |  |  |  | Resource use | Need for further intervention | Use of anesthesia | Duration of anesthesia (minutes) | Clinician-reported |
|  |  |  |  |  |  |  | Resource use | Need for further intervention | Use of anesthesia | Type of general anesthesia | Clinician-reported |
|  |  |  |  |  |  |  | Physiological/clinical | Blood and lymphatic system outcomes | Anemia | Anemia (pre- and post-operative) | Clinician-reported |
|  |  |  |  |  |  |  | Physiological/clinical | Blood and lymphatic system outcomes | Blood transfusion | Blood transfusion | Clinician-reported |
|  |  |  |  |  |  |  | Adverse events | Adverse events/effects | Adverse events | Adverse events (cardiac, pulmonary, cerebrovascular, renal/urinary, wound infection, hemorrhage) | Clinician-reported |
|  |  |  |  |  |  |  | Resource use | Hospital | Duration of stay | Length of admission (days) | Clinician-reported |
|  |  |  |  |  |  |  | Resource use | Hospital | Admission | Living in a nursing home | Clinician-reported |
|  |  |  |  |  |  |  | Resource use | Hospital | Readmission | Re-admission (30-days) | Clinician-reported |
|  |  |  |  |  |  |  | Death | Mortality/survival | Mortality | Mortality (1-year) | Clinician-reported |
|  |  |  |  |  |  |  | Death | Mortality/survival | Survival | Median survival time | Clinician-reported |
| 100. | Ichihashi, et al. [159] | Case study | 1M  (82) | Gangrene from Buerger’s disease: 1  (NA) | TFA: 1  (UL: 1) | Acute care | Physiological/clinical | Vascular outcome | Radiology | Computed tomography angiography assessment of occlusion (right external iliac, common femoral, superficial femoral and profunda femoris arteries) | Clinician-reported |
|  |  |  |  |  |  |  | Resource use | Hospital | Operative success | Preservation of collateral circulation  Success of endovascular aneurysm repair procedure | Clinician-reported |
|  |  |  |  |  |  |  | Physiological/clinical | Vascular outcome | Diagnostic test | Intravascular ultrasound | Clinician-reported |
| 101. | Mtshali, et al. [93] | RCS | 16F, 18M  (64 ± 9.6) | Diabetes-related: 34  (NA) | Foot: 8  TTA/TFA: 26  (NA) | Acute care | Resource use | Economic | Cost | Total summative costs of managing patients in dollars (mean and standard deviation for compensation of employees, theatre costs, radiology costs, laboratory costs, costs of medicines, goods and services for intensive care unit and general wards) | Clinician-reported |
|  |  |  |  |  |  |  | Resource use | Hospital | Duration of stay | Length of hospital stay | Clinician-reported |
| 102. | Gurel, et al. [75] | RCS | TTA with torniquet: 4F, 25M  (67.7 ± 12.3)  TTA without torniquet:  9F, 31M  (70.7 ± 11.9) | TTA with torniquet: DFI/PVD: 29  (NA)  TTA without torniquet:  DFI/PVD: 40  (NA) | TTA with torniquet: TTA: 29  (NA)  TTA without torniquet: TTA: 40  (NA) | Acute care | Resource use | Hospital | Duration of operation | Duration of operation (minutes) | Clinician-reported |
|  |  |  |  |  |  |  | Physiological/clinical | Blood and lymphatic system outcomes | Blood transfusion | Use of blood transfusion (intra- and post-operative) | Clinician-reported |
|  |  |  |  |  |  |  | Resource use | Need for further intervention | Torniquet use | Use of torniquet during the operation | Clinician-reported |
|  |  |  |  |  |  |  | Physiological/clinical | Blood and lymphatic system outcomes | Laboratory parameters | Hemoglobin levels | Clinician-reported |
|  |  |  |  |  |  |  | Death | Mortality/survival | Mortality | Mortality (60-day) | Clinician-reported |
|  |  |  |  |  |  |  | Physiological/clinical | Infection and infestation outcomes | Infection | Surgical site infection | Clinician-reported |
|  |  |  |  |  |  |  | Resource use | Need for further intervention | Reoperation | Need for re-operation (60-day) | Clinician-reported |
|  |  |  |  |  |  |  | Resource use | Need for further intervention | Specialist consultation | Visit to outpatient clinics (2 and 6 weeks after discharge) | Clinician-reported |
|  |  |  |  |  |  |  | Resource use | Hospital | Duration of stay | Length of hospital stay (days) | Clinician-reported |
| 103. | Brugliera, et al. [195] | Case series | 3M  (68 ± 8.1) | Acute ischemia: 3  (NA) | TFA: 4  (UL: 3, BL: 1) | Acute care, rehabilitation | Resource use | Need for further intervention | Use of medication | Use of medications (antibiotics, antiviral, acetylsalicylic acid, statins, low molecular weight heparin, alpostradil, Iloprost) | Clinician-reported |
|  |  |  |  |  |  |  | Resource use | Need for further intervention | Respiratory support | Use of respiratory support (intubation, continuous positive airway support) | Clinician-reported |
|  |  |  |  |  |  |  | Physiological/clinical | Blood and lymphatic system outcomes | Laboratory parameters | Fibrinogen level  Lactate dehydrogenase level  Platelet count  Partial thromboplastin time | Clinician-reported |
|  |  |  |  |  |  |  | Physiological/clinical | Blood and lymphatic system outcomes | Biomarker | D-dimer levels | Clinician-reported |
|  |  |  |  |  |  |  | Resource use | Hospital | Admission | Admission to rehabilitation unit | Clinician-reported |
|  |  |  |  |  |  |  | Life impact | Physical functioning | Functional independence | Functional independence | Clinician-reported |
|  |  |  |  |  |  |  | Life impact | Physical functioning | Locomotion ability | Walking ability with support | Clinician-reported |
|  |  |  |  |  |  |  | Resource use | Need for further intervention | Rehabilitation | Duration of rehabilitation | Clinician-reported |
| 104. | Dallas, et al. [156] | Case study | 1M  (81) | Diabetes, PAD and hypercholesterolemia: 1  (NA) | TFA: 1  (UL: 1) | Acute care | Physiological/clinical | Vascular outcome | Radiology | Computed tomography angiography to ascertain pseudoaneurysm | Clinician-reported |
|  |  |  |  |  |  |  | Life impact | Physical functioning | Mobility potential | K-level mobility with prosthesis | Clinician-reported |
|  |  |  |  |  |  |  | Life impact | Physical functioning | Prosthesis fit | Prosthesis fitting | Patient-reported |
|  |  |  |  |  |  |  | Life impact | Physical functioning | Prosthesis use | Duration of prosthesis use (hours/day) | Patient-reported |
|  |  |  |  |  |  |  | Life impact | Physical functioning | Endurance | Standing tolerance (minutes) | Patient-reported |
|  |  |  |  |  |  |  | Life impact | Physical functioning | Locomotion ability | Walking ability (with walker assistance) | Patient-reported |
|  |  |  |  |  |  |  | Physiological/ clinical | Nervous system outcomes | General pain | Numerical rating of non-radiating pain | Patient-reported |
|  |  |  |  |  |  |  | Physiological/clinical | General outcomes | Diagnostic test | Ultrasound imaging | Clinician-reported |
|  |  |  |  |  |  |  | Resource use | Hospital | Admission | Admission to operating room | Clinician-reported |
|  |  |  |  |  |  |  | Resource use | Hospital | Discharge | Discharge from hospital | Clinician-reported |
|  |  |  |  |  |  |  | Life impact | Physical functioning | Prosthesis use | Re-initiation of prosthesis use | Clinician-reported |
|  |  |  |  |  |  |  | Physiological/ clinical | Skin and subcutaneous tissue outcomes | Stump wound healing | Wound healing | Clinician-reported |
| 105. | Ogaki, et al. [102] | RCS | 50F, 88M  (72 ± 11) | CLTI and after endovascular therapy: 138  (NA) | Major amputation (level not reported  (NA) | Acute care | Death | Mortality/survival | Mortality | Mortality (30-day)  Mortality (2-year) | Clinician-reported |
|  |  |  |  |  |  |  | Adverse events | Adverse events/effects | Re-amputation/revision | Additional (ipsilateral/ contralateral) amputation (30-day)  Additional (ipsilateral/ contralateral) amputation (2-year) | Clinician-reported |
|  |  |  |  |  |  |  | Death | Mortality/survival | Cause of mortality | Cause of mortality (infection, cardiovascular disease, other causes) | Clinician-reported |
|  |  |  |  |  |  |  | Physiological/clinical | Cardiovascular outcomes | Diagnostic test | Echocardiography (ejection fraction) | Clinician-reported |
|  |  |  |  |  |  |  | Life impact | Physical functioning | Mobility | Mobility at 2-year (wheelchair, bedridden, ambulatory) | Clinician-reported |
| 106. | Shamir, et al. [114] | RCS | 161F, 229M  (73.5 ± 12.5) | Diabetic foot: 197  Ischemia: 122  Other: 40  (NA) | Foot: 20  Toe: 7  TTA: 140  TFA: 200  Other: 3  (NA) | Acute care | Adverse events | Adverse events/effects | Re-amputation/revision | Re-amputation (1-month) | Clinician-reported |
|  |  |  |  |  |  |  | Death | Mortality/survival | Mortality | Mortality during hospitalization | Clinician-reported |
|  |  |  |  |  |  |  | Adverse events | Adverse events/effects | Complications | Post-operative complications (nosocomial bacteremia, Clostridioides difficile infection) | Clinician-reported |
|  |  |  |  |  |  |  | Resource use | Hospital | Duration of stay | Duration of hospitalization (days) | Clinician-reported |
|  |  |  |  |  |  |  | Resource use | Need for further intervention | Respiratory support | Intubation | Clinician-reported |
|  |  |  |  |  |  |  | Resource use | Need for further intervention | Resuscitation | Need for resuscitation | Clinician-reported |
|  |  |  |  |  |  |  | Resource use | Need for further intervention | Use of medication | Duration of antibiotic treatment (days) | Clinician-reported |
|  |  |  |  |  |  |  | Life impact | Delivery of care | Time to receive services | Duration of delayed amputation (days)  Reason for delayed amputation (no delay, technical issue, patient medical status, patient/guardian refusal) | Clinician-reported |
| 107. | Friedman, et al. [71] | RCS | 2F, 14M  (57.5 ± 10.1) | Diabetes and vascular causes: 16  (NA) | TTA: 15  TFA: 1  (UL: 16) | Rehabilitation | Physiological/clinical | General outcomes | Amputation | Amputation of dominant limb | Clinician-reported |
| 108. | Günay, et al. [74] | RCS | 35F, 52M  (70.6 ± 1.3) | Diabetic foot ulcer: 64  PAD: 23 | TTA: 73  TKA: 2  TFA: 12  (UL: 87) | Acute care | Resource use | Hospital | Duration of stay | Duration of hospital stay (days) | Clinician-reported |
|  |  |  |  |  |  |  | Resource use | Hospital | Duration of operation | Duration of operation (minutes) | Clinician-reported |
|  |  |  |  |  |  |  | Physiological/clinical | Immune system outcomes | Laboratory parameters | White blood cell count (pre-, post-operative)  Neutrophil count (pre-, post-operative)  Lymphocyte count (pre-, post-operative)  C-reactive protein count (pre-, post-operative) | Clinician-reported |
|  |  |  |  |  |  |  | Physiological/clinical | Blood and lymphatic system outcomes | Laboratory parameters | Hemoglobin level (pre-, post-operative)  Platelet count (pre-, post-operative)  Albumin level (pre-, post-operative) | Clinician-reported |
|  |  |  |  |  |  |  | Physiological/clinical | Renal and urinary outcomes | Laboratory parameters | Creatinine level (pre-, post-operative) | Clinician-reported |
|  |  |  |  |  |  |  | Physiological/clinical | Immune system outcomes | Biomarkers | Neutrophil /Albumin ratio (pre-, post-operative)  CRP/Albumin ratio (pre-, post-operative)  Platelet/lymphocyte ratio (pre-, post-operative)  Neutrophil/lymphocyte ratio (pre-, post-operative) | Clinician-reported |
|  |  |  |  |  |  |  | Death | Mortality/survival | Mortality | Mortality (≤30-day)  Mortality (>30-day) | Clinician-reported |
| 109. | Junaidi, et al. [82] | RCS | 31F, 27M  (60.2 ± 8.6) | PAD: 58  (NA) | TTA: 58  (NA) | Acute care | Physiological/clinical | Vascular outcome | Diagnostic test | Distal artery spectral waveform (triphasic, biphasic, monophasic)  Peak systolic velocity (cm/sec)  Volume flow (ml/min)  Arterial diameter (cm) with Doppler ultrasonography on popliteal artery, anterior tibial artery, and posterior tibial artery | Clinician-reported |
| 110. | Kishikawa, et al. [163] | Case study | 1M  (51) | Acute arterial thrombosis: 1  (1) | TTA: 1  (UL: 1) | Acute care, rehabilitation | Physiological/ clinical | Nervous system outcomes | Phantom limb pain | Phantom limb pain post-operative (subjective) | Patient-reported |
|  |  |  |  |  |  |  | Resource use | Need for further intervention | Use of anesthesia | Use of general and epidural anesthesia  Use of subarachnoid block (hyperbaric tetracaine) | Clinician-reported |
|  |  |  |  |  |  |  | Physiological/ clinical | Nervous system outcomes | Residual limb pain | Stump pain (subjective) | Patient-reported |
|  |  |  |  |  |  |  | Resource use | Need for further intervention | Use of medication | Use of medications (pentazocine, midazolam, diclofenac, hydroxyzine, antibiotics) | Clinician-reported |
|  |  |  |  |  |  |  | Resource use | Hospital | Duration of operation | Duration of surgery (minutes) | Clinician-reported |
|  |  |  |  |  |  |  | Adverse events | Adverse events/effects | Complications | Post-operative complications (wound pain, stump pain, pyrexia) | Clinician-reported |
|  |  |  |  |  |  |  | Resource use | Hospital | Admission | Transfer to general ward, orthopedics | Clinician-reported |
|  |  |  |  |  |  |  | Life impact | Physical functioning | Prosthesis-related mobility | Walking ability with prosthesis and support | Performance-related |
|  |  |  |  |  |  |  | Resource use | Hospital | Discharge | Discharge to home | Clinician-reported |
|  |  |  |  |  |  |  | Physiological/clinical | Nervous system outcomes | Low back pain | Low back pain (subjective) | Patient-reported |
| 111. | Clemens, et al. [138] | Cross-sectional study | 19F, 30M  (62.1 ± 9.7) | Dysvascular: 49  (6.9 ± 6.6) | TTA: 32  TFA: 8  (UL: 40, BL: 9) | Community | Life impact | Physical functioning | Mobility | TUG | Performance-related |
|  |  |  |  |  |  |  | Life impact | Physical functioning | Gait: spatiotemporal walking | 2MWT | Performance-related |
|  |  |  |  |  |  |  | Life impact | Physical functioning | Prosthesis-related mobility | Prosthetic limb user survey of mobility (12-item short form) | Patient-reported |
|  |  |  |  |  |  |  | Life impact | Social functioning | Social participation | PROMIS (participation in social roles and activities) | Patient-reported |
|  |  |  |  |  |  |  | Physiological/clinical | General outcomes | Comorbidities | Functional comorbidity index | Patient-reported |
|  |  |  |  |  |  |  | Life impact | Physical functioning | Prosthesis use | Environmental factors item bank (assessed effects of environment on prosthetic functioning, accessibility) | Patient-reported |
| 112. | Hagan, et al. [76] | RCS | 68F, 58M  (60.9 ± 19) | Diabetic foot ulcer: 54  PVD: 43  Infection/chronic osteomyelitis/chronic ulcer with deformity: 9  Tumor: 7  Other: 1  (NA) | TTA: 55  TKA: 1  TFA: 53  Ray’s amputation: 6  Digit (hand): 2  Below elbow: 1  Rays + TFA: 1  (UL: 120, BL: 6) | Acute care | Resource use | Economic | Insurance coverage | National health insurance coverage of amputees | Patient-reported |
|  |  |  |  |  |  |  | Physiological/clinical | General outcome | Amputation | Stages of amputation (single/double stage) | Clinician-reported |
|  |  |  |  |  |  |  | Physiological/clinical | General outcomes | Co-morbidities | Presence of diabetes as comorbidity | Clinician-reported |
| 113. | Cotton, et al. [60] | RCS | 298M  (67 ± 10) | PVD: 298  (NA) | TTA: 164  TFA: 134  (NA) | Acute care | Physiological/clinical | General outcomes | Frailty | Modified frailty index-11 (classify frail/non-frail)  Risk analysis index to classify frailty | Clinician-reported |
|  |  |  |  |  |  |  | Death | Mortality/survival | Mortality | Mortality (30-day)  Mortality (180-day)  Mortality (365-day) | Clinician-reported |
|  |  |  |  |  |  |  | Adverse events | Adverse events/effects | Adverse events | Adverse events within 30-day (surgical site infection, cardiac events, stroke, renal failure) | Clinician-reported |
|  |  |  |  |  |  |  | Resource use | Hospital | Readmission | Readmission (30-day) | Clinician-reported |
|  |  |  |  |  |  |  | Resource use | Need for further intervention | Reoperation | Unplanned operation (30-day) | Clinician-reported |
|  |  |  |  |  |  |  | Resource use | Hospital | Duration of stay | Length of hospital stay (days) | Clinician-reported |
| 114. | Mayo, et al. [142] | Cross-sectional study | 45F, 186M  (63.4, 31 to 93) | Diabetes mellitus and/or PAD 231  (3.4, 0.5 to 27) | TTA: 171  TFA: 24  (UL: 195, BL: 36) | Community | Physiological/clinical | General outcomes | Comorbidities | Dysvascular conditions scale based on the Secondary conditions scale | Patient-reported |
|  |  |  |  |  |  |  | Life impact | Physical functioning | Mobility | Special interest group in amputee medicine to assess clinical mobility (grade A to F) | Patient-reported |
| 115. | de Boer, et al. [65] | RCS | 65F, 210M  (66.6 ± 15.5) | Ischemia: 143  Infection: 88  Tumor: 22  Trauma: 13  Other: 9  (NA) | TTA: 155  TFA: 120  (UL: 275) | Rehabilitation | Life impact | Physical functioning | Mobility | Mobility status (ambulant, non-ambulant) | Clinician-reported |
|  |  |  |  |  |  |  | Resource use | Hospital | Duration of stay | Length of hospital stay (days) | Clinician-reported |
|  |  |  |  |  |  |  | Resource use | Hospital | Discharge | Discharge destination (Rehabilitation, own home, other hospital, residential care) | Clinician-reported |
|  |  |  |  |  |  |  | Life impact | Physical functioning | Prosthesis use | Prosthesis use | Clinician-reported |
| 116. | Kalkan, et al. [161] | Case study | 1M  (58) | Stent occlusion: 1  (11) | TFA: 1  (UL: 1) | Acute care | Physiological/ clinical | Skin and subcutaneous tissue outcomes | Stump wound healing | Physical examination of stump wound (presence of edema, discharge, infection) | Clinician-reported |
|  |  |  |  |  |  |  | Physiological/clinical | Cardiovascular outcome | Diagnostic test | Blood pressure  Heart rate | Clinician-reported |
|  |  |  |  |  |  |  | Physiological/clinical | Immune system outcomes | Laboratory parameters | White blood cell count  C-reactive protein level | Clinician-reported |
|  |  |  |  |  |  |  | Physiological/clinical | Renal and urinary outcomes | Laboratory parameters | Blood urea nitrogen level  Creatinine level | Clinician-reported |
|  |  |  |  |  |  |  | Physiological/clinical | Infection and infestation outcomes | Microbial culture | Wound culture | Clinician-reported |
|  |  |  |  |  |  |  | Resource use | Need for further intervention | Use of medication | Use of medications (ceftriaxone, metronidazole) | Clinician-reported |
|  |  |  |  |  |  |  | Resource use | Hospital | Duration of stay | Length of hospital stay | Clinician-reported |
| 117. | Otsuka, et al. [104] | RCS | 41F, 81M  (66.6 ± 11) | Arteriosclerosis obliterans/ diabetes mellitus: 122  (NA) | TTA: 71  TFA: 51  (UL: 122) | Acute care | Death | Mortality/survival | Mortality | Mortality (30-day) | Clinician-reported |
|  |  |  |  |  |  |  | Resource use | Need for further intervention | Emergency procedures | Need for emergency surgery | Clinician-reported |
|  |  |  |  |  |  |  | Resource use | Need for further intervention | Use of anesthesia | American Society of Anesthesiologists-Physical Status classification | Clinician-reported |
|  |  |  |  |  |  |  | Adverse events | Adverse events/effects | Complications | Peri-operative complications (sepsis, acute kidney injury, delirium, respiratory insufficiency, hypoalbuminemia) | Clinician-reported |
|  |  |  |  |  |  |  | Resource use | Hospital | Admission | ICU admission | Clinician-reported |
|  |  |  |  |  |  |  | Physiological/clinical | Blood and lymphatic system outcomes | Blood transfusion | Need for blood transfusion | Clinician-reported |
| 118. | Haleem, et al. [77] | RCS | Amputee: 16F, 12M  (78 ± 9.9)  Control: 5816F, 1971F  (80.5 ± 11.3) | Amputee:  PVD: 13  Diabetes: 9  Rheumatoid arthritis: 1  Not reported: 5  (NA) | Amputee: TTA: 25  TFA: 6  (UL: 24, BL: 4) | Acute care | Death | Mortality/survival | Mortality | Mortality (1-year) | Clinician-reported |
|  |  |  |  |  |  |  | Physiological/ clinical | Nervous system outcomes | General pain | Pain score | Clinician-reported |
|  |  |  |  |  |  |  | Life impact | Physical functioning | Mobility | Mobility score (1-year) | Clinician-reported |
|  |  |  |  |  |  |  | Resource use | Need for further intervention | Mobility aid | Use of aids pre-injury, post-operative (none, one stick, two sticks, Zimmer, wheelchair) | Clinician-reported |
|  |  |  |  |  |  |  | Life impact | Personal circumstances | Residential status | Residential status pre-injury, post-operative (own home, residential home, nursing home) | Clinician-reported |
|  |  |  |  |  |  |  | Physiological/clinical | Injury and poisoning outcomes | Fracture | Frequency of fractures (intracapsular, extracapsular) | Clinician-reported |
|  |  |  |  |  |  |  | Resource use | Need for further intervention | Management of fractures | Management of fractures (multiple parallel screws, cemented hemiarthroplasty, uncemented hemiarthroplasty, sliding hip screw, intramedullary nail, cemented hemiarthroplasty, non-operative) | Clinician-reported |
| 119. | Shin, et al. [115] | RCS | Delirium: 3F, 37M  (73.6 ± 7)  No-delirium: 2F, 57M  (73.3 ± 8) | Delirium: CLTI: 40  (NA)  No-delirium: 59  (NA) | Delirium: TTA: 35  TFA: 5  (UL: 40)  No-delirium: TTA: 45  TFA: 14  (UL: 59) | Acute care | Resource use | Hospital | Duration of operation | Duration of operation (minutes) | Clinician-reported |
|  |  |  |  |  |  |  | Resource use | Need for further intervention | Use of anesthesia | Type of anesthesia (general/regional)  Duration of anesthesia | Clinician-reported |
|  |  |  |  |  |  |  | Physiological/clinical | Blood and lymphatic system outcomes | Blood loss | Estimated blood loss (ml) | Clinician-reported |
|  |  |  |  |  |  |  | Physiological/clinical | Blood and lymphatic system outcomes | Blood transfusion | Blood transfusion | Clinician-reported |
|  |  |  |  |  |  |  | Resource use | Need for further intervention | Use of medication | Use of inotropic drugs during operation | Clinician-reported |
|  |  |  |  |  |  |  | Physiological/clinical | Blood and lymphatic system outcomes | Laboratory parameters | Laboratory values Hemoglobin  Sodium  Potassium  Albumin  Total protein  Erythrocyte sedimentation rate  PaO_2_  PaCO_2_ | Clinician-reported |
|  |  |  |  |  |  |  | Physiological/clinical | Renal and urinary outcomes | Laboratory parameters | Blood urea nitrogen  Creatinine level | Clinician-reported |
|  |  |  |  |  |  |  | Physiological/clinical | Immune system outcomes | Laboratory parameters | C-reactive protein | Clinician-reported |
|  |  |  |  |  |  |  | Physiological/clinical | Endocrine outcome | Laboratory parameters | Fasting blood sugar | Clinician-reported |
|  |  |  |  |  |  |  | Resource use | Hospital | Admission | Transfer to ICU | Clinician-reported |
|  |  |  |  |  |  |  | Resource use | Hospital | Duration of stay | Duration of hospital (days)  Duration of ICU stay (days) | Clinician-reported |
|  |  |  |  |  |  |  | Adverse events | Adverse events/effects | Complications | Post-operative complications | Clinician-reported |
|  |  |  |  |  |  |  | Death | Mortality/survival | Mortality | Mortality (6-month) | Clinician-reported |
| 120. | Meyer, et al. [91] | RCS | 136F, 300M  (74.7, 45 to 98) | Chronic limb ischemia: 272  Acute ischemia/occlusion: 113  Uncontrolled infection with sepsis: 50  Not reported 1  (NA) | TTA: 133  TKA: 5  TFA: 298  (UL: 436) | Acute care | Adverse events | Adverse events/effects | Complications | Post-operative complications (including re-surgery following index amputation (infection, stump ischemia, wound revision, proximal lower/upper leg amputation) | Clinician-reported |
|  |  |  |  |  |  |  | Death | Mortality/survival | Survival | Duration of post-operative survival (days)  Survival rate (6-month)  Survival rate (1-year)  Survival rate (5-year) | Clinician-reported |
|  |  |  |  |  |  |  | Death | Mortality/survival | Mortality | In-hospital mortality | Clinician-reported |
| 121. | Rammell, et al. [110] | RCS | Non-anemic: 26F, 79M  (66 ± 15)  Mild anemic: 37F, 74M  (66 ± 12)  Severe anemic: 52F, 77M  (66 ± 12) | Non-anemic: PAD: 105  (NA)  Mild anemic: PAD: 111  (NA)  Severe anemic: PAD: 129  (NA) | Non-anemic: TTA: 49  TFA: 56  (UL: 105)  Mild anemic: TTA: 59  TFA: 52  (UL: 111)  Severe anemic: TTA: 39  TFA: 91  (UL: 129) | Acute care | Death | Mortality/survival | Mortality | Overall Mortality (30-day)  Overall Mortality (23-month) | Clinician-reported |
|  |  |  |  |  |  |  | Resource use | Hospital | Duration of stay | Length of hospital stay (days) | Clinician-reported |
|  |  |  |  |  |  |  | Adverse events | Adverse events/effects | Complications | Complications (medical: myocardial infarction, pneumonia, surgical: surgical site infection, stump dehiscence, need for surgical revision) | Clinician-reported |
|  |  |  |  |  |  |  | Physiological/clinical | Blood and lymphatic system outcomes | Blood transfusion | Blood transfusion (day of surgery to 7^th^ post-operative day) | Clinician-reported |
|  |  |  |  |  |  |  | Resource use | Hospital | Duration of operation | Length of operation (minutes) | Clinician-reported |
| 122. | Bosanquet, et al. [185] | RCT | 10F, 40M  (69.8 ± 10) | PAD: 50  (NA) | TTA: 31  TFA: 19  (UL: 50) | Acute care | Physiological/ clinical | Nervous system outcomes | General pain | Verbal rating scale for pain (0 to 10) | Patient-reported |
|  |  |  |  |  |  |  | Life impact | Global QOL | QOL | EQ-5D-5L | Patient-reported |
|  |  |  |  |  |  |  | Physiological/ clinical | Psychiatric outcomes | Depression | Depression (Hospital anxiety and depression scale) | Patient-reported |
|  |  |  |  |  |  |  | Physiological/ clinical | Psychiatric outcomes | Anxiety | Anxiety (Hospital anxiety and depression scale) | Patient-reported |
|  |  |  |  |  |  |  | Resource use | Need for further intervention | Use of medication | Use of opioids | Clinician-reported |
|  |  |  |  |  |  |  | Life impact | Delivery of care | Feasibility | Proportion of eligible participants: Recruited  Provided informed consent  Reached follow-up (2-month, 6-month) | Clinician-reported |
|  |  |  |  |  |  |  | Life impact | Delivery of care | Fidelity | Proportion of recruited participants who provided adequate putative primary effectiveness outcomes data | Clinician-reported |
|  |  |  |  |  |  |  | Resource use | Economic | Cost | Economic evaluation (key cost drivers, resource usage during first 6-month post-operation i.e., general practitioner consultation, practice nurse consultation, primary care contact, other hospital contact, National Health Service contact, ambulance/paramedic contact, attendance at accident and emergency care, nights spent at hospital, contact at surgical/rehabilitation department, visits to hospital, saw a specialist nurse, saw a physiotherapist/occupational therapist)  Intervention costs with perineural catheter (equipment used: reservoir pump, epidural mini, catheter replacement, staff time, drug acquisition) | Clinician-reported |
|  |  |  |  |  |  |  | Physiological/clinical | Nervous system outcome | Pain relief | Overall benefit of analgesia score | Patient-reported |
|  |  |  |  |  |  |  | Physiological/clinical | Nervous system outcome | Neuropathic pain | Leeds assessment of neuropathic symptoms and signs scores | Patient-reported |
|  |  |  |  |  |  |  | Adverse events | Adverse events/effects | Adverse events | Incidence of nausea and vomiting (during in-patient stay) | Clinician-reported |
|  |  |  |  |  |  |  | Physiological/clinical | Nervous system outcome | Phantom limb pain | Groningen questionnaire (phantom limb pain and chronic stump pain) | Patient-reported |
|  |  |  |  |  |  |  | Physiological/clinical | Nervous system outcome | Residual limb pain | Groningen questionnaire (chronic stump pain) | Patient-reported |
|  |  |  |  |  |  |  | Physiological/clinical | Infection and infestation outcomes | Infection | Rate of surgical site infection | Clinician-reported |
|  |  |  |  |  |  |  | Resource use | Hospital | Operative success | Rate of successful identification of nerve and perineural catheter placement | Clinician-reported |
| 123. | Hebenton, et al. [78] | RCS | 155F, 488M  (67 ± 13) | PAD and diabetes: 404  PAD: 239  (NA) | TTA: 645  (UL: 643) | Rehabilitation | Life impact | Delivery of care | Time to receive services | Days to compression therapy  Days to early walking aid  Days to prosthetic delivery  Days to casting | Clinician-reported |
|  |  |  |  |  |  |  | Resource use | Hospital | Discharge | Days to outpatient, inpatient discharge | Clinician-reported |
|  |  |  |  |  |  |  | Resource use | Need for further intervention | Special provisions | Service provision by vascular center (immediate postoperative rigid dressing, specialist physiotherapist in first 14 days, daily inpatient gym, inpatient gym session ≥1-hour, prosthetic center on site when inpatient, prosthetic provision as inpatient, specialist physiotherapy outpatient service) | Clinician-reported |
| 124. | Majstorović, et al. [90] | RCS | 20F, 55M  (63.5 ± 9) | PVD as late complication of diabetes mellitus and peripheral occlusive arterial disease: 75  (NA) | TTA: 75  (UL: 75) | Rehabilitation | Life impact | Delivery of care | Time to receive services | Duration from amputation to prosthesis mounting  Duration from amputation to rehabilitation | Clinician-reported |
|  |  |  |  |  |  |  | Life impact | Physical functioning | Locomotion ability | Locomotor capability index | Patient-reported |
|  |  |  |  |  |  |  | Life impact | Physical functioning | Mobility potential | K-level mobility assessment | Clinician-reported |
| 125. | Andersen, et al. [50] | RCS | 652F, 1388  (63.6 ± 12.4) | Ischemic tissue loss: 1007  Ischemic rest pain: 116  Acute ischemia: 110  Uncontrolled infection: 704 Neuropathic tissue loss: 259  Other: 44  (NA) | TTA: 2040  (UL: 2040) | Acute care, rehabilitation | Physiological/clinical | General outcomes | Frailty | 5-item modified frailty index | Clinician-reported |
|  |  |  |  |  |  |  | Adverse events | Adverse events/effects | Complications | Overall complications (myocardial infarction, stroke, congestive heart failure, skin and soft tissue infection, reperfusion symptoms, or unplanned re-operation) | Clinician-reported |
|  |  |  |  |  |  |  | Death | Mortality/survival | Mortality | Mortality (30-day) | Clinician-reported |
|  |  |  |  |  |  |  | Physiological/clinical | Infection and infestation outcomes | Infection | Surgical site infection | Clinician-reported |
|  |  |  |  |  |  |  | Resource use | Hospital | Operative outcome | Return to operation | Clinician-reported |
|  |  |  |  |  |  |  | Adverse events | Adverse events/effects | Re-amputation/revision | Revision to higher level amputation | Clinician-reported |
|  |  |  |  |  |  |  | Resource use | Hospital | Discharge | Discharge status (other than home/rehabilitation) | Clinician-reported |
|  |  |  |  |  |  |  | Life impact | Physical functioning | Prosthesis use | Failure to long-term prosthetic use | Clinician-reported |
| 126. | Christiansen, et al. [196] | Randomized crossover design | Group 1: 16M  (67.9 ± 6.2)  Group 2: 15M  (63.4 ± 8.9) | Group 1: Non-traumatic vascular etiology: 16  (3 ± 3.4)  Group 2: Non-traumatic vascular etiology: 15  (3 ± 1.3) | Group 1: TTA: 14  TFA: 2  (UL: 16)  Group 2: TTA: 12  TFA: 3  (UL: 15) | Community | Life impact | Physical functioning | Impact of disease/condition on ADL | Late life function and disability index-disability scale (frequency, limitation) | Patient-reported |
|  |  |  |  |  |  |  | Life impact | Physical functioning | Gait: spatiotemporal walking | Free living physical activity-accelerometer based average daily step count  Gait speed  2MWT | Performance-related |
|  |  |  |  |  |  |  | Life impact | Emotional functioning/well being | Motivation | Intrinsic motivation inventory-interest and enjoyment scale | Patient-reported |
|  |  |  |  |  |  |  | Life impact | Physical functioning | Mobility | TUG | Performance-related |
|  |  |  |  |  |  |  | Physiological/clinical | Psychiatric outcomes | Behavior change | Exercise stages of change scale | Patient-reported |
|  |  |  |  |  |  |  | Life impact | Physical functioning | Prosthesis-related mobility | PEQ-mobility subscale | Patient-reported |
|  |  |  |  |  |  |  | Life impact | Physical functioning | Balance confidence | Falls efficacy scale-Internation | Patient-reported |
|  |  |  |  |  |  |  | Resource use | Societal/carer burden | Social support | Multidimensional scale of perceived social support | Patient-reported |
|  |  |  |  |  |  |  | Life impact | Emotional functioning/wellbeing | Self-efficacy | Self-efficacy of exercise scale | Patient-reported |
|  |  |  |  |  |  |  | Adverse events | Adverse events/effects | Adverse events | Adverse and serious events (falls, wounds) | Patient-reported |
|  |  |  |  |  |  |  | Life impact | Delivery of care | Feasibility | Participant retention rate | Clinician-reported |
|  |  |  |  |  |  |  | Life impact | Delivery of care | Fidelity | Dose attainment goal | Clinician-reported |
| 127. | Nieuwstraten, et al. [97] | RCS | Amputee: 1F, 8M  (67.9 ± 16.1)  Caregivers: 7F  (59.6 ± 5.3)  Healthcare professional: 4F, 3M  (41.7 ± 11) | Amputee: Atherosclerosis: 6  Diabetic foot: 3  (0.8 ± 0.25) | Amputee: TTA: 8  TFA: 1  (UL: 9) | Rehabilitation | Life impact | Delivery of care | Patient satisfaction | Semi-structured interviews for patients (assessing course of disease and treatment, moment amputation discusses 1^st^ time and impact on life, decision making process to amputate, what is important in life, limitations due to disease, doctor’s enquiry about goals, information about amputation, rehabilitation, quality of life after amputation and best moment to talk about amputation) | Patient-reported |
|  |  |  |  |  |  |  | Life impact | Delivery of care | Caregiver satisfaction | Semi-structured interviews for caregivers (assessing course of disease and treatment, moment amputation discusses 1^st^ time and impact on life, decision making process to amputate, what is important in life, limitations due to disease, doctor’s enquiry about goals, information about amputation, rehabilitation, quality of life after amputation and best moment to talk about amputation) | Patient-reported |
|  |  |  |  |  |  |  | Life impact | Delivery of care | Clinician satisfaction | Semi-structured interviews for healthcare professional (assessing typical disease process and treatment, when amputation is offered as treatment, communication with patient, when do you see amputation as realistic option, advantages/disadvantages and most important reason for amputation, information about amputation and rehabilitation, important in life for these patients, inquiring after goals, values and preferences, suggestions for improving communication, quality of life after amputation | Clinician-reported |
| 128. | Kizilkurt, et al. [140] | Cross-sectional study | 27F, 38M  (57.8 ± 7.6) | Diabetic foot ulcer: 65  (NA) | TTA: 48  TFA: 17  (UL: 65) | Rehabilitation | Life impact | Global QOL | QOL | SF-36 | Patient-reported |
|  |  |  |  |  |  |  | Life impact | Global QOL | Prosthesis-related QOL | Trinity amputation and prosthesis experience scale | Patient-reported |
|  |  |  |  |  |  |  | Life impact | Emotional functioning/well-being | Coping ability | Coping attitudes evaluation scale | Patient-reported |
|  |  |  |  |  |  |  | Resource use | Societal/carer burden | Social support | Multidimensional scale of perceived social support | Patient-reported |
|  |  |  |  |  |  |  | Life impact | Emotional functioning/well-being | Self-esteem | Rosenberg self-esteem scale | Patient-reported |
|  |  |  |  |  |  |  | Life impact | Emotional functioning/well-being | Body image | Amputee body image scale | Patient-reported |
|  |  |  |  |  |  |  | Physiological/ clinical | Psychiatric outcomes | Anxiety | General anxiety disorder-7 | Patient-reported |
|  |  |  |  |  |  |  | Physiological/ clinical | Nervous system outcome | Phantom limb pain | Phantom pain | Patient-reported |
|  |  |  |  |  |  |  | Physiological/ clinical | Nervous system outcome | Residual limb pain | Stump pain | Patient-reported |
|  |  |  |  |  |  |  | Physiological/ clinical | Psychiatric outcomes | Depression | PHQ-9 | Patient-reported |
|  |  |  |  |  |  |  | Life impact | Physical functioning | Prosthesis use | Duration of prosthesis use (years)  Type of prosthesis used | Patient-reported |
| 129. | Hurwitz, et al. [79] | RCS | White: 41F, 4315M  (67.6 ± 10.1)  Black: 25F, 2277M  (67.2 ± 10.9)  Hispanic: 5F, 633M  (71.5 ± 11.5) | White:  Diabetes and/or PAD: 4356  (NA)  Black:  Diabetes and/or PAD: 2302  (NA)  Hispanic:  Diabetes and/or PAD: 638  (NA) | White:  TMT: 940  TTA: 2060  TFA: 1356  (NA)  Black:  TMT: 475  TTA: 1017  TFA: 810  (NA)  Hispanic:  TMT: 120  TTA: 225  TFA: 293  (NA) | Acute care | Physiological/clinical | General outcome | Amputation | Incident dysvascular amputation level (according to geographical regions, race/ethnicity)  Odds of TFA as compared to TTA or TMT | Clinician-reported |
| 130. | Wied, et al. [128] | RCS | 42F, 39M  (76.8 ± 11.2) | Diabetes: 22  Atherosclerosis: 52  Other: 7  (NA) | TFA: 81  (UL: 81) | Acute care | Physiological/clinical | Blood and lymphatic system outcomes | Blood loss | Total blood loss (Based on difference between pre-operative and 4^th^ post-operative day hemoglobin, and estimated blood volume) | Clinician-reported |
|  |  |  |  |  |  |  | Physiological/clinical | Blood and lymphatic system outcomes | Blood transfusion | Red blood cell transfusion | Clinician-reported |
|  |  |  |  |  |  |  | Resource use | Hospital | Duration of operation | Duration of surgery (minutes) | Clinician-reported |
|  |  |  |  |  |  |  | Resource use | Need for further intervention | Use of medication | Use of medications (non-steroidal anti-inflammatory drugs, acetylsalicylic acid, clopidogrel) | Clinician-reported |
|  |  |  |  |  |  |  | Resource use | Need for further intervention | Specialist consultation | Operator (surgeon/senior consultant) | Clinician-reported |
| 131. | Luetmer, et al. [89] | RCS | Dysvascular: TFA: 43F, 39M  (76.4 ± 11.2)  Non-TFA: 430F, 410M  (76.4 ± 11.3)  Trauma/cancer: TFA: 3F, 9M  (48.6 ± 20)  Non-TFA: 30F, 90M  (48.6 ± 20.8) | Dysvascular: 924  (NA)  Trauma or cancer: 132  (NA) | Dysvascular: TFA: 84  Non-TFA: 840  (NA)  Trauma/cancer: TFA: 12  Non-TFA: 120  (NA) | Community | Physiological/ clinical | Nervous system outcomes | Phantom limb pain | % with phantom limb pain | Clinician-reported |
|  |  |  |  |  |  |  | Death | Mortality/survival | Mortality | Mortality (mean years) after index amputation | Clinician-reported |
|  |  |  |  |  |  |  | Death | Mortality/survival | Mortality | Mortality rate (1-year, 5-year) | Clinician-reported |
|  |  |  |  |  |  |  | Physiological/clinical | General outcomes | Comorbidities | Charlson comorbidity index | Clinician-reported |
|  |  |  |  |  |  |  | Physiological/ clinical | Nervous system outcomes | Low back pain | Number of low back pain events (prior and after the index amputation), age at 1^st^ low back pain event | Clinician-reported |
|  |  |  |  |  |  |  | Life impact | Physical functioning | Prosthesis use | % received prosthesis (receipt) | Clinician-reported |
| 132. | Fard, et al. [68] | RCS | 235F, 247M  (71.9 ± 12.5) | Diabetes and/or PAD: 375  Acute arterial occlusion: 7  (NA) | TTA: 176  TKA: 25  TFA: 179  (UL: 365, BL: 17) | Acute care | Death | Mortality/survival | Mortality | Mortality (30-day)  Mortality (1-year) | Clinician-reported |
|  |  |  |  |  |  |  | Death | Mortality/survival | Survival | Survival duration (years) | Clinician-reported |
|  |  |  |  |  |  |  | Adverse events | Adverse events/effects | Re-amputation/revision | Ipsilateral re-amputation (1-year) | Clinician-reported |
| 133. | Mundell, et al. [94] | RCS | 68F, 94M  (56 ± 25.4)  Controls: 1620 (?) | Dysvascular: 107  Trauma/cancer: 39  (NA) | TFA: 162  (NA) | Acute care | Death | Mortality/survival | Mortality | Mortality rate (1-year)  Mortality rate (5-year) | Clinician-reported |
|  |  |  |  |  |  |  | Adverse events | Adverse events/effects | Adverse events | Major adverse cardiovascular events | Clinician-reported |
|  |  |  |  |  |  |  | Life impact | Physical functioning | Prosthesis use | Received prosthesis (influence on mortality, major adverse cardiovascular events) | Clinician-reported |
| 134. | Chang, et al. [56] | RCS | Negative pressure wound management: 9F, 14M  (67 ± 12)  Standard wound management: 14F, 17M  (73 ± 15) | Negative pressure wound management: Ischemic tissue loss: 18  Ischemic uncontrolled infection: 3  Rest pain: 2  (NA)  Standard wound management: Ischemic tissue loss: 23  Ischemic uncontrolled infection: 2  Rest pain: 6  (NA) | Negative pressure wound management: TTA: 12  TFA: 11  (UL: 23)  Standard wound management:  TTA: 17  TFA: 14  (UL: 31) | Acute care | Adverse events | Adverse events/effects | Complications | Post-operative complications following major amputation within 30-day (deep and superficial surgical site infection, necrosis, dehiscence, hematoma, seroma) | Clinician-reported |
|  |  |  |  |  |  |  | Death | Mortality/survival | Mortality | Mortality (30-day) | Clinician-reported |
|  |  |  |  |  |  |  | Adverse events | Adverse events/effects | Re-amputation/revision | Revision (30-day) | Clinician-reported |
|  |  |  |  |  |  |  | Resource use | Hospital | Re-admission | Re-admission rate (30-day) | Clinician-reported |
|  |  |  |  |  |  |  | Resource use | Hospital | Duration of stay | Length of post-operative stay (days) | Clinician-reported |
|  |  |  |  |  |  |  | Physiological/ clinical | Skin and subcutaneous tissue outcomes | Stump wound healing | Wound classification (clean, clean-contaminated, contaminated, dirty) | Clinician-reported |
|  |  |  |  |  |  |  | Resource use | Hospital | Admission | Admission to emergency care | Clinician-reported |
|  |  |  |  |  |  |  | Physiological/clinical | Blood and lymphatic system outcomes | Blood transfusion | Mean perioperative blood transfusion (units) | Clinician-reported |
|  |  |  |  |  |  |  | Physiological/clinical | Blood and lymphatic system outcomes | Blood loss | Mean blood loss (ml) | Clinician-reported |
| 135. | Gwilym, et al. [174] | PCS | 105F, 432M  (68, 19 to 94) | Chronic limb ischemia and/or diabetes: 537  (NA) | TTA: 271  TKA: 17  TFA: 249  (UL: 537) | Acute care | Resource use | Need for further interventions | Predicting outcome | Estimation of likely outcomes of surgery by the healthcare professionals (surgeons: consultants and registrar level trainees regarding % likelihood prediction of death, morbidity and revision within 30-day) (anesthetists: consultants and registrar level trainees regarding morbidity and mortality at 30-day using visual analogue scale (0 to 100%)  Healthcare professionals using existing risk prediction tools | Clinician-reported |
|  |  |  |  |  |  |  | Death | Mortality/survival | Mortality | Mortality (30-day) | Clinician-reported |
|  |  |  |  |  |  |  | Adverse events | Adverse events/effects | Re-amputation/revision | Revision of major lower limb amputation | Clinician-reported |
|  |  |  |  |  |  |  | Physiological/clinical | Infection and infestation outcomes | Infection | Surgical site infection  COVID-19 infection | Clinician-reported |
|  |  |  |  |  |  |  | Physiological/clinical | Blood and lymphatic system outcomes | Blood transfusion | Blood transfusion | Clinician-reported |
| 136. | Silva, et al. [117] | RCS | Staged: 39F, 62M  (63.8 ± 10.5)  One-staged: 45F, 61M  (64.1 ± 10.7) | PAD and critical limb ischemia: 185  (NA) | Foot:14  Malleolar:7  TTA:98  KDA:6  TFA:82  (UL:185, BL:22) | Acute care | Resource use | Hospital | Operative success | Operative success | Clinician-reported |
|  |  |  |  |  |  |  | Resource use | Hospital | Duration of stay | Length of hospital stay (days) | Clinician-reported |
|  |  |  |  |  |  |  | Death | Mortality/survival | Mortality | Mortality (operative)  Mortality (30-day)  Mortality (1-year) | Clinician-reported |
|  |  |  |  |  |  |  | Physiological/clinical | Infection and infestation outcomes | Infection | Presence of infection | Clinician-reported |
| 137. | Alfawaz, et al. [46] | RCS | 39F, 96M  (61.5 ±11.9) | Atraumatic vasculopathies: 135  (NA) | TTA: 135  (UL: 135) | Acute care | Life impact | Physical functioning | Prosthesis fit | Fit with prosthesis | Patient-reported |
|  |  |  |  |  |  |  | Physiological/clinical | Vascular outcomes | Radiology | Pre-operative angiography to demonstrate vessel run-off | Clinician-reported |
|  |  |  |  |  |  |  | Adverse events | Adverse events/effects | Complications | Post-operative complications (hematoma, dehiscence, non-healing stump, chronic infection/osteomyelitis | Clinician-reported |
|  |  |  |  |  |  |  | Life impact | Physical functioning | Locomotor ability | Duration to ambulation (days)  Post-operative ambulatory status (non-ambulatory, wheelchair, outdoor ambulation) | Clinician-reported |
|  |  |  |  |  |  |  | Adverse events | Adverse events/effects | Re-amputation/revision | Mean time to TFA progression | Clinician-reported |
|  |  |  |  |  |  |  | Physiological/ clinical | Skin and subcutaneous tissue outcomes | Stump wound healing | Duration to healing (days) | Clinician-reported |
|  |  |  |  |  |  |  | Life impact | Delivery of care | Time to receive services | Duration to revision (days) | Clinician-reported |
|  |  |  |  |  |  |  | Resource use | Hospital | Discharge | Duration to home discharge (days) | Clinician-reported |
| 138. | Yaqoob, et al. [154] | Cross-sectional study | 39F, 261M  (18-67) | Diabetes: 51  PVD:10  Trauma: 184  Others: 55  (NA) | Toe: 6  Transmetatarsal: 7  Lisfrance: 2  Chopart: 10  Syme: 16  TTA: 259  (UL: 300) | Community | Life impact | Global QOL | QOL | World Health Organization quality of life-BREF | Patient-reported |
|  |  |  |  |  |  |  | Life impact | Physical functioning | Mobility potential | K-level classification | Clinician-reported |
| 139. | Ner, et al. [95] | RCS | 19F,53M  (72 ±10) | Diabetes and ischemia : 53  Diabetes : 9  Ischemia: 10  (NA) | TTA:31  TFA:41  (UL: 72) | Acute care | Physiological/clinical | General outcomes | Comorbidities | Comorbidities  Age-adjusted Charlson comorbidity index | Clinician-reported |
|  |  |  |  |  |  |  | Death | Mortality/survival | Mortality | Mortality (30-day)  Mortality (180-day)  Mortality (1-year)  Mortality (2-year) | Clinician-reported |
|  |  |  |  |  |  |  | Life impact | Physical functioning | Functional independence | Pre-amputation functional status (excursion: bed ridden, assisted transfer to chair, limited to hoe, outside with limitations, outside without limitations; ambulation: bed ridden, wheel chair, crutches/walkway, walking stick) | Clinician-reported |
|  |  |  |  |  |  |  | Death | Mortality/survival | Survival | Duration of survival (months) | Clinician-reported |
| 140. | de Boer, et al. [157] | Case study | 1F  (60) | Ischemia and non-healing wound: 1  (NA) | TFA: 1  (UL:1) | Acute care, rehabilitation | Life impact | Physical functioning | Prosthesis fit | Fit of prosthesis | Patient-reported |
|  |  |  |  |  |  |  | Physiological/clinical | Nervous system outcome | General pain | Pain visual analogue scale (0 to 10) | Patient-reported |
|  |  |  |  |  |  |  | Resource use | Need for further intervention | Specialist consultation | Consultation with vascular surgeon, neurologist | Clinician-reported |
|  |  |  |  |  |  |  | Physiological/clinical | Musculoskeletal and connective tissue outcomes | Radiology | X-ray | Clinician-reported |
|  |  |  |  |  |  |  | Physiological/ clinical | Vascular outcomes | Radiology | Computed tomography | Clinician-reported |
|  |  |  |  |  |  |  | Resource use | Need for further intervention | Use of medication | Use of medications (acetylsalicyic acid, dalteparin) | Clinician-reported |
|  |  |  |  |  |  |  | Life impact | Physical functioning | Prosthesis use | Duration of prosthesis use (minutes) | Patient-reported |
|  |  |  |  |  |  |  | Life impact | Physical functioning | Mobility | Changes in functional mobility (wheelchair, walking ability) and activities of daily living (washing, dressing, toileting) | Clinician-reported |
| 141. | Cruz Silva, et al. [61] | RCS | 28F,78M  (72.1 ±10.8) | Gangrene: 9  Critical Limb threatening Ischemia: 91  (NA) | TTA:41  TFA: 78  (UL: 93, BL: 13) | Acute care | Life impact | Global QOL | QOL | Vascular amputee adapted quality of life questionnaire adapted from World Health Organization quality of life-BREF and SF36 questionnaires | Patient-reported |
|  |  |  |  |  |  |  | Life impact | Physical functioning | Functional independence | Modified Barthel index | Clinician- reported |
|  |  |  |  |  |  |  | Death | Mortality/survival | Mortality | Mortality (30-day)  Mortality (1-year) | Clinician-reported |
|  |  |  |  |  |  |  | Life impact | Physical functioning | Prosthesis use | Use of prosthesis | Patient-reported |
|  |  |  |  |  |  |  | Life impact | Delivery of care | Time to receive services | Waiting for prosthesis | Patient-reported |
|  |  |  |  |  |  |  | Adverse events | Adverse events/effects | Re-amputation/revision | Re-amputation, revision surgery | Clinician-reported |
|  |  |  |  |  |  |  | Life impact | Delivery of care | Feasibility | % of participants agreeing to participate | Clinician-reported |
| 142. | Varino, et al. [122] | RCS | 298F, 259M  (86.3 ± 4.4) | Acute ischemia: 68  Chronic limb ischemia: 330  Diabetic infection: 8  Pressure lesion: 150  (NA) | TTA: 25  TFA:423  (UL: 448, BL: 109) | Acute care | Death | Mortality/survival | Mortality | Mortality (30-day)  Mortality (1-year)  Mortality (2-year)  Overall mortality  Time to death (months) | Clinician-reported |
|  |  |  |  |  |  |  | Life impact | Physical functioning | Functional independence | Functional status (bedridden) | Clinician-reported |
|  |  |  |  |  |  |  | Resource use | Hospital | Discharge | Discharged to (died before discharge, home, inpatient rehabilitation, nursing home, hospital) | Clinician-reported |
|  |  |  |  |  |  |  | Resource use | Hospital | Admission | Admitted from (home, nursing home, inpatient rehabilitation, hospital) | Clinician-reported |
| 143. | Baysal, et al. [52] | RCS | Diabetes mellitus: 17F, 88M  (63.7 ± 9.9)  PAD: 8F, 20M  (69.3 ±16) | Diabetes Mellitus: 105  PAD: 28  (NA) | Toe:15  TMT:28  TTA:58  TFA:32  (UL:133) | Acute care | Adverse events | Adverse events/effects | Re-amputation/revision | Need for re-amputation  Duration from first to second amputation | Clinician-reported |
|  |  |  |  |  |  |  | Resource use | Hospital | Admission | Postoperative ICU requirement | Clinician-reported |
|  |  |  |  |  |  |  | Resource use | Hospital | Duration of stay | Length of stay in hospital  Length of stay in ICU | Clinician-reported |
|  |  |  |  |  |  |  | Death | Mortality/survival | Survival | Survival status (survived/deceased) | Clinician-reported |
| 144. | Collins, et al. [59] | RCS | 20F, 73M  70 (30-89) | PVD: 53  Diabetes mellitus: 15  PVD and Diabetes mellitus: 16  Trauma: 6  Malignancy: 3  (NA) | TTA: 60  TFA: 32  Hemipelvectomy: 1  (UL: 93) | Rehabilitation | Life impact | Physical functioning | Locomotion ability | Locomotor capability index-5 | Performance-related |
|  |  |  |  |  |  |  | Life impact | Physical functioning | Mobility | Specialist Interest Group in Amputee Medicine scale  Pre-amputation mobility as per Taylor, et al. [206] | Performance-related |
|  |  |  |  |  |  |  | Life impact | Physical functioning | Gait: spatiotemporal walking | 10-meter timed walk Test | Performance-related |
| 145. | Miller, et al. [144] | Cross-sectional study | 13F, 108M  (62.5 ± 8) | Dysvascular: 100  Trauma: 22  (4.9 ± 4.8) | TTA: 83  TFA: 25  (UL: 108,  BL: 14) | Community | Life impact | Emotional functioning/well being | Coping ability | Connor-Davidson resilience questionnaire-25 and 10 | Patient-reported |
|  |  |  |  |  |  |  | Life impact | Physical functioning | Prosthesis-related mobility | PEQ-mobility subscale | Patient-reported |
|  |  |  |  |  |  |  | Life impact | Physical functioning | Impact of disease/condition on ADL | 12-itemWorld Health Organization disability assessment schedule | Patient-reported |
|  |  |  |  |  |  |  | Physiological/clinical | General outcomes | Comorbidities | Functional comorbidity index | Patient-reported |
|  |  |  |  |  |  |  | Life impact | Physical functioning | Balance confidence | Fall efficacy scale-International | Patient-reported |
|  |  |  |  |  |  |  | Life impact | Physical functioning | Mobility | Life space assessment questionnaire | Patient-reported |
|  |  |  |  |  |  |  | Physiological/ clinical | Psychiatric outcomes | Depression | Depression (Hospital anxiety and depression scale) | Patient-reported |
|  |  |  |  |  |  |  | Physiological/ clinical | Psychiatric outcomes | Anxiety | Anxiety (Hospital anxiety and depression scale) | Patient-reported |
|  |  |  |  |  |  |  | Life impact | Emotional functioning/wellbeing | Self-efficacy | Self-efficacy of managing chronic disease questionnaire | Patient-reported |
|  |  |  |  |  |  |  | Resource use | Societal/carer burden | Social support | Multidimensional Scale of Perceived Social Support | Patient-reported |
| 146. | Ratliff [149] | Cross-sectional study | 6F,11M  66 (44-82) | PAD: 17  (NA) | TTA: 11  TFA: 6  (UL. 17) | Acute care | Physiological/clinical | Injury and poisoning outcomes | Pressure ulcers | Braden scale  Pressure injuries present on admission and acquired at hospital (stage 1 to 4) | Clinician-reported |
| 147. | Wied, et al. [126] | RCS | Survivor: 74F, 90M  (74 ± 11)  Non survivor: 12F, 19M  (78 ± 13) | Survivor:  Dysvascular: 164  (NA)  Non-survivor:  Dysvascular: 31  (NA) | Survivor:  TTA: 71  TKA: 5  TFA: 88  (UL: 164)  Non-survivor:  TTA: 9  TKA: 0  TFA: 22  (UL: 31) | Acute care | Death | Mortality/survival | Mortality | Mortality (30-day)  Postoperative days to death | Clinician-reported |
|  |  |  |  |  |  |  | Death | Mortality/survival | Cause of mortality | Estimated cause of mortality (stroke, acute myocardial infarction, pneumonia or respiratory failure, sepsis, renal failure, gastrointestinal ileus or hemorrhage) | Clinician-reported |
|  |  |  |  |  |  |  | Resource use | Hospital | Operative outcome | Failure to rescue score with active care curtailed (yes/no) | Clinician-reported |
| 148. | Madsen, et al. [177] | PCS | 15F, 43M  (69 ± 11) | Diabetes: 24  PAD: 34  (NA) | TTA: 23  TFA: 35  (UL: 49, BL: 9) | Acute care | Life impact | Global QOL | QOL | SF-36 | Patient-reported |
|  |  |  |  |  |  |  | Life impact | Emotional functioning/wellbeing | Self-efficacy | General self-efficacy scale | Patient-reported |
|  |  |  |  |  |  |  | Life impact | Physical functioning | Functional independence | Barthel index | Clinician-reported |
| 149. | Endoh, et al. [134] | RCS | 5080F, 8694M  (72.4 ± 13.4) | PVD: 7231  Not reported: 6543  (NA) | Foot: 2148  TTA: 5631  TFA: 5995  (UL: 13774) | Acute care | Physiological/clinical | General outcomes | Comorbidities | Charlson comorbidity index | Clinician-reported |
|  |  |  |  |  |  |  | Death | Mortality/survival | Mortality | In-hospital mortality rate | Clinician-reported |
|  |  |  |  |  |  |  | Resource use | Hospital | Discharge | Discharge (home, facility or another hospital) | Clinician-reported |
|  |  |  |  |  |  |  | Adverse events | Adverse events/effects | Re-amputation/revision | Re-amputation rate during hospitalization | Clinician-reported |
| 150. | Gabel, et al. [72] | RCS | 1046F, 1893M  (66) | Ischemic rest pain: 202  Ischemic tissue loss: 1464  Acute ischemia: 223  Uncontrolled infection: 842  Neuropathic tissue loss: 64  (NA) | TTA: 1656  TFA: 1283  (NA) | Acute care | Death | Mortality/survival | Mortality | Mortality (30-day) | Clinician-reported |
|  |  |  |  |  |  |  | Adverse events | Adverse events/effects | Re-amputation/revision | Revision to a higher level | Clinician-reported |
|  |  |  |  |  |  |  | Resource use | Need for further intervention | Reoperation | Re-operation (bleeding, infection, revision) | Clinician-reported |
|  |  |  |  |  |  |  | Adverse events | Adverse events/effects | Complications | Post-operative complication (pneumonia/ventilator, renal complication, myocardial infarction, infection) | Clinician-reported |
|  |  |  |  |  |  |  | Physiological/clinical | Blood and lymphatic system outcomes | Blood loss | Estimated blood loss (<250 ml, >250 ml) | Clinician-reported |
|  |  |  |  |  |  |  | Resource use | Hospital | Discharge | Discharge status (home, rehabilitation unit, nursing home, dead, other hospital) | Clinician-reported |
|  |  |  |  |  |  |  | Life impact | Physical functioning | Locomotion ability | Ambulatory status at discharge (ambulatory, ambulatory with assistance, wheelchair, bedridden) | Clinician-reported |
|  |  |  |  |  |  |  | Life impact | Physical functioning | Impact of disease/condition on ADL | Negative changes in ambulatory status | Clinician-reported |
| 151. | Langnas, et al. [165] | Case study | 1M  (74) | Critical limb ischemia: 1  (0.08) | TTA: 1  (UL: 1) | Acute care | Resource use | Need for further intervention | Use of medication | Use of medications (heparin, propofol, fentanyl, rocuronium, lidocaine, cefazolin, sugammadex, ondasetron, acetaminophen, oxycodone, hydromorphone) | Clinician-reported |
|  |  |  |  |  |  |  | Physiological/clinical | Blood and lymphatic system outcomes | Laboratory parameters | Partial thromboplastin time | Clinician-reported |
|  |  |  |  |  |  |  | Resource use | Need for further intervention | Use of anesthesia | Use of general anesthesia  Use of nerve block (erector spinae plane) | Clinician-reported |
|  |  |  |  |  |  |  | Physiological/clinical | Nervous system outcome | General pain | Numerical rating scale for pain (0 to 10) | Patient-reported |
|  |  |  |  |  |  |  | Life impact | Delivery of care | Patient experience | Subjective interview regarding experience with nerve block | Patient-reported |
| 152. | De Klerk, et al. [172] | PCS | 95F, 129M  (64, 24 to 93) | PVD: 224  (NA) | TTA: 119  TFA: 105  (UL: 224) | Acute care | Death | Mortality/survival | Mortality | Cumulative mortality (2-year) | Clinician-reported |
|  |  |  |  |  |  |  | Death | Mortality/survival | Cause of mortality | Possible cause of mortality | Clinician-reported |
|  |  |  |  |  |  |  | Life impact | Delivery of care | Feasibility | Response rate of the participants | Patient-reported |
|  |  |  |  |  |  |  | Life impact | Physical functioning | Mobility | Interviews regarding mobility (walking with prosthesis, crutch, bedridden, wheelchair) | Patient-reported |
|  |  |  |  |  |  |  | Life impact | Personal circumstances | Employment | Change in employment | Patient-reported |
| 153. | Dillingham, et al. [202] | Quasi-experimental (pre- vs. post-test) | 4F, 22M (50 ± 10.2) | Diabetes/vascular disease: 16  Trauma: 10  (NA) | TTA: 26  (NA) | Rehabilitation | Life impact | Global QOL | Prosthesis-related QOL | PEQ | Patient-reported |
|  |  |  |  |  |  |  | Life impact | Physical functioning | Gait: spatiotemporal walking | Gait speed  Step length  Limp index  Stance characteristics using Vicon motion analysis | Performance-related |
|  |  |  |  |  |  |  | Life impact | Physical functioning | Prosthesis use | Peak intra socket pressure analysis (7 to 28 psi using Fujifilm pre-scale film) | Clinician-reported |
| 154. | Kennedy, et al. [84] | RCS | 27F, 57M  (72, 34 to 99) | PVD and/or diabetes: 79  (1.4, 0 to 3.2) | TTA/TFA: 79  (UL: 59, BL: 20) | Acute care | Death | Mortality/survival | Mortality | Mortality (30-day)  Mortality (6-month)  Mortality (1-year)  Mortality (2-year,)  Mortality (3-year)  Mean age at the time of death | Clinician-reported |
|  |  |  |  |  |  |  | Resource use | Need for further intervention | Use of medication | Use of medications (anticoagulants, antiplatelet)  Indication for anticoagulant usage (indication unclear, atrial fibrillation, left ventricular thrombus, post-operative thrombus, post-operative deep vein thrombosis, post-operative pulmonary embolus, recurrent deep vein thrombosis) | Clinician-reported |
| 155. | Miller, et al. [200] | Cross-sectional study (secondary analysis) | 3F, 55M  (64.4 ± 9) | Diabetes and/or PAD: 58  (NA) | TTA: 55  TKA/TFA: 3  (NA) | Rehabilitation | Life impact | Physical functioning | Prosthesis-related-mobility | Prosthesis evaluation questionnaire-mobility subscale | Patient-reported |
|  |  |  |  |  |  |  | Life impact | Physical functioning | Gait: spatiotemporal walking | 2MWT  5-meter walk test  Average daily step count | Performance -related |
|  |  |  |  |  |  |  | Resource use | Need for further interventions | Mobility aid | Use of assistive device | Clinician-reported |
|  |  |  |  |  |  |  | Life impact | Physical functioning | Mobility | TUG | Performance-related |
|  |  |  |  |  |  |  | Life impact | Physical functioning | Impact of disease/condition on ADL | Pain with gait assessed by a numerical rating scale (0 to 10) | Patient-reported |
|  |  |  |  |  |  |  | Life impact | Physical functioning | Physical activity | Accelerometer wear time (minutes) for assessing physical activity | Performance-related |
|  |  |  |  |  |  |  | Life impact | Cognitive functioning | Cognitive function | Folstein mental state examination version 1 | Patient-reported |
|  |  |  |  |  |  |  | Physiological/ clinical | Psychiatric outcomes | Depression | 15-item Geriatric depression scale | Patient-reported |
|  |  |  |  |  |  |  | Physiological/ clinical | Skin and subcutaneous tissue outcomes | Stump wound healing | Chakrabarty scale determined residual limb quality and presence of wound | Clinician-reported |
|  |  |  |  |  |  |  | Physiological/clinical | General outcomes | Comorbidities | Functional comorbidity index | Clinician-reported |
|  |  |  |  |  |  |  | Life impact | Physical functioning | Gait: spatiotemporal walking | Walking time prior to lower limb amputation (minutes) | Patient-reported |
|  |  |  |  |  |  |  | Physiological/ clinical | Skin and subcutaneous tissue outcomes | Stump wound healing | Presence of residual limb wound | Clinician-reported |
|  |  |  |  |  |  |  | Physiological/clinical | Psychiatric outcomes | Addiction outcomes | Prior smoking history | Patient-reported |
| 156. | Sjödin, et al. [119] | RCS | 109F, 53M  (85.1, 54 to 100) | PAD: 126  Other: 36 | TFA: 162  (UL: 162) | Acute care | Death | Mortality/survival | Mortality | Mortality (1-week)  Mortality (1-year) | Clinician-reported |
| 157. | Orłowski, et al. [103] | RCS | 38F, 54M  (70 ± 9.3) | Ischemia due to PAD: 92  (NA) | TFA: 92  (NA) | Acute care | Physiological/ clinical | Skin and subcutaneous tissue outcomes | Stump wound healing | Time from amputation to healing (days) | Clinician-reported |
|  |  |  |  |  |  |  | Adverse events | Adverse events/effects | Complications | Necrosis % | Clinician-reported |
|  |  |  |  |  |  |  | Resource use | Hospital | Duration of stay | Duration of hospital stay (days) | Clinician-reported |
|  |  |  |  |  |  |  | Physiological/clinical | Blood and lymphatic system outcomes | Laboratory parameters | Laboratory values hematocrit %  hemoglobin | Clinician-reported |
|  |  |  |  |  |  |  | Physiological/clinical | Blood and lymphatic system outcomes | Blood transfusion | Need of transfusion % | Clinician-reported |
|  |  |  |  |  |  |  | Physiological/clinical | Blood and lymphatic system outcomes | Blood loss | Bleeding (very good, good, medium, minimal) | Clinician-reported |
|  |  |  |  |  |  |  | Resource use | Need for further intervention | Use of anesthesia | American society of anesthesiologist’s scale  Need for anesthesia (conduction, general) | Clinician-reported |
|  |  |  |  |  |  |  | Resource use | Hospital | Duration of operation | Duration of operation (minutes) | Clinician-reported |
|  |  |  |  |  |  |  | Resource use | Need for further intervention | Specialist consultation | Operator (resident/ specialist) | Clinician-reported |
|  |  |  |  |  |  |  | Resource use | Need for further intervention | Use of medication | Use of medications (non-steroidal anti-inflammatory drugs | Clinician-reported |
|  |  |  |  |  |  |  | Physiological/clinical | Vascular outcomes | Diagnostic test | Arterial flow: Biphasic, monophasic, no flow (external iliac, femoral, deep artery of thigh, superficial femoral artery) | Clinician-reported |
| 158. | Murray, et al. [146] | Cross-sectional study | Amputee: 9 (?F, ?M)  (56.9 ± 4.6)  Diabetes: 10 (?F, ?M)  (63.5 ± 8.3)  Healthy: 11 (?F, ?M)  (59.7 ± 7) | Diabetes: 9  (NA) | TTA: 9  (UL: 9) | Rehabilitation | Life impact | Physical functioning | Gait: spatiotemporal walking | Self-selected gait speed | Performance-related |
|  |  |  |  |  |  |  | Life impact | Physical functioning | Gait: kinetics | Peak internal moments with step ascent and descent during single limb support (low back: extension, lateral bend, hip: extension, abduction, knee: extension, adduction) | Performance-related |
|  |  |  |  |  |  |  | Life impact | Physical functioning | Mobility potential | K-level mobility | Clinician-reported |
| 159. | Anderson, et al. [205] | RCT (secondary data) | Faller: 30 (?F, ?M)  (63.7 ± 8.5)  Non-faller: 38 (?F, ?M)  (65.2 ± 8.6) | Faller: Dysvascular: 30  (1.1 ± 1.3)  Non-faller: Dysvascular: 38  (1.3 ± 1.4) | Faller:  TTA: 29  TFA: 2  (UL: 27, BL: 3)  Non-faller: TTA: 38  TKA: 1  (UL: 39) | Community | Life impact | Physical functioning | Mobility | TUG | Performance-related |
|  |  |  |  |  |  |  | Life impact | Physical functioning | Gait: spatiotemporal walking | 2MWT  Step count (accelerometer, steps/day)  Gait speed | Performance-related |
|  |  |  |  |  |  |  | Physiological/clinical | Injury and poisoning outcomes | Falls | Fall description (activity at time of fall transfer, sit/stand transition, reaching/stationary activity, walking on level/uneven surface, other, curbs/steps, stair ascent/descent; prosthesis status: on, off, fell off, unknown; fall location: community, home) | Patient-reported |
| 160. | Strelec, et al. [168] | Case study | 1M  (72) | PAD: 1  (0.17) | TFA: 2  (BL: 1) | Rehabilitation | Life impact | Physical functioning | ROM | Active range of motion (shoulder, elbow, wrist)  Passive range of motion (shoulder, elbow, wrist) | Performance-related |
|  |  |  |  |  |  |  | Physiological/clinical | Nervous system outcomes | Sensation | Sensation in lower limbs | Performance-related |
|  |  |  |  |  |  |  | Life impact | Physical functioning | Strength | Strength (trunk) | Performance-related |
|  |  |  |  |  |  |  | Life impact | Physical functioning | Impact of disease/condition on ADLs | Activity limitations (bed mobility, sitting, standing balance transfer, standing tolerance, gait) | Clinician-reported |
|  |  |  |  |  |  |  | Resource use | Need for further intervention | Rehabilitation | Duration of rehabilitation treatment (minutes) | Clinician-reported |
|  |  |  |  |  |  |  | Life impact | Physical functioning | Prosthesis use | Use of prosthesis | Clinician-reported |
| ABC: Activities and balance confidence scale, ADL: Activities of daily living, SF-36: Short form-36, 6MWT: 6-minute walk test, 2MWT: 2-minute walk test, TUG: Timed up and go test, ECG: Electrocardiogram, PEQ: Prosthesis evaluation questionnaire, BBS: Berg balance scale, EMG: Electromyography, PHQ-9: Patient health questionnaire, BMI: Body mass index, TMT: Transmetatarsal, TFA: Transfemoral, TTA: Transtibial | | | | | | | | | | | |

**Supplementary table 3.** Complete classification of outcome measures and indicators from the present review and from Ambler et al. (outcomes from Ambler et al. shown in red), organized according to Dodd's hierarchical framework across core areas, domains, subdomains, and measures/indicators

| **Death** | | | |  |  |
| --- | --- | --- | --- | --- | --- |
| Outcome domains | Outcome Subdomains | Outcome measures/indicators | Number of studies reporting measures/indicators | % of overall measures/indicators | Unique outcome measures/indicators |
| Mortality/survival | Cause of mortality | Cause of mortality | 1+**19** | 0.81% | 4 |
|  |  | Possible cause of mortality | 2 | 0.08% |  |
|  |  | *Cause of in-hospital death* | **1** | 0.04% |  |
|  |  | *Mortality from purulent and necrotic complications* | **1** | 0.04% |  |
|  | Survival | Duration of post-operative survival (days) | 1 | 0.04% | 21 |
|  |  | Duration of survival (months) | 1 | 0.04% |  |
|  |  | Median survival time | 1 | 0.04% |  |
|  |  | Overall survival probability | 1 | 0.04% |  |
|  |  | Survival duration (years) | 1 | 0.04% |  |
|  |  | Life years per patient | 1 | 0.04% |  |
|  |  | Survival status (survived/deceased) | 1 | 0.04% |  |
|  |  | Survival (1-month) | 1 | 0.04% |  |
|  |  | Survival rate (6-month) | 1 | 0.04% |  |
|  |  | Survival (6-month) | 1 | 0.04% |  |
|  |  | Survival rate (1-year) | 2 | 0.08% |  |
|  |  | Survival (1-year) | 4+**4** | 0.32% |  |
|  |  | Survival (2-year) | 1+**1** | 0.08% |  |
|  |  | Survival (3-year) | 1+**1** | 0.08% |  |
|  |  | Survival rate (5-year) | 1 | 0.04% |  |
|  |  | Survival (5-year) | 1**+1** | 0.08% |  |
|  |  | *Survival (2-month)* | **1** | 0.04% |  |
|  |  | *Survival* | **63** | 2.55% |  |
|  |  | *Long term patient survival* | **1** | 0.04% |  |
|  |  | *Additional amputation free survival* | **1** | 0.04% |  |
|  |  | *Contralateral amputation free survival* | **2** | 0.08% |  |
|  | Mortality | All-cause mortality | 1+**1** | 0.08% | 52 |
|  |  | All-cause mortality (1-year) | 1 | 0.04% |  |
|  |  | All-cause mortality (30-day) | 1 | 0.04% |  |
|  |  | All-cause mortality (5-year) | 1 | 0.04% |  |
|  |  | All-cause mortality (90-day) | 1 | 0.04% |  |
|  |  | Cumulative mortality (2-year) | 1 | 0.04% |  |
|  |  | Death (3 to 5 years) | 1 | 0.04% |  |
|  |  | Early <30-day mortality | 1 | 0.04% |  |
|  |  | In-hospital mortality | 4+**14** | 0.73% |  |
|  |  | Long term mortality | 1 | 0.04% |  |
|  |  | Mean age at time of death | 1 | 0.04% |  |
|  |  | Mortality | 5+**101** | 4.30% |  |
|  |  | Mortality (>30-day) | 1 | 0.04% |  |
|  |  | Mortality (≤30-day) | 1 | 0.04% |  |
|  |  | Mortality (1-week) | 1+**1** | 0.08% |  |
|  |  | Mortality (1-year) | 19+**11** | 1.22% |  |
|  |  | Mortality (2-year) | 5+**6** | 0.45% |  |
|  |  | Mortality (30-day) | 31+**46** | 3.12% |  |
|  |  | Mortality (3-year) | 1 | 0.04% |  |
|  |  | Mortality (48-hour) | 1 | 0.04% |  |
|  |  | Mortality (60-day) | 1 | 0.04% |  |
|  |  | Mortality (6-month) | 6+**6** | 0.49% |  |
|  |  | Mortality (in-patient) | 1 | 0.04% |  |
|  |  | Mortality (mean years) after index amputation | 1 | 0.04% |  |
|  |  | Mortality before (1 year) | 2 | 0.08% |  |
|  |  | Mortality before (28 days) | 1 | 0.04% |  |
|  |  | Mortality following ICU stay (1 year) | 1 | 0.04% |  |
|  |  | Mortality following ICU stay (28 days) | 1 | 0.04% |  |
|  |  | Mortality prior to contralateral major/minor lower limb amputation | 1 | 0.04% |  |
|  |  | Mortality rate (1-year) | 2 | 0.08% |  |
|  |  | Mortality rate (5-year) | 2 | 0.08% |  |
|  |  | Overall mortality | 1 | 0.04% |  |
|  |  | Overall mortality (23-month) | 1 | 0.04% |  |
|  |  | Overall mortality (30-day) | 1 | 0.04% |  |
|  |  | Postoperative days to death | 1 | 0.04% |  |
|  |  | Time to death (months) | 1 | 0.04% |  |
|  |  | Mortality (operative) | 1 | 0.04% |  |
|  |  | *Mortality (8-year)* | **1** | 0.04% |  |
|  |  | *Death after discharge* | **1** | 0.04% |  |
|  |  | *Death before discharge* | **1** | 0.04% |  |
|  |  | *Died before healing* | **1** | 0.04% |  |
|  |  | *Died within (2-year) of amputation* | **1** | 0.04% |  |
|  |  | *Early mortality rate within (14-day)* | **3** | 0.12% |  |
|  |  | *In-patient death in rehabilitation* | **2** | 0.08% |  |
|  |  | *Mortality (3-month)* | **4** | 0.16% |  |
|  |  | *Mortality (3-year)* | **2** | 0.08% |  |
|  |  | *Mortality (5-year)* | **5** | 0.20% |  |
|  |  | *Perioperative mortality* | **19** | 0.77% |  |
|  |  | *Postoperative death* | **2** | 0.08% |  |
|  |  | *Postoperative early mortality* | **1** | 0.04% |  |
|  |  | *Postoperative mortality* | **3** | 0.12% |  |
|  |  | *Total mortality* | **2** | 0.08% |  |
| **Life impact** \| \| \| \| \|; Physical functioning \| Gait: Spatiotemporal \| 10-meter walk test \| 4+**1** \| 0.24% \| 24; \| \| 2MWT \| 11+**3** \| 0.57% \|; \| \| 5-meter walk test \| 1+**1** \| 0.08% \|; \| \| 6MWT \| 6 \| 0.24% \|; \| \| Gait speed \| 4+**2** \| 0.24% \|; \| \| Gait speed (self-selected) \| 2 \| 0.08% \|; \| \| Gait speed (fast gait) \| 1 \| 0.04% \|; \| \| Stance time asymmetry index (self-selected) \| 1 \| 0.04% \|; \| \| Stance time asymmetry index (fast gait) \| 1 \| 0.04% \|; \| \| Static weight bearing and ability to walk (without prosthesis/ with light prosthesis/ with Osseo integrated implant) \| 1 \| 0.04% \|; \| \| Step count \| 6 \| 0.24% \|; \| \| Step length \| 1+**1** \| 0.08% \|; \| \| Step length asymmetry index (self-selected) \| 1 \| 0.04% \|; \| \| Step length asymmetry index (fast gait) \| 1 \| 0.04% \|; \| \| Limp index \| 1 \| 0.04% \|; \| \| Time spent walking \| 1 \| 0.04% \|; \| \| Walking time prior to lower limb amputation (minutes) \| 1 \| 0.04% \|; \| \| Stance characteristics \| 1 \| 0.04% \|; \| \| Step ascent task completion time \| 1 \| 0.04% \|; \| \| Step descent task completion time \| 1 \| 0.04% \|; \| \| *Cadence* \| **1** \| 0.04% \|; \| \| *Step time* \| **1** \| 0.04% \|; \| \| *Velocity* \| **1** \| 0.04% \|; \| \| *Distance walked* \| **2** \| 0.08% \|; \| Gait: Kinetics \| Index scores of peak sagittal plane moment \| 1 \| 0.04% \| 4; \| \| Peak vertical ground reaction force \| 1 \| 0.04% \|; \| \| Vertical impulse \| 1 \| 0.04% \|; \| \| Peak internal moments with step ascent and descent during single limb support \| 1 \| 0.04% \|; \| Gait: Kinematics \| Trunk rotational angular momentum during step ascent \| 1 \| 0.04% \| 2; \| \| Trunk rotational angular momentum during step descent \| 1 \| 0.04% \|; \| Balance \| Berg balance scale \| 4 \| 0.16% \| 7; \| \| Four square step test \| 1 \| 0.04% \|; \| \| One leg stand test \| 1 \| 0.04% \|; \| \| Centre of gravity excursions \| 1 \| 0.04% \|; \| \| *Inclination angles obtained through second order regression analysis using stabilogram data* \| **1** \| 0.04% \|; \| \| *Distribution of reaction forces between the two legs: balance* \| **1** \| 0.04% \|; \| \| *Centre of pressure trajectories and time function* \| **1** \| 0.04% \|; \| Balance confidence \| Activities and balance confidence scale \| 3 \| 0.12% \| 3; \| \| Falls efficacy scale-international \| 2 \| 0.08% \|; \| \| Modified falls efficacy scale \| 1 \| 0.04% \|; \| Endurance \| Standing tolerance (minutes) \| 1 \| 0.04% \| 1; \| Energy efficiency \| Physical activity scale for individuals with physical disabilities questionnaire \| 1 \| 0.04% \| 1; \| Functional independence \| Barthel index \| 4 \| 0.16% \| 18; \| \| Functional impact of symptoms \| 1 \| 0.04% \|; \| \| Functional independence \| 1+**4** \| 0.20% \|; \| \| Functional independence measure \| 3+**5** \| 0.32% \|; \| \| Functional status (bedridden) \| 1 \| 0.04% \|; \| \| Modified Barthel index \| 2 \| 0.08% \|; \| \| Physical function (independent, partially dependent, totally dependent) \| 1 \| 0.04% \|; \| \| Loss of independence \| 1 \| 0.04% \|; \| \| Pre-amputation functional status \| 1 \| 0.04% \|; \| \| *Barthel index effectiveness at discharge* \| **3** \| 0.12% \|; \| \| *Discharge functional status* \| **4** \| 0.16% \|; \| \| *Return to pre-morbid functional status* \| **1** \| 0.04% \|; \| \| *Functional activity level* \| **1** \| 0.04% \|; \| \| *Functional measure for amputees* \| **1** \| 0.04% \|; \| \| *Functional outcome* \| **2** \| 0.08% \|; \| \| *Independence of activities of daily living* \| **1** \| 0.04% \|; \| \| *Maintenance of independent living* \| **6** \| 0.24% \|; \| \| *Independence* \| **2** \| 0.08% \|; \| Impact of disease/conditional on activities of daily living \| World Health Organization disability assessment schedule \| 5 \| 0.20% \| 10; \| \| Activity limitations (bed mobility, sitting, standing balance transfer, standing tolerance, gait) \| 1 \| 0.04% \|; \| \| Effects of phantom phenomena on daily activities \| 1 \| 0.04% \|; \| \| Impact of pain and comorbid conditions (semi-structured interview) \| 1 \| 0.04% \|; \| \| Late life function and disability index-disability scale (frequency, limitation) \| 1 \| 0.04% \|; \| \| Negative changes in ambulatory status \| 1 \| 0.04% \|; \| \| Pain disability index \| 1 \| 0.04% \|; \| \| Pain with gait assessed by a numerical rating scale \| 1 \| 0.04% \|; \| \| *Patient specific functional scale* \| **1** \| 0.04% \|; \| \| *Groningen activity restriction scale (1-year)* \| **2** \| 0.08% \|; \| Locomotion ability \| Locomotor capability index-5 \| 7+**14** \| 0.85% \| 27; \| \| Modified Locomotor Capability Index \| 1 \| 0.04% \|; \| \| Post-operative ambulatory status (non-ambulatory, wheelchair, outdoor ambulation \| 1 \| 0.04% \|; \| \| Walking ability (no ability, at home/with barrier, no limitation) \| 1 \| 0.04% \|; \| \| Walking ability (with walker assistance) \| 1 \| 0.04% \|; \| \| Walking ability Index: Ability to walk 10 ft on a level (Gait, use of stick, crutch) \| 1 \| 0.04% \|; \| \| Walking ability with support \| 1 \| 0.04% \|; \| \| Walking category (independent community, limited community, limited household) \| 1 \| 0.04% \|; \| \| 100-meter walking ability outdoor and indoor (with/without cane) \| 1 \| 0.04% \|; \| \| Ambulatory status at discharge (ambulatory, ambulatory with assistance, wheelchair, bedridden) \| 1 \| 0.04% \|; \| \| Duration to ambulation (days) \| 1 \| 0.04% \|; \| \| *Functional ambulation level* \| **7** \| 0.28% \|; \| \| *Successful ambulation with prosthesis* \| **6** \| 0.24% \|; \| \| *Maintenance of ambulation* \| **4** \| 0.16% \|; \| \| *Independent Walking/ambulation* \| **3** \| 0.12% \|; \| \| *Walking ability at follow-up* \| **3** \| 0.12% \|; \| \| *Satisfactory ambulation by Walking ability index* \| **2** \| 0.08% \|; \| \| *Ambulation* \| **2** \| 0.08% \|; \| \| *Walking with pneumatic walking aid* \| **1** \| 0.04% \|; \| \| *Appearance and gait at 6 months* \| **1** \| 0.04% \|; \| \| *Locomotor performance after prosthesising* \| **1** \| 0.04% \|; \| \| *Means of ambulation* \| **1** \| 0.04% \|; \| \| *Method of ambulation at discharge* \| **1** \| 0.04% \|; \| \| *Mobilized without crutches* \| **1** \| 0.04% \|; \| \| *Prosthetic gait (mobile in or out of doors with prosthesis)* \| **1** \| 0.04% \|; \| \| *Walking in prosthesis (6-week, 4-month, 12-month)* \| **1** \| 0.04% \|; \| \| *Satisfactory walking results* \| **1** \| 0.04% \|; \| Mobility \| Timed up and go test \| 12+**5** \| 0.69% \| 30; \| \| Timed up and down stairs test \| 1 \| 0.04% \|; \| \| Basic amputee mobility score \| 1 \| 0.04% \|; \| \| Changes in functional mobility (wheelchair, walking ability) and activities of daily living (washing, dressing, toileting) \| 1 \| 0.04% \|; \| \| Interviews regarding mobility (walking with prosthesis, crutch, bedridden, wheelchair) \| 1 \| 0.04% \|; \| \| Life space assessment questionnaire \| 2 \| 0.08% \|; \| \| L-test of functional mobility \| 1 \| 0.04% \|; \| \| Mobility at 2-year (wheelchair, bedridden, ambulatory) \| 1 \| 0.04% \|; \| \| Mobility score \| 1+**1** \| 0.08% \|; \| \| Mobility status (ambulant/non-ambulant) \| 1 \| 0.04% \|; \| \| Pre-amputation mobility \| 1 \| 0.04% \|; \| \| Sitting, standing, and stepping behavior \| 1 \| 0.04% \|; \| \| Stanmore Harold Wood mobility grade \| 1+**1** \| 0.08% \|; \| \| Special Interest Group Amputation Medicine- Workgroup Amputation and Prosthetics mobility scale \| 1+**3** \| 0.16% \|; \| \| Special interest group in amputee medicine \| 3 \| 0.12% \|; \| \| Functional independence measure-mobility subscale \| 1+**2** \| 0.12% \|; \| \| Ambulation \| 1 \| 0.04% \|; \| \| Ambulatory rate (1-year) \| 1 \| 0.04% \|; \| \| Ambulatory status \| 1 \| 0.04% \|; \| \| Amputee mobility predictor \| 1 \| 0.04% \|; \| \| Amputee mobility predictor with/without prosthesis \| 1 \| 0.04% \|; \| \| Post-operative time to mobilization (days) \| 1 \| 0.04% \|; \| \| Amputee Single Item Mobility Measure \| 1 \| 0.04% \|; \| \| *Mobility level* \| **16** \| 0.65% \|; \| \| *Mobility at 6-month* \| **2** \| 0.08% \|; \| \| *Mobility at 1-year* \| **2** \| 0.08% \|; \| \| *Mobility in the home at discharge from rehabilitation* \| **2** \| 0.08% \|; \| \| *Minimum Data Set Activities of Daily Living Long Form Score* \| **1** \| 0.04% \|; \| \| *Mobility unaided without wheelchair* \| **1** \| 0.04% \|; \| \| *Rivermead mobility index* \| **1** \| 0.04% \|; \| Flexibility \| Modified Thomas test \| 1 \| 0.04% \| 1; \| Strength \| Strength (trunk) \| 1 \| 0.04% \| 1; \| Mobility potential \| K-level mobility \| 7+**1** \| 0.32% \| 1; \| Physical activity \| Percentage time in activity (sedentary, light, moderate/vigorous activity) \| 1 \| 0.04% \| 3; \| \| Accelerometer wear time for assessing physical activity \| 1 \| 0.04% \|; \| \| *Improvement of physical function at rehabilitation discharge* \| **1** \| 0.04% \|; \| Prosthesis fit \| Prosthesis fitting \| 8 \| 0.32% \| 7; \| \| Fit and function of the prosthesis (semi-structured interview) \| 1 \| 0.04% \|; \| \| *Successful fitting with prosthesis* \| **66** \| 2.68% \|; \| \| *Independent fitting of prosthesis* \| **1** \| 0.04% \|; \| \| *Rate of prosthetic fitting (1-year)* \| **1** \| 0.04% \|; \| \| *Initial prosthetic casting* \| **1** \| 0.04% \|; \| \| *Readiness for prosthetic fitting* \| **1** \| 0.04% \|; \| Prosthesis use \| Assessed suitability for prosthesis at discharge \| 1 \| 0.04% \| 39; \| \| Duration of prosthesis use (hours) \| 3+**1** \| 0.16% \|; \| \| Duration of prosthesis use (minutes) \| 1 \| 0.04% \|; \| \| Duration of prosthesis use (years) \| 1 \| 0.04% \|; \| \| Environmental factors item bank (assessed effects of environment on prosthetic functioning, accessibility) \| 1 \| 0.04% \|; \| \| Failure to long-term prosthetic use \| 1 \| 0.04% \|; \| \| Foot, socket type, and suspension method of prosthesis \| 1 \| 0.04% \|; \| \| Houghton scale \| 5+**1** \| 0.28% \|; \| \| Peak intra socket pressure analysis (7 to 28 psi using Fujifilm pre-scale film) \| 1 \| 0.04% \|; \| \| Pre-operative intention to use prosthesis \| 1 \| 0.04% \|; \| \| Prosthesis modification \| 1 \| 0.04% \|; \| \| Prosthesis use \| 5 \| 0.20% \|; \| \| Prosthesis use (rate and timing) \| 1 \| 0.04% \|; \| \| Prosthetic use score \| 1 \| 0.04% \|; \| \| Questionnaire for persons with a TFA modified for TTA for daily hours of prosthesis use \| 1 \| 0.04% \|; \| \| Received prosthesis (influence on mortality, major adverse cardiovascular events) \| 1 \| 0.04% \|; \| \| Re-initiation of prosthesis use \| 1 \| 0.04% \|; \| \| % received prosthesis \| 1 \| 0.04% \|; \| \| Type of prosthesis used \| 1 \| 0.04% \|; \| \| *Achievement of prosthesis use* \| **5** \| 0.20% \|; \| \| *Prosthetic prescription rate* \| **5** \| 0.20% \|; \| \| *Prosthetic use at follow-up* \| **5** \| 0.20% \|; \| \| *Prosthesis use (1-year)* \| **3** \| 0.12% \|; \| \| *Prosthesis use (6-month)* \| **3** \| 0.12% \|; \| \| *Rates of abandoning prosthesis use* \| **3** \| 0.12% \|; \| \| *Prosthetic prescription (1-year of amputation)* \| **2** \| 0.08% \|; \| \| *Daily hours of prosthetic wear (12-month, 18-month, 24-month)* \| **1** \| 0.04% \|; \| \| *Need for socket change* \| **1** \| 0.04% \|; \| \| *Prosthesis comfort (6-month)* \| **1** \| 0.04% \|; \| \| *Prosthetic device acquisition* \| **1** \| 0.04% \|; \| \| *Prosthetic restoration* \| **1** \| 0.04% \|; \| \| *Prosthetic use at 6 weeks and 4 months* \| **1** \| 0.04% \|; \| \| *Prosthetic wearing at follow-up* \| **1** \| 0.04% \|; \| \| *Referral for prosthetic rehabilitation* \| **1** \| 0.04% \|; \| \| *Self-reported hours of prosthetic use (6-month)* \| **1** \| 0.04% \|; \| \| *Unsuitable for prosthesis* \| **1** \| 0.04% \|; \| \| *Progress to definitive prosthesis* \| **1** \| 0.04% \|; \| \| *Use of prosthesis on discharge* \| **1** \| 0.04% \|; \| \| *Rate of progression through temporary prostheses* \| **1** \| 0.04% \|; \| Prosthesis-related mobility \| PEQ-mobility subscale \| 7+**1** \| 0.32% \| 8; \| \| Prosthetic limb user survey of mobility \| 5+**1** \| 0.24% \|; \| \| Satisfaction with mobility questionnaire \| 1 \| 0.04% \|; \| \| Walking ability with prosthesis and support \| 1 \| 0.04% \|; \| \| *Mobility with prosthesis* \| **11** \| 0.45% \|; \| \| *Function with a prosthesis (1-year)* \| **2** \| 0.08% \|; \| \| *Independent mobility with prosthesis* \| **1** \| 0.04% \|; \| \| *Mobility with temporary prosthesis* \| **1** \| 0.04% \|; \| Range of motion \| Knee joint range of motion \| 1 \| 0.04% \| 4; \| \| Peak hip extension range of motion \| 1 \| 0.04% \|; \| \| Active range of motion (shoulder, elbow, wrist) \| 1 \| 0.04% \|; \| \| Passive range of motion (shoulder, elbow, wrist) \| 1 \| 0.04% \|; \| Weight bearing \| Weight bearing on remaining leg \| 1 \| 0.04% \| 1; Social functioning \| Social participation \| Participation scale \| 1 \| 0.04% \| 8; \| \| Reintegration to normal living index \| 1+**1** \| 0.08% \|; \| \| PROMIS (participation in social roles and activities) \| 1 \| 0.04% \|; \| \| *Effect on home and work capacity* \| **1** \| 0.04% \|; \| \| *Return to work* \| **1** \| 0.04% \|; \| \| *Community participation (12-month)* \| **1** \| 0.04% \|; \| \| *Social integration (12-month)* \| **1** \| 0.04% \|; \| \| *Low social functioning (6-month)* \| **1** \| 0.04% \|; \| Social relationship \| Quality of relationship inventory \| 1 \| 0.04% \| 2; \| \| Social network index \| 1 \| 0.04% \|; \| Sexual activity \| *Self-reported frequency of sexual activity* \| **1** \| 0.04% \| 3; \| \| *Desire for sexual activity* \| **1** \| 0.04% \|; \| \| *Importance of sexual activity to satisfaction with life* \| **1** \| 0.04% \|; Cognitive functioning \| Cognitive function \| Montreal Cognitive assessment \| 1 \| 0.04% \| 9; \| \| Mini Mental State Examination \| 1 \| 0.04% \|; \| \| Folstein mental state examination (version 1) \| 1 \| 0.04% \|; \| \| *Digit span* \| **1** \| 0.04% \|; \| \| *List learning* \| **1** \| 0.04% \|; \| \| *List recall* \| **1** \| 0.04% \|; \| \| *Semantic fluency* \| **1** \| 0.04% \|; \| \| *Short portable mental status questionnaire* \| **1** \| 0.04% \|; \| \| *Right-left foot recognition* \| **1** \| 0.04% \| | | | | | |
| Delivery of care | Time to receive services | Time interval to delivery of prosthetic and orthotic care services | 2 | 0.08% | 18 |
|  |  | Time to receive angiography | 1 | 0.04% |  |
|  |  | Time to start physiotherapy | 1 | 0.04% |  |
|  |  | Days with no physiotherapy in hospital | 1 | 0.04% |  |
|  |  | Duration of delayed amputation | 1 | 0.04% |  |
|  |  | Reason for delayed amputation | 1 | 0.04% |  |
|  |  | Duration from amputation to rehabilitation | 1 | 0.04% |  |
|  |  | Duration from amputation to prosthesis mounting | 1 | 0.04% |  |
|  |  | Waiting for prosthesis | 1 | 0.04% |  |
|  |  | Days to early walking aid | 1 | 0.04% |  |
|  |  | Duration to revision | 1 | 0.04% |  |
|  |  | Days to compression therapy | 1 | 0.04% |  |
|  |  | Days to casting | 1+**1** | 0.08% |  |
|  |  | *Time until definitive prosthesis* | **5** | 0.20% |  |
|  |  | *Time to prosthetic fitting* | **5** | 0.20% |  |
|  |  | *Time to walking with prosthesis* | **2** | 0.08% |  |
|  |  | *Time to mobilisation* | **2** | 0.08% |  |
|  |  | *Days until patella tendon bearing cast* | **1** | 0.04% |  |
|  | Feasibility | Participant recruitment rate | 3 | 0.12% | 8 |
|  |  | Participant consent rate | 2 | 0.08% |  |
|  |  | Participant response rate | 2 | 0.08% |  |
|  |  | Participant retention rate | 2 | 0.08% |  |
|  |  | Completion of questionnaire rate | 1 | 0.04% |  |
|  |  | Participants reaching follow-up assessment | 1 | 0.04% |  |
|  |  | Time to conduct questionnaires | 1 | 0.04% |  |
|  |  | Recruited participants who provided adequate putative primary effectiveness outcomes data | 1 | 0.04% |  |
|  | Patient satisfaction | Semi-structured interviews for patients (assessing course of disease and treatment, moment amputation discusses 1st time and impact on life, decision making process to amputate, what is important in life, limitations due to disease, doctor’s enquiry about goals, information about amputation, rehabilitation, quality of life after amputation and best moment to talk about amputation) | 1 | 0.04% | 3 |
|  |  | *Satisfaction with mobility (12-month)* | **1** | 0.04% |  |
|  |  | *Satisfaction with treatment of pain* | **1** | 0.04% |  |
|  | Patient experience | Subjective interview regarding experience with nerve block | 1 | 0.04% | 2 |
|  |  | *Experiences of living with an amputation* | **1** | 0.04% |  |
|  | Patient preference | Patient plan to have prosthesis at day 21 | 1 | 0.04% | 1 |
|  | Fidelity | Dose attainment goal | 1 | 0.04% | 1 |
|  | Reliability of scoring | Reliability of Basic amputee mobility score scoring between experienced and inexperienced rater | 1 | 0.04% | 1 |
|  | Global impression of change | Patient global impression of change | 1 | 0.04% | 1 |
|  | Accessibility, quality and adequacy of intervention | Availability of and access to community resources (semi-structured interview) | 1 | 0.04% | 1 |
|  | Caregiver satisfaction | Semi-structured interviews for caregivers (assessing course of disease and treatment, moment amputation discusses 1st time and impact on life, decision making process to amputate, what is important in life, limitations due to disease, doctor’s enquiry about goals, information about amputation, rehabilitation, quality of life after amputation and best moment to talk about amputation) | 1 | 0.04% | 1 |
|  | Clinician satisfaction | Semi-structured interviews for healthcare professional (assessing typical disease process and treatment, when amputation is offered as treatment, communication with patient, when do you see amputation as realistic option, advantages/disadvantages and most important reason for amputation, information about amputation and rehabilitation, important in life for these patients, inquiring after goals, values and preferences, suggestions for improving communication, quality of life after amputation | 1 | 0.04% | 1 |
|  | Barriers to care | Barrier to care | 1 | 0.04% | 1 |
| Emotional functioning/well being | Self-efficacy | Self-efficacy of managing chronic disease questionnaire | 2 | 0.08% | 5 |
|  |  | 6-item self-efficacy for managing chronic disease scale | 1 | 0.04% |  |
|  |  | General self-efficacy scale | 1 | 0.04% |  |
|  |  | Self-efficacy of exercise scale | 1 | 0.04% |  |
|  |  | Washington self-efficacy scale | 1 | 0.04% |  |
|  | Coping ability | Connor-Davidson resilience questionnaire | 2 | 0.08% | 5 |
|  |  | Brief COPE scale to assess dispositional coping strategies | 1 | 0.04% |  |
|  |  | Coping attitudes evaluation scale | 1 | 0.04% |  |
|  |  | *Coping Strategies Questionnaire (6-month)* | **1** | 0.04% |  |
|  |  | *Psychological adaptation to limb loss* | **2** | 0.08% |  |
|  | Motivation | Intrinsic motivation inventory (interest and enjoyment subscale) | 2 | 0.08% | 2 |
|  |  | Motivation and attitude (semi-structured interview) | 1 | 0.04% |  |
|  | Loneliness | University of California, Los Angeles loneliness scale | 1 | 0.04% | 2 |
|  |  | Living alone | 1 | 0.04% |  |
|  | Self-esteem | Rosenberg self-esteem scale | 1 | 0.04% | 1 |
|  | Emotional functioning | *Low emotional functioning (6-month)* | **1** | 0.04% | 1 |
|  | Body image | Amputee body image scale | 1 | 0.04% | 2 |
|  |  | *Body Image Questionnaire (inpatient, in-rehabilitation, after discharge from rehabilitation)* | **1** | 0.04% |  |
| Personal circumstances | Residential status | Living alone | 1 | 0.04% | 6 |
|  |  | Accommodation (independent, wheelchair accessibility, nursing home) | 1 | 0.04% |  |
|  |  | Residential status pre-injury, post-operative (own home, residential home, nursing home) | 1 | 0.04% |  |
|  |  | *Change of residence* | **1** | 0.04% |  |
|  |  | *Living environment* | **1** | 0.04% |  |
|  |  | *Independent living status* | **1** | 0.04% |  |
|  | Employment | Change in employment | 1 | 0.04% | 1 |
| Perceived health status | Health status | Supporting Active Ageing through Multimodal coaching questionnaire | 1 | 0.04% | 3 |
|  |  | EQ-5D utility index | 1 | 0.04% |  |
|  |  | Sickness impact profile-68 | **1** | 0.04% |  |
|  | Perceived exertion | Rate of perceived exertion | 1 | 0.04% | 1 |
| Global quality of life | Prosthesis-related quality of life | Prosthesis evaluation questionnaire | 6 | 0.24% | 4 |
|  |  | Trinity amputation and prosthesis experience scale | 2 | 0.08% |  |
|  |  | Prostheses-specific Trinity amputation and prosthesis experience scales-revised questionnaire | 1 | 0.04% |  |
|  |  | Questionnaire for persons with transfemoral amputation | 1 | 0.04% |  |
|  | Quality of life | Short form-36 | 8+**2** | 0.41% | 12 |
|  |  | World Health Organization Quality of life Bref score | 3 | 0.12% |  |
|  |  | Euroqual-5D | 3+**1** | 0.16% |  |
|  |  | Satisfaction with life scale | 2 | 0.08% |  |
|  |  | Life satisfaction-11 questionnaire | 1 | 0.04% |  |
|  |  | Vascular amputee adapted quality of life questionnaire (adapted from World Health Organization quality of life-BREF and SF-36 questionnaires) | 1 | 0.04% |  |
|  |  | Structured interviews on quality-of-life domains relevant to vascular amputees (mobility, pain, concern about their remaining limb, mental health, independence, social function, personal postoperative experience and feelings at time of interview), additional focus group interviews for further exploration of structured interviews) | 1 | 0.04% |  |
|  |  | 12-item short form health survey | 1 | 0.04% |  |
|  |  | Semi-structured interviews concerning perceptions and experiences of living with amputation on altered mobility, social disruption, psychological impact, socioeconomic factors, accessibility, social support | 1 | 0.04% |  |
|  |  | Semi-structured interview (general health, rehabilitation after amputation, physical exercise, prosthesis, post-amputation phase) | 1 | 0.04% |  |
|  |  | *Quality of life* | **3** | 0.12% |  |
|  |  | *Satisfaction with life (12-month)* | **1** | 0.04% |  |
| **Physiological/clinical** | | | | | |
| Blood and lymphatic system outcomes | Laboratory parameters | Hemoglobin | 7+**3** | 0.41% | 15 |
|  |  | Hematocrit% | 3 | 0.12% |  |
|  |  | Platelet count | 3 | 0.12% |  |
|  |  | Albumin level | 3 | 0.12% |  |
|  |  | Partial thromboplastin time | 2 | 0.08% |  |
|  |  | Potassium level | 2 | 0.08% |  |
|  |  | Fibrinogen level | 1 | 0.04% |  |
|  |  | Sodium level | 1 | 0.04% |  |
|  |  | Erythrocyte sedimentation rate | 2 | 0.08% |  |
|  |  | Total protein | 1 | 0.04% |  |
|  |  | Thrombocyte level | 1 | 0.04% |  |
|  |  | PaCO_2_ | 1 | 0.04% |  |
|  |  | PaO_2_ | 1 | 0.04% |  |
|  |  | Lactate dehydrogenase levels | 1 | 0.04% |  |
|  |  | *Resting Tcpo_2_ (post therapy)* | **1** | 0.04% |  |
|  | Blood transfusion | Blood transfusion | 5+**2** | 0.28% | 6 |
|  |  | Need for blood transfusion | 2 | 0.08% |  |
|  |  | Blood transfusion per kilogram of bodyweight | 1 | 0.04% |  |
|  |  | Mean perioperative blood transfusion (units) | 1 | 0.04% |  |
|  |  | Use of blood transfusion (intra- and post-operative) | 1 | 0.04% |  |
|  |  | Red blood cell transfusion | 1 | 0.04% |  |
|  | Blood loss | Estimated blood loss | 1 | 0.04% | 10 |
|  |  | Total blood loss | 2 | 0.08% |  |
|  |  | Estimated blood loss (<250 ml, >250 ml) | 1 | 0.04% |  |
|  |  | Mean blood loss | 1 | 0.04% |  |
|  |  | Delta total intraoperative blood loss | 1 | 0.04% |  |
|  |  | Bleeding (very good, good, medium, minimal) | 1 | 0.04% |  |
|  |  | *Blood loss* | **2** | 0.08% |  |
|  |  | *Haemorrhage* | **1** | 0.04% |  |
|  |  | *Bleeding requiring 4 units blood (within 72 hours of surgery)* | **1** | 0.04% |  |
|  |  | *Wound bleeding* | **1** | 0.04% |  |
|  | Biomarkers | D-dimer levels | 1 | 0.04% | 2 |
|  |  | *CK-B level* | **1** | 0.04% |  |
|  | Diagnostic test | Oxygen saturation | 1+**1** | 0.08% | 1 |
|  | Anemia | Anemia (pre- and post-operative) | 1 | 0.04% | 1 |
| Cardiovascular outcomes | Diagnostic test | Ankle brachial index | 4 | 0.16% | 11 |
|  |  | Heart rate | 2 | 0.08% |  |
|  |  | Doppler ultrasonography | 1 | 0.04% |  |
|  |  | Echocardiography (ejection fraction) | 1 | 0.04% |  |
|  |  | Electrocardiogram | 1+**1** | 0.08% |  |
|  |  | Cardiovascular assessment prior to amputation (7 to 90 days, >91 days) | 1 | 0.04% |  |
|  |  | Blood pressure | 1 | 0.04% |  |
|  |  | *Resting heart rate* | **1** | 0.04% |  |
|  |  | *Heart rate maximum* | **1** | 0.04% |  |
|  |  | *Blood pressure maximum* | **1** | 0.04% |  |
|  |  | *% predicted heart rate maximum* | **1** | 0.04% |  |
| Endocrine outcomes | Diagnostic test | HbA1c levels | 2 | 0.08% | 1 |
|  | Laboratory parameters | Fasting blood glucose level | 1 | 0.04% | 2 |
|  |  | Blood glucose level | 1 | 0.04% |  |
|  | Physical examination of diabetic neuropathy | Michigan neuropathy screen physical assessment | 1 | 0.04% | 1 |
| General outcomes | Comorbidities | Functional comorbidity index | 5 | 0.20% | 11 |
|  |  | Age-adjusted Charlson comorbidity index | 2 | 0.08% |  |
|  |  | Charlson comorbidity index | 2 | 0.08% |  |
|  |  | Comorbidities | 3 | 0.12% |  |
|  |  | Dysvascular condition scale | 2 | 0.08% |  |
|  |  | *Pulmonary embolism* | **8** | 0.32% |  |
|  |  | *Venous thrombosis (deep vein thrombosis/pulmonary embolism)* | **7** | 0.28% |  |
|  |  | *Deep vein thrombosis* | **8** | 0.32% |  |
|  |  | *Peripheral vascular disease* | **1** | 0.04% |  |
|  |  | *Morbidity* | **3** | 0.12% |  |
|  |  | *Major morbidity* | **1** | 0.04% |  |
|  | Frailty | Modified frailty index | 3 | 0.12% | 5 |
|  |  | Frailty status | 2 | 0.08% |  |
|  |  | Modified frailty index (5-item) | 1 | 0.04% |  |
|  |  | Modified frailty index (11-item) | 1 | 0.04% |  |
|  |  | Risk analysis to classify frailty | 1 | 0.04% |  |
|  | Diagnostic test | Ultrasound | 2 | 0.08% | 2 |
|  |  | Frequency of diagnosis of single/multiple risk factors | 1 | 0.04% |  |
|  | Amputation | Age-standardized rates of major lower limb amputation | 1 | 0.04% | 7 |
|  |  | Stages of amputation (single/double stage) | 1 | 0.04% |  |
|  |  | Incident dysvascular amputation level (according to geographical regions, race/ethnicity) | 1 | 0.04% |  |
|  |  | Amputation of dominant limb | 1 | 0.04% |  |
|  |  | Odds of TFA as compared to TTA or TMT | 1 | 0.04% |  |
|  |  | *Contralateral limb preservation* | **2** | 0.08% |  |
|  |  | *Contralateral limb survival* | **1** | 0.04% |  |
|  | Physical examination | Physical examination | 2 | 0.08% | 2 |
|  |  | *Fever* | **1** | 0.04% |  |
| Immune system outcomes | Laboratory parameters | White blood cell count | 6 | 0.24% | 9 |
|  |  | C-reactive protein level | 4 | 0.16% |  |
|  |  | Lymphocyte level | 1 | 0.04% |  |
|  |  | Neutrophil level | 1 | 0.04% |  |
|  |  | *Oxidative burst capacity* | **1** | 0.04% |  |
|  |  | *Expression of CD-3, 4, 8, 19, 25, 69* | **1** | 0.04% |  |
|  |  | *IFN-gamma* | **1** | 0.04% |  |
|  |  | *Interleukin-4* | **1** | 0.04% |  |
|  |  | *Phagocytosis burst* | **1** | 0.04% |  |
|  | Biomarkers | Neutrophil /Albumin ratio | 1 | 0.04% | 5 |
|  |  | CRP/Albumin ratio | 1 | 0.04% |  |
|  |  | Platelet/lymphocyte ratio | 1 | 0.04% |  |
|  |  | Neutrophil/lymphocyte ratio | 1 | 0.04% |  |
|  |  | Neutrophil/cytokine ratio | 1 | 0.04% |  |
| Infection and infestation outcomes | Infection | Surgical site infection | 6+**1** | 0.28% | 12 |
|  |  | COVID-19 infection | 1 | 0.04% |  |
|  |  | Presence of infection | 1 | 0.04% |  |
|  |  | Post-operative skin infection | 1 | 0.04% |  |
|  |  | *Wound infection* | **15** | 0.61% |  |
|  |  | *Superficial wound infection* | **1** | 0.04% |  |
|  |  | *Deep wound infection* | **1** | 0.04% |  |
|  |  | *Stump infection* | **3** | 0.12% |  |
|  |  | *Wound infection (requiring re-operation)* | **1** | 0.04% |  |
|  |  | *Urinary tract infection* | **4** | 0.16% |  |
|  |  | *Sepsis* | **5** | 0.20% |  |
|  |  | *Lipid peroxidation processes* | 1 | 0.04% |  |
|  | Microbial culture | Microbial culture | 1 | 0.04% | 2 |
|  |  | Wound culture | 1 | 0.04% |  |
| Injury and poisoning outcomes | Falls | Fall incidence | 3+**1** | 0.16% | 3 |
|  |  | Falls per 1000 person years (fatal, non-fatal) | 1 | 0.04% |  |
|  |  | Fall description (activity at time of fall transfer, sit/stand transition, reaching/stationary activity, walking on level/uneven surface, other, curbs/steps, stair ascent/descent; prosthesis status: on, off, fell off, unknown; fall location: community, home) | 1 | 0.04% |  |
|  | Pressure ulcers | Braden scale | 1 | 0.04% | 3 |
|  |  | Pressure injuries present on admission and acquired at hospital (stage 1 to 4) | 1 | 0.04% |  |
|  |  | *Pressure sore incidence* | **2** | 0.08% |  |
|  | Fracture | Frequency of fractures (intracapsular, extracapsular) | 1 | 0.04% | 1 |
| Metabolic and nutritional outcomes | Nutritional control | Dieting i.e., nutritional control (occasional, daily) | 1 | 0.04% | 2 |
|  |  | Nutritional status (>10% weight loss in 6 months before operation) | 1 | 0.04% |  |
|  | Body mass | Body mass index | 2+**1** | 0.12% | 1 |
| Musculoskeletal and connective tissue outcomes | Radiology | X-ray | 3 | 0.12% | 1 |
|  | Diagnostic test | Integrated-EMG during step ascent and descent | 1 | 0.04% | 2 |
|  |  | 3-phase bone scintigraphy | 1 | 0.04% |  |
|  | Contractures | *Contracture of knee joint* | **1** | 0.04% | 4 |
|  |  | *flexion contractures* | **1** | 0.04% |  |
|  |  | *Frequency of contractures* | **1** | 0.04% |  |
|  |  | *Degree of contractures* | **1** | 0.04% |  |
| Nervous system outcomes | General pain | Numerical rating scale | 4 | 0.16% | 12 |
|  |  | Visual analogue scale | 2 | 0.08% |  |
|  |  | Changes/relapse in painful involuntary contractions | 1 | 0.04% |  |
|  |  | Pain | 1 | 0.04% |  |
|  |  | Pain intensity (0 to 10 scale) | 1 | 0.04% |  |
|  |  | Pain score | 1 | 0.04% |  |
|  |  | PROMIS (3a, 8a) short form for pain | 1 | 0.04% |  |
|  |  | Rest pain within 30 days before operation | 1 | 0.04% |  |
|  |  | Verbal rating scale for pain (0 to 10) | 1 | 0.04% |  |
|  |  | Wong-Baker FACES score | 1 | 0.04% |  |
|  |  | *Intraoperative pain in the sciatic nerve distribution* | **1** | 0.04% |  |
|  |  | *Pain during injection* | **1** | 0.04% |  |
|  | Low back pain | Number of low back pain events (prior and after the index amputation) | 1 | 0.04% | 3 |
|  |  | Age at 1st low back pain event | 1 | 0.04% |  |
|  |  | Low back pain (subjective) | 1 | 0.04% |  |
|  | Neuropathic pain | Leeds assessment of neuropathic symptoms and signs scores | 1 | 0.04% | 3 |
|  |  | Modified DN4 questionnaire | 1 | 0.04% |  |
|  |  | *Neuropathic pain scale* | **1** | 0.04% |  |
|  | Pain catastrophizing | Pain catastrophizing scale | 1 | 0.04% | 1 |
|  | Pain relief | Overall benefit of analgesia score | 1 | 0.04% | 4 |
|  |  | *Effective pain relief* | **1** | 0.04% |  |
|  |  | *Pain relief with peripheral block* | **1** | 0.04% |  |
|  |  | *Successful pain control* | **1** | 0.04% |  |
|  | Pain interference | Pain interference scale | 1 | 0.04% | 1 |
|  | Phantom limb pain | Phantom limb pain | 3+**15** | 0.73% | 11 |
|  |  | Phantom limb pain post-operative (subjective) | 1 | 0.04% |  |
|  |  | Phantom pain (no/little vs some/much) | 1 | 0.04% |  |
|  |  | % with phantom limb pain | 1 | 0.04% |  |
|  |  | Brief pain inventory short form (phantom limb pain) | 1 | 0.04% |  |
|  |  | Groningen questionnaire (phantom limb pain and chronic stump pain) | 1 | 0.04% |  |
|  |  | Phantom pain sensation amputee questionnaire | 1 | 0.04% |  |
|  |  | Modified phantom pain questionnaire modified | 1 | 0.04% |  |
|  |  | *Postoperative phantom pain intensity* | **1** | 0.04% |  |
|  |  | *Phantom pain (1-year)* | **1** | 0.04% |  |
|  |  | *Sherman's phantom limb questionnaire* | **1** | 0.04% |  |
|  | Phantom limb sensation | Phantom limb sensations | 1+**6** | 0.28% | 7 |
|  |  | Modified phantom phenomena questionnaire | 1 | 0.04% |  |
|  |  | *Quantitative sensory testing* | **1** | 0.04% |  |
|  |  | *Kinetic sensation* | **1** | 0.04% |  |
|  |  | *Kinesthetic sensation* | **1** | 0.04% |  |
|  |  | *Exteroceptive sensation* | **1** | 0.04% |  |
|  |  | *Super added sensation* | **1** | 0.04% |  |
|  | Radiology | Side of corticospinal tracts, lines, fractional anisotropy and apparent diffusion coefficient assessed by magnetic brain resonance imaging with diffusion tensor imaging | 1 | 0.04% | 1 |
|  | Sensation | Sensation in lower limbs | 1 | 0.04% | 2 |
|  |  | *Paresthesia during injection* | **1** | 0.04% |  |
|  | Residual limb pain | Residual limb pain | 2 | 0.08% | 30 |
|  |  | Stump limb pain | 1+**9** | 0.41% |  |
|  |  | Stump pain (no/little vs some/much) | 1 | 0.04% |  |
|  |  | Stump pain (subjective) | 1 | 0.04% |  |
|  |  | Brief pain inventory short form (residual limb pain) | 1 | 0.04% |  |
|  |  | Euroqual 5D-visual analogue scale | 1 | 0.04% |  |
|  |  | Groningen questionnaire (chronic stump pain) | 1 | 0.04% |  |
|  |  | *Pain (6-month)* | **2** | 0.08% |  |
|  |  | *Pain (3-month)* | **1** | 0.04% |  |
|  |  | *Stump pain (1-year)* | **2** | 0.08% |  |
|  |  | *Pain (3 to 6 months)* | **1** | 0.04% |  |
|  |  | *Stump pain in first 30 days, at 3 months, and at 6 months* | **1** | 0.04% |  |
|  |  | *Visual analogue scale* | **2** | 0.08% |  |
|  |  | *Numerical rating scale* | **2** | 0.08% |  |
|  |  | *McGill Pain Questionnaire* | **3** | 0.12% |  |
|  |  | *Pain characterization and intensity over time* | **1** | 0.04% |  |
|  |  | *Average pain scores per day* | **1** | 0.04% |  |
|  |  | *Chronic stump pain* | **2** | 0.08% |  |
|  |  | *Highest pain score* | **1** | 0.04% |  |
|  |  | *Pain intensity (first 24-hour)* | **1** | 0.04% |  |
|  |  | *Pain intensity/frequency* | **1** | 0.04% |  |
|  |  | *Pain threshold* | **2** | 0.08% |  |
|  |  | *Perioperative pain scores* | **1** | 0.04% |  |
|  |  | *Post-amputation chronic pain* | **2** | 0.08% |  |
|  |  | *Post-operative pain* | **3** | 0.12% |  |
|  |  | *Post-operative wound pain intensity* | **1** | 0.04% |  |
|  |  | *Prolonged limb pain* | **1** | 0.04% |  |
|  |  | *Reflex sympathetic dystrophy in stump* | **1** | 0.04% |  |
|  |  | *Stump allodynia* | **2** | 0.08% |  |
|  |  | *Temporal summation of pain* | **1** | 0.04% |  |
| Psychiatric outcomes | Addiction outcomes | Smoking history | 2 | 0.08% | 4 |
|  |  | Alcohol consumption | 2 | 0.08% |  |
|  |  | Illicit drug use | 2 | 0.08% |  |
|  |  | Tobacco use | 1 | 0.04% |  |
|  | Anxiety | General anxiety disorder-7 | 1 | 0.04% | 2 |
|  |  | Anxiety (Hospital Anxiety and Depression Score) | 2+**1** | 0.12% |  |
|  | Behavior change | Exercise stages of change scale | 1 | 0.04% | 1 |
|  | Delirium | Delirium | 1+**1** | 0.08% | 3 |
|  |  | Length of delirium | 1 | 0.04% |  |
|  |  | Post-operative confusion episodes | 1 | 0.04% |  |
|  | Mental health | Mental health diagnosis | 1 | 0.04% | 6 |
|  |  | Short portable mental status questionnaire | 1 | 0.04% |  |
|  |  | Change in mental status | 1 | 0.04% |  |
|  |  | Psychological impact of symptoms | 1 | 0.04% |  |
|  |  | *Low mental health (6-month)* | **1** | 0.04% |  |
|  |  | *Rate of suicidal ideation* | **1** | 0.04% |  |
|  | Fear of falling | Fear of falling (semi-structured interview) | 1 | 0.04% | 1 |
|  | Depression | Patient health questionnaire-9 | 4+**1** | 0.20% | 6 |
|  |  | Depression (Hospital anxiety and depression scale) | 2+**1** | 0.12% |  |
|  |  | Geriatric depression scale (15-item) | 1 | 0.04% |  |
|  |  | Center for epidemiological studies depression scale | 1 | 0.04% |  |
|  |  | *Depression (6-month)* | **1** | 0.04% |  |
|  |  | *Beck depression inventory (in-patient, in-rehabilitation, after discharge from rehabilitation)* | **1** | 0.04% |  |
| Respiratory, thoracic and mediastinal outcomes | Cardiopulmonary fitness | VO_2_ max | 1 | 0.04% | 1 |
| Renal and urinary outcomes | Laboratory parameters | Creatinine level | 5+**1** | 0.24% | 3 |
|  |  | Blood urea nitrogen | 4 | 0.16% |  |
|  |  | Estimated glomerular filtration rate | 1 | 0.04% |  |
| Skin and subcutaneous tissue outcomes | Stump wound healing | Wound healing | 2+**93** | 3.85% | 18 |
|  |  | Duration to healing (days) | 1+**6** | 0.28% |  |
|  |  | Postoperative wound healing (primary, delayed, no healing) | 1 | 0.04% |  |
|  |  | Residual limb wound healing | 1 | 0.04% |  |
|  |  | Time from amputation to healing (days) | 1 | 0.04% |  |
|  |  | Visual analogue scale (wound healing) | 1 | 0.04% |  |
|  |  | *Primary healing rate* | **6** | 0.24% |  |
|  |  | Healing rate | 1 | 0.04% |  |
|  |  | *Mode of wound healing* | **1** | 0.04% |  |
|  |  | *Stump healing* | **8** | 0.32% |  |
|  |  | *Delayed wound healing* | **4** | 0.16% |  |
|  |  | *Failed primary healing* | **2** | 0.08% |  |
|  |  | *Failure to heal* | **1** | 0.04% |  |
|  |  | *Secondary healing* | **1** | 0.04% |  |
|  |  | *Healing after revision* | **1** | 0.04% |  |
|  |  | *Healing after re-amputation to AKA* | **1** | 0.04% |  |
|  |  | *Not healed at follow-up* | **1** | 0.04% |  |
|  |  | *Stump volume reduction* | **1** | 0.04% |  |
|  | Wound | Chakrabarty scale determined residual limb quality and presence of wound | 1 | 0.04% | 10 |
|  |  | Physical examination of stump wound (presence of edema, discharge, infection) | 1 | 0.04% |  |
|  |  | Presence of residual limb wound | 1 | 0.04% |  |
|  |  | Wound classification (clean, clean-contaminated, contaminated, dirty) | 1 | 0.04% |  |
|  |  | Wound ischemia and foot infections core | 1 | 0.04% |  |
|  |  | *Wounds from prosthesis use (6-month)* | **1** | 0.04% |  |
|  |  | *Skin maceration* | **1** | 0.04% |  |
|  |  | *Decubitus ulcer* | **1** | 0.04% |  |
|  |  | *Wound occurrence* | **2** | 0.08% |  |
|  |  | *Stump defects and type* | **1** | 0.04% |  |
| Vascular outcomes | Chronic limb threatning ischemia staging | Infrainguinal Global Limb Anatomic Staging System  stage (I to III) | 1 | 0.04% | 1 |
|  | Diagnostic test | Doppler ultrasound | 2 | 0.08% | 13 |
|  |  | Duplex ultrasound | 1 | 0.04% |  |
|  |  | Arterial flow: Biphasic, monophasic, no flow (external iliac, femoral, deep artery of thigh, superficial femoral artery) | 1 | 0.04% |  |
|  |  | Intravascular ultrasound | 1 | 0.04% |  |
|  |  | Post-occlusive reactive hyperemia response following applied physical stress | 1 | 0.04% |  |
|  |  | Physical examination of popliteal and femoral pulse (palpable, not palpable, not described) | 1 | 0.04% |  |
|  |  | Transcutaneous oxygen tension by near infrared spectroscopy | 1 | 0.04% |  |
|  |  | Tissue saturation index by near infrared spectroscopy | 1 | 0.04% |  |
|  |  | Distal artery spectral waveform (triphasic, biphasic, monophasic) with Doppler ultrasonography on popliteal artery, anterior tibial artery, and posterior tibial artery | 1 | 0.04% |  |
|  |  | Peak systolic velocity (cm/sec) with Doppler ultrasonography on popliteal artery, anterior tibial artery, and posterior tibial artery | 1 | 0.04% |  |
|  |  | Volume flow (ml/min) with Doppler ultrasonography on popliteal artery, anterior tibial artery, and posterior tibial artery | 1 | 0.04% |  |
|  |  | Arterial diameter (cm) with Doppler ultrasonography on popliteal artery, anterior tibial artery, and posterior tibial artery | 1 | 0.04% |  |
|  |  | Deepest spectroscopy receiver (total hemoglobin, oxyhemoglobin, deoxyhemoglobin) | 1 | 0.04% |  |
|  | Radiology | Computed tomography angiogram | 6 | 0.24% | 4 |
|  |  | Angiogram scores (Grade 1 to 3) for femoropopliteal, infrapopliteal, and inframalleolar grades | 1 | 0.04% |  |
|  |  | Pre-operative angiography to demonstrate vessel run-off | 1 | 0.04% |  |
|  |  | Optical coherence tomography-based microangiography | 1 | 0.04% |  |
| **Resource use** | | | | | |
| Societal/carer burden | Carer burden | Zarit burden interview | 1 | 0.04% | 1 |
|  | Family support | Family support | 1 | 0.04% | 1 |
|  | Social support | Multidimensional scale of perceived social support | 3 | 0.12% | 7 |
|  |  | Modified social support survey | 3 | 0.12% |  |
|  |  | Daily assistance (homecare, relatives) | 1 | 0.04% |  |
|  |  | Informal and formal support (semi-structured interview) | 1 | 0.04% |  |
|  |  | Interpersonal support evaluation list–short-form scale | 1 | 0.04% |  |
|  |  | *Social service use* | **1** | 0.04% |  |
|  |  | *Home care arrangements* | **1** | 0.04% |  |
| Need for further intervention | Hemodialysis | Hemodialysis (admission) | 1 | 0.04% | 2 |
|  |  | *New onset dialysis* | **2** | 0.08% |  |
|  | Emergency procedure | Emergency procedures | 1 | 0.04% | 4 |
|  |  | Need for emergency surgery | 1 | 0.04% |  |
|  |  | *Deep vein thrombosis requiring therapy* | **1** | 0.04% |  |
|  |  | *Requirement for skin graft* | **1** | 0.04% |  |
|  | Respiratory support | Intubation | 1 | 0.04% | 8 |
|  |  | Use of respiratory support (intubation, continuous positive airway support) | 1 | 0.04% |  |
|  |  | *Re-intubation* | **1** | 0.04% |  |
|  |  | *Ventilator dependence* | **1** | 0.04% |  |
|  |  | *Prolonged mechanical ventilation requiring tracheostomy* | **1** | 0.04% |  |
|  |  | *Unplanned intubation for respiratory/cardiac failure* | **1** | 0.04% |  |
|  |  | *Prolonged intubation (48 hours)* | **1** | 0.04% |  |
|  |  | *Ventilatory support (port-operative)* | **1** | 0.04% |  |
|  | Management of fractures | Management of fractures (multiple parallel screws, cemented hemiarthroplasty, uncemented hemiarthroplasty, sliding hip screw, intramedullary nail, cemented hemiarthroplasty, non-operative) | 1 | 0.04% | 1 |
|  | Mobility aid | Use of assistive device | 1 | 0.04% | 10 |
|  |  | Discharge mobility aid | 1+**5** | 0.24% |  |
|  |  | Discharged TKA patients provided/not provided with prosthesis | 1 | 0.04% |  |
|  |  | Lacking assistive devices | 1 | 0.04% |  |
|  |  | Use of aids (pre-injury, post-operative, i.e., none, one stick, two sticks, Zimmer, wheelchair) | 1 | 0.04% |  |
|  |  | *Mobility aids used at follow-up* | **3** | 0.12% |  |
|  |  | *Wheelchair use (dependence)* | **2** | 0.08% |  |
|  |  | *Need for mobility (6-month)* | **1** | 0.04% |  |
|  |  | *Use of early walking aids* | **1** | 0.04% |  |
|  |  | *Ability to use parallel bars/walkers/crutches on discharge* | **1** | 0.04% |  |
|  | Predicting outcome | Estimation of likely outcomes of surgery by the healthcare professionals (surgeons: consultants and registrar level trainees regarding % likelihood prediction of death, morbidity and revision within 30-day) (anesthetists: consultants and registrar level trainees regarding morbidity and mortality at 30-day using visual analogue scale (0 to 100%) | 1 | 0.04% | 2 |
|  |  | Healthcare professionals using existing risk prediction tools | 1 | 0.04% |  |
|  | Prescription of medication | Use of medications | 22+**17** | 1.58% | 5 |
|  |  | Duration of medication use | 2+**3** | 0.20% |  |
|  |  | Dosage of medications | 2+**5** | 0.28% |  |
|  |  | Time until medication (analgesia) | 1 | 0.04% |  |
|  |  | Indication of medications | 1 | 0.04% |  |
|  | Resuscitation | Need for resuscitation | 1 | 0.04% | 1 |
|  | Rehabilitation | Duration of rehabilitation | 3+**6** | 0.36% | 12 |
|  |  | Delivery of physiotherapy | 1 | 0.04% |  |
|  |  | Time of inpatient physical therapy consultation (≤1 day vs >1 day) post-operatively (days) | 1 | 0.04% |  |
|  |  | Frequency of physiotherapy | 1 | 0.04% |  |
|  |  | *Rehabilitation success* | **11** | 0.45% |  |
|  |  | *Degree of rehabilitation* | **6** | 0.24% |  |
|  |  | *Rehabilitation outcome* | **4** | 0.16% |  |
|  |  | *Successful maintained level of rehabilitation at follow-up* | **1** | 0.04% |  |
|  |  | *Rehabilitation accomplishments* | **1** | 0.04% |  |
|  |  | *Rehabilitation status* | **1** | 0.04% |  |
|  |  | *Number and duration of physiotherapy treatments during early walking aid/prosthesis use* | **1** | 0.04% |  |
|  |  | *Weeks to achieve maximum benefit* | **2** | 0.08% |  |
|  | Torniquet use | Duration of torniquet | 1 | 0.04% | 2 |
|  |  | Use of torniquet during operation | 1 | 0.04% |  |
|  | Wound management | Wound vac (admission) | 1 | 0.04% | 2 |
|  |  | Postoperative stump dressing (elastic bandage, Plaster of Paris) | 1 | 0.04% |  |
|  | Use of anesthesia | American Society of Anesthesiologists grade | 6 | 0.24% | 11 |
|  |  | Use of anesthesia | 3 | 0.12% |  |
|  |  | Duration of anesthesia | 2 | 0.08% |  |
|  |  | Use of nerve block | 3 | 0.12% |  |
|  |  | Type of anesthesia | 2 | 0.08% |  |
|  |  | Conversion to general anesthesia | 1 | 0.04% |  |
|  |  | Need for anesthesia | 1 | 0.04% |  |
|  |  | *Onset time of nerve block* | **1** | 0.04% |  |
|  |  | *Sedation* | **2** | 0.08% |  |
|  |  | *Mean sedation score over first 96 hours* | **1** | 0.04% |  |
|  |  | *Sedation on a 1-5 numerical rating scale (first 3 days)* | **1** | 0.04% |  |
|  | Special provisions | Service provision by vascular center (immediate postoperative rigid dressing, specialist physiotherapist in first 14 days, daily inpatient gym, inpatient gym session ≥1-hour, prosthetic center on site when inpatient, prosthetic provision as inpatient, specialist physiotherapy outpatient service) | **1** | 0.04% | 3 |
|  |  | *Use of compression therapy* | **1** | 0.04% |  |
|  |  | *Quality improvement framework compliance* | **1** | 0.04% |  |
|  | Specialist consultation | Experience of specialist (resident/consultant) | 3 | 0.12% | 7 |
|  |  | Specialty of doctor (vascular, non-vascular) | 2 | 0.08% |  |
|  |  | Mental health outpatient visits (≥1 past year) | 1 | 0.04% |  |
|  |  | Visit to outpatient clinics (2 and 6 weeks after discharge) | 1 | 0.04% |  |
|  |  | Outpatient treatment per 1000 person years | 1 | 0.04% |  |
|  |  | Primary care consultation prior to amputation (7 to 30, 31 to 90, 91 to 180, 181 to 365, >365 days) | 1 | 0.04% |  |
|  |  | Palliative care consultation (time of death, amputation) | 1 | 0.04% |  |
|  | Reoperation | Reoperation | 2 | 0.08% | 9 |
|  |  | Reoperation rate | 1 | 0.04% |  |
|  |  | Repeat debridement | 1 | 0.04% |  |
|  |  | Reintervention | 1 | 0.04% |  |
|  |  | Need for re-operation (60-day) | 1 | 0.04% |  |
|  |  | Number of operations required | 1 | 0.04% |  |
|  |  | Unplanned operation (30-day) | 1 | 0.04% |  |
|  |  | *Debridement of stump* | **1** | 0.04% |  |
|  |  | *Further procedures* | **1** | 0.04% |  |
| Economic | Cost | Cost-effectiveness | 1 | 0.04% | 16 |
|  |  | Budget-impact analysis of microprocessor vs. non-microprocessor controlled prosthetic knee | 1 | 0.04% |  |
|  |  | Costs in Euros (prosthesis costs, fall-related medical costs, total costs) | 1 | 0.04% |  |
|  |  | Total summative costs of managing patients in dollars (mean and standard deviation for compensation of employees, theatre costs, radiology costs, laboratory costs, costs of medicines, goods and services for intensive care unit and general wards) | 1 | 0.04% |  |
|  |  | Economic evaluation (key cost drivers, resource usage during first 6-month post-operation i.e., general practitioner consultation, practice nurse consultation, primary care contact, other hospital contact, National Health Service contact, ambulance/paramedic contact, attendance at accident and emergency care, nights spent at hospital, contact at surgical/rehabilitation department, visits to hospital, saw a specialist nurse, saw a physiotherapist/occupational therapist) | 1 | 0.04% |  |
|  |  | Bespoke health resource use questionnaire | 1 | 0.04% |  |
|  |  | Intervention costs with perineural catheter (equipment used: reservoir pump, epidural mini, catheter replacement, staff time, drug acquisition) | 1 | 0.04% |  |
|  |  | *Resource utilization* | **1** | 0.04% |  |
|  |  | *Acute medical costs* | **1** | 0.04% |  |
|  |  | *Post acute medical costs* | **1** | 0.04% |  |
|  |  | *Hospital charges* | **2** | 0.08% |  |
|  |  | *Expenses* | **1** | 0.04% |  |
|  |  | *Hospital costs* | **1** | 0.04% |  |
|  |  | *Cost analysis* | **1** | 0.04% |  |
|  |  | *Financial burden of prosthesis* | **1** | 0.04% |  |
|  |  | *Rehabilitation charges* | **1** | 0.04% |  |
|  | Socioeconomic disadvantage | Area deprivation index | 1 | 0.04% | 1 |
|  | Insurance coverage | National health insurance coverage of amputees | 1 | 0.04% | 1 |
| Hospital | Admission | Admitted to intensive care unit | 3 | 0.12% | 12 |
|  |  | Need for intensive care unit | 2 | 0.08% |  |
|  |  | Admitted to emergency care | 2 | 0.08% |  |
|  |  | Admission to high dependency area/intensive care unit | 1+**1** | 0.08% |  |
|  |  | Admission to operating room | 1 | 0.04% |  |
|  |  | Admission to rehabilitation unit | 1 | 0.04% |  |
|  |  | Admitted from (home, nursing home, inpatient rehabilitation, hospital) | 1 | 0.04% |  |
|  |  | Hospitalizations (per 1000 person years) | 1 | 0.04% |  |
|  |  | Number of patients admitted to nursing home, residential care | 1 | 0.04% |  |
|  |  | Living in a nursing home | 1 | 0.04% |  |
|  |  | Transfer to ward (general, orthopedic) | 1 | 0.04% |  |
|  |  | Transfer to intensive care unit | 1 | 0.04% |  |
|  | Discharge | Discharge destination | 11+**24** | 1.42% | 17 |
|  |  | Days to discharge | 3 | 0.12% |  |
|  |  | Number of patients discharged home | 2 | 0.08% |  |
|  |  | Discharge from clinic | 1 | 0.04% |  |
|  |  | Discharge from hospital | 1 | 0.04% |  |
|  |  | Discharge to independent living | 1 | 0.04% |  |
|  |  | Discharged/not discharged to previous residence | 1 | 0.04% |  |
|  |  | Unplanned discharge from rehabilitation unit | 1 | 0.04% |  |
|  |  | *Discharge to independent living* | **2** | 0.08% |  |
|  |  | *Ultimately returned home* | **1** | 0.04% |  |
|  |  | *Proportion returned to previous living conditions (1-year)* | **1** | 0.04% |  |
|  |  | *Discharge setting* | **1** | 0.04% |  |
|  |  | *Disposition of patients* | **1** | 0.04% |  |
|  |  | *Residential status at discharge* | **1** | 0.04% |  |
|  |  | *Institutionalization* | **1** | 0.04% |  |
|  |  | *Discharge supports* | **2** | 0.08% |  |
|  |  | *Discharge with assistance* | **1** | 0.04% |  |
|  | Duration of operation | Duration of operation | 9+**2** | 0.81% | 1 |
|  | Operative outcome | Return to operation room | 4 | 0.16% | 4 |
|  |  | Intra-operative events (transfusion, hypertension, vasopressor use, hypoxia, tachycardia) | 1 | 0.04% |  |
|  |  | Failure to rescue score with active care curtailed (yes/no) | 1 | 0.04% |  |
|  |  | *Medical stability* | **1** | 0.04% |  |
|  | Operative success | Successful preservation of collateral circulation | 1 | 0.04% | 6 |
|  |  | Success of endovascular aneurysm repair procedure | 1 | 0.04% |  |
|  |  | Rate of successful identification of nerve and perineural catheter placement | 1 | 0.04% |  |
|  |  | Operative success | 1 | 0.04% |  |
|  |  | *Successful surgery after peripheral nerve blockade* | **9** | 0.36% |  |
|  |  | *Long term graft survival* | **1** | 0.04% |  |
|  | Duration of stay | Length of hospital stay | 20+**1** | 0.85% | 13 |
|  |  | Length of intensive care unit stay | 3+**1** | 0.16% |  |
|  |  | Postoperative length of hospital stay | 3 | 0.12% |  |
|  |  | Length of inpatient rehabilitation stay | 1 | 0.04% |  |
|  |  | Length of stay | 1+**51** | 2.11% |  |
|  |  | Length of stay at orthopedic department | 1 | 0.04% |  |
|  |  | Hospital or rehabilitation days | 1 | 0.04% |  |
|  |  | Length of admission | 1 | 0.04% |  |
|  |  | Length of acute rehabilitation unit stay | 1 | 0.04% |  |
|  |  | *Rehabilitation length of stay* | **11** | 0.45% |  |
|  |  | *Time in recovery room* | **1** | 0.04% |  |
|  |  | *Time to discharge* | **1** | 0.04% |  |
|  |  | *Prolonged hospital stay* | **1** | 0.04% |  |
|  | Readmission | Readmission (30-day) | 8 | 0.32% | 12 |
|  |  | Readmission rate (30-day) | 2 | 0.08% |  |
|  |  | Readmission (within 30 days of index discharge) | 1 | 0.04% |  |
|  |  | Readmission (within 90 days of index discharge) | 1 | 0.04% |  |
|  |  | Readmission (within 180 days of index discharge) | 1 | 0.04% |  |
|  |  | Readmission rate | 1 | 0.04% |  |
|  |  | Cause specific re-admission | 1 | 0.04% |  |
|  |  | Re-admission | 1 | 0.04% |  |
|  |  | Unexpected re-admission (30-day) | 1 | 0.04% |  |
|  |  | *Proportion having readmission* | **4** | 0.16% |  |
|  |  | *Occurrence of transfer back to acute service* | **1** | 0.04% |  |
|  |  | *Time to readmission with acute coronary syndrome* | **1** | 0.04% |  |
| **Adverse events** | | | | | |
| Adverse events/effects | Adverse events | Adverse events | 5 | 0.20% | 36 |
|  |  | New York heart association classification | 1 | 0.04% |  |
|  |  | Major adverse cardiovascular events | 2 | 0.08% |  |
|  |  | Incidence of nausea and vomiting (during in-patient stay) | 1 | 0.04% |  |
|  |  | Adverse events within 30-day | 1 | 0.04% |  |
|  |  | Adverse effect/ reactions | 1 | 0.04% |  |
|  |  | Adverse and serious events | 1 | 0.04% |  |
|  |  | Nausea | **1** | 0.04% |  |
|  |  | *Side effect occurrences* | **1** | 0.04% |  |
|  |  | *Stroke* | **14** | 0.57% |  |
|  |  | *Haemorrhagic stroke rate* | **1** | 0.04% |  |
|  |  | *Stroke (1-year)* | **1** | 0.04% |  |
|  |  | *Late stroke* | **1** | 0.04% |  |
|  |  | *Coma (24-hour)* | **1** | 0.04% |  |
|  |  | *Cardiac event* | **1** | 0.04% |  |
|  |  | *Cardiac arrhythmias* | **3** | 0.12% |  |
|  |  | *Cardiac arrest* | **2** | 0.08% |  |
|  |  | *Angina* | **1** | 0.04% |  |
|  |  | *Myocardial infarction* | **15** | 0.61% |  |
|  |  | *Myocardial infarction (1-year)* | **1** | 0.04% |  |
|  |  | *Late Myocardial infarction* | **1** | 0.04% |  |
|  |  | *Congestive heart failure* | **3** | 0.12% |  |
|  |  | *Acute renal failure* | **6** | 0.24% |  |
|  |  | *Renal insufficiency* | **1** | 0.04% |  |
|  |  | *Urinary retention* | **1** | 0.04% |  |
|  |  | *Delayed renal graft function* | **1** | 0.04% |  |
|  |  | *Primary nonfunction* | **1** | 0.04% |  |
|  |  | *Graft loss* | **1** | 0.04% |  |
|  |  | *Pneumonia* | **11** | 0.45% |  |
|  |  | *Pneumothorax* | **1** | 0.04% |  |
|  |  | *Aspiration* | **1** | 0.04% |  |
|  |  | *Venous thromboembolism (at 14 days in first 8 weeks)* | **1** | 0.04% |  |
|  |  | *Skin irritation (6-month)* | **1** | 0.04% |  |
|  |  | *Stump irritation* | **1** | 0.04% |  |
|  |  | *Synovial effusion* | **2** | 0.08% |  |
|  |  | *Leg oedema* | **1** | 0.04% |  |
|  | Complications | Postoperative complications | 10+**4** | 0.57% | 39 |
|  |  | Complications | 4 | 0.16% |  |
|  |  | Complications (30-day) | 2 | 0.08% |  |
|  |  | Gangrene (30-day before operation) | 1 | 0.04% |  |
|  |  | Postoperative myocardial infarction | 1 | 0.04% |  |
|  |  | Amputation complications | 1 | 0.04% |  |
|  |  | Clavien-Dindo severity grading system (post-operative complications) | 1 | 0.04% |  |
|  |  | Clinical course including complications | 1 | 0.04% |  |
|  |  | Inpatient complications | 1 | 0.04% |  |
|  |  | Necrosis % | 1 | 0.04% |  |
|  |  | Overall complications | 1 | 0.04% |  |
|  |  | Postoperative complications (30-day) | 1 | 0.04% |  |
|  |  | Pre-operative complications | 1 | 0.04% |  |
|  |  | Risk factors for wound complications (surgical site infection, wound dehiscence) | 1 | 0.04% |  |
|  |  | Stump complications | 1 | 0.04% |  |
|  |  | Stump revisions/complications | 1 | 0.04% |  |
|  |  | Surgical complications | 1 | 0.04% |  |
|  |  | Wound complications (30-day) | 1 | 0.04% |  |
|  |  | Perioperative complications | 1 | 0.04% |  |
|  |  | *Perioperative systemic complications* | **4** | 0.16% |  |
|  |  | *Cardiorespiratory complications* | **1** | 0.04% |  |
|  |  | *Nerve complications* | **1** | 0.04% |  |
|  |  | *Seroma/hematoma* | **1** | 0.04% |  |
|  |  | *Hematoma* | **1** | 0.04% |  |
|  |  | *Diabetic complications* | **1** | 0.04% |  |
|  |  | *Perforated duodenal ulcer* | **1** | 0.04% |  |
|  |  | *Gastrointestinal tract complications (constipation)* | **1** | 0.04% |  |
|  |  | *Cardiac complications* | **3** | 0.12% |  |
|  |  | *Bleeding complications* | **2** | 0.08% |  |
|  |  | *Block-related complications* | **1** | 0.04% |  |
|  |  | *Renal complications* | **1** | 0.04% |  |
|  |  | *Renal/urological complications* | **1** | 0.04% |  |
|  |  | *Respiratory/pulmonary complications* | **2** | 0.08% |  |
|  |  | *Stump related complications* | **1** | 0.04% |  |
|  |  | *Wound complications* | **17** | 0.69% |  |
|  |  | *Soft tissue complications* | **1** | 0.04% |  |
|  |  | *Postoperative stump necrosis* | **4** | 0.16% |  |
|  |  | *Biopsy-proven acute rejection* | **1** | 0.04% |  |
|  |  | *Wound dehiscence* | **2** | 0.08% |  |
|  | Reamputation/revision | Reamputation | 6 | 0.24% | 42 |
|  |  | Ipsilateral reamputation (1-year) | 2 | 0.08% |  |
|  |  | Revision surgery | 4 | 0.16% |  |
|  |  | Revision to higher level amputation | 3+**15** | 0.73% |  |
|  |  | Revision of major lower limb amputation | 1 | 0.04% |  |
|  |  | Revision (30-day) | 1 | 0.04% |  |
|  |  | Freedom from amputation stump revision/failure | 1 | 0.04% |  |
|  |  | Need for stump revision | 1 | 0.04% |  |
|  |  | Need of revision surgery | 1+**1** | 0.08% |  |
|  |  | Number of revision procedures (1-year) | 1 | 0.04% |  |
|  |  | Re-amputation (1-year) | 2+**1** | 0.12% |  |
|  |  | Re-amputation (30-day) | 3 | 0.12% |  |
|  |  | Re-amputation rate during hospitalization | 1 | 0.04% |  |
|  |  | Reamputation (within 180-day of index discharge) | 1 | 0.04% |  |
|  |  | Reamputation (within 90-day of index discharge) | 1 | 0.04% |  |
|  |  | Risk of contralateral minor lower limb amputation | 1 | 0.04% |  |
|  |  | Additional (ipsilateral/ contralateral) amputation (2-year) | 1 | 0.04% |  |
|  |  | Additional (ipsilateral/ contralateral) amputation (30-day) | 1 | 0.04% |  |
|  |  | Conversion from TTA to TFA | 1 | 0.04% |  |
|  |  | Duration from first to second amputation | 1 | 0.04% |  |
|  |  | Incidence of contralateral major lower limb amputation (30-days after index procedure) | 1 | 0.04% |  |
|  |  | Incidence of ipsilateral reamputation | 1 | 0.04% |  |
|  |  | Ipsilateral reamputation (30-day) | 1 | 0.04% |  |
|  |  | Mean time to TFA progression | 1 | 0.04% |  |
|  |  | Need for reamputation | 1 | 0.04% |  |
|  |  | Proximal major ipsilateral re-amputation | 1 | 0.04% |  |
|  |  | Re-amputation (1-month) | 1 | 0.04% |  |
|  |  | *Stump revision rate* | **9** | 0.36% |  |
|  |  | *Revision to AKA* | **24** | 0.97% |  |
|  |  | *Revision at same level* | **1** | 0.04% |  |
|  |  | *Revision rate* | **26** | 1.05% |  |
|  |  | *Revision or reamputation (30-day)* | **2** | 0.08% |  |
|  |  | *Re-amputation rate* | **40** | 1.62% |  |
|  |  | *Major amputation of the contralateral lower limb* | 15 | 0.61% |  |
|  |  | *Final amputation level* | **3** | 0.12% |  |
|  |  | *Ipsilateral reamputation* | **1** | 0.04% |  |
|  |  | *Time to contralateral amputation* | **1** | 0.04% |  |
|  |  | *Additional amputation rate* | **3** | 0.12% |  |
|  |  | *Time from amputation to revision* | **1** | 0.04% |  |
|  |  | *Re-amputation level* | **1** | 0.04% |  |
|  |  | *Re-amputation timing* | **1** | 0.04% |  |
|  |  | *Late revision or re-amputation* | **1** | 0.04% |  |
| TFA: Transfemoral amputation, TTA: Transtibial amputation, TKA: Through knee amputation, TMT: Transmetatarsal amputation | | | | | |

**References**

1.	Godlwana L, Stewart A, Musenge E. The effect of a home exercise intervention on persons with lower limb amputations: A randomized controlled trial. Clinical Rehabilitation. 2020;34(1):99-110.

2.	Akhtar MA, Hoellwarth JS, Al-Jawazneh S, Lu W, Roberts C, Al Muderis M. Transtibial osseointegration for patients with peripheral vascular disease: A case series of 6 patients with minimum 3-year follow-up. JBJS Open Access. 2021;6(2).

3.	AlMehman DA, Faden AS, Aldahlawi BM, Bafail MS, Alkhatieb MT, Kaki AM. Post-amputation pain among lower limb amputees in a tertiary care hospital in Jeddah, Saudi Arabia: A retrospective study. Saudi Medical Journal. 2022;43(2):187.

4.	Brier MJ, Williams RM, Turner AP, Henderson AW, Roepke AM, Norvell DC, et al. Quality of relationships with caregivers, depression, and life satisfaction after dysvascular lower extremity amputation. Archives of physical medicine and rehabilitation. 2018;99(3):452-8.

5.	Hanafi M, Hafiz A, Kassim NK, Chung Ty. Rehabilitation Medicine Management of a Bilateral Lower Limbs Amputee with Hemiparesis. International Medical Journal (1994). 2019;26:533-4.

6.	Davie-Smith F, Paul L, Nicholls N, Stuart WP, Kennon B. The impact of gender, level of amputation and diabetes on prosthetic fit rates following major lower extremity amputation. Prosthetics and orthotics international. 2017;41(1):19-25.

7.	Kwong M, Curtis EE, Mell MW. Underutilization of palliative care for patients with advanced peripheral arterial disease. Annals of Vascular Surgery. 2021;76:211-7.

8.	Fard B, Geertzen JHB, Dijkstra PU. Return home after dysvascular major amputation of the lower limb: A multicentre observational study in the Netherlands. Journal of rehabilitation medicine. 2020;52(1):00008.

9.	Gaffney BMM, Christiansen CL, Murray AM, Davidson BS. Trunk movement compensations and corresponding core muscle demand during step ambulation in people with unilateral transtibial amputation. Journal of electromyography and kinesiology. 2018;39:16-25.

10.	Shiraev T, de Boer M, Qasabian R. Indications for and outcomes of major lower limb amputations at a tertiary-referral centre in Australia. Vascular. 2023;31(5):941-7.

11.	Pourghaderi P, Yuquimpo KM, Guetter CR, Mansfield L, Park HS-M. Outcomes following lower extremity amputation in patients with diabetes mellitus and peripheral arterial disease. Annals of Vascular Surgery. 2020;63:259-68.

12.	Miller MJ, Blankenship JM, Kline PW, Melanson EL, Christiansen CL. Patterns of sitting, standing, and stepping after lower limb amputation. Physical therapy. 2021;101(2):pzaa212.

13.	Batten HR, McPhail SM, Mandrusiak AM, Varghese PN, Kuys SS. Gait speed as an indicator of prosthetic walking potential following lower limb amputation. Prosthetics and orthotics international. 2019;43(2):196-203.

14.	Kline PW, Murray AM, Miller MJ, Fields T, Christiansen CL. Error-augmentation gait training to improve gait symmetry in patients with non-traumatic lower limb amputation: A proof-of-concept study. Prosthetics and orthotics international. 2019;43(4):426-33.

15.	Kwak DG, Hur J-Y, Moon JS, Chang MC. Short-Term Walking Outcomes in Diabetic and Non-Diabetic Unilateral Transtibial Amputees. Diabetes & Metabolism Journal. 2020;44(4):614.

16.	Jayaraman C, Mummidisetty CK, Albert MV, Lipschutz R, Hoppe-Ludwig S, Mathur G, et al. Using a microprocessor knee (C-Leg) with appropriate foot transitioned individuals with dysvascular transfemoral amputations to higher performance levels: a longitudinal randomized clinical trial. Journal of neuroengineering and rehabilitation. 2021;18(1):88.

17.	Kimble SL. Acute inpatient rehabilitation interventions and outcomes for a person with quadrilateral amputation. Physical Therapy. 2017;97(2):161-6.

18.	Siriwardena GJ, Bertrand PV. Factors influencing rehabilitation of arteriosclerotic lower limb amputees. Journal of rehabilitation research and development. 1991;28(3):35-44.

19.	Wong CK, Chihuri ST. Impact of Vascular Disease, Amputation Level, and the Mismatch Between Balance Ability and Balance Confidence in a Cross-Sectional Study of the Likelihood of Falls Among People With Limb Loss: Perception Versus Reality. American Journal of Physical Medicine & Rehabilitation. 2019;98(2):130-5.

20.	Frengopoulos C, Payne MW, Viana R, Hunter SW. MoCA Domain Score Analysis and Relation to Mobility Outcomes in Dysvascular Lower Extremity Amputees. Archives of Physical Medicine and Rehabilitation. 2018;99(2):314-20.

21.	Zorn D, Lombardo J, Poiset S, Gutman M, Cappelli L, Hurwitz M, et al. Single-Dose Radiation Therapy Without Additional Surgery as a Treatment for Heterotopic Ossification Developing After Transfemoral Amputation. American Journal of Physical Medicine & Rehabilitation. 2022;101(11):e158-e61.

22.	Norvell DC, Williams RM, Turner AP, Czerniecki JM. The development and validation of a novel outcome measure to quantify mobility in the dysvascular lower extremity amputee: the amputee single item mobility measure. Clinical Rehabilitation. 2016;30(9):878-89.

23.	Wong CK, Gibbs WB. Factors associated with committed participation in a wellness-walking program for people with lower limb loss: a prospective cohort study. Prosthetics and Orthotics International. 2019;43(2):180-7.

24.	Torbjörnsson E, Ottosson C, Boström L, Blomgren L, Malmstedt J, Fagerdahl A-M. Health-related quality of life and prosthesis use among patients amputated due to peripheral arterial disease–a one-year follow-up. Disability and Rehabilitation. 2022;44(10):2149-57.

25.	Laroche D, Barnay J-L, Tourlonias B, Orta C, Obert C, Casillas J-M. Microcirculatory assessment of arterial Below-Knee stumps: Near-infrared spectroscopy versus transcutaneous oxygen Tension—A preliminary study in prosthesis users. Archives of Physical Medicine and Rehabilitation. 2017;98(6):1187-94.

26.	Christensen JC, Kline PW, Murray AM, Christiansen CL. Movement asymmetry during low and high demand mobility tasks after dysvascular transtibial amputation. Clinical Biomechanics. 2020;80:105102.

27.	Christiansen CL, Miller MJ, Murray AM, Stephenson RO, Stevens-Lapsley JE, Hiatt WR, et al. Behavior-change intervention targeting physical function, walking, and disability after dysvascular amputation: a randomized controlled pilot trial. Archives of physical medicine and rehabilitation. 2018;99(11):2160-7.

28.	Atallah R, Li JJ, Lu W, Leijendekkers R, Frölke JP, Al Muderis M. Osseointegrated transtibial implants in patients with peripheral vascular disease: a multicenter case series of 5 patients with 1-year follow-up. JBJS. 2017;99(18):1516-23.

29.	Miller MJ, Magnusson DM, Lev G, Fields TT, Cook PF, Stevens-Lapsley JE, et al. Relationships among perceived functional capacity, self-efficacy, and disability after dysvascular amputation. PM&R. 2018;10(10):1056-61.

30.	Czerniecki JM, Thompson ML, Littman AJ, Boyko EJ, Landry GJ, Henderson WG, et al. Predicting reamputation risk in patients undergoing lower extremity amputation due to the complications of peripheral artery disease and/or diabetes. Journal of British Surgery. 2019;106(8):1026-34.

31.	Lee LS, Hitzig SL, Mayo A, Devlin M, Dilkas S, MacKay C. Factors influencing physical activity among individuals with lower limb amputations: a qualitative study. Disability and Rehabilitation. 2023;45(9):1461-70.

32.	García LM, Torres EM, Velescu A, Duran CD, Peñart ML, Velasco AC. Natural history after below-knee amputation: analysis of survival and prosthetic fitting over 2 decades. Cirugía Española (English Edition). 2020;98(7):403-8.

33.	Chopra A, Azarbal AF, Jung E, Abraham CZ, Liem TK, Landry GJ, et al. Ambulation and functional outcome after major lower extremity amputation. Journal of vascular surgery. 2018;67(5):1521-9.

34.	Cheng R, Smith SR, Kalpakjian CZ. Comorbidity has no impact on unplanned discharge or functional gains in persons with dysvascular amputation. Journal of Rehabilitation Medicine (Stiftelsen Rehabiliteringsinformation). 2019;51(5).

35.	Richardson C, Crawford K, Milnes K, Bouch E, Kulkarni J. A clinical evaluation of postamputation phenomena including phantom limb pain after lower limb amputation in dysvascular patients. Pain Management Nursing. 2015;16(4):561-9.

36.	Pandit V, Nelson P, Kempe K, Gage K, Zeeshan M, Kim H, et al. Racial and ethnic disparities in lower extremity amputation: Assessing the role of frailty in older adults. Surgery. 2020;168(6):1075-8.

37.	Pandit V, Tan T-W, Kempe K, Chitwood J, Kim H, Horst V, et al. Frailty syndrome in patients with lower extremity amputation: simplifying how we calculate frailty. Journal of Surgical Research. 2021;263:230-5.

38.	Priyadharshan KP, Kumar N, Shanmugam D, Kadambari D, Kar SS. Quality of life in lower limb amputees: a cross-sectional study from a tertiary care center of South India. Prosthetics and orthotics international. 2022;46(3):246-51.

39.	Torbjörnsson E, Fagerdahl A-M, Blomgren L, Boström L, Ottosson C, Malmstedt J. Risk factors for reamputations in patients amputated after revascularization for critical limb-threatening ischemia. Journal of vascular surgery. 2021;73(1):258-66.

40.	Hitzig SL, Dilkas S, Payne MW, MacKay C, Viana R, Devlin M, et al. Examination of social disconnectedness and perceived social isolation on health and life satisfaction in community-dwelling adults with dysvascular lower limb loss. Prosthetics and orthotics international. 2022;46(2):155-63.

41.	Vasilchenko EM. Current Issues of the Rehabilitation of Disabled Elderly and Senile Persons with Limb Loss due to Obliterative Arterial Disease. ADVANCES IN GERONTOLOGY. 2019;9(4):478-82.

42.	Ratliff HT, Shibuya N, Jupiter DC. Minor vs. major leg amputation in adults with diabetes: six-month readmissions, reamputations, and complications. Journal of Diabetes and its Complications. 2021;35(5):107886.

43.	Camazine MN, Rountree KM, Smith JB, Bath J, Vogel TR. Opioid utilization after lower extremity amputation for peripheral vascular disease and discharge prescribing recommendations. Vascular. 2023;31(5):954-60.

44.	Wurdeman SR, Miller TA, Stevens PM, Campbell JH. Stability and Falls Evaluations in AMPutees (SAFE-AMP 1): Microprocessor knee technology reduces odds of incurring an injurious fall for individuals with diabetic/dysvascular amputation. Assistive Technology. 2023;35(3):205-10.

45.	Çamur S, Batıbay SG, Bayram S. Effect of lower extremity amputation on caregiving burden in caregivers of patients with diabetic foot: Prospective cohort study. International Wound Journal. 2020;17(4):890-6.

46.	Park YH, Song JH, Choi GW, Kim HJ. Predictors of complication following lower extremity amputation in diabetic end‐stage renal disease. Nephrology. 2018;23(6):518-22.

47.	Franchin M, Palermo V, Iannuzzi C, Rivolta N, Mozzetta G, Tozzi M, et al. A predictive score for 30-day survival for patients undergoing major lower limb amputation for peripheral arterial obstructive disease. Updates in Surgery. 2021;73:1989-2000.

48.	Pereira MG, Ramos C, Lobarinhas A, Machado JC, Pedras S. Satisfaction with life in individuals with a lower limb amputation: The importance of active coping and acceptance. Scandinavian Journal of Psychology. 2018;59(4):414-21.

49.	Huseynova K, Sutradhar R, Booth GL, Huang A, Ray JG. Risk of contralateral lower limb amputation and death after initial lower limb amputation–a population-based study. Heliyon. 2018;4(10).

50.	Baran U, Swanson E, Sanders JE, Wang RK. OCT-based microangiography for reactive hyperemia assessment within residual limb skin of people with lower-limb loss. Skin research and technology: official journal of International Society for Bioengineering and the Skin (ISBS)[and] International Society for Digital Imaging of Skin (ISDIS)[and] International Society for Skin Imaging (ISSI). 2018;24(1):152.

51.	Peters CML, De Vries J, Veen EJ, De Groot HG, Ho GH, Lodder P, et al. Is amputation in the elderly patient with critical limb ischemia acceptable in the long term? Clinical interventions in aging. 2019:1177-85.

52.	Koivunen V, Dabravolskaite V, Nikulainen V, Juonala M, Helmiö P, Hakovirta H. Major lower limb amputations and amputees in an aging population in Southwest Finland 2007–2017. Clinical Interventions in Aging. 2022:925-36.

53.	Aziz F, Reichardt B, Sourij C, Dimai H-P, Reichart D, Köhler G, et al. Epidemiology of major lower extremity amputations in individuals with diabetes in Austria, 2014–2017: A retrospective analysis of health insurance database. Diabetes Research and Clinical Practice. 2020;170:108477.

54.	Imanawanto K, Utami DA. Case Report Dysvascular Transtibial Amputation Due to Artery Stenosis: A Case Report Study. Surabaya Physical Medicine and Rehabilitation Journal. 2021.

55.	Zhang GQ, Canner JK, Kayssi A, Abularrage CJ, Hicks CW. Geographical socioeconomic disadvantage is associated with adverse outcomes following major amputation in diabetic patients. Journal of vascular surgery. 2021;74(4):1317-26.

56.	Chahrour MA, Habib JR, El Moheb MN, Cherfan P, Mahmoud D, El Rahyel A, et al. Incidence and predictors of surgical site infection complications in diabetic patients undergoing lower limb amputation. Annals of Vascular Surgery. 2022;81:343-50.

57.	Pande SD, Kamal A, Zaw E, Tin AS. Patients following lower limb amputation: A retrospective cohort study showing how to improve survival and rehabilitation outcomes. International Journal of Surgery Open. 2019;20:24-8.

58.	Albright-Trainer B, Phan T, Trainer RJ, Crosby ND, Murphy DP, Disalvo P, et al. Peripheral nerve stimulation for the management of acute and subacute post-amputation pain: a randomized, controlled feasibility trial. Pain Management. 2022;12(3):357-69.

59.	MacKay C, Cimino SR, Guilcher SJT, Mayo AL, Devlin M, Dilkas S, et al. A qualitative study exploring individuals’ experiences living with dysvascular lower limb amputation. Disability and rehabilitation. 2022;44(10):1812-20.

60.	England DL, Miller TA, Stevens PM, Campbell JH, Wurdeman SR. Mobility Analysis of AmpuTees (MAAT 7): Normative Mobility Values for Lower Limb Prosthesis Users of Varying Age, Etiology, and Amputation Level. American Journal of Physical Medicine & Rehabilitation. 2022;101(9):850-8.

61.	Nickinson ATO, Coles B, Zaccardi F, Gray LJ, Payne T, Bown MJ, et al. Missed opportunities for timely recognition of chronic limb threatening ischaemia in patients undergoing a major amputation: a population based cohort study using the UK's clinical practice research Datalink. European Journal of Vascular and Endovascular Surgery. 2020;60(5):703-10.

62.	Kuhlmann A, Hagberg K, Kamrad I, Ramstrand N, Seidinger S, Berg H. The Kenevo microprocessor-controlled prosthetic knee compared with non–microprocessor-controlled knees in individuals older than 65 years in Sweden: A cost-effectiveness and budget-impact analysis. Prosthetics and Orthotics International. 2022;46(5):414-24.

63.	Wurdeman SR, Stevens PM, Campbell JH. Mobility analysis of AmpuTees (MAAT 5): Impact of five common prosthetic ankle-foot categories for individuals with diabetic/dysvascular amputation. Journal of Rehabilitation and Assistive Technologies Engineering. 2019;6:2055668318820784.

64.	Alothman S, Alenazi A, Waitman LR, LeMaster J, Kluding P. Neuropathy and Other Risk Factors for Lower Extremity Amputation in People with Diabetes Using a Clinical Data Repository System. Journal of Allied Health. 2018;47(3):217-21.

65.	Shaw JL, Hackney JM, Vandeloecht KS, Newton SC, Rainey MA, Reed JJ, et al. Is Maximum Available Hip Extension Range of Motion of the Residual Limb Reached During Passive Prone Lying in the Acute Stage After Dysvascular Transtibial Amputation? JPO: Journal of Prosthetics and Orthotics. 2022;34(3):165-73.

66.	Wurdeman SR, Stevens PM, Campbell JH. Mobility Analysis of AmpuTees (MAAT I): Quality of life and satisfaction are strongly related to mobility for patients with a lower limb prosthesis. Prosthetics and orthotics international. 2018;42(5):498-503.

67.	Morisaki K, Yamaoka T, Iwasa K. Risk factors for wound complications and 30-day mortality after major lower limb amputations in patients with peripheral arterial disease. Vascular. 2018;26(1):12-7.

68.	Wang H, Yan Z, Nie M, Fu J. Ultrasound-Guided peripheral nerve blocks for major amputation in critically ill patients with peripheral artery disease. Acta Chirurgica Belgica. 2023;123(6):625-31.

69.	Roepke AM, Williams RM, Turner AP, Henderson AW, Norvell DC, Henson H, et al. A Longitudinal Study of Social Participation After Dysvascular Lower Extremity Amputation. American Journal of Physical Medicine & Rehabilitation. 2017;96(10):741-7.

70.	D'Cruz RT, Chong TT, Tan TF, Ting ZYP, Lee QWS, Wong TH, et al. The modified frailty index does not predict mortality after major lower extremity amputation for peripheral arterial disease in an Asian population. Annals of vascular surgery. 2020;69:298-306.

71.	Kuy S, Romero RAL, Kuy S. Gas gangrene of the diabetic foot. The Journal of the Louisiana State Medical Society. 2015;167(5):213-5.

72.	Vanicek N, Coleman E, Watson J, Bell K, McDaid C, Barnett C, et al. STEPFORWARD study: a randomised controlled feasibility trial of a self-aligning prosthetic ankle-foot for older patients with vascular-related amputations. BMJ open. 2021;11(3):e045195.

73.	O’Banion LA, Dirks R, Farooqui E, Kaups K, Qumsiyeh Y, Rome C, et al. Outcomes of major lower extremity amputations n dysvascular patients: room for improvement. The American Journal of Surgery. 2020;220(6):1506-10.

74.	Niskakangas M, Dahlbacka S, Liisanantti J, Vakkala M, Kaakinen T. Spinal or general anaesthesia for lower‐limb amputation in peripheral artery disease–a retrospective cohort study. Acta Anaesthesiologica Scandinavica. 2018;62(2):226-33.

75.	Cheun TJ, Jayakumar L, Sideman MJ, Ferrer L, Mitromaras C, Miserlis D, et al. Short-term contemporary outcomes for staged versus primary lower limb amputation in diabetic foot disease. Journal of vascular surgery. 2020;72(2):658-66.

76.	Vascular Quality I, Andersen J, Gabel J, Mannoia K, Kiang S, Patel S, et al. Association between preoperative indications and outcomes after major lower extremity amputation. The American Surgeon. 2019;85(10):1083-8.

77.	Satam K, Aboian E, Huttler J, Zhuo H, Zhang Y, Tonnessen B, et al. Eligibility of Patients with Chronic Limb Threatening Ischemia for Deep Venous Arterialization. Annals of Vascular Surgery. 2022;86:260-7.

78.	Beyaz S, Güler ÜÖ, Bağır GŞ. Factors affecting lifespan following below-knee amputation in diabetic patients. Acta orthopaedica et traumatologica turcica. 2017;51(5):393-7.

79.	Debeljak M, Vidmar G, Matjačić Z, Burger H. Information and communication technology used by people with lower limb loss in Slovenia. International Journal of Rehabilitation Research. 2020;43(2):188-91.

80.	Nijmeijer R, Voesten HGJM, Geertzen JHB, Dijkstra PU. Disarticulation of the knee: Analysis of an extended database on survival, wound healing, and ambulation. Journal of vascular surgery. 2017;66(3):866-74.

81.	Madsen UR, Bååth C, Berthelsen CB, Hommel A. A prospective study of short-term functional outcome after dysvascular major lower limb amputation. International journal of orthopaedic and trauma nursing. 2018;28:22-9.

82.	Adiyeke L, Karagoz B. Analysis of Doppler ultrasonography and computer tomography angiography for predicting amputation level and re-amputation rate. Northern Clinics of Istanbul. 2022;9(4):401.

83.	Spiera Z, Ilonzo N, Kaplan H, Leitman IM. Loss of independence as a metric for racial disparities in lower extremity amputation for diabetes: A National Surgery Quality Improvement Program (NSQIP) analysis. Journal of Diabetes and its Complications. 2022;36(1):108105.

84.	Wurdeman SR, Stevens PM, Campbell JH. Mobility analysis of AmpuTees (MAAT 6): mobility, satisfaction, and quality of life among long-term dysvascular/diabetic prosthesis users—results of a cross-sectional analysis. JPO: Journal of Prosthetics and Orthotics. 2021;33(3):161-7.

85.	Roepke AM, Turner AP, Henderson AW, Goldberg SB, Norvell DC, Czerniecki JM, et al. A prospective longitudinal study of trajectories of depressive symptoms after dysvascular amputation. Archives of physical medicine and rehabilitation. 2019;100(8):1426-33.

86.	Rombauts M, Duinslaeger E, Peers K, Kiekens C. Jumping stump phenomenon: a case report. Prosthetics and Orthotics International. 2022;46(2):191-4.

87.	Aljarrah Q, Allouh MZ, Bakkar S, Aleshawi A, Obeidat H, Hijazi E, et al. Major lower extremity amputation: a contemporary analysis from an academic tertiary referral centre in a developing community. BMC surgery. 2019;19:1-10.

88.	Kelly DA, Pedersen S, Tosenovsky P, Sieunarine K. Major lower limb amputation: outcomes are improving. Annals of vascular surgery. 2017;45:29-34.

89.	Lee SY, Kang SH, Kim D-K, Seo KM, Ro HJ, Kim JK. Changes in the corticospinal tract after wearing prosthesis in bilateral transtibial amputation. Prosthetics and Orthotics International. 2017;41(5):507-11.

90.	Kristensen MT, Nielsen AØ, Topp UM, Holmehave‐Brandt J, Petterson CF, Gebuhr P. Development and psychometric properties of the Basic Amputee Mobility Score for use in patients with a major lower extremity amputation. Geriatrics & Gerontology International. 2018;18(1):138-45.

91.	Janssen ERI, van Silfhout L. Duplex ultrasound may predict the best level of lower limb amputation in patients with chronic limb-threatening ischemia: a retrospective observational cohort study. Annals of Vascular Surgery. 2020;67:403-10.

92.	Gailey R, Gaunaurd I, Raya M, Kirk-Sanchez N, Prieto-Sanchez LM, Roach K. Effectiveness of an evidence-based amputee rehabilitation program: a pilot randomized controlled trial. Physical therapy. 2020;100(5):773-87.

93.	Boussi L, Safaya A, Goyal A, Mateo R, Laskowski I, Babu S. “Inflammatory Bowel Disease—Not Just the Bowel’s Bane”: Peripheral Arterial and Venous Thrombosis in a Patient With Crohn Disease. Vascular and Endovascular Surgery. 2020;54(7):646-9.

94.	Columbo JA, Davies L, Kang R, Barnes JA, Leinweber KA, Suckow BD, et al. Patient experience of recovery after major leg amputation for arterial disease. Vascular and endovascular surgery. 2018;52(4):262-8.

95.	Norvell DC, Czerniecki JM. Risks and risk factors for ipsilateral re-amputation in the first year following first major unilateral dysvascular amputation. European Journal of Vascular and Endovascular Surgery. 2020;60(4):614-21.

96.	Molina-Rueda F, Molero-Sánchez A, Carratalá-Tejada M, Cuesta-Gómez A, Miangolarra-Page JC, Alguacil-Diego IM. Limits of stability in patients with vascular (due to diabetes) and nonvascular unilateral transtibial amputation: a cross-sectional study. International Journal of Rehabilitation Research. 2017;40(3):227-31.

97.	Miller MJ, Morris MA, Magnusson DM, Putnam K, Cook PF, Schenkman ML, et al. Psychosocial factors influence physical activity after dysvascular amputation: a convergent mixed‐methods study. PM&R. 2021;13(7):737-45.

98.	Wied C, Tengberg PT, Holm G, Kallemose T, Foss NB, Troelsen A, et al. Tourniquets do not increase the total blood loss or re-amputation risk in transtibial amputations. World Journal of Orthopedics. 2017;8(1):62.

99.	Roijers JP, Hopmans CJ, Janssen TL, Mulder PGH, Buimer MG, Ho GH, et al. The role of delirium and other risk factors on mortality in elderly patients with critical limb ischemia undergoing major lower limb amputation. Annals of Vascular Surgery. 2019;60:270-8.

100.	Ichihashi S, Yamamoto T, Bolstad F, Koshi K. Direct puncture of an occluded common femoral artery as a new approach for endovascular aortic aneurysm repair. CVIR Endovascular. 2021;4(1):58.

101.	Mtshali S, Hongoro C, Mahomed O. Hospitalization Costs for Diabetes Related Lower Extremity Amputation at a Referral Hospital in KwaZulu Natal, South Africa. Current Diabetes Reviews. 2022;18(9):96-101.

102.	Gurel R, Elbaz E, Ashkenazi I, Rotman D, Factor S, Chechik O, et al. Trans tibial amputation with or without a tourniquet in patients with diabetic foot infection and peripheral vascular disease: Comparison of postoperative outcomes. Journal of Orthopaedic Surgery. 2022;30(2):10225536221102694.

103.	Brugliera L, Spina A, Castellazzi P, Cimino P, Arcuri P, Deriu MG, et al. REHABILITATION OF COVID-19 PATIENTS WITH ACUTE LOWER EXTREMITY ISCHAEMIA AND AMPUTATION. Journal of Rehabilitation Medicine (Stiftelsen Rehabiliteringsinformation). 2020;52(9).

104.	Dallas J, Block JJ, Bastas G. Femoral Artery Bypass Graft Pseudoaneurysm Rupture in a Transfemoral Amputee. American Journal of Physical Medicine & Rehabilitation. 2018;97(11):e104-e6.

105.	Ogaki T, Iida O, Hata Y, Yamauchi N, Yokoi C, Takahara M, et al. The perioperative and long‐term fates of patients with chronic limb‐threatening ischaemia who underwent secondary major amputations. International Wound Journal. 2022;19(1):36-43.

106.	Shamir S, Schwartz Y, Cohen D, Bdolah-Abram T, Yinnon AM, Wiener-Well Y. The timing of limb amputation in nontraumatic patients: impact on mortality and postoperative complication rates. The Journal of Foot and Ankle Surgery. 2022;61(2):293-7.

107.	Friedman A, Zilberman S, Genis A, Khutornyuk T, Lutsky L, Treger I. Leg dominance as a determinant in laterality of lower extremity amputation in diabetic patients: retrospective study and literature review. International Journal of Therapy and Rehabilitation. 2020;27(10):1-7.

108.	Günay AE, Ekici M. Relationship between neutrophil/albumin ratio and early mortality after major lower extremity amputation. Cureus. 2021;13(9).

109.	Junaidi F, Muradi A, Pratama D, Suhartono R, Kekalih A. Effectiveness of Doppler ultrasonography as a predictor of wound healing after below-knee amputation for peripheral arterial disease. Chirurgia (Bucur). 2020;115:618-25.

110.	Kishikawa H, Wajima Zi, Shitara T, Shimizu T, Adachi H, Sakamoto A. Subarachnoid block-induced deafferentation pain successfully treated with pentazocine. Journal of Nippon Medical School. 2017;84(4):183-5.

111.	Clemens SM, Kershaw KN, McDonald CL, Darter BJ, Bursac Z, Garcia SJ, et al. Disparities in functional recovery after dysvascular lower limb amputation are associated with employment status and self-efficacy. Disability and Rehabilitation. 2023;45(14):2280-7.

112.	Hagan R, Kadzi J, Rahman G, Morna M. PATTERNS AND INDICATIONS OF AMPUTATION IN CAPE COAST TEACHING HOSPITAL: A FOUR YEAR RETROSPECTIVE REVIEW. Journal of the West African College of Surgeons. 2018;8(3):45-58.

113.	Cotton J, Cabot J, Buckner J, Field A, Pounds L, Quint C. Increased frailty associated with higher long-term mortality after major lower extremity amputation. Annals of Vascular Surgery. 2022;86:295-304.

114.	Mayo AL, Viana R, Dilkas S, Payne M, Devlin M, MacKay C, et al. Self-reported health condition severity and ambulation status postmajor dysvascular limb loss. Prosthetics and Orthotics International. 2022;46(3):239-45.

115.	de Boer M, Shiraev T, Waller J, Aitken S, Qasabian R. Patient and geographical disparities in functional outcomes after major lower limb amputation in Australia. Annals of Vascular Surgery. 2022;85:125-32.

116.	Kalkan A, Bas B, Kocer T, Atis SE, Unver SS, Koca YS. Stent Graft Expulsion Outside the Skin: A Rare Case. Advances in Skin & Wound Care. 2020;33(5):1-3.

117.	Otsuka T, Arai M, Sugimura K, Sakai M, Nishizawa Y, Suzuki Y, et al. Preoperative sepsis is a predictive factor for 30-day mortality after major lower limb amputation among patients with arteriosclerosis obliterans and diabetes. Journal of Orthopaedic Science. 2020;25(3):441-5.

118.	Haleem S, Yousaf S, Hamid T, Nagappa S, Parker MJ. Characteristics and outcomes of hip fractures in lower limb amputees. Injury. 2021;52(4):914-7.

119.	Shin Y-H, Yoon JS, Jeon HJ, Kim YB, Kim YS, Park JY. Postoperative delirium in elderly patients with critical limb ischemia undergoing major leg amputation: a retrospective study. Korean journal of anesthesiology. 2018;71(4):311-6.

120.	Meyer A, Griesbach C, Maudanz N, Lang W, Almasi-Sperling V, Rother U. Influence of end-stage renal disease on long-term survival after major amputation. Vasa. 2020.

121.	Rammell J, Perre D, Boylan L, Prentis J, Nesbitt C, Elmallah A, et al. The adverse impact of pre-operative anaemia on survival following major lower limb amputation. Vascular. 2023;31(2):379-86.

122.	Bosanquet DC, Ambler GK, Waldron C-A, Thomas-Jones E, Brookes-Howell L, Kelson M, et al. Perineural local anaesthetic catheter after major lower limb amputation trial (PLACEMENT): results from a randomised controlled feasibility trial. BMJ Open. 2019;9(11):e029233.

123.	Hebenton J, Scott H, Seenan C, Davie-Smith F. Relationship between models of care and key rehabilitation milestones following unilateral transtibial amputation: a national cross-sectional study. Physiotherapy. 2019;105(4):476-82.

124.	Majstorović B, Pešta M. Factors predicting rehabilitation outcome in patients after unilateral transtibial amputation due to peripheral vascular disease. Vojnosanitetski pregled. 2020;77(4).

125.	Andersen JC, Gabel JA, Mannoia KA, Kiang SC, Patel ST, Teruya TH, et al. 5-Item modified frailty index predicts outcomes after below-knee amputation in the vascular quality initiative amputation registry. The American Surgeon. 2020;86(10):1225-9.

126.	Christiansen CL, Miller MJ, Kline PW, Fields TT, Sullivan WJ, Blatchford PJ, et al. Biobehavioral intervention targeting physical activity behavior change for older veterans after nontraumatic amputation: a randomized controlled trial. PM&R. 2020;12(10):957-66.

127.	Nieuwstraten JA, van Doorn LP, Gebhardt WA, Hamming JF. Stakeholder values and preferences in lower limb amputation for no-option chronic limb threatening ischemia. Patient preference and adherence. 2021:1051-9.

128.	Kizilkurt OK, Kizilkurt T, Gulec MY, Giynas FE, Polat G, Kilicoglu OI, et al. Quality of life after lower extremity amputation due to diabetic foot ulcer: the role of prosthesis-related factors, body image, self-esteem, and coping styles. Dusunen adam: Journal of psychiatry & neurological sciences. 2020;33(2).

129.	Hurwitz M, Norvell DC, Czerniecki JM. Racial and ethnic amputation level disparities in veterans undergoing incident dysvascular lower extremity amputation. PM&R. 2022;14(10):1198-206.

130.	Wied C, Tengberg PT, Kristensen MT, Holm G, Kallemose T, Troelsen A, et al. Total blood loss after transfemoral amputations is twice the intraoperative loss: An observational cohort study of 81 nontraumatic amputations. Geriatric Orthopaedic Surgery & Rehabilitation. 2017;8(3):123-7.

131.	Luetmer M, Mundell B, Kremers HM, Visscher S, Hoppe KM, Kaufman KR. Low Back Pain in Adults With Transfemoral Amputation: A Retrospective Population‐Based Study. PM&R. 2019;11(9):926-33.

132.	Fard B, Dijkstra PU, Voesten HGJM, Geertzen JHB, Group NS. Mortality, reamputation, and preoperative comorbidities in patients undergoing dysvascular lower limb amputation. Annals of vascular surgery. 2020;64:228-38.

133.	Mundell BF, Luetmer MT, Kremers HM, Visscher S, Hoppe KM, Kaufman KR. The risk of major cardiovascular events for adults with transfemoral amputation. Journal of neuroengineering and rehabilitation. 2018;15:1-7.

134.	Chang H, Maldonado TS, Rockman CB, Cayne NS, Berland TL, Barfield ME, et al. Closed incision negative pressure wound therapy may decrease wound complications in major lower extremity amputations. Journal of Vascular Surgery. 2021;73(3):1041-7.

135.	Gwilym BL, Pallmann P, Waldron C-A, Thomas-Jones E, Milosevic S, Brookes-Howell L, et al. Short-term risk prediction after major lower limb amputation: PERCEIVE study. British Journal of Surgery. 2022;109(12):1300-11.

136.	Silva LR, Fernandes GM, Morales NU, Sobreira ML, Moura R, Bertanha M, et al. Results of one-stage or staged amputations of lower limbs consequent to critical limb ischemia and infection. Annals of vascular surgery. 2018;46:218-25.

137.	Alfawaz A, Kotha VS, Nigam M, Bekeny JC, Black CK, Tefera E, et al. Popliteal artery patency is an indicator of ambulation and healing after below-knee amputation in vasculopaths. Vascular. 2022;30(4):708-14.

138.	Yaqoob I, Khalil K, Fayyaz R, Khan A. Functional ability and quality of life of below knee amputees with prosthesis. Rawal Medical Journal. 2018;43(4):708-.

139.	Ner EB, Ron G, Essa A, Levy A, Finestone AS, Tamir E. Lower extremity amputee outcomes with reference to co-morbidities. The Israel Medical Association journal: IMAJ. 2022;24(7):470-4.

140.	de Boer RG, Paping MA, Kap B, Geertzen JH. Residual limb claudication after vascular transfemoral amputation. Prosthetics and Orthotics International. 2017;41(6):601-4.

141.	Cruz Silva J, Constâncio Oliveira V, Lima P, Correia M, Moreira M, Anacleto G. Change in Domains that Influence Quality of Life after Major Lower Limb Amputation in Patients with Peripheral Arterial Disease. Annals of Vascular Surgery. 2021;75:179-88.

142.	Varino J, Mendes C, Marinho A, Rodrigues R, Pereira B, Moreira M, et al. Mortalidade a curto e longo prazo após amputação major do membro inferior numa população octagenária. Angiologia e Cirurgia Vascular. 2017;13(2):15-25.

143.	Baysal O, Msangi WJ, GÜNar B, Bulent E. Does re-amputation following lower extremity amputation in diabetic or dysvascular patients negatively affect survival? Marmara Medical Journal. 2022;35(1):73-8.

144.	Collins A, Timmons S. Mobility outcomes for those with primary lower limb amputation attending a regional outpatient prosthetic rehabilitation service. Physiotherapy Practice and Research. 2018;39(1):27-35.

145.	Taylor SM, Kalbaugh CA, Blackhurst DW, Hamontree SE, Cull DL, Messich HS, et al. Preoperative clinical factors predict postoperative functional outcomes after major lower limb amputation: an analysis of 553 consecutive patients. Journal of vascular surgery. 2005;42(2):227-34.

146.	Miller MJ, Mealer ML, Cook PF, Kittelson AJ, Christiansen CL. Psychometric Assessment of the Connor-Davidson Resilience Scale for People With Lower-Limb Amputation. Phys Ther. 2021;101(4).

147.	Ratliff CR. What is the frequency of pressure injury in vascular patients undergoing major amputations? J Vasc Nurs. 2020;38(2):72-5.

148.	Wied C, Foss NB, Tengberg PT, Holm G, Troelsen A, Kristensen MT. Avoidable 30-day mortality analysis and failure to rescue in dysvascular lower extremity amputees. Acta Orthop. 2018;89(2):246-50.

149.	Madsen UR, Baath C, Berthelsen CB, Hommel A. Age and health-related quality of life, general self-efficacy, and functional level 12 months following dysvascular major lower limb amputation: a prospective longitudinal study. Disabil Rehabil. 2019;41(24):2900-9.

150.	Endoh S, Yamana H, Nakahara Y, Matsui H, Fushimi K, Yasunaga H, et al. Risk Factors for In-hospital Mortality and Reamputation Following Lower Limb Amputation. Progress in Rehabilitation Medicine. 2017;2.

151.	Gabel J, Jabo B, Patel S, Kiang S, Bianchi C, Chiriano J, et al. Analysis of Patients Undergoing Major Lower Extremity Amputation in the Vascular Quality Initiative. Annals of Vascular Surgery. 2018;46:75-82.

152.	Langnas EM, Gray A, Braehler M. Erector spinae plane block for postoperative analgesia for above-the-knee amputation: a case report. Perioperative Medicine. 2022;11(1):40.

153.	De Klerk C, Du Plessis G, Fourie JJ, O’Neill A, Smit SJA, Joubert G. The eventual outcome of patients who had lower limb amputations due to peripheral vascular disease at Pelonomi Hospital, Bloemfontein. South African Family Practice. 2017;59(1):1-4.

154.	Dillingham T, Kenia J, Shofer F, Marschalek J. A prospective assessment of an adjustable, immediate fit, transtibial prosthesis. PM&R. 2019;11(11):1210-7.

155.	Kennedy GEM, McGarry K, Bradley G, Harkin DW. All-cause mortality amongst patients undergoing above and below knee amputation in a regional vascular centre within 2014-2015. The Ulster medical journal. 2019;88(1):30.

156.	Miller MJ, Cook PF, Kline PW, Anderson CB, Stevens‐Lapsley JE, Christiansen CL. Physical function and Pre‐Amputation characteristics explain daily step count after Dysvascular amputation. PM&R. 2019;11(10):1050-8.

157.	Sjödin L, Enocson A, Rotzius P, Lapidus LJ. Increased mortality among patients with diabetes following first-ever transfemoral amputation. Diabetes research and clinical practice. 2018;143:225-31.

158.	Orłowski M, Pluta P, Kowalczyk A, Kasprzak-Szczepańska K, Pająk M, Dziki A. Risk factors for impaired wound healing and prolonged hospitalization in patients after amputation of the lower limb above the knee joint. Polish Journal of Surgery. 2019;91(5):27-33.

159.	Murray AM, Gaffney BM, Davidson BS, Christiansen CL. Biomechanical compensations of the trunk and lower extremities during stepping tasks after unilateral transtibial amputation. Clinical Biomechanics. 2017;49:64-71.

160.	Anderson CB, Miller MJ, Murray AM, Fields TT, So NF, Christiansen CL. Falls after dysvascular transtibial amputation: a secondary analysis of falling characteristics and reduced physical performance. PM&R. 2021;13(1):19-29.

161.	Strelec J, Akeju OA, Gras LZ. Rehabilitation Program Using Short Prostheses after Bilateral Transfemoral Amputation: A Case Report. JPO: Journal of Prosthetics and Orthotics. 2021;33(3):234-40.

162.	Dodd S, Clarke M, Becker L, Mavergames C, Fish R, Williamson PR. A taxonomy has been developed for outcomes in medical research to help improve knowledge discovery. J Clin Epidemiol. 2018;96:84-92.
